# Chinese and global trends in pediatric spinal cord injury burden (1990–2021) with projections to 2045

**DOI:** 10.1007/s12519-025-00991-7

**Published:** 2025-11-05

**Authors:** You-Ping Tao, Chen-Hao Zhao, Ji-Gong Wu

**Affiliations:** Department of Spine Surgery, The Ninth Medical Center of Chinese PLA General Hospital, No. 9 Anxiangbeili, ChaoYang District, Beijing, 100101 China

**Keywords:** Burden, Global disease burden, Pediatrics, Projection analysis, Spinal cord injury

## Abstract

**Background:**

Spinal cord injury is a catastrophic medical condition and a growing global public health priority, with divergent etiologies, risk factors, and epidemiology. Comprehensive analyses of trends in pediatric spinal cord injury burden both in China and globally are lacking.

**Methods:**

We investigated temporal trends in pediatric spinal cord injury burden [incidence, prevalence, and years lived with disability (YLDs)] from 1990 to 2021 and projected future burden to 2045 using Global Burden of Disease (GBD) 2021 data. The year with the most significant changes in trends was identified via joinpoint regression analysis. Age-standardized rates, average annual percentage changes (AAPC), and subgroup analyses by injury location, sex, age, and sociodemographic index were calculated. To project burden data to 2045, we employed a Bayesian age‒period‒cohort model.

**Results:**

Our analyses revealed that global pediatric spinal cord injury incidence [AAPC: − 1.13; 95% confidence interval (CI): − 1.49 to − 0.76], prevalence (AAPC: − 1.13; 95% CI: − 1.16 to − 1.11), and YLDs rates (AAPC: − 1.37; 95% CI: − 1.41 to − 1.35) decreased significantly from 1990 to 2021. The highest burden was observed in males, adolescents (15–19 years), and high-sociodemographic index regions; falls were the leading cause. China exhibited similar declining trends (e.g., incidence AAPC: − 1.33; 95% CI: − 1.71 to − 0.83). It is predicted that by 2045, the global trend of pediatric spinal cord injury will decline; China will exhibit an upward trend.

**Conclusions:**

Persistent disparities in pediatric spinal cord injury disease burden and causes necessitate targeted prevention strategies, optimized rehabilitation services, and equitable resource allocation. This is particularly important in high-risk groups and regions with rising burdens.

**Graphical Abstract:**

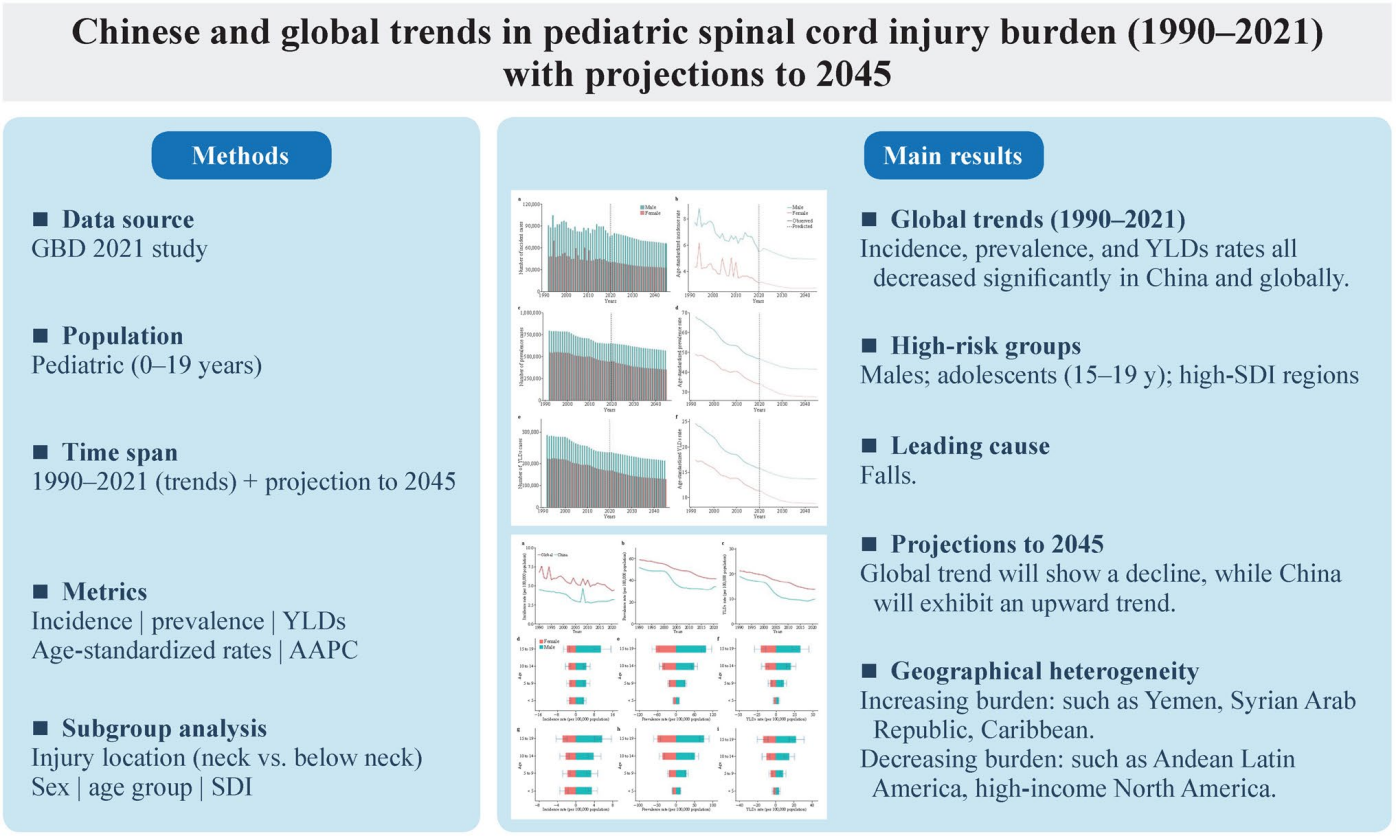

**Supplementary Information:**

The online version contains supplementary material available at 10.1007/s12519-025-00991-7.

## Introduction

Spinal cord injury (SCI) is a catastrophic medical condition and a growing global public health priority [1–7]. It imposes substantial physical, emotional, and socioeconomic burdens on individuals, families, and healthcare systems worldwide. Of note, an overall in-hospital mortality rate of 17.88% for traumatic cervical SCI has been recently described [8]. Furthermore, population-based data from the World Health Organization (WHO) indicate a global annual incidence of SCI ranging from 250,000 to 500,000 cases [9]. While SCI has traditionally been perceived as predominantly affecting middle-aged and elderly populations, its disproportionate impact on children and adolescents (aged 0–19 years) demands urgent attention.

A recent systematic review [10] quantified the global epidemiology of pediatric traumatic SCI, reporting an incidence of 4.3 cases per million children (aged 0–15 years) in developed nations. Critically, their meta-analysis revealed divergent etiological patterns across economic settings. Transport accidents constitute the primary cause in developed countries, whereas falls represent the leading cause in developing nations. Chien and colleagues [11] reported an overall pediatric SCI incidence rate (under 18 years) of about 5.99 per 100,000 person-years, with traumatic cervical injuries constituting the majority. Their study also identified lower socioeconomic status as a significant risk factor for SCI in children. De Vivo and Vogel [12] reported that complete injuries were most common in children aged 0–5 years, whereas incomplete injuries predominated in the 13- to 15-year age group. In addition, they described distinct epidemiological patterns, both within the pediatric population (across age groups) and between pediatric and adult SCI patients.

Despite these insights, comprehensive analyses of global temporal trends and healthcare utilization patterns, specifically among children and adolescents (aged 0–19 years) with SCI, remain critically deficient. This knowledge gap impedes the development of age-specific prevention initiatives, resource-appropriate rehabilitation frameworks, and global burden projection models.

The Global Burden of Disease, Injuries and Risk Factors Study (GBD) 2021 provides a comprehensive dataset offering valuable insights into the global burden of SCI [13–16]. Although several prior studies have described the burden of SCI, detailed data on the burden of pediatric SCI in both China and globally are unclear [17–28]. To address this issue, we used the GBD 2021 dataset to conduct an in depth, up-to-date analysis of the SCI burden within the 0- to 19-year-old demographic from 1990 to 2021. Specifically, we quantified age-standardized incidence, prevalence, and years lived with disability (YLDs) rates. We established temporal trajectories and projected disease evolution to 2045 to anticipate future healthcare demands. This disaggregated data serves as a foundation for the development of targeted prevention strategies and for the tailoring public health policies and interventions to address SCI within this pediatric population.

## Methods

### Data source and study population

The GBD 2021 comprehensively estimated the health burdens of 371 diseases, injuries, and impairments and 88 risk factors for 204 countries and territories from 1990 to 2021 [29, 30]. Metrics in GBD 2021 included 95% uncertainty intervals (UIs) that were calculated on the basis of the 25th and 975th ordered values of the 1000 estimates following the GBD algorithm. All rates are reported per 100,000 people. Furthermore, the sociodemographic index (SDI), which serves as a composite indicator of the total fertility rate in those under 25 years of age, mean education for those aged 15 years or older, and lag-distributed income per capita was calculated via GBD 2021 and used in this study. Each country or territory was categorized into one of five SDI quintiles: high, high-middle, middle, low-middle, and low [30]. According to WHO definitions and earlier publications [31], children and adolescents were defined as those aged 0–19 years and were further divided into four age subgroups: (1) < 5 years; (2) 5–9 years; (3) 10–14 years, and (4) 15–19 years.

### Statistical analysis

First, we investigated global trends in the incidence, prevalence, and YLDs rates of SCI among children and adolescents from 1990 to 2021. Average annual percentage change (AAPC), reflecting the trend over a prespecified fixed interval, was calculated as a weighted average of the annual percentage change (APC) by the span width of the segmented interval [32, 33]. AAPC between 1990 and 1999, between 2000 and 2009, and between 2010 and 2021 were also calculated. If the AAPC value and the lower boundary of the 95% confidence interval (CI) were both > 0, an increasing trend was deemed within this period. In contrast, if the AAPC value and the upper boundary of the 95% CI were both < 0, a decreasing trend was deemed within the period. If neither were true, the trend was deemed stable. Second, the year with the most significant changes in trends was identified via joinpoint regression analysis. This analysis approach fits the simplest model to the data by connecting several different line segments (i.e., joinpoints) on a logarithmic scale. Each added joinpoint was tested via a Monte Carlo permutation method [34, 35]. Third, global trends were stratified by injury location, sex, age subgroup, and SDI. Fourth, regional and national trends were investigated. Fifth, a log-linear age‒period‒cohort model that eliminates exponential growth and limits linear trend projection was utilized to predict the number of cases and rate of SCI burden among children and adolescents through 2045 [36, 37]. All statistical analyses and visualizations were performed via R software and the joinpoint regression program.

## Results

### Global trends

Global incidence of SCI among children and adolescents decreased from 6.79 (95% UI: 5.35–8.56) per 100,000 people in 1990 to 4.51 (95% UI: 3.51–5.85) per 100,000 people in 2021, with an AAPC of − 1.13 (95% CI: − 1.49 to − 0.76). Prevalence declined from 59.38 (95% UI: 51.64–71.14) per 100,000 people in 1990 to 41.87 (95% UI: 36.24–48.80) per 100,000 people in 2021, with an AAPC of − 1.13 (95% CI: − 1.16 to − 1.11). YLDs decreased from 21.47 (95% UI: 14.79–29.18) per 100,000 people in 1990 to 13.99 (95% UI: 9.52–18.62) per 100,000 people in 2021, reflecting an AAPC of − 1.37 (95% CI: − 1.41 to − 1.35) (Tables 1, 2, 3, 4).

**Table 1:** Global average annual percentage changes in incidence, prevalence, and years lived with disability of spinal cord injury among children and adolescents

Years	Incidence		Prevalence		YLDs	
	AAPC (95% CI)	*P*	AAPC (95% CI)	*P*	AAPC (95% CI)	*P*
1990–1999	− 1.13 (− 1.49 to − 0.76)	< 0.001	− 0.69 (− 0.76 to − 0.61)	< 0.001	− 0.87 (− 0.93 to − 0.79)	< 0.001
2000–2009	− 1.13 (− 1.49 to − 0.76)	< 0.001	− 1.37 (− 1.42 to − 1.27)	< 0.001	− 1.73 (− 1.79 to − 1.63)	< 0.001
2010–2021	− 1.13 (− 1.49 to − 0.76)	< 0.001	− 1.38 (− 1.46 to − 1.30)	< 0.001	− 1.56 (− 1.65 to − 1.50)	< 0.001
1990–2021	− 1.13 (− 1.49 to − 0.76)	< 0.001	− 1.13 (− 1.16 to − 1.11)	< 0.001	− 1.37 (− 1.41 to − 1.35)	< 0.001

*AAPC* average annual percentage change, *YLDs* years lived with disability, *CI* confidence interval. *P* values were calculated using the Monte Carlo permutation test

**Table 2:** Incidence of spinal cord injury and their average annual percentage changes from 1990 to 2021 among children and adolescents at global level, stratified by injury location, sex, age, and sociodemographic index

Characteristics	Case (*n*), 1990	Rate (per 100,000), 1990	Case (*n*), 2021	Rate (per 100,000), 2021	AAPC, 1990–2021	*P*
Global	153,382.42 (120,802.71–193,386.44)	6.79 (5.35–8.56)	118,859.70 (92,569.14–154,106.07)	4.51 (3.51–5.85)	− 1.13 (− 1.49 to − 0.76)	< 0.001
Injury location						
Spinal cord lesion at neck level	74,232.47 (55,019.84–101,795.20)	3.29 (2.44–4.51)	57,318.37 (42,413.63–79,515.53)	2.17 (1.61–3.02)	− 1.15 (− 1.5 to − 0.81)	< 0.001
Spinal cord lesion below neck level	79,149.95 (57,713.67–110,188.56)	3.50 (2.56–4.88)	61,541.33 (44,036.09–85,881.93)	2.33 (1.67–3.26)	− 1.09 (− 1.46 to − 0.72)	< 0.001
Sex						
Male	100,125.22 (78,942.46–125,284.33)	8.65 (6.82–10.82)	77,572.98 (60,838.76–99,398.42)	5.71 (4.48–7.32)	− 1.05 (− 1.34 to − 0.77)	< 0.001
Female	53,257.21 (41,189.88–68,657.41)	4.84 (3.74–6.23)	41,286.72 (30,994.71–55,096.27)	3.23 (2.43–4.31)	− 1.26 (− 1.68 to − 0.84)	< 0.001
Age (y)						
< 5	34,861.07 (28,124.67–43,350.66)	5.62 (4.54–6.99)	21,353.17 (16,955.27–26,920.41)	3.24 (2.58–4.09)	− 1.83 (− 2.18 to − 1.48)	< 0.001
5–9	31,338.05 (23,028.71–42,773.82)	5.37 (3.95–7.33)	25,277.53 (18,503.15–34,983.82)	3.68 (2.69–5.09)	− 1.04 (− 1.40 to − 0.7)	< 0.001
10–14	29,653.17 (22,190.06–40,103.35)	5.54 (4.14–7.49)	25,996.91 (19,074.29–35,779.49)	3.90 (2.86–5.37)	− 1.15 (− 1.42 to − 0.88)	< 0.001
15–19	57,530.14 (41,258.32–78,452.92)	11.08 (7.94–15.10)	46,232.09 (33,041.27–63,352.32)	7.41 (5.30–10.15)	− 1.21 (− 1.66 to − 0.93)	< 0.001
SDI						
High SDI	25,160.12 (19,635.30–32,058.03)	10.01 (7.81–12.76)	15,519.46 (11,731.36–20,796.41)	6.67 (5.04–8.94)	− 1.33 (− 1.37 to − 1.3)	< 0.001
High-middle SDI	26,458.81 (20,829.12–33,466.59)	7.15 (5.63–9.04)	15,565.61 (11,767.07–20,707.30)	5.13 (3.88–6.83)	− 1.08 (− 1.44 to − 0.88)	< 0.001
Middle SDI	45,907.28 (36,680.76–58,403.24)	6.00 (4.80–7.64)	27,170.73 (21,245.16–35,626.51)	3.63 (2.84–4.76)	− 1.67 (− 2.29 to − 1.09)	< 0.001
Low-middle SDI	30,176.85 (23,503.88–38,822.97)	5.11 (3.98–6.57)	25,942.70 (20,433.89–33,189.60)	3.39 (2.67–4.34)	− 1.55 (− 1.95 to − 1.14)	< 0.001
Low SDI	25,537.14 (15,772.60–41,148.68)	9.13 (5.64–14.72)	34,544.05 (23,066.64–53,504.95)	5.91 (3.95–9.16)	− 0.13 (− 1.20 to 0.98)	0.803

*AAPC* average annual percentage change, *SDI* sociodemographic index.* P* values were calculated using the Monte Carlo permutation test

**Table 3:** Prevalence of spinal cord injury and their average annual percentage changes from 1990 to 2021 among children and adolescents at the global level, stratified by injury location, sex, age, and sociodemographic index

Characteristics	Case (*n*), 1990	Rate (per 100,000), 1990	Case (*n*), 2021	Rate (per 100,000), 2021	AAPC, 1990–2021	*P*
Global	1,341,193.52 (1,166,252.20–1,606,812.70)	59.38 (51.64–71.14)	1,103,572.06 (955,328.24–1,286,261.40)	41.87 (36.24–48.80)	− 1.13 (− 1.16 to − 1.11)	< 0.001
Location						
Spinal cord lesion at neck level	629,145.94 (537,988.39–776,144.91)	27.86 (23.82–34.36)	520,737.54 (440,604.67–624,044.83)	19.76 (16.72–23.68)	− 1.11 (− 1.14 to − 1.08)	< 0.001
Spinal cord lesion below neck level	712,047.58 (609,642.49–881,462.62)	31.53 (26.99–39.03)	582,834.52 (500,578.84–691,456.33)	22.11 (18.99–26.23)	− 1.15 (− 1.17 to − 1.12)	< 0.001
Sex						
Male	806,284.65 (694,911.20–973,481.26)	69.66 (60.04–84.11)	653,619.87 (567,460.19–757,096.28)	48.11 (41.77–55.73)	− 1.16 (− 1.19 to − 1.14)	< 0.001
Female	534,908.87 (464,558.94–637,151.30)	48.58 (42.19–57.86)	449,952.18 (383,977.92–527,537.04)	35.23 (30.06–41.30)	− 1.05 (− 1.09 to − 1.00)	< 0.001
Age (y)						
< 5	92,951.35 (84,654.65–102,523.84)	14.99 (13.66–16.54)	61,235.92 (55,706.39–66,913.95)	9.30 (8.46–10.17)	− 1.56 (− 1.69 to − 1.39)	< 0.001
5–9	247,171.92 (217,423.31–289,106.74)	42.36 (37.26–49.54)	186,399.76 (164,278.79–212,071.21)	27.13 (23.91–30.87)	− 1.44 (− 1.49 to − 1.39)	< 0.001
10–14	398,337.82 (337,926.58–492,352.41)	74.36 (63.08–91.91)	345,850.37 (29,5046.65–409,057.73)	51.88 (44.26–61.36)	− 1.18 (− 1.21 to − 1.15)	< 0.001
15–19	602,732.43 (510,121.03–733,695.00)	116.04 (98.21–141.25)	510,086.01 (432,508.35–603,072.75)	81.75 (69.31–96.65)	− 1.12 (− 1.13 to − 1.10)	< 0.001
SDI						
High SDI	241,516.94 (212,355.62–271,950.17)	96.10 (84.50–108.21)	157,258.15 (136,548.14–179,013.41)	67.57 (58.67–76.92)	− 1.13 (− 1.15 to − 1.11)	< 0.001
High-middle SDI	266,816.97 (236,979.22–298,738.21)	72.08 (64.02–80.70)	155,950.51 (135,966.99–179,038.91)	51.41 (44.82–59.02)	− 1.07 (− 1.09 to − 1.04)	< 0.001
Middle SDI	419,976.79 (370,961.12–485,423.68)	54.93 (48.52–63.49)	307,878.07 (263,883.52–365,361.19)	41.09 (35.22–48.77)	− 0.90 (− 0.94 to − 0.87)	< 0.001
Low-middle SDI	255,918.10 (217,360.73–317,304.50)	43.30 (36.78–53.69)	243,127.20 (211,512.00–279,449.84)	31.81 (27.67–36.56)	− 0.99 (− 1.06 to − 0.91)	< 0.001
Low SDI	155,667.82 (102,294.94–280,517.98)	55.68 (36.59–100.34)	238,058.45 (191,228.56–320,143.29)	40.75 (32.73–54.80)	− 0.87 (− 0.98 to − 0.71)	< 0.001

*AAPC* average annual percentage change, *SDI* sociodemographic index. *P* values were calculated using the Monte Carlo permutation test

**Table 4:** Years lived with disability of spinal cord injury and their average annual percentage changes from 1990 to 2021 among children and adolescents at global level, stratified by injury location, sex, age, and sociodemographic index

Characteristics	Case (*n*), 1990	Rate (per 100,000), 1990	Case (*n*), 2021	Rate (per 100,000), 2021	AAPC, 1990–2021	*P*
Global	484,879.20 (334,146.36–659,102.24)	21.47 (14.79–29.18)	368,870.41 (250,919.37–490,710.38)	13.99 (9.52–18.62)	− 1.37 (− 1.41 to − 1.35)	< 0.001
Location						
Spinal cord lesion at neck level	281,727.75 (193,070.86–375,981.95)	12.47 (8.55–16.65)	224,874.18 (154,336.77–306,345.30)	8.53 (5.86–11.62)	− 1.22 (− 1.25 to − 1.20)	< 0.001
Spinal cord lesion below neck level	203,151.45 (135,549.56–292,913.59)	8.99 (6.00–12.97)	143,996.24 (96,887.39–206,254.19)	5.46 (3.68–7.82)	− 1.60 (− 1.64 to − 1.57)	< 0.001
Sex						
Male	293,900.43 (202,678.34–400,819.53)	25.39 (17.51–34.63)	220,425.52 (149,593.53–290,699.12)	16.23 (11.01–21.40)	− 1.46 (− 1.49 to − 1.42)	< 0.001
Female	190,978.76 (131,348.48–259,108.82)	17.34 (11.93–23.53)	148,444.89 (101,880.47–202,045.07)	11.62 (7.98–15.82)	− 1.30 (− 1.34 to − 1.24)	< 0.001
Age (y)						
< 5	34,935.07 (24,618.14–44,571.65)	5.64 (3.97–7.19)	21,396.00 (14,949.93–27,805.21)	3.25 (2.27–4.22)	− 1.86 (− 2.00 to − 1.71)	< 0.001
5–9	90,547.49 (63,221.52–119,007.87)	15.52 (10.83–20.39)	63,255.20 (44,288.90–84,920.10)	9.21 (6.45–12.36)	− 1.70 (− 1.76 to − 1.63)	< 0.001
10–14	143,918.93 (97,157.50–202,183.01)	26.87 (18.14–37.74)	115,289.60 (78,299.62–155,294.18)	17.29 (11.75–23.30)	− 1.43 (− 1.47 to − 1.40)	< 0.001
15–19	215,477.71 (148,027.43–299,303.67)	41.48 (28.50–57.62)	168,929.61 (112,966.17–232,189.00)	27.07 (18.10–37.21)	− 1.37 (− 1.39 to − 1.36)	< 0.001
SDI						
High SDI	73,655.08 (50,790.22–97,849.50)	29.31 (20.21–38.93)	45,684.47 (31,617.49–61,477.89)	19.63 (13.59–26.42)	− 1.29 (− 1.31 to − 1.26)	< 0.001
High-middle SDI	90,587.83 (63,451.15–118,344.81)	24.47 (17.14–31.97)	46,121.20 (31,484.86–62,064.64)	15.20 (10.38–20.46)	− 1.51 (− 1.54 to − 1.48)	< 0.001
Middle SDI	156,222.70 (107,737.75–202,584.16)	20.43 (14.09–26.50)	98,877.98 (66,693.00–132,963.81)	13.20 (8.90–17.75)	− 1.40 (− 1.43 to − 1.37)	< 0.001
Low-middle SDI	100,335.74 (68,266.63–136,321.84)	16.98 (11.55–23.07)	86,988.60 (59,584.82–113,870.11)	11.38 (7.80–14.90)	− 1.30 (− 1.36 to − 1.23)	< 0.001
Low SDI	63,625.24 (37,844.93–117,372.18)	22.76 (13.54–41.98)	90,750.61 (61,424.16–129,875.94)	15.53 (10.51–22.23)	− 1.09 (− 1.24 to − 0.90)	< 0.001

*YLDs* years lived with disability, *AAPC* average annual percentage change, *SDI* sociodemographic index. *P* values were calculated using the Monte Carlo permutation test

**Fig. 1:**
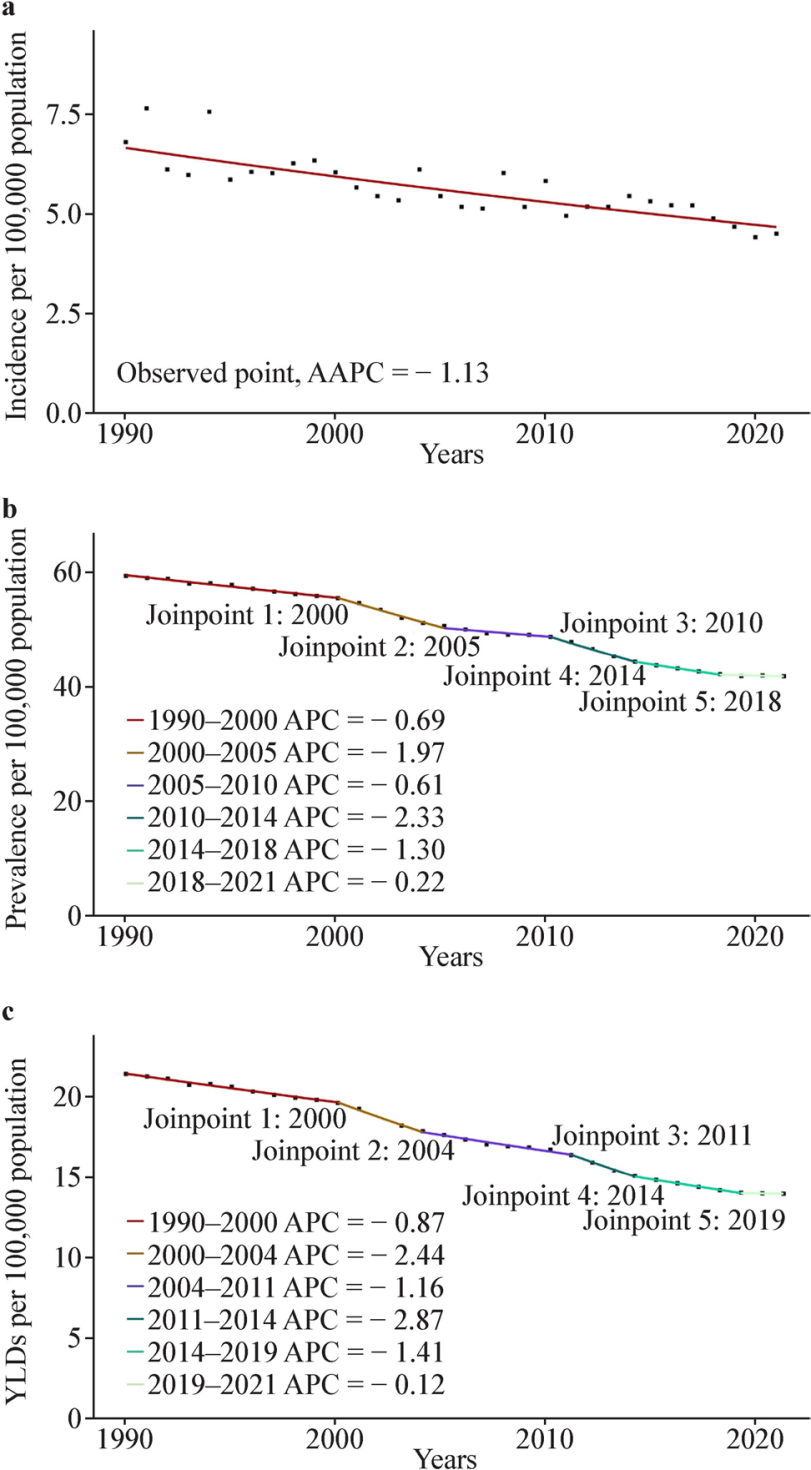
Jointpoint regression analysis of the global spinal cord injury burden among children and adolescents from 1990 to 2021. **a** Incidence; **b** prevalence; **c** YLDs. *YLDs* years lived with disability, *APC* annual percentage change, *AAPC* average annual percentage changes

The data show an overall downward trend in the global burden of SCI among children and adolescents from 1990 to 2021. Joinpoint regression analysis identified significant changes in pediatric SCI prevalence trends in 2000, 2005, 2010, 2014, and 2018. Joinpoint regression analyses of incidence, prevalence, and YLDs rates of pediatric SCI at the global level are presented in Fig. 1 and Supplementary Table 1.

### Global trends by injury location

Global incidence of SCI at the level of the neck decreased from 3.29 (95% UI: 2.44–4.51) per 100,000 people in 1990 to 2.17 (95% UI: 1.61–3.02) per 100,000 people in 2021, with an AAPC of − 1.15 (95% CI: − 1.50 to − 0.81). The incidence of SCI below the neck decreased from 3.50 (95% UI: 2.56–4.88) per 100,000 people in 1990 to 2.33 (95% UI: 1.67–3.26) per 100,000 people in 2021, with an AAPC of − 1.09 (95% CI: − 1.46 to − 0.72). Of note, the decline associated with neck-level SCI is faster than that associated with SCI below the neck. Moreover, neck-level SCI is associated with a greater burden of YLDs. Data on the incidence, prevalence, and YLDs rates of SCI and their AAPC stratified by injury location from 1990 to 2021 is shown in Tables 2, 3 and 4. Results of the joinpoint regression analyses are presented in Supplementary Table 2.

### Global trends by sex and age group

As shown in Tables 2, 3 and 4, from 1990 to 2021, global analysis data indicated that both sexes presented with decreased SCI incidence, prevalence, and YLDs. In addition, males have higher incidence rates, prevalence rates, and YLDs rates for SCI than females. Detailed joinpoint regression analysis data can be found in Supplementary Table 3.

A decline in incidence, prevalence, and YLDs was observed across all age groups. Peak burden for each metric was in the 15- to 19-year age group in 1990 and 2021. Of note, the under 5 years age group exhibited the fastest decrease in SCI incidence (AAPC: − 1.83; 95% CI: − 2.18 to − 1.48), prevalence (AAPC: − 1.56; 95% CI: − 1.69 to − 1.39), and YLDs (AAPC: − 1.86; 95% CI: − 2.00 to − 1.71). Results of the joinpoint regression analyses are presented in Supplementary Table 4.

### Global trends by sociodemographic index

In 2021, high-SDI countries presented with the highest incidence (6.67 per 100,000 people; 95% UI: 5.04 to 8.94), and the low-middle-SDI group presented the lowest incidence (3.39 per 100,000 people; 95% UI: 2.67 to 4.34). The fastest decrease in SCI incidence was found in the middle-SDI group (from 6.00 per 100,000 people in 1990 to 3.63 per 100,000 people in 2021), with an AAPC of − 1.67 (95% CI: − 2.29 to − 1.09). Collectively, all the SDI groups presented with decreasing trends in incidence, prevalence, and YLDs. The exception was for incidence in the low-SDI group. These data are presented in Tables 2, 3 and 4. Results of the joinpoint regression analyses are presented in Supplementary Table 5.

### Causes of pediatric spinal cord injury

The main causes of pediatric SCI among children and adolescents worldwide are falls, road injuries, conflicts, and terrorism; this is presented in Supplementary Tables 6‒8. Detailed joinpoint regression analysis of the incidence, prevalence, and YLDs of SCI from 1990 to 2021 among children and adolescents at the global level, stratified by injury causes, is presented in Supplementary Table 9.

### Regional trends

Regionally, the largest decreases in SCI incidence between 1990 and 2021 occurred in Andean Latin America (from 8.56 per 100,000 people in 1990 to 3.82 per 100,000 people in 2021), with an AAPC of − 2.77 (95% CI: − 3.03 to − 2.47). This was followed by high-income North America (from 9.44 per 100,000 people in 1990 to 5.22 per 100,000 people in 2021), with an AAPC of − 1.93 (95% CI: − 2.00 to − 1.86).

With respect to YLDs, Australasia presented the highest regional rate (41.32 per 100,000 people; 95% UI: 27.29–57.59) in 2021, whereas Southern Sub-Saharan Africa presented the lowest rate (8.95 per 100,000 people; 95% UI: 6.20–11.69). Furthermore, a decreasing trend was observed in most regions. Eastern Sub-Saharan Africa showed the fastest decline (from 25.84 per 100,000 people in 1990 to 10.76 per 100,000 people in 2021), with an AAPC of − 2.75 (95% CI: − 2.99 to − 2.45). However, the Caribbean presented a markedly increasing trend (from 15.68 per 100,000 people in 1990 to 38.76 per 100,000 people in 2021), with an AAPC of 2.88 (95% CI: 2.44–3.39). Regional incidence, prevalence, YLDs, and their corresponding AAPC values are detailed in Supplementary Tables 10–12. Joinpoint regression analysis of SCI for the 21 regions is presented in Supplementary Table 13.

### National trends

At the national level, SCI incidence in Yemen has markedly increased (from 4.55 per 100,000 people in 1990 to 26.81 per 100,000 people in 2021), with an AAPC of 5.67 (95% CI: 3.97–7.92). In 2021, Afghanistan had the highest national SCI incidence rate (49.35 per 100,000 people; 95% UI: 23.44–94.56), whereas Kiribati had the lowest incidence rate (1.69 per 100,000 people; 95% UI: 1.32–2.15).

In terms of prevalence, the Syrian Arab Republic exhibited a markedly increasing trend (from 49.89 per 100,000 people in 1990 to 448.10 per 100,000 people in 2021), with an AAPC of 7.57 (95% CI: 5.87–8.52). In 2021, the Syrian Arab Republic also had the highest SCI prevalence rate (448.10 per 100,000 people; 95% UI: 205.19–922.59), whereas Kiribati had the lowest prevalence rate (13.70 per 100,000 people; 95% UI: 12.08–15.38).

With respect to YLDs, the Syrian Arab Republic showed a markedly increasing trend (from 18.09 per 100,000 people in 1990 to 137.04 per 100,000 people in 2021), with an AAPC of 6.84 (95% CI: 5.48–7.71). In 2021, the Syrian Arab Republic also had the highest YLDs rate (137.04 per 100,000 people; 95% UI: 58.87–277.46), while the lowest was observed in Kiribati (5.55 per 100,000 people; 95% UI: 3.44–7.69). National-level incidence, prevalence, YLDs, and corresponding AAPC values are detailed in Supplementary Tables 14–16. Joinpoint regression analyses are presented in Supplementary Tables 17‒19.

In general, the pediatric SCI burden exhibited geographical heterogeneity at both national and regional levels.

### Relationships between incidence, prevalence, years lived with disability, and sociodemographic index

**Fig. 2:**
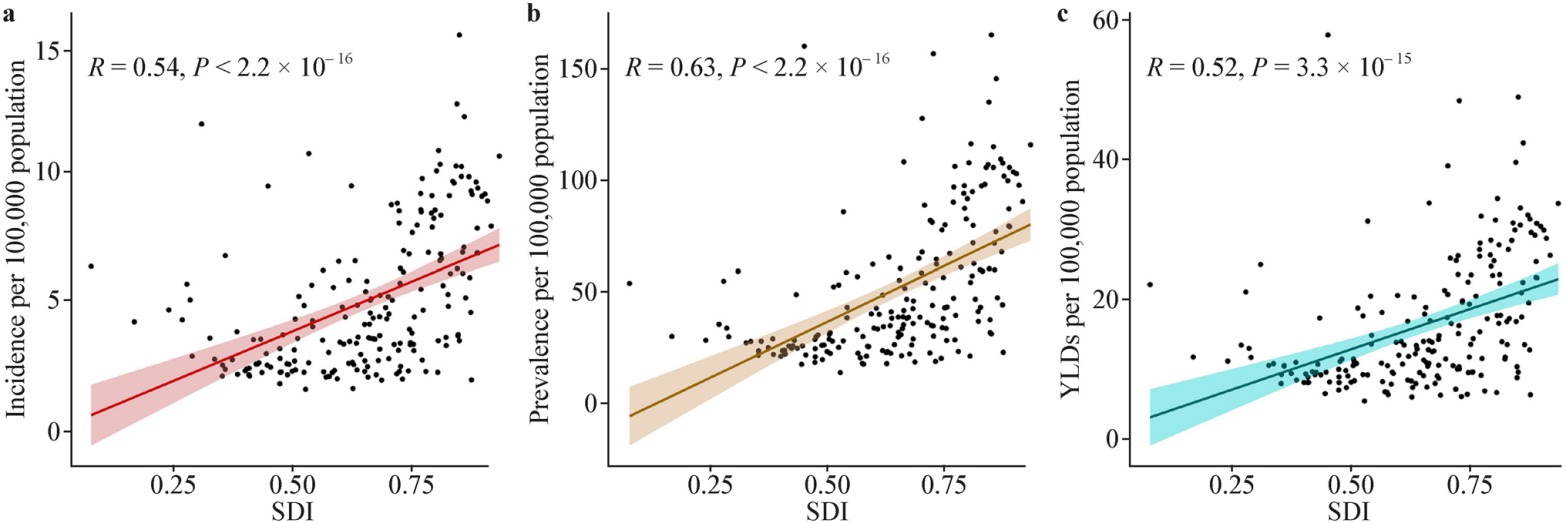
Correlation between national spinal cord injury burden and sociodemographic index among children and adolescents in 2021. **a** Incidence; **b** prevalence; **c** YLDs. *SDI* sociodemographic index, *YLDs* years lived with disability

As shown in Fig. 2, the incidence (*R* = 0.54, *P* < 2.2 × 10^–16^), prevalence (*R* = 0.63, *P* < 2.2 × 10^–16^), and YLDs (*R* = 0.52, *P* < 3.3 × 10^–15^) of pediatric SCI in 2021 were positively associated with the SDI. This suggests that countries with higher SDI should face greater burdens of pediatric SCI.

### Projections of the global burden to 2045

**Fig. 3:**
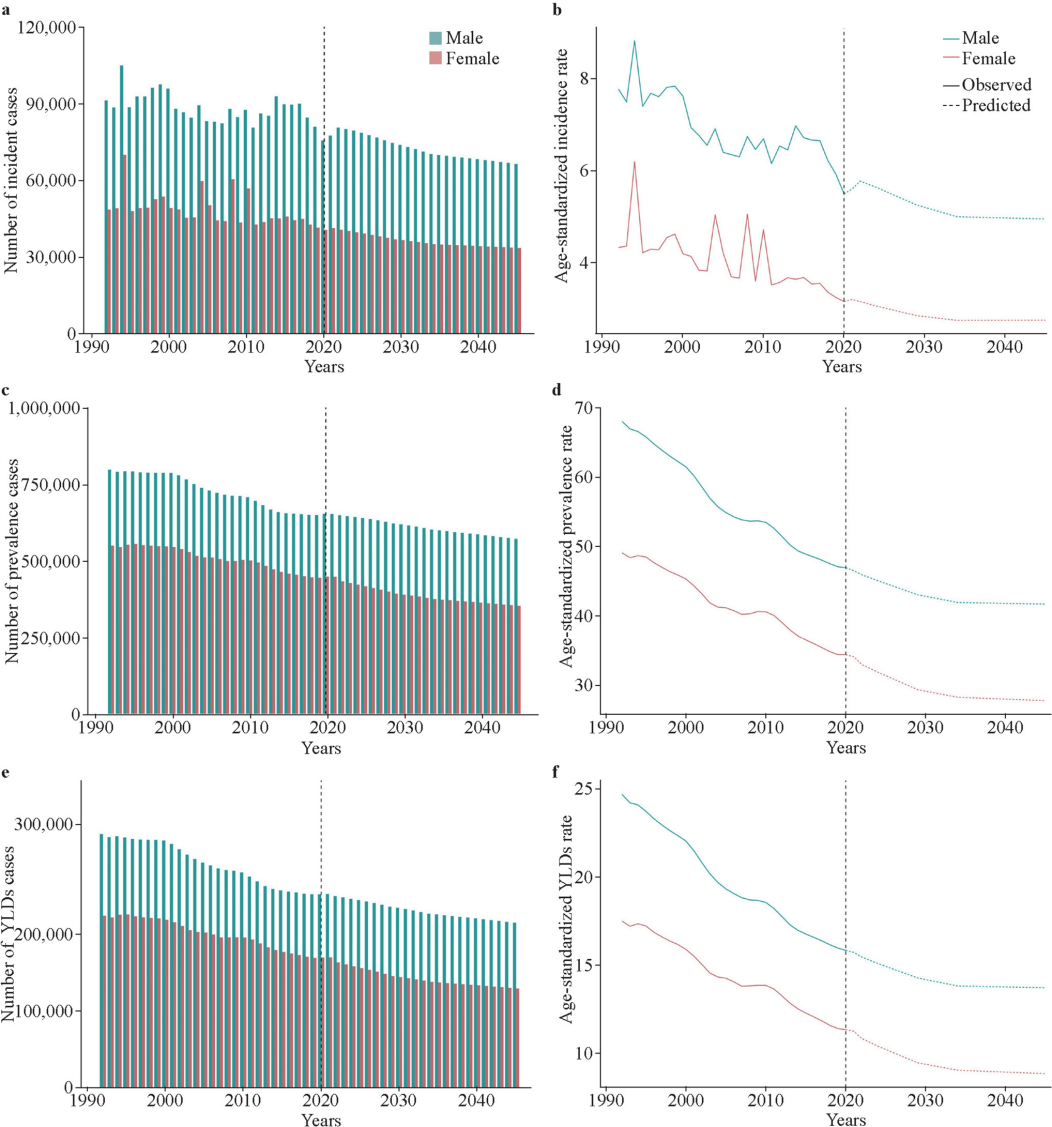
Global predicted changes in the case numbers and age-standardized rate of the spinal cord injury burden among children and adolescents to 2045. **a** Number of incident cases; **b** age-standardized incidence rate (per 100,000 people); **c** number of prevalent cases; **d** age-standardized prevalence rate (per 100,000 people); **e** number of YLDs cases; **f** age-standardized YLDs rate. *YLDs* years lived with disability

Forecast analysis indicated that the burden of SCI among children and adolescents is projected to decrease globally for both males and females by 2045 (Fig. 3). The projected number of SCI cases, incidence, prevalence, and YLDs rates of SCI to 2045 are presented in Supplementary Tables 20–21.

### Epidemiological characteristics and trends of pediatric spinal cord injury in China

**Fig. 4:**
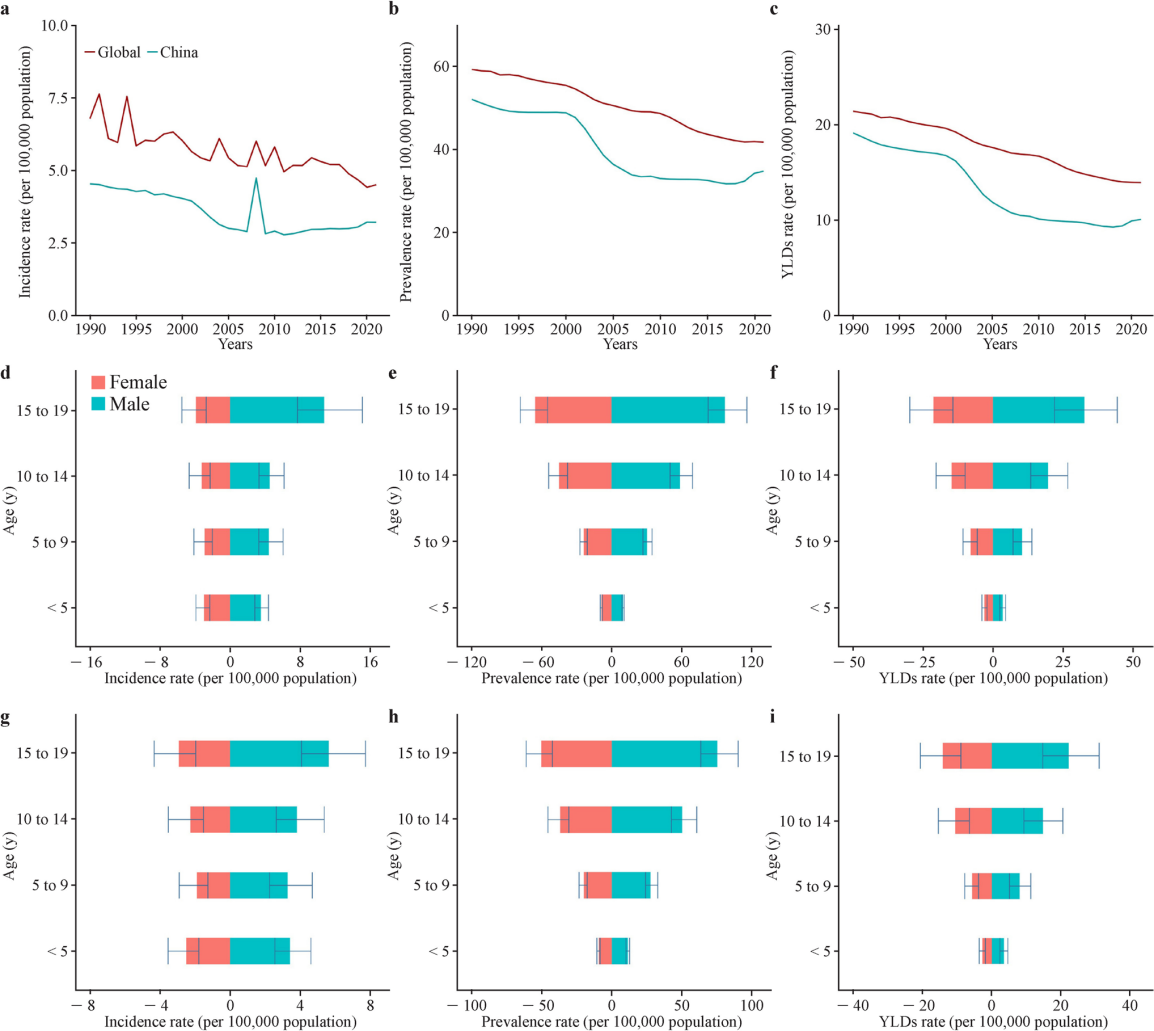
Comparison of the burden of spinal cord injury among children and adolescents in China and globally. **a** Incidence rate of SCI from 1990 to 2021 globally and in China; **b** prevalence rates of SCI from 1990 to 2021 globally and in China; **c** YLDs rates of SCI from 1990 to 2021 globally and in China; **d** sex distribution of the incidence rate of SCI at the global level in 2021; **e** sex distribution of the prevalence rate of SCI at the global level in 2021; **f** sex distribution of the YLDs rate of SCI at the global level in 2021; **g** sex distribution of the incidence rate of SCI in China in 2021; **h** sex distribution of the prevalence rate of SCI in China in 2021; **i** sex distribution of the YLDs rate of SCI in China in 2021. *SCI* spinal cord injury, *YLDs* years lived with disability

In 2021, there were 10,773.59 (95% UI: 8142.92–14,518.75) incident cases, 116,642.53 (95% UI: 101,054.42–136,259.91) prevalent cases, and 33,874.54 (95% UI: 22,733.04–46,017.78) YLDs. Our analysis revealed an overall downward trend in the burden of pediatric SCI from 1990 to 2021 in China (Fig. 4 and Supplementary Tables 14–16).

The incidence of pediatric SCI decreased from 4.54 (95% UI: 3.57–5.92) per 100,000 people in 1990 to 3.22 (95% UI: 2.44–4.34) per 100,000 people in 2021, with an AAPC of − 1.33 (95% CI: − 1.71 to − 0.83). Prevalence declined from 52.16 (95% UI: 46.37–58.89) per 100,000 people in 1990 to 34.89 (95% UI: 30.23–40.76) per 100,000 people in 2021, with an AAPC of − 1.28 (95% CI: − 1.34 to − 1.22). YLDs decreased from 19.19 (95% UI: 13.35–24.76) per 100,000 people in 1990 to 10.13 (95% UI: 6.80–13.77) per 100,000 people in 2021, with an AAPC of − 2.03 (95% CI: − 2.09 to − 1.97).

Joinpoint regression analysis identified significant changes in pediatric SCI prevalence trends in 1993, 2004, 2007, and 2018. Results of joinpoint regression analysis of SCI in China are presented in Supplementary Tables 17‒19.

In addition, males have higher incidence, prevalence, and YLDs rates for SCI than females in China. Comparison of the burden of SCI among children and adolescents in China and globally are presented in Fig. 4. Falls, road injuries, and interpersonal violence were identified as the main causes of the incidence, prevalence, and YLDs of the pediatric SCI in China in 2021 (Supplementary Table 22).

Projections to 2045 indicate an upward trend for pediatric SCI in China (Supplementary Fig. 1 and Supplementary Table 23).

## Discussion

SCI is a catastrophic medical condition and a significant global public health challenge. However, current understanding of its burden among children and adolescents is limited. Current and comprehensive estimates of the SCI burden in the pediatric population (aged 0–19 years) are urgently needed to inform healthcare planning, resource allocation, and prevention strategies aimed at reducing its impact in this vulnerable group. Using the GBD 2021 dataset, this study provides an in-depth and comprehensive analysis of changing trends in the SCI burden among children and adolescents (aged 0–19 years) at global, regional, and national levels from 1990 to 2021 along with projections to 2045.

Our analysis revealed a downward trend in the burden of SCI among children and adolescents from 1990 to 2021 in China and globally. The incidence, prevalence, and YLDs rates significantly decreased for both males and females. This decline is likely attributable to several synergistic factors. Global improvements in access to advanced healthcare systems and the implementation of targeted intervention strategies over recent decades have played crucial roles. In summary, the convergence of advancements in healthcare access, transportation safety innovation, injury prevention programs and targeted behavioral education provides a compelling explanation for the declining SCI burden observed in this pediatric population during the study period.

This study revealed significant geographical heterogeneity in the burden of pediatric SCI at both national and regional levels. For example, the marked increasing trends observed in the Caribbean may potentially relate to road traffic accidents, violence, or natural disasters in the context of limited preventive infrastructure. In nations like Yemen and the Syrian Arab Republic, increases may result from the impact of prolonged armed conflict and terrorism on healthcare systems, infrastructure, and population safety. Significant decreasing trends in regions like Andean Latin America and high-income North America could reflect improvements in trauma care, road safety legislation, fall prevention programs, and advanced healthcare systems. Therefore, targeted interventions must be tailored to the specific socioeconomic and geopolitical context of a region or country, making our findings more actionable for policymakers [10, 16, 18].

This study revealed a significant effect of age on the SCI burden within this population. Peak rates for incidence, prevalence and YLDs consistently occurred among individuals aged 15–19 years in both 1990 and 2021. This finding aligns with previous research indicating that adolescents and young adults are significantly more likely to sustain SCI than preschool-aged children [11, 38]. This pronounced burden among adolescents likely stems from their increased levels of activity, mobility and participation in higher-risk, high-velocity sports compared with infants and younger children. Collectively, these findings underscore the critical need for age-specific public health interventions. Tailored strategies, such as school-based spinal cord health education programs targeting adolescents, are urgently needed.

We found higher SCI incidence, prevalence, and YLDs rates among males than females, both in China and globally. This finding aligns with prior GBD studies [15, 18] and is corroborated by Chien et al. [11], who reported that male children had a significantly greater likelihood of SCI than females. This disparity is likely attributable to a greater participation in activities associated with higher SCI risk in males. Consequently, these findings underscore the critical need for increased focus on and the development of gender-specific public health intervention strategies targeting children and adolescents [39, 40].

In this study, falls were the leading cause of SCI among children and adolescents. Other common causes included road injuries, conflict and terrorism. Several studies have reported similar results [13, 17, 18]. Context-specific interventions prove effective, as illustrated by the work of Hoque et al. [41], demonstrating how safe tree-climbing education significantly reduced fall-related SCI. Furthermore, the WHO's 2004 global report on preventing road traffic injuries stressed that legislation must address three areas: regulating speed and alcohol use, enforcing seatbelt and helmet compliance and ensuring safer road infrastructure and vehicle design [42]. A study by Rasouli et al. [43] underscores the critical impact of prevention and education in reducing motor vehicle crash-related morbidity and mortality. These findings indicate that enhancing safer road user behavior, improving infrastructure and implementing vehicle safety innovations, such as enforced seat belt usage and airbag deployment, can substantially decrease motor vehicle crash-related SCI incidence in children. Given this diverse etiology, targeted prevention strategies for children and adolescents are paramount. A deeper understanding of these causative factors is essential for developing effective interventions, such as enacting universal helmet laws and vehicle safety standards for primary prevention and enforcing seatbelt use, to reduce trauma related mortality [43, 44].

Our analysis revealed higher YLDs rates for SCI at the level of the neck than below the neck. This finding is consistent with prior GBD 2019 studies [18, 22]. Of note, SCI at the level of the neck warrants special emphasis because of its association with higher mortality rates, increased morbidity and greater long-term disability [7, 12, 45–48].

The current study revealed that there is a significantly greater burden of SCI in high-SDI regions. These elevated incidence, prevalence and YLDs rates likely reflect a complex interplay of factors: advanced diagnostics improving detection and emergency treatments, higher survival rates increasing the prevalence of long-term disability, greater engagement in high-risk behaviors and comprehensive healthcare systems enabling long-term management. In contrast, low-SDI regions may substantially underestimate the SCI burden due to data collection gaps, limited diagnostic capabilities and increased acute-phase mortality, preventing long-term disability registration. These clear disparities underscore the urgent need for SDI-stratified interventions. Collectively, targeted resource allocation and policy action are imperative to address these inequities [18].

This study specifically projected the burden of SCI among children and adolescents to 2045. Our findings indicated that the absolute burden of pediatric SCI is substantial. Moreover, while a declining global trend in pediatric SCI is projected, an upward trend is anticipated in China. These projections provide critical, granular data to inform national health policy formulation. Nevertheless, the sustained implementation of effective, evidence-based prevention strategies is paramount to further reduce the burden of SCI in this vulnerable population.

A major strength of this study lies in its comprehensive analysis, providing a disaggregated assessment of the pediatric SCI burden at the global, regional and national levels over three decades. We believe that these findings will contribute to advancing our understanding of pediatric SCI epidemiology and underscore an urgent need to implement targeted prevention strategies, optimize disability management, improve clinical outcomes for the pediatric population and advance SCI research [2, 49, 50].

Several limitations should be acknowledged. First, the GBD data used in this study relied heavily on modeled data; estimate accuracy remains dependent on the quality and quantity of underlying source data. Estimates for regions with underdeveloped healthcare systems, limited surveillance, or active conflict are associated with greater uncertainty and may lead to an underestimation of the true burden and potentially affect cross-country comparisons and trend interpretations. Second, current projections are based on historical trends and do not account for future changes in healthcare infrastructure, policy interventions, or prevention programs that may alter the trajectory of SCI burden. Third, this study is primarily epidemiological and does not directly propose specific clinical or preventive interventions. Our findings highlight areas of need; further translational research is required to develop and implement firm action plans. Fourth, owing to lack of data, subnational trends cannot be further investigated.

In conclusion, these findings represent a pivotal epidemiological foundation to guide evidence-driven prioritization of healthcare investments and accelerate the adoption of precision prevention protocols tailored to children and adolescents aged 0–19 years with SCI. However, despite these advances, the development of innovative preventive modalities and targeted therapeutic interventions must be prioritized within translational pipelines to mitigate the disproportionate burden of disability in this special cohort. These evidence-based insights bridge a persisting knowledge gap in epidemiology, transforming empirical data into actionable strategies for clinical practice and health economic planning.

## Supplementary Information

Below is the link to the electronic supplementary material.

Supplementary file1 (PDF 297 kb)

Supplementary file2 (PDF 3818 kb)

## Data Availability

Datasets generated and analyzed are available from the corresponding author upon reasonable request.
