# Supplementary material for: Chinese and global trends in pediatric spinal cord injury burden (1990–2021) with projections to 2045
Source: World J Pediatr. 2025 Nov 5;21(12):1275–88. doi: 10.1007/s12519-025-00991-7 (PMC12678510; doi:10.1007/s12519-025-00991-7)
Supplement: Supplementary file 1 — Supplementary file1 (PDF 297 kb) [file 12519_2025_991_MOESM1_ESM.pdf]

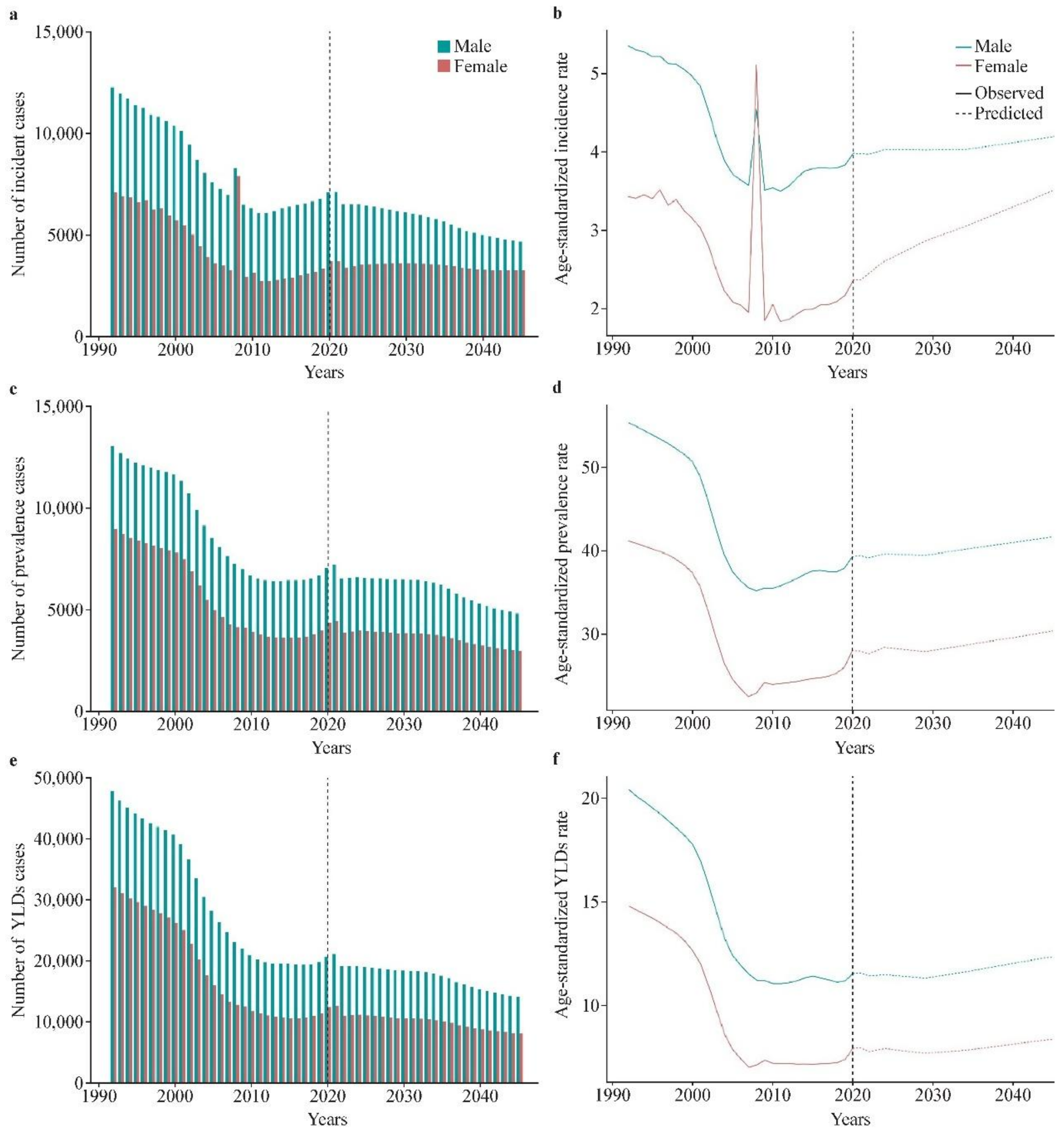

Supplementary Fig. 1 The case number and ASR of the spinal cord injury burden among children and adolescents in China are projected through 2045. **a** Number of incident cases; **b** Age-standardized incidence rate; **c** Number of prevalent cases; **d** Age-standardized prevalence rate; **e** Number of YLDs cases; **f** Age-standardized YLDs rate.

ASR age-standardized rate, *YLDs* years lived with disability
