# Supplementary material for: Chinese and global trends in pediatric spinal cord injury burden (1990–2021) with projections to 2045
Source: World J Pediatr. 2025 Nov 5;21(12):1275–88. doi: 10.1007/s12519-025-00991-7 (PMC12678510; doi:10.1007/s12519-025-00991-7)
Supplement: Supplementary file 2 — Supplementary file2 (PDF 3818 kb) [file 12519_2025_991_MOESM2_ESM.pdf]

Supplementary Table 1 Joinpoint regression analysis of incidence, prevalence, and YLDs of spinal cord injury from 1990 to 2021 among children and adolescents at global level

Measure	Year	APC (95% CI)	*P*
Incidence	1990-2021	-1.13 (-1.49 to -0.76)	<0.001
Prevalence	1990-2000	-0.69 (-0.77 to -0.6)	0.004
Prevalence	2000-2005	-1.97 (-2.24 to -1.69)	<0.001
Prevalence	2005-2010	-0.61 (-0.91 to -0.35)	0.006
Prevalence	2010-2014	-2.33 (-2.64 to -1.89)	0.001
Prevalence	2014-2018	-1.3 (-1.98 to -0.94)	<0.001
Prevalence	2018-2021	-0.22 (-0.62 to 0.35)	0.282
YLDs	1990-2000	-0.87 (-0.94 to -0.79)	<0.001
YLDs	2000-2004	-2.44 (-2.8 to -2.05)	<0.001
YLDs	2004-2011	-1.16 (-1.3 to -0.99)	<0.001
YLDs	2011-2014	-2.87 (-3.09 to -2.27)	<0.001
YLDs	2014-2019	-1.41 (-1.87 to -1.17)	<0.001
YLDs	2019-2021	-0.12 (-0.84 to 0.3)	0.468

YLDs years lived with disability, *APC* annual percentage change, *CI* confidence interval.

Supplementary Table 2 Joinpoint regression analysis of incidence, prevalence, and YLDs of spinal cord injury from 1990 to 2021 among children and adolescents at global level, stratified by injury location

Measure	Injury location	Year	APC (95% CI)	*P*
Incidence	Spinal cord lesion at neck level	1990-2021	-1.15 (-1.5 to -0.81)	<0.001
Incidence	Spinal cord lesion below neck level	1990-2021	-1.09 (-1.46 to -0.72)	<0.001
Prevalence	Spinal cord lesion at neck level	1990-2000	-0.67 (-0.77 to -0.58)	0.009
Prevalence	Spinal cord lesion at neck level	2000-2004	-2.01 (-2.36 to -0.72)	0.0003
Prevalence	Spinal cord lesion at neck level	2004-2011	-0.85 (-1.93 to -0.66)	<0.001
Prevalence	Spinal cord lesion at neck level	2011-2014	-2.66 (-2.89 to -0.85)	<0.001
Prevalence	Spinal cord lesion at neck level	2014-2018	-1.21 (-2.47 to -0.9)	<0.001
Prevalence	Spinal cord lesion at neck level	2018-2021	-0.25 (-0.68 to 0.32)	0.250
Prevalence	Spinal cord lesion below neck level	1990-2000	-0.68 (-0.76 to -0.6)	0.003
Prevalence	Spinal cord lesion below neck level	2000-2005	-2.05 (-2.3 to -1.78)	<0.001
Prevalence	Spinal cord lesion below neck level	2005-2010	-0.63 (-0.88 to -0.4)	0.005
Prevalence	Spinal cord lesion below neck level	2010-2014	-2.33 (-2.62 to -1.95)	0.0003
Prevalence	Spinal cord lesion below neck level	2014-2018	-1.31 (-1.92 to -0.95)	<0.001
Prevalence	Spinal cord lesion below neck level	2018-2021	-0.21 (-0.6 to 0.36)	0.313
YLDs	Spinal cord lesion at neck level	1990-2000	-0.76 (-0.83 to -0.69)	0.0007
YLDs	Spinal cord lesion at neck level	2000-2004	-2.2 (-2.54 to -1.83)	<0.001
YLDs	Spinal cord lesion at neck level	2004-2011	-0.97 (-1.11 to -0.8)	<0.001
YLDs	Spinal cord lesion at neck level	2011-2014	-2.74 (-2.97 to -2.13)	<0.001

YLDs	Spinal cord lesion at neck level	2014-2019	-1.26 (-1.74 to -1.03)	<0.001
YLDs	Spinal cord lesion at neck level	2019-2021	-0.01 (-0.75 to 0.41)	0.823
YLDs	Spinal cord lesion below neck level	1990-2000	-1.03 (-1.13 to -0.94)	0.0003
YLDs	Spinal cord lesion below neck level	2000-2005	-2.59 (-2.93 to -2.23)	<0.001
YLDs	Spinal cord lesion below neck level	2005-2011	-1.33 (-2 to -1.06)	<0.001
YLDs	Spinal cord lesion below neck level	2011-2014	-3.12 (-3.36 to -1.42)	<0.001
YLDs	Spinal cord lesion below neck level	2014-2019	-1.66 (-2.68 to -1.38)	<0.001
YLDs	Spinal cord lesion below neck level	2019-2021	-0.3 (-1.15 to 0.15)	0.184

YLDs years lived with disability, *APC* annual percentage change, *CI* confidence interval.

Supplementary Table 3 Joinpoint regression analysis of incidence, prevalence, and YLDs of spinal cord injury from 1990 to 2021 among children and adolescents at global level, stratified by sex

Measure	Sex	Year	APC (95% CI)	*P*
Incidence	Female	1990-2021	-1.26 (-1.68 to -0.84)	<0.001
Incidence	Male	1990-2021	-1.05 (-1.34 to -0.77)	<0.001
Prevalence	Female	1990-1994	0.25 (-0.12 to 0.93)	0.160
Prevalence	Female	1994-2000	-0.76 (-1.09 to -0.5)	<0.001
Prevalence	Female	2000-2004	-2.11 (-2.63 to -1.68)	0.002
Prevalence	Female	2004-2010	-0.57 (-0.84 to -0.08)	0.037
Prevalence	Female	2010-2017	-2.17 (-2.51 to -1.95)	<0.001
Prevalence	Female	2017-2021	-0.43 (-0.86 to 0.23)	0.122
Prevalence	Male	1990-2001	-0.9 (-0.96 to -0.84)	<0.001
Prevalence	Male	2001-2005	-2.13 (-2.36 to -1.85)	<0.001
Prevalence	Male	2005-2010	-0.76 (-0.93 to -0.59)	<0.001
Prevalence	Male	2010-2014	-2.27 (-2.5 to -1.98)	<0.001
Prevalence	Male	2014-2018	-1.01 (-1.6 to -0.79)	<0.001
Prevalence	Male	2018-2021	-0.23 (-0.54 to 0.25)	0.230
YLDs	Female	1990-1994	0.1 (-0.3 to 0.89)	0.583
YLDs	Female	1994-2000	-1 (-1.37 to -0.71)	<0.001
YLDs	Female	2000-2004	-2.41 (-2.99 to -1.91)	0.000
YLDs	Female	2004-2010	-0.87 (-1.17 to -0.21)	0.023

YLDs	Female	2010-2018	-2.33 (-2.67 to -2.14)	<0.001
YLDs	Female	2018-2021	-0.32 (-1.02 to 0.78)	0.289
YLDs	Male	1990-1993	-1.67 (-2.26 to -1.19)	<0.001
YLDs	Male	1993-2000	-0.88 (-1.04 to -0.51)	<0.001
YLDs	Male	2000-2005	-2.33 (-2.71 to -2.05)	<0.001
YLDs	Male	2005-2010	-1.11 (-1.37 to -0.66)	<0.001
YLDs	Male	2010-2015	-2.34 (-2.72 to -2.1)	<0.001
YLDs	Male	2015-2021	-0.83 (-1.01 to -0.58)	0.000

YLDs years lived with disability, *APC* annual percentage change, *CI* confidence interval.

Supplementary Table 4 Joinpoint regression analysis of incidence, prevalence, and YLDs of spinal cord injury from 1990 to 2021 among children and adolescents at global level, stratified by age

Measure	Age (y)	Year	APC (95% CI)	*P*
Incidence	0 to 4	1990-2021	-1.83 (-2.18 to -1.48)	<0.001
Incidence	5 to 9	1990-2021	-1.04 (-1.4 to -0.7)	<0.001
Incidence	10 to 14	1990-2021	-1.15 (-1.42 to -0.88)	<0.001
Incidence	15 to 19	1990-2011	-1.5 (-2.16 to -1.08)	0.011
Incidence	15 to 19	2011-2015	6 (0.82 to 11.1)	0.022
Incidence	15 to 19	2015-2021	-4.77 (-9.74 to -2.37)	0.006
Prevalence	0 to 4	1990-1993	3.57 (1.15 to 7.33)	0.006
Prevalence	0 to 4	1993-1997	-5.39 (-7.31 to -3.61)	<0.001
Prevalence	0 to 4	1997-2004	-1.74 (-2.88 to -0.02)	0.047
Prevalence	0 to 4	2004-2011	0.74 (-5.21 to 3.67)	0.118
Prevalence	0 to 4	2011-2014	-7.05 (-8.45 to -2.27)	0.024
Prevalence	0 to 4	2014-2021	-1.12 (-1.91 to 0.4)	0.082
Prevalence	5 to 9	1990-1998	-0.69 (-0.95 to -0.41)	0.001
Prevalence	5 to 9	1998-2003	-3.15 (-4.02 to -2.63)	<0.001
Prevalence	5 to 9	2003-2012	-0.41 (-0.62 to -0.09)	0.022
Prevalence	5 to 9	2012-2021	-2.17 (-2.43 to -1.97)	<0.001
Prevalence	10 to 14	1990-2001	-1.15 (-1.24 to -1.04)	<0.001
Prevalence	10 to 14	2001-2006	-2.01 (-2.54 to -1.74)	<0.001

Prevalence	10 to 14	2006-2011	-0.43 (-0.71 to -0.02)	0.046
Prevalence	10 to 14	2011-2015	-2.58 (-3.05 to -2.23)	<0.001
Prevalence	10 to 14	2015-2021	-0.21 (-0.41 to 0)	0.054
Prevalence	15 to 19	1990-1996	-0.58 (-0.69 to -0.35)	0.010
Prevalence	15 to 19	1996-2000	-0.91 (-2.53 to -0.78)	<0.001
Prevalence	15 to 19	2000-2005	-2.53 (-2.63 to -0.37)	<0.001
Prevalence	15 to 19	2005-2010	-0.31 (-1.8 to -0.21)	0.000
Prevalence	15 to 19	2010-2013	-2.02 (-2.15 to -1.13)	<0.001
Prevalence	15 to 19	2013-2021	-0.91 (-0.97 to -0.8)	<0.001
YLDs	0 to 4	1990-1994	1.75 (0.5 to 3.75)	0.012
YLDs	0 to 4	1994-1997	-7.25 (-8.39 to -4.85)	<0.001
YLDs	0 to 4	1997-2007	-1.38 (-2.05 to -0.85)	0.001
YLDs	0 to 4	2007-2011	1.82 (0.3 to 3.74)	0.023
YLDs	0 to 4	2011-2014	-8.21 (-9.49 to -5.51)	0.002
YLDs	0 to 4	2014-2021	-1.45 (-2.11 to -0.4)	0.021
YLDs	5 to 9	1990-1998	-0.87 (-1.15 to -0.53)	0.001
YLDs	5 to 9	1998-2003	-3.38 (-4.54 to -2.77)	<0.001
YLDs	5 to 9	2003-2013	-0.73 (-0.95 to -0.4)	0.004
YLDs	5 to 9	2013-2021	-2.65 (-3 to -2.36)	<0.001

YLDs	10 to 14	1990-2001	-1.37 (-1.47 to -1.25)	<0.001
YLDs	10 to 14	2001-2007	-2.2 (-2.7 to -2.01)	<0.001
YLDs	10 to 14	2007-2010	-0.15 (-0.8 to 0.17)	0.301
YLDs	10 to 14	2010-2016	-2.36 (-2.7 to -2.2)	<0.001
YLDs	10 to 14	2016-2021	-0.26 (-0.56 to 0.17)	0.086
YLDs	15 to 19	1990-2000	-0.89 (-0.94 to -0.83)	<0.001
YLDs	15 to 19	2000-2005	-2.98 (-3.07 to -2.89)	<0.001
YLDs	15 to 19	2005-2010	-0.61 (-0.72 to -0.49)	0.000
YLDs	15 to 19	2010-2013	-2.15 (-2.32 to -1.78)	<0.001
YLDs	15 to 19	2013-2021	-1.14 (-1.21 to -1.03)	<0.001

YLDs years lived with disability, *APC* annual percentage change, *CI* confidence interval.

Supplementary Table 5 Joinpoint regression analysis of incidence, prevalence, and YLDs of spinal cord injury from 1990 to 2021 among children and adolescents at global level, stratified by SDI

Measure	SDI category	Year	APC (95% CI)	*P*
Incidence	High SDI	1990-1996	-0.83 (-1.06 to -0.51)	0.001
Incidence	High SDI	1996-2000	-2.41 (-2.98 to -1.95)	<0.001
Incidence	High SDI	2000-2011	-0.85 (-0.95 to -0.71)	<0.001
Incidence	High SDI	2011-2014	-3.57 (-3.91 to -2.74)	<0.001
Incidence	High SDI	2014-2021	-0.94 (-1.13 to -0.69)	<0.001
Incidence	High-middle SDI	1990-1992	4.04 (-1.33 to 7.4)	0.248
Incidence	High-middle SDI	1992-2021	-1.42 (-2.08 to -1.22)	0.030
Incidence	Middle SDI	1990-1994	-4.87 (-13.68 to -0.2)	0.029
Incidence	Middle SDI	1994-2016	0.74 (0.22 to 5.81)	0.016
Incidence	Middle SDI	2016-2021	-9.24 (-17.25 to -4.12)	0.002
Incidence	Low-middle SDI	1990-2021	-1.55 (-1.95 to -1.14)	<0.001
Incidence	Low SDI	1990-2021	-0.13 (-1.2 to 0.98)	0.803
Prevalence	High SDI	1990-1996	-0.64 (-0.72 to -0.55)	<0.001
Prevalence	High SDI	1996-2000	-2.26 (-2.36 to -2.15)	<0.001
Prevalence	High SDI	2000-2010	-0.46 (-0.51 to -0.42)	<0.001
Prevalence	High SDI	2010-2015	-2.68 (-2.84 to -2.59)	<0.001
Prevalence	High SDI	2015-2018	-1.37 (-2.02 to -0.97)	<0.001
Prevalence	High SDI	2018-2021	0.01 (-0.25 to 0.5)	0.906

Prevalence	High-middle SDI	1990-1995	0.04<br>(-0.1 to 0.39)	0.524
Prevalence	High-middle SDI	1995-2001	-0.33<br>(-0.68 to -0.23)	0.001
Prevalence	High-middle SDI	2001-2005	-2.49<br>(-2.77 to -2.26)	<0.001
Prevalence	High-middle SDI	2005-2011	-1.58<br>(-1.71 to -1.21)	<0.001
Prevalence	High-middle SDI	2011-2019	-1.94<br>(-2.09 to -1.86)	<0.001
Prevalence	High-middle SDI	2019-2021	1.96<br>(1.52 to 2.37)	<0.001
Prevalence	Middle SDI	1990-1995	-1.49<br>(-2.05 to -1.21)	<0.001
Prevalence	Middle SDI	1995-2000	0.02<br>(-0.2 to 0.58)	0.796
Prevalence	Middle SDI	2000-2003	-2.24<br>(-2.57 to -1.72)	<0.001
Prevalence	Middle SDI	2003-2009	0.49<br>(0.22 to 0.79)	0.000
Prevalence	Middle SDI	2009-2021	-1.39<br>(-1.49 to -1.3)	<0.001
Prevalence	Low-middle SDI	1990-1992	4<br>(2 to 5.55)	<0.001
Prevalence	Low-middle SDI	1992-2006	-1.05<br>(-1.27 to -0.96)	<0.001
Prevalence	Low-middle SDI	2006-2009	0.63<br>(-0.48 to 1.1)	0.250
Prevalence	Low-middle SDI	2009-2016	-2.95<br>(-3.5 to -2.68)	<0.001
Prevalence	Low-middle SDI	2016-2021	-0.97<br>(-1.42 to -0.22)	0.034
Prevalence	Low SDI	1990-2000	0.21<br>(-0.34 to 0.9)	0.360
Prevalence	Low SDI	2000-2008	-4.37<br>(-5.38 to -3.84)	0.006
Prevalence	Low SDI	2008-2011	2.35<br>(0.69 to 3.33)	0.020

Prevalence	Low SDI	2011-2014	-2.85 (-3.66 to -1.36)	0.004
Prevalence	Low SDI	2014-2021	1.18 (0.7 to 2.12)	0.003
YLDs	High SDI	1990-1996	-0.99 (-1.11 to -0.87)	<0.001
YLDs	High SDI	1996-2000	-2.52 (-2.69 to -2.28)	<0.001
YLDs	High SDI	2000-2010	-0.58 (-0.64 to -0.52)	<0.001
YLDs	High SDI	2010-2015	-2.69 (-2.9 to -2.56)	<0.001
YLDs	High SDI	2015-2018	-1.43 (-2.24 to -0.8)	<0.001
YLDs	High SDI	2018-2021	-0.05 (-0.39 to 0.48)	0.746
YLDs	High-middle SDI	1990-1996	-0.35 (-0.5 to -0.01)	0.050
YLDs	High-middle SDI	1996-2001	-0.68 (-3.42 to -0.55)	<0.001
YLDs	High-middle SDI	2001-2005	-3.23 (-3.55 to -2.21)	<0.001
YLDs	High-middle SDI	2005-2019	-2.25 (-2.3 to -2.19)	<0.001
YLDs	High-middle SDI	2019-2021	1.67 (1.2 to 2.04)	<0.001
YLDs	Middle SDI	1990-1995	-1.93 (-2.22 to -1.74)	<0.001
YLDs	Middle SDI	1995-2000	-0.48 (-0.66 to -0.19)	0.014
YLDs	Middle SDI	2000-2003	-2.53 (-2.78 to -2.1)	<0.001
YLDs	Middle SDI	2003-2009	0.02 (-0.13 to 0.19)	0.755
YLDs	Middle SDI	2009-2017	-1.81 (-1.9 to -1.22)	0.020
YLDs	Middle SDI	2017-2021	-2.33 (-2.9 to -2.02)	<0.001

YLDs	Low-middle SDI	1990-1992	3.8 (1.87 to 5.05)	<0.001
YLDs	Low-middle SDI	1992-2006	-1.31 (-1.51 to -1.23)	<0.001
YLDs	Low-middle SDI	2006-2009	0.23 (-0.81 to 0.67)	0.670
YLDs	Low-middle SDI	2009-2016	-3.27 (-3.76 to -3.01)	<0.001
YLDs	Low-middle SDI	2016-2021	-1.37 (-1.82 to -0.62)	0.012
YLDs	Low SDI	1990-2000	0.15 (-0.73 to 1.02)	0.525
YLDs	Low SDI	2000-2008	-4.71 (-6.46 to 0.51)	0.060
YLDs	Low SDI	2008-2011	2.14 (-4.91 to 3.45)	0.155
YLDs	Low SDI	2011-2014	-3.09 (-4.28 to -0.61)	0.018
YLDs	Low SDI	2014-2021	0.87 (0.16 to 3.14)	0.036

YLDs years lived with disability, *SDI* sociodemographic index, *APC* annual percentage change, *CI* confidence interval.

Supplementary Table 6 The incidence of spinal cord injury and their AAPCs from 1990 to 2021 among children and adolescents at global level, stratified by injury causes

	Injury causes	Case (n), 1990	Rate (per 100, 000), 1990	Case (n), 2021	Rate (per 100, 000), 2021	AAPC, 1990-2021	*P*
Incidence	Animal contact	3906.87 (1660.23 to 7571.71)	0.17 (0.07 to 0.34)	2967.47 (1248.21 to 5773.52)	0.11 (0.05 to 0.22)	-1.38 (-1.4 to -1.37)	<0.001
Incidence	Conflict and terrorism	29179.88 (12227.36 to 57680.95)	1.29 (0.54 to 2.55)	21187.74 (8681.66 to 43148.92)	0.80 (0.33 to 1.64)	-1.16 (-2.66 to 0.29)	0.079
Incidence	Drowning	1196.37 (546.02 to 2187.67)	0.05 (0.02 to 0.10)	489.46 (223.97 to 894.33)	0.02 (0.01 to 0.03)	-3.35 (-3.37 to -3.33)	<0.001
Incidence	Environmental heat and cold exposure	1911.44 (971.70 to 3284.93)	0.08 (0.04 to 0.15)	1416.29 (701.29 to 2515.45)	0.05 (0.03 to 0.10)	-1.46 (-1.48 to -1.44)	<0.001
Incidence	Exposure to forces of nature	4038.16 (1608.30 to 8288.75)	0.18 (0.07 to 0.37)	892.35 (363.15 to 1860.08)	0.03 (0.01 to 0.07)	-4.81 (-8.73 to -0.73)	0.025
Incidence	Exposure to mechanical forces	9428.44 (4650.29 to 17419.45)	0.42 (0.21 to 0.77)	7124.64 (3452.77 to 13190.70)	0.27 (0.13 to 0.50)	-1.41 (-1.42 to -1.39)	<0.001
Incidence	Falls	48096.09 (28786.68 to 76597.86)	2.13 (1.27 to 3.39)	43691.36 (26528.74 to 70185.03)	1.66 (1.01 to 2.66)	-0.82 (-0.84 to -0.79)	<0.001
Incidence	Fire, heat, and hot substances	1231.45 (581.93 to 2402.67)	0.05 (0.03 to 0.11)	925.82 (430.38 to 1811.11)	0.04 (0.02 to 0.07)	-1.42 (-1.45 to -1.39)	<0.001

Incidence	Foreign body	6289.58 (3442.58 to 11247.85)	0.28 (0.15 to 0.50)	5749.41 (3051.48 to 10518.29)	0.22 (0.12 to 0.40)	-0.78 (-0.8 to -0.77)	<0.001
Incidence	Interpersonal violence	15061.23 (8390.68 to 25714.94)	0.67 (0.37 to 1.14)	11841.45 (6704.27 to 20107.25)	0.45 (0.25 to 0.76)	-1.26 (-1.28 to -1.24)	<0.001
Incidence	Other transport injuries	2389.78 (1076.68 to 4920.28)	0.11 (0.05 to 0.22)	2066.67 (917.13 to 4399.65)	0.08 (0.03 to 0.17)	-0.88 (-0.94 to -0.83)	<0.001
Incidence	Poisonings	815.46 (327.22 to 1567.00)	0.04 (0.01 to 0.07)	570.85 (226.00 to 1105.55)	0.02 (0.01 to 0.04)	-1.65 (-1.67 to -1.63)	<0.001
Incidence	Police conflict and executions	688.10 (274.56 to 1330.64)	0.03 (0.01 to 0.06)	3153.62 (1335.85 to 5960.51)	0.12 (0.05 to 0.23)	4.45 (3.07 to 5.57)	<0.001
Incidence	Road injuries	22935.80 (15793.05 to 32530.45)	1.02 (0.70 to 1.44)	11885.34 (8189.26 to 16753.56)	0.45 (0.31 to 0.64)	-2.59 (-2.61 to -2.57)	<0.001
Incidence	Self-harm	2258.80 (901.44 to 4794.86)	0.10 (0.04 to 0.21)	1842.25 (720.94 to 3955.16)	0.07 (0.03 to 0.15)	-1.15 (-1.17 to -1.13)	<0.001

AAPC average annual percentage change.

Supplementary Table 7 The prevalence of spinal cord injury and their AAPCs from 1990 to 2021 among children and adolescents at global level, stratified by injury causes

	Injury causes	Case (n), 1990	Rate (per 100, 000), 1990	Case (n), 2021	Rate (per 100, 000), 2021	AAPC, 1990-2021	*P*
Prevalence	Animal contact	299.63 (127.16 to 603.52)	0.01 (0.01 to 0.03)	227.58 (96.62 to 459.61)	0.01 (0.00 to 0.02)	-1.38 (-1.4 to -1.36)	<0.001
Prevalence	Conflict and terrorism	162349.67 (53533.65 to 376092.56)	7.19 (2.37 to 16.65)	129079.71 (52794.15 to 268742.61)	4.90 (2.00 to 10.20)	-1.19 (-1.32 to -1.02)	<0.001
Prevalence	Drowning	15783.14 (11712.29 to 21468.50)	0.70 (0.52 to 0.95)	6640.08 (4999.24 to 8592.51)	0.25 (0.19 to 0.33)	-3.25 (-3.27 to -3.23)	<0.001
Prevalence	Environmental heat and cold exposure	23925.12 (19030.85 to 31956.40)	1.06 (0.84 to 1.41)	19118.90 (15061.28 to 25299.57)	0.73 (0.57 to 0.96)	-1.21 (-1.23 to -1.19)	<0.001
Prevalence	Exposure to forces of nature	22779.72 (9204.42 to 47675.11)	1.01 (0.41 to 2.11)	47529.92 (19523.98 to 101465.95)	1.80 (0.74 to 3.85)	1.98 (1.72 to 2.3)	<0.001
Prevalence	Exposure to mechanical forces	95119.47 (73348.16 to 121747.74)	4.21 (3.25 to 5.39)	75182.05 (58749.22 to 95116.82)	2.85 (2.23 to 3.61)	-1.24 (-1.26 to -1.22)	<0.001
Prevalence	Falls	448086.29 (338354.12 to 584282.14)	19.84 (14.98 to 25.87)	394445.76 (297661.69 to 530734.40)	14.96 (11.29 to 20.14)	-0.91 (-0.94 to -0.89)	<0.001

Prevalence	Fire, heat, and hot substances	14385.30 (10683.76 to 18331.83)	0.64 (0.47 to 0.81)	10528.27 (7756.61 to 13740.58)	0.40 (0.29 to 0.52)	-1.5 (-1.54 to -1.46)	<0.001
Prevalence	Foreign body	76683.99 (62656.82 to 97911.83)	3.40 (2.77 to 4.34)	72994.12 (58839.04 to 94661.70)	2.77 (2.23 to 3.59)	-0.67 (-0.69 to -0.65)	<0.001
Prevalence	Interpersonal violence	183742.55 (160899.61 to 214413.23)	8.14 (7.12 to 9.49)	155312.35 (137007.18 to 179100.92)	5.89 (5.20 to 6.79)	-1.03 (-1.06 to -1.01)	<0.001
Prevalence	Other transport injuries	27062.91 (20461.61 to 37744.21)	1.20 (0.91 to 1.67)	22826.87 (17043.36 to 30975.01)	0.87 (0.65 to 1.18)	-1.03 (-1.08 to -0.98)	<0.001
Prevalence	Poisonings	10159.76 (7982.48 to 13470.06)	0.45 (0.35 to 0.60)	7165.17 (5700.50 to 9209.44)	0.27 (0.22 to 0.35)	-1.62 (-1.64 to -1.59)	<0.001
Prevalence	Police conflict and executions	6905.63 (2346.10 to 18139.02)	0.31 (0.10 to 0.80)	25416.10 (11095.73 to 47405.72)	0.96 (0.42 to 1.80)	3.94 (3.8 to 4.07)	<0.001
Prevalence	Road injuries	202763.78 (181228.44 to 226322.21)	8.98 (8.02 to 10.02)	95848.01 (85963.92 to 106669.48)	3.64 (3.26 to 4.05)	-2.88 (-2.91 to -2.86)	<0.001
Prevalence	Self-harm	9616.43 (6205.46 to 15478.76)	0.43 (0.27 to 0.69)	8288.52 (5445.01 to 12468.80)	0.31 (0.21 to 0.47)	-0.99 (-1.01 to -0.97)	<0.001

AAPC average annual percentage change.

Supplementary Table 8 The YLDs of spinal cord injury and their AAPCs from 1990 to 2021 among children and adolescents at global level, stratified by injury causes

	Injury causes	Case (n), 1990	Rate (per 100, 000), 1990	Case (n), 2021	Rate (per 100, 000), 2021	AAPC, 1990-2021	*P*
YLDs	Animal contact	203.04 (87.08 to 408.98)	0.01 (0.00 to 0.02)	154.19 (64.96 to 310.25)	0.01 (0.00 to 0.01)	-1.38 (-1.4 to -1.37)	<0.001
YLDs	Conflict and terrorism	64040.54 (21839.90 to 154931.49)	2.84 (0.97 to 6.86)	46001.08 (19008.28 to 94597.78)	1.75 (0.72 to 3.59)	-1.53 (-1.68 to -1.33)	<0.001
YLDs	Drowning	5976.09 (3971.70 to 8444.20)	0.26 (0.18 to 0.37)	2301.27 (1567.93 to 3285.65)	0.09 (0.06 to 0.12)	-3.51 (-3.54 to -3.49)	<0.001
YLDs	Environmental heat and cold exposure	9013.75 (5945.42 to 12770.75)	0.40 (0.26 to 0.57)	6636.53 (4390.32 to 9294.98)	0.25 (0.17 to 0.35)	-1.47 (-1.5 to -1.45)	<0.001
YLDs	Exposure to forces of nature	8619.33 (3209.50 to 18630.26)	0.38 (0.14 to 0.82)	16839.34 (6088.08 to 36822.29)	0.64 (0.23 to 1.40)	1.7 (1.42 to 2.02)	<0.001
YLDs	Exposure to mechanical forces	33817.93 (22680.25 to 45910.04)	1.50 (1.00 to 2.03)	25264.26 (16786.68 to 34386.36)	0.96 (0.64 to 1.30)	-1.42 (-1.46 to -1.39)	<0.001
YLDs	Falls	160041.06 (105794.19 to 223370.70)	7.09 (4.68 to 9.89)	128907.68 (84877.52 to 179213.01)	4.89 (3.22 to 6.80)	-1.19 (-1.22 to -1.17)	<0.001
YLDs	Fire, heat, and hot substances	5139.11 (3278.28 to 7246.78)	0.23 (0.15 to 0.32)	3513.23 (2248.07 to 4986.15)	0.13 (0.09 to 0.19)	-1.71 (-1.75 to -1.67)	<0.001

YLDs	Foreign body	28060.68 (19365.24 to 36976.67)	1.24 (0.86 to 1.64)	25064.67 (17035.51 to 33976.40)	0.95 (0.65 to 1.29)	-0.88 (-0.91 to -0.86)	<0.001
YLDs	Interpersonal violence	65231.96 (44774.67 to 85679.58)	2.89 (1.98 to 3.79)	50660.37 (34778.12 to 67098.30)	1.92 (1.32 to 2.55)	-1.31 (-1.33 to -1.29)	<0.001
YLDs	Other transport injuries	9705.03 (5902.85 to 14311.50)	0.43 (0.26 to 0.63)	7445.66 (4490.34 to 10735.92)	0.28 (0.17 to 0.41)	-1.3 (-1.35 to -1.25)	<0.001
YLDs	Poisonings	3617.15 (2342.73 to 5008.10)	0.16 (0.10 to 0.22)	2360.62 (1529.06 to 3246.38)	0.09 (0.06 to 0.12)	-1.85 (-1.88 to -1.83)	<0.001
YLDs	Police conflict and executions	2496.19 (817.14 to 6377.98)	0.11 (0.04 to 0.28)	8880.07 (3520.96 to 18548.15)	0.34 (0.13 to 0.70)	3.84 (3.59 to 4.04)	<0.001
YLDs	Road injuries	70950.60 (49333.83 to 91880.25)	3.14 (2.18 to 4.07)	31478.55 (21871.84 to 41306.46)	1.19 (0.83 to 1.57)	-3.09 (-3.11 to -3.06)	<0.001
YLDs	Self-harm	3547.03 (2099.45 to 5793.73)	0.16 (0.09 to 0.26)	2770.90 (1665.49 to 4417.20)	0.11 (0.06 to 0.17)	-1.28 (-1.31 to -1.27)	<0.001

YLDs years lived with disability, *AAPC* average annual percentage change.

Supplementary Table 9 Joinpoint regression analysis of incidence, prevalence, and YLDs of spinal cord injury from 1990 to 2021 among children and adolescents at global level, stratified by injury causes

Measure	Injury causes	Year	APC (95% CI)	*P*
Incidence	Animal contact	1990-1993	-1.33 (-1.68 to -1.09)	<0.001
Incidence	Animal contact	1993-2000	-0.79 (-0.86 to -0.63)	<0.001
Incidence	Animal contact	2000-2005	-1.54 (-1.74 to -1.42)	<0.001
Incidence	Animal contact	2005-2010	-0.89 (-0.99 to -0.75)	<0.001
Incidence	Animal contact	2010-2017	-2.39 (-2.46 to -2.34)	<0.001
Incidence	Animal contact	2017-2021	-1.08 (-1.2 to -0.96)	<0.001
Incidence	Conflict and terrorism	1990-2011	-2.5 (-5.13 to -0.83)	0.030
Incidence	Conflict and terrorism	2011-2014	34.26 (3.66 to 50.87)	0.036
Incidence	Conflict and terrorism	2014-2021	-9.67 (-30.73 to -0.67)	0.046
Incidence	Drowning	1990-1996	-2.75 (-2.91 to -2.61)	<0.001
Incidence	Drowning	1996-2005	-3.82 (-4.02 to -3.73)	<0.001
Incidence	Drowning	2005-2011	-3.57 (-3.7 to -3.3)	<0.001
Incidence	Drowning	2011-2014	-4.66 (-4.87 to -4.32)	<0.001
Incidence	Drowning	2014-2018	-3.77 (-3.94 to -3.47)	<0.001
Incidence	Drowning	2018-2021	-0.77 (-1.07 to -0.52)	0.004
Incidence	Environmental heat and cold exposure	1990-1995	-1.16 (-1.29 to -0.94)	<0.001
Incidence	Environmental heat and cold exposure	1995-2004	-1.55 (-1.64 to -1.49)	<0.001
Incidence	Environmental heat and cold exposure	2004-2011	-0.59 (-0.66 to -0.51)	<0.001

Incidence	Environmental heat and cold exposure	2011-2015	-3.63 (-3.75 to -3.51)	<0.001
Incidence	Environmental heat and cold exposure	2015-2019	-1.47 (-1.71 to -1.35)	<0.001
Incidence	Environmental heat and cold exposure	2019-2021	-0.45 (-0.86 to -0.12)	0.004
Incidence	Exposure to forces of nature	1990-2021	-4.81 (-8.73 to -0.73)	0.025
Incidence	Exposure to mechanical forces	1990-2004	-1.48 (-1.52 to -1.44)	<0.001
Incidence	Exposure to mechanical forces	2004-2010	-0.59 (-0.73 to -0.42)	0.000
Incidence	Exposure to mechanical forces	2010-2015	-2.62 (-2.77 to -2.48)	<0.001
Incidence	Exposure to mechanical forces	2015-2021	-1.04 (-1.16 to -0.91)	<0.001
Incidence	Falls	1990-1996	-0.01 (-0.16 to 0.28)	0.981
Incidence	Falls	1996-2001	-0.77 (-1.1 to -0.44)	0.002
Incidence	Falls	2001-2005	-1.66 (-2.04 to -1.34)	0.006
Incidence	Falls	2005-2010	-0.15 (-0.36 to 0.09)	0.128
Incidence	Falls	2010-2017	-2.22 (-2.34 to -2.12)	<0.001
Incidence	Falls	2017-2021	0.44 (0.22 to 0.65)	0.004
Incidence	Fire heat and hot substances	1990-1994	-2.04 (-2.42 to -1.76)	<0.001
Incidence	Fire heat and hot substances	1994-2000	-0.48 (-0.65 to -0.17)	0.015
Incidence	Fire heat and hot substances	2000-2005	-1.39 (-1.81 to -1.18)	<0.001
Incidence	Fire heat and hot substances	2005-2010	-0.71 (-0.9 to -0.38)	0.004
Incidence	Fire heat and hot substances	2010-2017	-2.78 (-2.92 to -2.66)	<0.001

Incidence	Fire heat and hot substances	2017-2021	-0.75 (-1.02 to -0.51)	0.003
Incidence	Foreign body	1990-1994	-1.57 (-1.78 to -1.43)	<0.001
Incidence	Foreign body	1994-2005	-0.92 (-0.96 to -0.89)	<0.001
Incidence	Foreign body	2005-2011	0.08 (0.02 to 0.14)	0.024
Incidence	Foreign body	2011-2015	-1.74 (-1.89 to -1.63)	0.019
Incidence	Foreign body	2015-2018	-0.6 (-1.08 to -0.42)	<0.001
Incidence	Foreign body	2018-2021	0.16 (-0.03 to 0.5)	0.081
Incidence	Interpersonal violence	1990-1996	-1.13 (-1.25 to -0.9)	<0.001
Incidence	Interpersonal violence	1996-2005	-1.48 (-1.62 to -1.41)	<0.001
Incidence	Interpersonal violence	2005-2010	-0.51 (-0.68 to -0.28)	0.010
Incidence	Interpersonal violence	2010-2018	-1.86 (-1.95 to -1.79)	<0.001
Incidence	Interpersonal violence	2018-2021	-0.51 (-0.82 to 0.07)	0.063
Incidence	Other transport injuries	1990-1995	-1.82 (-2.6 to -1.43)	<0.001
Incidence	Other transport injuries	1995-2000	-0.15 (-0.59 to 0.7)	0.510
Incidence	Other transport injuries	2000-2005	-2.78 (-3.64 to -2.34)	0.002
Incidence	Other transport injuries	2005-2010	-0.77 (-1.18 to 0.16)	0.098
Incidence	Other transport injuries	2010-2015	-2.04 (-2.8 to -1.68)	0.008
Incidence	Other transport injuries	2015-2021	1.8 (1.48 to 2.14)	<0.001
Incidence	Poisonings	1990-1995	-1.29 (-1.43 to -1.08)	<0.001

Incidence	Poisonings	1995-2005	-1.7 (-1.79 to -1.65)	<0.001
Incidence	Poisonings	2005-2011	-0.78 (-0.88 to -0.63)	<0.001
Incidence	Poisonings	2011-2014	-3.6 (-3.79 to -3.39)	<0.001
Incidence	Poisonings	2014-2018	-2.22 (-2.5 to -1.94)	<0.001
Incidence	Poisonings	2018-2021	-1.05 (-1.33 to -0.56)	<0.001
Incidence	Police conflict and executions	1990-2006	2.16 (-0.65 to 4.29)	0.108
Incidence	Police conflict and executions	2006-2017	15.27 (11.71 to 27.8)	0.001
Incidence	Police conflict and executions	2017-2021	-12.96 (-29.78 to -0.96)	0.037
Incidence	Road injuries	1990-1994	-2.56 (-2.9 to -2.38)	<0.001
Incidence	Road injuries	1994-2001	-1.99 (-2.08 to -1.83)	<0.001
Incidence	Road injuries	2001-2010	-2.72 (-2.77 to -2.65)	<0.001
Incidence	Road injuries	2010-2014	-4.01 (-4.2 to -3.88)	<0.001
Incidence	Road injuries	2014-2018	-3.42 (-3.56 to -3.2)	<0.001
Incidence	Road injuries	2018-2021	-0.6 (-0.82 to -0.42)	0.000
Incidence	Self-harm	1990-1996	0.18 (0.08 to 0.36)	0.001
Incidence	Self-harm	1996-2003	-0.16 (-0.27 to -0.08)	0.001
Incidence	Self-harm	2003-2006	-1.37 (-1.71 to -1.03)	0.001
Incidence	Self-harm	2006-2010	-2.82 (-3.05 to -2.63)	<0.001
Incidence	Self-harm	2010-2018	-2.38 (-2.44 to -2.22)	<0.001

Incidence	Self-harm	2018-2021	-0.34 (-0.6 to -0.13)	0.026
Prevalence	Animal contact	1990-1993	-1.33 (-1.68 to -1.08)	<0.001
Prevalence	Animal contact	1993-2000	-0.79 (-0.87 to -0.63)	<0.001
Prevalence	Animal contact	2000-2005	-1.54 (-1.75 to -1.42)	<0.001
Prevalence	Animal contact	2005-2010	-0.89 (-0.99 to -0.75)	<0.001
Prevalence	Animal contact	2010-2017	-2.39 (-2.46 to -2.34)	<0.001
Prevalence	Animal contact	2017-2021	-1.08 (-1.21 to -0.96)	<0.001
Prevalence	Conflict and terrorism	1990-1992	-5.04 (-6.98 to -1.56)	<0.001
Prevalence	Conflict and terrorism	1992-2000	-0.22 (-0.82 to 1.03)	0.560
Prevalence	Conflict and terrorism	2000-2007	-5.88 (-6.78 to -5.19)	<0.001
Prevalence	Conflict and terrorism	2007-2012	-1.65 (-2.83 to -0.06)	0.046
Prevalence	Conflict and terrorism	2012-2017	6.59 (5.63 to 7.93)	<0.001
Prevalence	Conflict and terrorism	2017-2021	-1.52 (-2.74 to -0.26)	0.027
Prevalence	Drowning	1990-2001	-2.53 (-2.62 to -2.47)	<0.001
Prevalence	Drowning	2001-2006	-3.92 (-4.03 to -2.47)	<0.001
Prevalence	Drowning	2006-2011	-4.37 (-4.53 to -4.05)	<0.001
Prevalence	Drowning	2011-2014	-5.07 (-5.26 to -4.75)	<0.001
Prevalence	Drowning	2014-2018	-3.96 (-4.11 to -3.68)	<0.001
Prevalence	Drowning	2018-2021	-0.03 (-0.28 to 0.2)	0.761

Prevalence	Environmental heat and cold exposure	1990-2000	-0.69 (-0.75 to -0.61)	<0.001
Prevalence	Environmental heat and cold exposure	2000-2011	-1.04 (-1.1 to -0.99)	<0.001
Prevalence	Environmental heat and cold exposure	2011-2015	-3.94 (-4.06 to -3.81)	<0.001
Prevalence	Environmental heat and cold exposure	2015-2019	-1.16 (-1.38 to -1.01)	<0.001
Prevalence	Environmental heat and cold exposure	2019-2021	0.71 (0.15 to 1.08)	0.014
Prevalence	Exposure to forces of nature	1990-1992	49.27 (42.88 to 57.55)	<0.001
Prevalence	Exposure to forces of nature	1992-2002	-0.59 (-1.6 to 0.22)	0.146
Prevalence	Exposure to forces of nature	2002-2011	6.1 (4.87 to 7.62)	<0.001
Prevalence	Exposure to forces of nature	2011-2021	-6.45 (-7.24 to -5.65)	<0.001
Prevalence	Exposure to mechanical forces	1990-1997	-1.03 (-1.11 to -0.8)	<0.001
Prevalence	Exposure to mechanical forces	1997-2005	-1.24 (-1.49 to -1.17)	<0.001
Prevalence	Exposure to mechanical forces	2005-2010	-0.66 (-0.79 to -0.46)	<0.001
Prevalence	Exposure to mechanical forces	2010-2015	-2.79 (-2.93 to -2.68)	<0.001
Prevalence	Exposure to mechanical forces	2015-2018	-1.1 (-1.82 to -0.89)	<0.001
Prevalence	Exposure to mechanical forces	2018-2021	-0.22 (-0.45 to 0.19)	0.189
Prevalence	Falls	1990-2000	-0.11 (-0.18 to -0.03)	0.011
Prevalence	Falls	2000-2005	-1.24 (-1.5 to -1)	0.008
Prevalence	Falls	2005-2010	-0.07 (-0.24 to 0.18)	0.442
Prevalence	Falls	2010-2015	-3.05 (-3.3 to -2.91)	0.005

Prevalence	Falls	2015-2018	-2.27 (-2.62 to -2.02)	<0.001
Prevalence	Falls	2018-2021	0.55 (0.21 to 0.89)	0.008
Prevalence	Fire heat and hot substances	1990-1994	-2.19 (-2.81 to -1.87)	<0.001
Prevalence	Fire heat and hot substances	1994-2000	-0.09 (-0.33 to 0.3)	0.300
Prevalence	Fire heat and hot substances	2000-2010	-1.06 (-1.2 to -0.39)	0.036
Prevalence	Fire heat and hot substances	2010-2015	-3.49 (-3.87 to -0.98)	0.000
Prevalence	Fire heat and hot substances	2015-2018	-2.46 (-3.44 to -1.68)	<0.001
Prevalence	Fire heat and hot substances	2018-2021	-0.53 (-1.07 to 0.25)	0.093
Prevalence	Foreign body	1990-1994	-1.15 (-1.47 to -0.95)	<0.001
Prevalence	Foreign body	1994-2001	-0.45 (-0.53 to -0.26)	0.008
Prevalence	Foreign body	2001-2004	-1.53 (-1.71 to -1.17)	<0.001
Prevalence	Foreign body	2004-2011	-0.36 (-0.44 to -0.22)	0.002
Prevalence	Foreign body	2011-2016	-1.62 (-1.79 to -1.49)	<0.001
Prevalence	Foreign body	2016-2021	0.48 (0.35 to 0.62)	<0.001
Prevalence	Interpersonal violence	1990-2000	-0.86 (-0.94 to -0.78)	<0.001
Prevalence	Interpersonal violence	2000-2006	-2.13 (-2.39 to -1.95)	<0.001
Prevalence	Interpersonal violence	2006-2011	-0.81 (-1.02 to -0.33)	0.000
Prevalence	Interpersonal violence	2011-2018	-1.6 (-1.76 to -1.49)	<0.001
Prevalence	Interpersonal violence	2018-2021	1.6 (1.26 to 1.91)	<0.001

Prevalence	Other transport injuries	1990-1994	-1.84 (-2.66 to -1.4)	<0.001
Prevalence	Other transport injuries	1994-2000	-0.59 (-0.88 to 0.01)	0.052
Prevalence	Other transport injuries	2000-2015	-2.11 (-2.23 to -2.01)	0.002
Prevalence	Other transport injuries	2015-2019	2.36 (-2.07 to 2.75)	0.136
Prevalence	Other transport injuries	2019-2021	0.83 (0.06 to 1.99)	0.033
Prevalence	Poisonings	1990-1998	-0.97 (-1.06 to -0.82)	<0.001
Prevalence	Poisonings	1998-2010	-1.27 (-1.33 to -1.22)	<0.001
Prevalence	Poisonings	2010-2015	-3.62 (-3.77 to -3.52)	<0.001
Prevalence	Poisonings	2015-2018	-2.33 (-2.72 to -1.99)	<0.001
Prevalence	Poisonings	2018-2021	-0.63 (-0.9 to -0.27)	0.007
Prevalence	Police conflict and executions	1990-2006	-1.45 (-1.75 to -1.19)	0.011
Prevalence	Police conflict and executions	2006-2009	6.07 (-1.4 to 7.07)	0.082
Prevalence	Police conflict and executions	2009-2012	13.81 (11.57 to 15.4)	0.005
Prevalence	Police conflict and executions	2012-2015	7.95 (6.51 to 9.68)	<0.001
Prevalence	Police conflict and executions	2015-2018	20.02 (18.31 to 21.62)	<0.001
Prevalence	Police conflict and executions	2018-2021	3.07 (1.39 to 4.54)	0.004
Prevalence	Road injuries	1990-1993	-2.63 (-3.03 to -2.3)	<0.001
Prevalence	Road injuries	1993-2001	-2.22 (-2.95 to -1.99)	<0.001
Prevalence	Road injuries	2001-2011	-3.28 (-3.34 to -3.19)	<0.001

Prevalence	Road injuries	2011-2014	-4.78 (-4.96 to -4.37)	<0.001
Prevalence	Road injuries	2014-2018	-3.94 (-4.11 to -3.66)	<0.001
Prevalence	Road injuries	2018-2021	-0.22 (-0.5 to 0.01)	0.060
Prevalence	Self-harm	1990-2000	-0.06 (-0.16 to -0.01)	0.019
Prevalence	Self-harm	2000-2003	0.55 (0.26 to 0.71)	0.002
Prevalence	Self-harm	2003-2006	-0.85 (-1.16 to -0.6)	0.001
Prevalence	Self-harm	2006-2010	-2.63 (-2.86 to -2.45)	<0.001
Prevalence	Self-harm	2010-2018	-2.18 (-2.24 to -2.04)	<0.001
Prevalence	Self-harm	2018-2021	-0.3 (-0.53 to -0.12)	0.014
YLDs	Animal contact	1990-1993	-1.33 (-1.68 to -1.09)	<0.001
YLDs	Animal contact	1993-2000	-0.79 (-0.86 to -0.63)	<0.001
YLDs	Animal contact	2000-2005	-1.54 (-1.74 to -1.42)	<0.001
YLDs	Animal contact	2005-2010	-0.89 (-0.99 to -0.75)	<0.001
YLDs	Animal contact	2010-2017	-2.4 (-2.46 to -2.34)	<0.001
YLDs	Animal contact	2017-2021	-1.08 (-1.2 to -0.96)	<0.001
YLDs	Conflict and terrorism	1990-1992	-5.3 (-7.45 to -1.15)	0.000
YLDs	Conflict and terrorism	1992-2000	-0.15 (-0.9 to 1.33)	0.789
YLDs	Conflict and terrorism	2000-2007	-6.53 (-7.86 to -5.68)	<0.001
YLDs	Conflict and terrorism	2007-2012	-2.22 (-3.89 to 0.03)	0.052

YLDs	Conflict and terrorism	2012-2017	5.82 (4.68 to 7.85)	<0.001
YLDs	Conflict and terrorism	2017-2021	-1.35 (-2.88 to 0.02)	0.052
YLDs	Drowning	1990-2001	-2.8 (-2.86 to -2.74)	<0.001
YLDs	Drowning	2001-2007	-4.24 (-4.37 to -4)	<0.001
YLDs	Drowning	2007-2016	-4.87 (-4.99 to -4.79)	<0.001
YLDs	Drowning	2016-2019	-3.23 (-3.6 to -2.99)	<0.001
YLDs	Drowning	2019-2021	0.63 (0.1 to 1.11)	0.024
YLDs	Environmental heat and cold exposure	1990-2000	-0.88 (-0.95 to -0.81)	<0.001
YLDs	Environmental heat and cold exposure	2000-2011	-1.33 (-1.39 to -1.27)	<0.001
YLDs	Environmental heat and cold exposure	2011-2015	-4.17 (-4.32 to -4.02)	<0.001
YLDs	Environmental heat and cold exposure	2015-2019	-1.56 (-1.87 to -1.38)	<0.001
YLDs	Environmental heat and cold exposure	2019-2021	0.5 (-0.09 to 0.89)	0.080
YLDs	Exposure to forces of nature	1990-1992	49.85 (43.34 to 58.09)	<0.001
YLDs	Exposure to forces of nature	1992-2002	-1.12 (-2.11 to -0.31)	0.011
YLDs	Exposure to forces of nature	2002-2011	6.08 (4.9 to 7.54)	<0.001
YLDs	Exposure to forces of nature	2011-2021	-6.81 (-7.59 to -6.01)	<0.001
YLDs	Exposure to mechanical forces	1990-2011	-1.24 (-1.38 to -0.94)	0.001
YLDs	Exposure to mechanical forces	2011-2015	-3.18 (-3.61 to -1.13)	<0.001
YLDs	Exposure to mechanical forces	2015-2019	-1.18 (-3.44 to -0.99)	<0.001

YLDs	Exposure to mechanical forces	2019-2021	-0.11 (-1.03 to 0.43)	0.556
YLDs	Falls	1990-2000	-0.37 (-0.45 to -0.29)	0.005
YLDs	Falls	2000-2005	-1.55 (-1.86 to -1.27)	0.002
YLDs	Falls	2005-2010	-0.42 (-0.64 to -0.13)	0.012
YLDs	Falls	2010-2015	-3.27 (-3.56 to -3.1)	<0.001
YLDs	Falls	2015-2018	-2.56 (-2.94 to -2.28)	<0.001
YLDs	Falls	2018-2021	0.28 (-0.08 to 0.66)	0.097
YLDs	Fire heat and hot substances	1990-1994	-2.51 (-3 to -2.21)	<0.001
YLDs	Fire heat and hot substances	1994-2000	-0.29 (-0.48 to 0.03)	0.058
YLDs	Fire heat and hot substances	2000-2010	-1.31 (-1.42 to -1.21)	0.008
YLDs	Fire heat and hot substances	2010-2015	-3.67 (-4.03 to -3.24)	<0.001
YLDs	Fire heat and hot substances	2015-2019	-2.35 (-3.13 to -1.93)	<0.001
YLDs	Fire heat and hot substances	2019-2021	-0.03 (-0.91 to 0.51)	0.825
YLDs	Foreign body	1990-1993	-1.57 (-2.1 to -1.24)	<0.001
YLDs	Foreign body	1993-2001	-0.65 (-0.72 to -0.48)	0.000
YLDs	Foreign body	2001-2004	-1.56 (-1.74 to -1.19)	<0.001
YLDs	Foreign body	2004-2011	-0.53 (-0.63 to -0.37)	0.002
YLDs	Foreign body	2011-2016	-1.86 (-2.06 to -1.7)	<0.001
YLDs	Foreign body	2016-2021	0.08 (-0.07 to 0.23)	0.252

YLDs	Interpersonal violence	1990-2000	-1.09 (-1.14 to -1.03)	<0.001
YLDs	Interpersonal violence	2000-2006	-2.37 (-2.52 to -2.25)	<0.001
YLDs	Interpersonal violence	2006-2011	-0.98 (-1.16 to -0.73)	<0.001
YLDs	Interpersonal violence	2011-2018	-1.94 (-2.04 to -1.84)	<0.001
YLDs	Interpersonal violence	2018-2021	1.09 (0.83 to 1.31)	<0.001
YLDs	Other transport injuries	1990-1994	-2.19 (-3.15 to -1.71)	<0.001
YLDs	Other transport injuries	1994-2000	-0.98 (-1.29 to -0.28)	0.006
YLDs	Other transport injuries	2000-2015	-2.34 (-2.42 to -2.27)	0.003
YLDs	Other transport injuries	2015-2021	1.66 (1.45 to 1.85)	<0.001
YLDs	Poisonings	1990-2000	-1.22 (-1.28 to -1.13)	<0.001
YLDs	Poisonings	2000-2008	-1.59 (-1.66 to -1.34)	<0.001
YLDs	Poisonings	2008-2011	-2.03 (-2.42 to -1.74)	<0.001
YLDs	Poisonings	2011-2015	-4.2 (-4.4 to -4.05)	<0.001
YLDs	Poisonings	2015-2019	-2.26 (-2.49 to -2.09)	<0.001
YLDs	Poisonings	2019-2021	-0.22 (-0.71 to 0.13)	0.190
YLDs	Police conflict and executions	1990-2006	-1.6 (-1.94 to -1.23)	<0.001
YLDs	Police conflict and executions	2006-2015	9.55 (8.48 to 10.25)	<0.001
YLDs	Police conflict and executions	2015-2018	19.5 (15.06 to 21.54)	<0.001
YLDs	Police conflict and executions	2018-2021	2.41 (-1.47 to 4.75)	0.117

YLDs	Road injuries	1990-1993	-2.93 (-3.38 to -2.59)	<0.001
YLDs	Road injuries	1993-2001	-2.41 (-2.5 to -2.18)	<0.001
YLDs	Road injuries	2001-2011	-3.5 (-3.56 to -3.39)	<0.001
YLDs	Road injuries	2011-2014	-4.84 (-5.03 to -3.59)	<0.001
YLDs	Road injuries	2014-2018	-4.15 (-4.36 to -3.86)	<0.001
YLDs	Road injuries	2018-2021	-0.46 (-0.76 to -0.2)	0.004
YLDs	Self-harm	1990-2005	-0.3 (-0.34 to -0.27)	<0.001
YLDs	Self-harm	2005-2011	-2.88 (-3.05 to -2.78)	<0.001
YLDs	Self-harm	2011-2019	-2.29 (-2.38 to -2.17)	<0.001
YLDs	Self-harm	2019-2021	0.3 (-0.18 to 0.64)	0.141

YLDs years lived with disability, *APC* annual percentage change, *CI* confidence interval.

Supplementary Table 10 The incidence of spinal cord injury and their AAPCs from 1990 to 2021 among children and adolescents at regional level

	Location	Case (n), 1990	Rate (per 100, 000), 1990	Case (n), 2021	Rate (per 100, 000), 2021	AAPC, 1990-2021	*P*
Incidence	Andean Latin America	1621.94 (1127.69 to 2450.97)	8.56 (5.95 to 12.93)	905.23 (700.61 to 1181.23)	3.82 (2.96 to 4.99)	-2.77 (-3.03 to -2.47)	<0.001
Incidence	Australasia	1037.36 (765.56 to 1403.90)	16.54 (12.20 to 22.38)	1007.56 (720.43 to 1430.12)	13.36 (9.55 to 18.96)	-0.68 (-0.7 to -0.67)	<0.001
Incidence	Caribbean	657.85 (539.47 to 802.02)	4.36 (3.57 to 5.31)	960.40 (725.90 to 1261.76)	6.29 (4.76 to 8.27)	0.07 (-0.63 to 0.78)	0.803
Incidence	Central Asia	2196.91 (1750.08 to 2796.19)	6.96 (5.54 to 8.85)	1737.95 (1357.97 to 2208.11)	5.02 (3.92 to 6.38)	-1.61 (-2.14 to -1.09)	<0.001
Incidence	Central Europe	4148.99 (3219.78 to 5316.36)	10.57 (8.20 to 13.54)	1910.93 (1421.88 to 2533.88)	8.11 (6.04 to 10.76)	-1.66 (-2.06 to -1.26)	<0.001
Incidence	Central Latin America	7683.03 (5919.60 to 9881.00)	9.30 (7.16 to 11.96)	4760.40 (3649.89 to 6209.15)	5.58 (4.28 to 7.28)	-1.7 (-2.04 to -1.38)	<0.001
Incidence	Central Sub-Saharan Africa	1538.39 (1129.84 to 2129.54)	4.96 (3.65 to 6.87)	2816.59 (2166.83 to 3842.18)	3.83 (2.95 to 5.22)	-1.09 (-2.35 to 0.18)	0.091
Incidence	East Asia	20688.37 (16272.73 to 26963.27)	4.50 (3.54 to 5.86)	10999.93 (8329.57 to 14787.91)	3.19 (2.41 to 4.29)	-1.34 (-1.71 to -0.85)	<0.001
Incidence	Eastern Europe	7176.90 (5611.80 to 9183.57)	10.67 (8.34 to 13.65)	3226.05 (2468.71 to 4244.11)	6.99 (5.35 to 9.19)	-1.52 (-1.74 to -1.31)	<0.001

Incidence	Eastern Sub-Saharan Africa	17721.66 (9463.63 to 31379.51)	15.98 (8.53 to 28.30)	9175.06 (6457.44 to 13526.55)	4.03 (2.84 to 5.94)	-1.83 (-3.05 to -0.59)	0.005
Incidence	High-income Asia Pacific	5334.84 (4130.70 to 6782.14)	10.60 (8.21 to 13.48)	1962.53 (1461.09 to 2701.80)	6.37 (4.75 to 8.78)	-1.65 (-1.75 to -1.54)	<0.001
Incidence	High-income North America	7713.85 (5924.62 to 9972.39)	9.44 (7.25 to 12.20)	4679.19 (3658.73 to 6004.82)	5.22 (4.09 to 6.70)	-1.93 (-2 to -1.86)	<0.001
Incidence	North Africa and Middle East	14365.17 (11107.91 to 18834.96)	8.13 (6.28 to 10.65)	21974.51 (14011.99 to 34973.29)	9.29 (5.92 to 14.79)	0.74 (0.02 to 1.29)	0.046
Incidence	Oceania	96.01 (75.69 to 123.47)	2.85 (2.25 to 3.67)	215.62 (167.20 to 283.03)	3.38 (2.62 to 4.43)	-0.24 (-0.92 to 0.45)	0.453
Incidence	South Asia	25800.41 (20031.09 to 33431.46)	4.76 (3.69 to 6.16)	20885.40 (16257.70 to 27488.90)	3.06 (2.38 to 4.02)	-1.46 (-2.2 to -0.75)	0.001
Incidence	Southeast Asia	11603.68 (8775.14 to 15362.44)	5.28 (3.99 to 6.99)	7889.28 (6188.06 to 10124.98)	3.44 (2.70 to 4.42)	-1.25 (-1.97 to -0.49)	0.001
Incidence	Southern Latin America	1793.14 (1392.66 to 2264.16)	9.25 (7.19 to 11.68)	1761.12 (1329.70 to 2352.82)	9.03 (6.82 to 12.06)	-0.09 (-0.13 to -0.05)	<0.001
Incidence	Southern Sub-Saharan Africa	1037.60 (835.26 to 1324.46)	3.92 (3.16 to 5.01)	868.38 (695.74 to 1100.83)	2.78 (2.23 to 3.52)	-1.11 (-1.29 to -0.93)	<0.001

Incidence	Tropical Latin America	4960.54 (3700.94 to 6782.82)	7.16 (5.34 to 9.79)	3660.75 (2760.95 to 4897.53)	5.50 (4.15 to 7.36)	-0.93 (-1.08 to -0.81)	<0.001
Incidence	Western Europe	11876.08 (9044.28 to 15728.26)	12.08 (9.20 to 15.99)	8393.07 (5914.02 to 12180.57)	9.15 (6.45 to 13.28)	-0.9 (-0.97 to -0.82)	<0.001
Incidence	Western Sub-Saharan Africa	4329.72 (3367.87 to 5651.17)	4.03 (3.13 to 5.26)	9069.74 (7127.61 to 11614.00)	3.38 (2.65 to 4.32)	-0.02 (-0.4 to 0.5)	0.941

AAPC average annual percentage change.

Supplementary Table 11 The prevalence of spinal cord injury and their AAPCs from 1990 to 2021 among children and adolescents at regional level

	Location	Case (n), 1990	Rate (per 100, 000), 1990	Case (n), 2021	Rate (per 100, 000), 2021	AAPC, 1990-2021	*P*
Prevalence	Andean Latin America	12178.14 (9921.48 to 15932.13)	64.24 (52.34 to 84.05)	8493.32 (7335.04 to 9900.84)	35.88 (30.98 to 41.82)	-1.88 (-1.93 to -1.83)	<0.001
Prevalence	Australasia	10131.41 (8535.92 to 11835.81)	161.50 (136.07 to 188.67)	10577.99 (8790.17 to 12617.36)	140.26 (116.55 to 167.30)	-0.46 (-0.48 to -0.44)	<0.001
Prevalence	Caribbean	6467.90 (5812.97 to 7224.32)	42.84 (38.50 to 47.85)	15793.13 (10008.81 to 26413.15)	103.48 (65.58 to 173.06)	2.8 (2.18 to 3.29)	<0.001
Prevalence	Central Asia	22622.01 (19650.91 to 25921.11)	71.63 (62.23 to 82.08)	16618.66 (14412.96 to 18875.66)	48.00 (41.63 to 54.52)	-1.19 (-1.35 to -1.03)	<0.001
Prevalence	Central Europe	41112.05 (36196.85 to 46468.85)	104.70 (92.18 to 118.34)	19099.85 (16420.05 to 22054.21)	81.08 (69.70 to 93.62)	-0.83 (-0.88 to -0.78)	<0.001
Prevalence	Central Latin America	83370.59 (69714.19 to 104964.94)	100.89 (84.37 to 127.03)	46970.98 (40468.81 to 55151.58)	55.08 (47.45 to 64.67)	-1.89 (-1.99 to -1.78)	<0.001
Prevalence	Central Sub-Saharan Africa	12800.17 (9950.87 to 17805.02)	41.31 (32.11 to 57.46)	20447.19 (17297.25 to 24542.75)	27.80 (23.51 to 33.36)	-1.16 (-1.28 to -1.02)	<0.001
Prevalence	East Asia	236967.12 (210779.04 to 267501.94)	51.50 (45.81 to 58.13)	118910.50 (103155.56 to 138649.35)	34.47 (29.90 to 40.19)	-1.27 (-1.33 to -1.22)	<0.001

Prevalence	Eastern Europe	63609.17 (55857.01 to 71885.36)	94.55 (83.03 to 106.85)	27351.79 (23533.67 to 31924.03)	59.25 (50.98 to 69.16)	-1.5 (-1.55 to -1.46)	<0.001
Prevalence	Eastern Sub-Saharan Africa	69185.22 (44849.34 to 115356.65)	62.39 (40.44 to 104.02)	63434.16 (54409.81 to 74751.04)	27.87 (23.91 to 32.85)	-2.53 (-2.73 to -2.24)	<0.001
Prevalence	High-income Asia Pacific	57292.62 (50874.22 to 64229.98)	113.84 (101.09 to 127.63)	21907.05 (18980.99 to 25366.24)	71.15 (61.65 to 82.39)	-1.52 (-1.54 to -1.5)	<0.001
Prevalence	High-income North America	67066.97 (59692.30 to 75682.65)	82.06 (73.04 to 92.60)	42084.88 (37666.16 to 47892.42)	46.99 (42.06 to 53.48)	-1.81 (-1.86 to -1.76)	<0.001
Prevalence	North Africa and Middle East	148431.92 (105534.10 to 257590.39)	83.97 (59.70 to 145.72)	176733.78 (123297.17 to 275108.01)	74.73 (52.14 to 116.33)	-0.28 (-0.38 to -0.21)	<0.001
Prevalence	Oceania	792.90 (691.27 to 913.84)	23.55 (20.53 to 27.15)	1625.37 (1405.85 to 1883.60)	25.45 (22.01 to 29.49)	0.15 (0.03 to 0.29)	0.020
Prevalence	South Asia	192572.90 (166817.68 to 223384.52)	35.50 (30.75 to 41.18)	205570.89 (175365.53 to 241476.97)	30.08 (25.66 to 35.33)	-0.51 (-0.56 to -0.48)	<0.001
Prevalence	Southeast Asia	96963.95 (75033.43 to 145207.63)	44.09 (34.12 to 66.03)	80366.02 (66872.49 to 99810.19)	35.05 (29.17 to 43.53)	-0.73 (-0.85 to -0.6)	<0.001

Prevalence	Southern Latin America	17145.52 (15328.42 to 19517.83)	88.47 (79.10 to 100.71)	17077.95 (14768.81 to 19855.47)	87.54 (75.70 to 101.77)	-0.03 (-0.07 to 0)	0.076
Prevalence	Southern Sub-Saharan Africa	11107.70 (9331.59 to 16153.86)	41.98 (35.27 to 61.05)	7725.34 (7003.42 to 8434.39)	24.71 (22.40 to 26.98)	-1.7 (-1.72 to -1.67)	<0.001
Prevalence	Tropical Latin America	45130.38 (38008.58 to 53280.02)	65.16 (54.88 to 76.93)	36293.05 (29361.54 to 45059.45)	54.50 (44.09 to 67.67)	-0.57 (-0.6 to -0.54)	<0.001
Prevalence	Western Europe	116303.09 (100198.60 to 133903.93)	118.26 (101.88 to 136.16)	90395.49 (75580.56 to 107001.18)	98.57 (82.41 to 116.67)	-0.58 (-0.6 to -0.57)	<0.001
Prevalence	Western Sub-Saharan Africa	29941.80 (26711.83 to 33485.23)	27.85 (24.85 to 31.15)	76094.65 (66333.38 to 86912.74)	28.33 (24.70 to 32.36)	0.04 (-0.02 to 0.09)	0.127

AAPC average annual percentage change.

Supplementary Table 12 The YLDs of spinal cord injury and their AAPCs from 1990 to 2021 among children and adolescents at regional level							
	**Location**	**Case (n), 1990**	**Rate (per 100, 000), 1990**	**Case (n), 2021**	**Rate (per 100, 000), 2021**	**AAPC, 1990-2021**	***P***
YLDs	Andean Latin America	4652.29 (3107.24 to 6815.25)	24.54 (16.39 to 35.95)	2772.72 (1852.44 to 3823.95)	11.71 (7.82 to 16.15)	-2.39 (-2.45 to -2.31)	<0.001
YLDs	Australasia	2985.72 (1961.15 to 4183.43)	47.60 (31.26 to 66.69)	3116.03 (2058.05 to 4343.56)	41.32 (27.29 to 57.59)	-0.45 (-0.48 to -0.43)	<0.001
YLDs	Caribbean	2367.63 (1633.76 to 3058.83)	15.68 (10.82 to 20.26)	5915.24 (3306.11 to 10586.20)	38.76 (21.66 to 69.36)	2.88 (2.44 to 3.39)	<0.001
YLDs	Central Asia	8056.04 (5538.03 to 10802.69)	25.51 (17.54 to 34.21)	5537.19 (3664.04 to 7486.75)	15.99 (10.58 to 21.62)	-1.39 (-1.55 to -1.25)	<0.001
YLDs	Central Europe	13631.18 (9350.66 to 18149.77)	34.71 (23.81 to 46.22)	5467.24 (3707.54 to 7410.50)	23.21 (15.74 to 31.46)	-1.3 (-1.36 to -1.25)	<0.001
YLDs	Central Latin America	30934.51 (21558.20 to 42826.03)	37.44 (26.09 to 51.83)	15499.90 (10496.04 to 20299.66)	18.17 (12.31 to 23.80)	-2.27 (-2.37 to -2.17)	<0.001
YLDs	Central Sub-Saharan Africa	5280.01 (3399.85 to 7855.05)	17.04 (10.97 to 25.35)	7908.64 (5266.65 to 11239.41)	10.75 (7.16 to 15.28)	-1.42 (-1.58 to -1.27)	<0.001
YLDs	East Asia	87074.02 (60605.65 to 112281.81)	18.92 (13.17 to 24.40)	34588.31 (23206.26 to 46968.19)	10.03 (6.73 to 13.62)	-2.02 (-2.08 to -1.96)	<0.001
YLDs	Eastern Europe	20906.90 (14470.28 to 28038.33)	31.08 (21.51 to 41.68)	8072.20 (5475.53 to 10966.34)	17.49 (11.86 to 23.76)	-1.83 (-1.88 to -1.79)	<0.001

YLDs	Eastern Sub-Saharan Africa	28650.79 (16912.16 to 50944.65)	25.84 (15.25 to 45.94)	24482.68 (16729.35 to 32453.70)	10.76 (7.35 to 14.26)	-2.75 (-2.99 to -2.45)	<0.001
YLDs	High-income Asia Pacific	17769.52 (12165.42 to 23556.95)	35.31 (24.17 to 46.81)	6361.62 (4364.43 to 8548.47)	20.66 (14.18 to 27.76)	-1.73 (-1.75 to -1.71)	<0.001
YLDs	High-income North America	19667.96 (13701.53 to 26424.71)	24.06 (16.76 to 32.33)	12125.74 (8343.67 to 16252.67)	13.54 (9.32 to 18.15)	-1.87 (-1.92 to -1.83)	<0.001
YLDs	North Africa and Middle East	55514.52 (33769.75 to 98103.70)	31.40 (19.10 to 55.50)	59411.14 (36125.59 to 95504.69)	25.12 (15.28 to 40.38)	-0.63 (-0.71 to -0.57)	<0.001
YLDs	Oceania	310.99 (213.41 to 420.95)	9.24 (6.34 to 12.50)	615.08 (411.91 to 866.99)	9.63 (6.45 to 13.58)	0.05 (-0.21 to 0.31)	0.645
YLDs	South Asia	76219.18 (51641.24 to 98553.35)	14.05 (9.52 to 18.17)	73592.89 (49981.20 to 97211.87)	10.77 (7.31 to 14.22)	-0.85 (-0.9 to -0.8)	<0.001
YLDs	Southeast Asia	37305.09 (24756.43 to 61098.83)	16.96 (11.26 to 27.78)	28195.23 (19120.49 to 39187.53)	12.30 (8.34 to 17.09)	-1.08 (-1.19 to -0.96)	<0.001
YLDs	Southern Latin America	6076.41 (4207.82 to 7996.00)	31.36 (21.71 to 41.26)	5407.35 (3711.78 to 7450.36)	27.72 (19.03 to 38.19)	-0.41 (-0.46 to -0.37)	<0.001
YLDs	Southern Sub-Saharan Africa	4146.39 (2756.17 to 6164.76)	15.67 (10.42 to 23.30)	2799.00 (1938.95 to 3653.29)	8.95 (6.20 to 11.69)	-1.8 (-1.83 to -1.77)	<0.001

YLDs	Tropical Latin America	16526.61 (11318.12 to 21950.78)	23.86 (16.34 to 31.69)	11862.67 (7733.61 to 16374.02)	17.82 (11.61 to 24.59)	-0.93 (-0.96 to -0.89)	<0.001
YLDs	Western Europe	34884.11 (23860.19 to 46475.17)	35.47 (24.26 to 47.26)	26466.72 (17694.48 to 36372.90)	28.86 (19.29 to 39.66)	-0.66 (-0.68 to -0.64)	<0.001
YLDs	Western Sub-Saharan Africa	11919.30 (8291.98 to 15307.07)	11.09 (7.71 to 14.24)	28672.83 (19490.24 to 38103.54)	10.68 (7.26 to 14.19)	-0.14 (-0.2 to -0.08)	<0.001

YLDs years lived with disability, *AAPC* average annual percentage change.

Supplementary Table 13 Joinpoint regression analysis of incidence, prevalence, and YLDs of spinal cord injury from 1990 to 2021 among children and adolescents at regional level

Measure	Location	Year	APC (95% CI)	*P*
Incidence	Andean Latin America	1990-1995	-12.29 (-14.98 to -9.92)	<0.001
Incidence	Andean Latin America	1995-2021	-0.83 (-1.01 to -0.65)	<0.001
Incidence	Australasia	1990-1993	-0.62 (-0.99 to -0.43)	<0.001
Incidence	Australasia	1993-2004	0.13 (0.09 to 0.17)	<0.001
Incidence	Australasia	2004-2011	-0.61 (-0.67 to -0.54)	0.003
Incidence	Australasia	2011-2014	-2.6 (-2.73 to -2.44)	<0.001
Incidence	Australasia	2014-2018	-1.87 (-1.99 to -1.65)	<0.001
Incidence	Australasia	2018-2021	-0.35 (-0.55 to -0.17)	0.020
Incidence	Caribbean	1990-2021	0.07 (-0.63 to 0.78)	0.803
Incidence	Central Asia	1990-2021	-1.61 (-2.14 to -1.09)	<0.001
Incidence	Central Europe	1990-2021	-1.66 (-2.06 to -1.26)	<0.001
Incidence	Central Latin America	1990-1995	-5.8 (-10.44 to -3.3)	0.000
Incidence	Central Latin America	1995-1999	8.73 (4.38 to 14.89)	0.000
Incidence	Central Latin America	1999-2003	-6.51 (-11.13 to -2.78)	0.000
Incidence	Central Latin America	2003-2010	1.87 (0.03 to 8.02)	0.047
Incidence	Central Latin America	2010-2021	-3.81 (-5.15 to -2.85)	0.004
Incidence	Central Sub-Saharan Africa	1990-2021	-1.09 (-2.35 to 0.18)	0.091
Incidence	East Asia	1990-2011	-2.49 (-3.15 to -2.05)	<0.001

Incidence	East Asia	2011-2021	1.13 (-0.3 to 4.21)	0.108
Incidence	Eastern Europe	1990-2000	2.03 (1.13 to 3.13)	<0.001
Incidence	Eastern Europe	2000-2021	-3.17 (-3.51 to -2.86)	<0.001
Incidence	Eastern Sub-Saharan Africa	1990-2021	-1.83 (-3.05 to -0.59)	0.005
Incidence	High-income Asia Pacific	1990-1995	-0.17 (-0.94 to 1.19)	0.662
Incidence	High-income Asia Pacific	1995-2008	-1.89 (-2.68 to -1.71)	0.001
Incidence	High-income Asia Pacific	2008-2011	1.1 (-0.71 to 2)	0.205
Incidence	High-income Asia Pacific	2011-2014	-5.93 (-6.89 to -4.11)	0.000
Incidence	High-income Asia Pacific	2014-2021	-1.53 (-2.02 to -0.59)	0.016
Incidence	High-income North America	1990-1996	-0.93 (-1.26 to -0.54)	0.007
Incidence	High-income North America	1996-2000	-6.82 (-7.33 to -5.9)	0.006
Incidence	High-income North America	2000-2006	-1.23 (-1.78 to -0.61)	0.026
Incidence	High-income North America	2006-2015	-2.31 (-2.86 to -1.57)	0.002
Incidence	High-income North America	2015-2019	1.22 (-2.23 to 2.33)	0.093
Incidence	High-income North America	2019-2021	-1.42 (-2.71 to 0.39)	0.094
Incidence	North Africa and Middle East	1990-2002	-0.64 (-2.62 to 0.31)	0.162
Incidence	North Africa and Middle East	2002-2006	10.24 (3.29 to 18.48)	0.008
Incidence	North Africa and Middle East	2006-2010	-8.95 (-17.31 to -3.02)	0.015
Incidence	North Africa and Middle East	2010-2014	28.18 (17.02 to 44.1)	0.008

Incidence	North Africa and Middle East	2014-2021	-9.54 (-15.05 to -5.05)	0.006
Incidence	Oceania	1990-2021	-0.24 (-0.92 to 0.45)	0.453
Incidence	South Asia	1990-2010	-0.5 (-1.54 to 3.69)	0.521
Incidence	South Asia	2010-2021	-3.19 (-11.03 to -1.24)	0.031
Incidence	Southeast Asia	1990-2021	-1.25 (-1.97 to -0.49)	0.001
Incidence	Southern Latin America	1990-1998	0.48 (0.29 to 0.62)	0.004
Incidence	Southern Latin America	1998-2005	1.64 (1.35 to 2.01)	0.004
Incidence	Southern Latin America	2005-2010	0.19 (-0.16 to 1.24)	0.188
Incidence	Southern Latin America	2010-2015	-1.88 (-2.09 to -0.17)	0.031
Incidence	Southern Latin America	2015-2019	-2.75 (-3.27 to -2.37)	<0.001
Incidence	Southern Latin America	2019-2021	0.86 (-0.08 to 1.55)	0.065
Incidence	Southern Sub-Saharan Africa	1990-2008	-0.71 (-1.01 to -0.37)	0.014
Incidence	Southern Sub-Saharan Africa	2008-2017	-2.49 (-4.9 to -0.73)	0.029
Incidence	Southern Sub-Saharan Africa	2017-2021	0.18 (-1.77 to 3.44)	0.828
Incidence	Tropical Latin America	1990-1996	1.79 (1.06 to 2.86)	0.002
Incidence	Tropical Latin America	1996-2007	-0.35 (-1.54 to -0.09)	0.016
Incidence	Tropical Latin America	2007-2010	2.8 (0.89 to 3.76)	0.002
Incidence	Tropical Latin America	2010-2013	-2.87 (-3.84 to -1.12)	0.002
Incidence	Tropical Latin America	2013-2019	1.49 (0.88 to 2.96)	0.000

Incidence	Tropical Latin America	2019-2021	-19.85 (-21.91 to -18.41)	<0.001
Incidence	Western Europe	1990-1997	-0.77 (-1.45 to -0.52)	0.002
Incidence	Western Europe	1997-2009	-0.23 (-0.36 to 0.24)	0.118
Incidence	Western Europe	2009-2018	-2.13 (-2.59 to -1.92)	<0.001
Incidence	Western Europe	2018-2021	-0.15 (-1.13 to 1.29)	0.575
Incidence	Western Sub-Saharan Africa	1990-2000	2.36 (0.8 to 5.83)	0.009
Incidence	Western Sub-Saharan Africa	2000-2005	-6.26 (-12.33 to -1.94)	0.005
Incidence	Western Sub-Saharan Africa	2005-2021	0.53 (-0.28 to 2.51)	0.168
Prevalence	Andean Latin America	1990-1992	0.45 (-1.01 to 1.44)	0.328
Prevalence	Andean Latin America	1992-1997	-3.84 (-4.33 to -3.53)	<0.001
Prevalence	Andean Latin America	1997-2007	-2.1 (-2.22 to -2.01)	<0.001
Prevalence	Andean Latin America	2007-2010	-0.85 (-1.35 to -0.62)	<0.001
Prevalence	Andean Latin America	2010-2017	-2.1 (-2.38 to -1.98)	<0.001
Prevalence	Andean Latin America	2017-2021	-0.41 (-0.76 to 0.13)	0.071
Prevalence	Australasia	1990-1994	-0.3 (-0.62 to -0.12)	0.006
Prevalence	Australasia	1994-2003	0.94 (0.87 to 1.05)	<0.001
Prevalence	Australasia	2003-2006	0.07 (-0.5 to 0.71)	0.616
Prevalence	Australasia	2006-2010	-0.78 (-2.02 to -0.62)	<0.001
Prevalence	Australasia	2010-2018	-2.26 (-2.35 to -2.17)	<0.001

Prevalence	Australasia	2018-2021	-0.07 (-0.34 to 0.16)	0.500
Prevalence	Caribbean	1990-2009	0.6 (0.37 to 0.82)	<0.001
Prevalence	Caribbean	2009-2012	54.39 (38.28 to 62.11)	<0.001
Prevalence	Caribbean	2012-2021	-6.04 (-9.71 to -3.42)	<0.001
Prevalence	Central Asia	1990-1994	4.54 (2.71 to 7.06)	<0.001
Prevalence	Central Asia	1994-2005	-1.47 (-1.95 to -0.92)	0.000
Prevalence	Central Asia	2005-2018	-3.68 (-4.08 to -3.41)	<0.001
Prevalence	Central Asia	2018-2021	3.45 (1.18 to 7.54)	0.006
Prevalence	Central Europe	1990-1994	2.69 (2.18 to 3.17)	<0.001
Prevalence	Central Europe	1994-2001	-0.06 (-0.31 to 0.27)	0.571
Prevalence	Central Europe	2001-2009	-1.22 (-1.5 to -1)	<0.001
Prevalence	Central Europe	2009-2018	-2.96 (-3.16 to -2.8)	<0.001
Prevalence	Central Europe	2018-2021	0.32 (-0.4 to 1.48)	0.238
Prevalence	Central Latin America	1990-1995	-5.49 (-6.16 to -4.76)	<0.001
Prevalence	Central Latin America	1995-2001	1.65 (1.2 to 2.63)	0.002
Prevalence	Central Latin America	2001-2005	-0.71 (-1.81 to 0.21)	0.144
Prevalence	Central Latin America	2005-2011	2.02 (1.61 to 2.68)	0.001
Prevalence	Central Latin America	2011-2019	-6.41 (-6.73 to -6.14)	<0.001
Prevalence	Central Latin America	2019-2021	1.62 (-0.89 to 3.11)	0.091

Prevalence	Central Sub-Saharan Africa	1990-1995	0.29 (-0.96 to 1.4)	0.578
Prevalence	Central Sub-Saharan Africa	1995-1998	18.42 (14.36 to 20.41)	0.006
Prevalence	Central Sub-Saharan Africa	1998-2012	-5.46 (-6.26 to -5.1)	0.020
Prevalence	Central Sub-Saharan Africa	2012-2016	-3.36 (-4.95 to -0.92)	0.008
Prevalence	Central Sub-Saharan Africa	2016-2021	0.79 (0.02 to 2.27)	0.048
Prevalence	East Asia	1990-1994	-1.41 (-2.44 to -0.92)	<0.001
Prevalence	East Asia	1994-2001	-0.21 (-0.42 to 0.21)	0.195
Prevalence	East Asia	2001-2004	-7.22 (-7.67 to -6.82)	<0.001
Prevalence	East Asia	2004-2007	-4.19 (-4.55 to -3.5)	0.001
Prevalence	East Asia	2007-2018	-0.58 (-0.71 to -0.46)	0.001
Prevalence	East Asia	2018-2021	3.18 (2.41 to 4.89)	<0.001
Prevalence	Eastern Europe	1990-2001	1.32 (1.23 to 1.42)	0.000
Prevalence	Eastern Europe	2001-2004	-1.89 (-2.38 to -0.6)	0.030
Prevalence	Eastern Europe	2004-2008	-3.8 (-4.36 to -3.38)	<0.001
Prevalence	Eastern Europe	2008-2014	-5.57 (-6.01 to -5.33)	<0.001
Prevalence	Eastern Europe	2014-2018	-2.42 (-3.21 to -1.68)	0.001
Prevalence	Eastern Europe	2018-2021	1.43 (0.69 to 2.52)	0.002
Prevalence	Eastern Sub-Saharan Africa	1990-2000	1.62 (0.28 to 3.19)	0.021
Prevalence	Eastern Sub-Saharan Africa	2000-2014	-6.53 (-7.25 to -5.95)	<0.001

Prevalence	Eastern Sub-Saharan Africa	2014-2021	-0.15 (-0.86 to 0.79)	0.577
Prevalence	High-income Asia Pacific	1990-1997	-1.07 (-1.16 to -0.97)	<0.001
Prevalence	High-income Asia Pacific	1997-2004	-1.56 (-1.73 to -1.47)	<0.001
Prevalence	High-income Asia Pacific	2004-2011	-0.66 (-0.76 to -0.53)	<0.001
Prevalence	High-income Asia Pacific	2011-2015	-3.73 (-4 to -3.57)	<0.001
Prevalence	High-income Asia Pacific	2015-2018	-2.66 (-3.01 to -2.32)	<0.001
Prevalence	High-income Asia Pacific	2018-2021	-0.32 (-0.63 to 0.04)	0.063
Prevalence	High-income North America	1990-1996	-0.46 (-0.69 to -0.23)	0.003
Prevalence	High-income North America	1996-1999	-8.98 (-9.43 to -8.58)	0.000
Prevalence	High-income North America	1999-2002	-3.33 (-3.66 to -2.7)	<0.001
Prevalence	High-income North America	2002-2015	-1.39 (-1.47 to -1.28)	<0.001
Prevalence	High-income North America	2015-2019	1.34 (1.01 to 2.12)	0.008
Prevalence	High-income North America	2019-2021	-1.27 (-2.25 to -0.21)	0.015
Prevalence	North Africa and Middle East	1990-2002	-2.82 (-2.97 to -2.67)	0.002
Prevalence	North Africa and Middle East	2002-2005	0.44 (-1.82 to 0.78)	0.154
Prevalence	North Africa and Middle East	2005-2008	2.42 (1.83 to 2.87)	0.008
Prevalence	North Africa and Middle East	2008-2011	-2.19 (-2.64 to -1.65)	0.008
Prevalence	North Africa and Middle East	2011-2018	4.28 (3.98 to 4.57)	<0.001
Prevalence	North Africa and Middle East	2018-2021	-1.86 (-3.07 to -0.32)	0.015

Prevalence	Oceania	1990-1997	2.18 (1.54 to 2.7)	<0.001
Prevalence	Oceania	1997-2000	15.41 (10.64 to 17.04)	<0.001
Prevalence	Oceania	2000-2015	-1.91 (-2.32 to -1.37)	0.001
Prevalence	Oceania	2015-2018	-8.12 (-9.26 to -5.5)	<0.001
Prevalence	Oceania	2018-2021	0.28 (-1.15 to 2.8)	0.655
Prevalence	South Asia	1990-1992	7.2 (6.52 to 7.9)	<0.001
Prevalence	South Asia	1992-1997	-0.58 (-0.97 to -0.33)	0.000
Prevalence	South Asia	1997-2010	-0.23 (-0.29 to 0.05)	0.069
Prevalence	South Asia	2010-2014	-3.25 (-3.67 to -2.78)	0.002
Prevalence	South Asia	2014-2019	-2.06 (-2.5 to -1.72)	<0.001
Prevalence	South Asia	2019-2021	-0.18 (-1.04 to 0.39)	0.419
Prevalence	Southeast Asia	1990-2003	-2.09 (-2.34 to -1.82)	0.010
Prevalence	Southeast Asia	2003-2010	4.58 (-1.01 to 5.22)	0.053
Prevalence	Southeast Asia	2010-2015	-3.6 (-5.6 to 4.62)	0.098
Prevalence	Southeast Asia	2015-2021	-1.37 (-2.27 to 0.57)	0.101
Prevalence	Southern Latin America	1990-1995	0.57 (0.07 to 0.81)	0.034
Prevalence	Southern Latin America	1995-2000	1.29 (0.97 to 2.06)	<0.001
Prevalence	Southern Latin America	2000-2005	2.67 (2.36 to 3.01)	0.019
Prevalence	Southern Latin America	2005-2011	-0.13 (-0.29 to 0.01)	0.065

Prevalence	Southern Latin America	2011-2019	-3.15 (-3.26 to -3.04)	<0.001
Prevalence	Southern Latin America	2019-2021	1.55 (0.93 to 2.07)	0.000
Prevalence	Southern Sub-Saharan Africa	1990-2001	-1.85 (-1.9 to -1.54)	<0.001
Prevalence	Southern Sub-Saharan Africa	2001-2005	-2.14 (-2.35 to -1.96)	<0.001
Prevalence	Southern Sub-Saharan Africa	2005-2010	-1.45 (-1.54 to -1.31)	<0.001
Prevalence	Southern Sub-Saharan Africa	2010-2015	-3.08 (-3.16 to -3)	<0.001
Prevalence	Southern Sub-Saharan Africa	2015-2018	-0.98 (-1.13 to -0.83)	<0.001
Prevalence	Southern Sub-Saharan Africa	2018-2021	0.63 (0.45 to 0.8)	<0.001
Prevalence	Tropical Latin America	1990-1994	2.72 (2.43 to 3.08)	<0.001
Prevalence	Tropical Latin America	1994-1998	0.8 (0.45 to 1.28)	0.003
Prevalence	Tropical Latin America	1998-2004	-0.36 (-0.63 to -0.12)	0.006
Prevalence	Tropical Latin America	2004-2011	-1.26 (-1.5 to -1.12)	<0.001
Prevalence	Tropical Latin America	2011-2016	-3.53 (-3.78 to -3.31)	<0.001
Prevalence	Tropical Latin America	2016-2021	-0.52 (-0.78 to -0.27)	0.018
Prevalence	Western Europe	1990-1994	-0.84 (-1.12 to -0.68)	<0.001
Prevalence	Western Europe	1994-2000	-0.15 (-0.31 to 0.03)	0.070
Prevalence	Western Europe	2000-2006	0.47 (0.36 to 0.72)	<0.001
Prevalence	Western Europe	2006-2010	-0.21 (-0.45 to -0.05)	0.009
Prevalence	Western Europe	2010-2018	-2.07 (-2.13 to -2.01)	<0.001

Prevalence	Western Europe	2018-2021	0.3 (0.09 to 0.47)	0.011
Prevalence	Western Sub-Saharan Africa	1990-1997	0.85 (0.16 to 1.1)	0.032
Prevalence	Western Sub-Saharan Africa	1997-2000	2.92 (1.66 to 3.36)	0.012
Prevalence	Western Sub-Saharan Africa	2000-2014	-1.15 (-1.29 to -1.03)	0.003
Prevalence	Western Sub-Saharan Africa	2014-2021	0.42 (0.1 to 0.89)	0.017
YLDs	Andean Latin America	1990-1992	-0.11 (-1.75 to 1.31)	0.778
YLDs	Andean Latin America	1992-1997	-4.57 (-5.54 to -4.23)	<0.001
YLDs	Andean Latin America	1997-2007	-2.51 (-2.97 to -2.31)	<0.001
YLDs	Andean Latin America	2007-2011	-1.43 (-2 to -0.79)	<0.001
YLDs	Andean Latin America	2011-2016	-2.95 (-3.96 to -2.56)	<0.001
YLDs	Andean Latin America	2016-2021	-1.04 (-1.5 to 0.11)	0.061
YLDs	Australasia	1990-1994	-0.14 (-0.53 to 0.11)	0.225
YLDs	Australasia	1994-2004	0.87 (0.77 to 0.97)	<0.001
YLDs	Australasia	2004-2010	-0.6 (-0.72 to 0.86)	0.095
YLDs	Australasia	2010-2016	-2.3 (-2.56 to -0.62)	<0.001
YLDs	Australasia	2016-2019	-1.67 (-2.27 to -1.47)	<0.001
YLDs	Australasia	2019-2021	0.29 (-0.33 to 0.73)	0.262
YLDs	Caribbean	1990-2009	0.47 (0.03 to 0.86)	0.037
YLDs	Caribbean	2009-2012	58.82 (39.54 to 66.62)	<0.001

YLDs	Caribbean	2012-2021	-6.43 (-9.22 to -3.95)	<0.001
YLDs	Central Asia	1990-1994	4.99 (3.33 to 6.99)	<0.001
YLDs	Central Asia	1994-2005	-1.66 (-2.06 to -1.24)	<0.001
YLDs	Central Asia	2005-2018	-4.07 (-4.42 to -3.81)	<0.001
YLDs	Central Asia	2018-2021	3.2 (0.9 to 7.13)	0.005
YLDs	Central Europe	1990-1994	2.07 (1.49 to 2.66)	<0.001
YLDs	Central Europe	1994-2001	-0.75 (-1 to -0.38)	0.004
YLDs	Central Europe	2001-2009	-1.78 (-2.08 to -1.55)	<0.001
YLDs	Central Europe	2009-2018	-3.26 (-3.51 to -3.09)	<0.001
YLDs	Central Europe	2018-2021	0.22 (-0.57 to 1.6)	0.408
YLDs	Central Latin America	1990-1995	-6.1 (-6.69 to -5.52)	<0.001
YLDs	Central Latin America	1995-2001	1.02 (0.61 to 1.99)	0.007
YLDs	Central Latin America	2001-2005	-0.91 (-1.98 to -0.03)	0.044
YLDs	Central Latin America	2005-2011	1.63 (1.21 to 2.32)	0.009
YLDs	Central Latin America	2011-2019	-6.61 (-6.92 to -6.34)	<0.001
YLDs	Central Latin America	2019-2021	1.48 (-0.47 to 2.95)	0.086
YLDs	Central Sub-Saharan Africa	1990-1995	0.24 (-1.16 to 1.48)	0.695
YLDs	Central Sub-Saharan Africa	1995-1998	18.07 (15.26 to 19.95)	<0.001
YLDs	Central Sub-Saharan Africa	1998-2014	-5.6 (-5.91 to -5.32)	<0.001

YLDs	Central Sub-Saharan Africa	2014-2021	-0.46 (-1.27 to 0.59)	0.243
YLDs	East Asia	1990-1993	-2.25 (-3.21 to -1.53)	<0.001
YLDs	East Asia	1993-2001	-1.04 (-1.2 to -0.68)	0.000
YLDs	East Asia	2001-2005	-7.87 (-8.33 to -7.56)	<0.001
YLDs	East Asia	2005-2008	-3.77 (-4.43 to -2.97)	<0.001
YLDs	East Asia	2008-2018	-1.19 (-1.34 to -1.01)	0.000
YLDs	East Asia	2018-2021	2.79 (2.06 to 3.44)	<0.001
YLDs	Eastern Europe	1990-2001	1.38 (1.28 to 1.5)	0.000
YLDs	Eastern Europe	2001-2004	-2.55 (-3.07 to -0.86)	0.036
YLDs	Eastern Europe	2004-2008	-4.56 (-5.15 to -4.02)	<0.001
YLDs	Eastern Europe	2008-2014	-6.1 (-6.56 to -5.87)	<0.001
YLDs	Eastern Europe	2014-2018	-2.91 (-3.56 to -2.22)	<0.001
YLDs	Eastern Europe	2018-2021	1.21 (0.5 to 2.01)	0.005
YLDs	Eastern Sub-Saharan Africa	1990-2000	1.43 (0.21 to 2.93)	0.028
YLDs	Eastern Sub-Saharan Africa	2000-2014	-6.78 (-7.67 to -6.13)	<0.001
YLDs	Eastern Sub-Saharan Africa	2014-2021	-0.35 (-1.63 to 1.39)	0.467
YLDs	High-income Asia Pacific	1990-1998	-1.6 (-1.68 to -1.48)	<0.001
YLDs	High-income Asia Pacific	1998-2004	-2 (-2.33 to -1.88)	<0.001
YLDs	High-income Asia Pacific	2004-2011	-0.67 (-0.78 to -0.54)	<0.001

YLDs	High-income Asia Pacific	2011-2015	-3.78 (-4.07 to -3.6)	<0.001
YLDs	High-income Asia Pacific	2015-2018	-2.58 (-3 to -2.2)	<0.001
YLDs	High-income Asia Pacific	2018-2021	-0.33 (-0.67 to 0.04)	0.066
YLDs	High-income North America	1990-1996	-0.56 (-0.78 to -0.33)	0.001
YLDs	High-income North America	1996-1999	-9.2 (-9.63 to -8.81)	<0.001
YLDs	High-income North America	1999-2002	-3.33 (-3.66 to -2.74)	<0.001
YLDs	High-income North America	2002-2015	-1.41 (-1.48 to -1.3)	<0.001
YLDs	High-income North America	2015-2019	1.32 (1.02 to 2.05)	0.002
YLDs	High-income North America	2019-2021	-1.4 (-2.31 to -0.35)	0.002
YLDs	North Africa and Middle East	1990-2002	-3.28 (-3.47 to -3.1)	<0.001
YLDs	North Africa and Middle East	2002-2005	0.08 (-3.38 to 0.53)	0.901
YLDs	North Africa and Middle East	2005-2008	1.89 (0.94 to 2.47)	0.029
YLDs	North Africa and Middle East	2008-2011	-2.46 (-2.94 to -1.72)	0.029
YLDs	North Africa and Middle East	2011-2018	4.05 (3.77 to 4.36)	<0.001
YLDs	North Africa and Middle East	2018-2021	-1.94 (-2.79 to -0.9)	0.002
YLDs	Oceania	1990-1997	2.11 (0.79 to 3.18)	0.021
YLDs	Oceania	1997-2000	15.3 (2.8 to 17.33)	0.019
YLDs	Oceania	2000-2015	-1.98 (-3.78 to 14.05)	0.074
YLDs	Oceania	2015-2018	-7.62 (-9.18 to 0.2)	0.054

YLDs	Oceania	2018-2021	-0.7 (-4.67 to 4.34)	0.611
YLDs	South Asia	1990-1992	6.67 (6 to 7.39)	<0.001
YLDs	South Asia	1992-2010	-0.61 (-0.67 to -0.56)	0.001
YLDs	South Asia	2010-2014	-3.63 (-4.01 to -0.66)	0.002
YLDs	South Asia	2014-2019	-2.48 (-3.31 to -2.07)	<0.001
YLDs	South Asia	2019-2021	-0.52 (-1.59 to 0.17)	0.102
YLDs	Southeast Asia	1990-2003	-2.33 (-2.67 to -2.01)	<0.001
YLDs	Southeast Asia	2003-2009	4.64 (3.51 to 6.18)	<0.001
YLDs	Southeast Asia	2009-2021	-2.48 (-2.85 to -2.12)	<0.001
YLDs	Southern Latin America	1990-1995	-0.04 (-0.65 to 0.25)	0.722
YLDs	Southern Latin America	1995-2000	0.83 (0.46 to 1.71)	<0.001
YLDs	Southern Latin America	2000-2005	2.37 (1.97 to 2.77)	0.025
YLDs	Southern Latin America	2005-2011	-0.48 (-0.68 to -0.32)	0.002
YLDs	Southern Latin America	2011-2019	-3.44 (-3.56 to -3.32)	<0.001
YLDs	Southern Latin America	2019-2021	1.24 (0.53 to 1.81)	0.004
YLDs	Southern Sub-Saharan Africa	1990-2001	-1.76 (-1.81 to -1.63)	<0.001
YLDs	Southern Sub-Saharan Africa	2001-2004	-2.29 (-2.49 to -1.92)	<0.001
YLDs	Southern Sub-Saharan Africa	2004-2010	-1.58 (-1.73 to -1.27)	<0.001
YLDs	Southern Sub-Saharan Africa	2010-2015	-3.4 (-3.68 to -3.19)	<0.001

YLDs	Southern Sub-Saharan Africa	2015-2018	-1.51 (-2.32 to -1.07)	<0.001
YLDs	Southern Sub-Saharan Africa	2018-2021	0.52 (0.17 to 1.05)	0.013
YLDs	Tropical Latin America	1990-1994	2.23 (1.9 to 2.67)	<0.001
YLDs	Tropical Latin America	1994-1997	0.54 (-0.47 to 1.3)	0.144
YLDs	Tropical Latin America	1997-2005	-0.67 (-1.08 to -0.54)	0.000
YLDs	Tropical Latin America	2005-2011	-1.66 (-2.09 to -1.46)	<0.001
YLDs	Tropical Latin America	2011-2016	-3.77 (-4.07 to -3.46)	<0.001
YLDs	Tropical Latin America	2016-2021	-0.94 (-1.21 to -0.62)	0.004
YLDs	Western Europe	1990-1994	-1.29 (-1.59 to -1.1)	<0.001
YLDs	Western Europe	1994-2000	-0.27 (-0.44 to -0.1)	0.012
YLDs	Western Europe	2000-2006	0.48 (0.36 to 0.76)	<0.001
YLDs	Western Europe	2006-2010	-0.2 (-0.46 to -0.04)	0.013
YLDs	Western Europe	2010-2018	-2.07 (-2.13 to -2.01)	<0.001
YLDs	Western Europe	2018-2021	0.26 (0.02 to 0.44)	0.033
YLDs	Western Sub-Saharan Africa	1990-1997	0.78 (-0.12 to 1.09)	0.065
YLDs	Western Sub-Saharan Africa	1997-2000	2.79 (1.53 to 3.28)	0.020
YLDs	Western Sub-Saharan Africa	2000-2013	-1.37 (-1.59 to -1.24)	0.004
YLDs	Western Sub-Saharan Africa	2013-2021	0.01 (-0.3 to 0.5)	0.925

YLDs years lived with disability, *APC* annual percentage change, *CI* confidence interval.

Supplementary Table 14 The incidence of spinal cord injury and their AAPCs from 1990 to 2021 among children and adolescents at national level

	Location	Case (n), 1990	Rate (per 100, 000), 1990	Case (n), 2021	Rate (per 100, 000), 2021	AAPC, 1990-2021	*P*
Incidence	Afghanistan	596.16 (384.73 to 942.63)	10.70 (6.90 to 16.91)	8772.00 (4166.91 to 16810.20)	49.35 (23.44 to 94.56)	4.29 (2.24 to 5.8)	0.002
Incidence	Albania	140.69 (107.83 to 185.03)	9.73 (7.46 to 12.80)	55.12 (41.39 to 73.11)	8.93 (6.71 to 11.85)	-0.47 (-1.06 to 0.14)	0.118
Incidence	Algeria	784.59 (628.20 to 978.17)	5.80 (4.65 to 7.24)	612.71 (484.99 to 788.66)	3.72 (2.95 to 4.79)	-1.45 (-1.86 to -0.95)	<0.001
Incidence	American Samoa	0.59 (0.46 to 0.75)	2.47 (1.91 to 3.14)	0.46 (0.35 to 0.60)	2.39 (1.81 to 3.13)	-0.07 (-0.74 to 0.63)	0.734
Incidence	Andorra	1.58 (1.15 to 2.22)	11.76 (8.54 to 16.50)	1.44 (1.01 to 2.14)	10.03 (7.04 to 14.91)	-0.52 (-0.55 to -0.51)	<0.001
Incidence	Angola	741.33 (420.64 to 1266.17)	12.86 (7.30 to 21.96)	477.75 (386.62 to 601.19)	2.56 (2.07 to 3.22)	-2.7 (-4.54 to -0.66)	0.006
Incidence	Antigua and Barbuda	0.89 (0.70 to 1.13)	3.74 (2.93 to 4.73)	0.91 (0.70 to 1.16)	3.90 (3.00 to 4.98)	0.32 (-0.49 to 0.97)	0.287
Incidence	Argentina	1218.99 (944.57 to 1542.08)	9.36 (7.25 to 11.84)	1192.18 (909.54 to 1572.71)	8.69 (6.63 to 11.47)	-0.26 (-0.32 to -0.2)	<0.001
Incidence	Armenia	103.53 (81.87 to 131.02)	7.80 (6.17 to 9.87)	35.46 (27.80 to 45.02)	4.65 (3.64 to 5.90)	-1.46 (-2.26 to -0.64)	0.002
Incidence	Australia	828.89 (605.66 to 1128.72)	16.03 (11.71 to 21.82)	804.16 (568.33 to 1150.25)	12.89 (9.11 to 18.44)	-0.69 (-0.71 to -0.68)	<0.001

Incidence	Austria	274.22 (208.14 to 366.58)	14.64 (11.11 to 19.57)	183.11 (131.20 to 260.01)	10.43 (7.48 to 14.82)	-1.08 (-1.11 to -1.05)	<0.001
Incidence	Azerbaijan	194.85 (156.39 to 243.38)	6.22 (5.00 to 7.78)	149.41 (116.29 to 191.85)	4.90 (3.82 to 6.30)	-1.2 (-1.96 to -0.46)	0.002
Incidence	Bahamas	3.80 (3.07 to 4.71)	3.53 (2.85 to 4.37)	4.15 (3.28 to 5.27)	3.60 (2.85 to 4.57)	0.17 (-0.33 to 0.66)	0.461
Incidence	Bahrain	7.20 (5.72 to 9.02)	3.60 (2.86 to 4.51)	13.97 (10.79 to 18.42)	3.46 (2.67 to 4.56)	-0.03 (-0.42 to 0.29)	0.829
Incidence	Bangladesh	1819.47 (1451.68 to 2339.56)	3.01 (2.41 to 3.88)	1627.12 (1250.74 to 2156.99)	2.67 (2.05 to 3.54)	-0.18 (-0.96 to 0.63)	0.590
Incidence	Barbados	2.90 (2.30 to 3.62)	3.42 (2.71 to 4.26)	2.29 (1.75 to 2.95)	3.44 (2.64 to 4.44)	0.35 (-0.01 to 0.84)	0.056
Incidence	Belarus	285.74 (223.84 to 367.72)	9.11 (7.13 to 11.72)	143.04 (108.34 to 191.05)	7.06 (5.35 to 9.43)	-0.81 (-0.83 to -0.8)	<0.001
Incidence	Belgium	334.90 (250.00 to 450.17)	13.55 (10.11 to 18.21)	255.33 (174.92 to 375.60)	10.04 (6.88 to 14.76)	-0.95 (-1.04 to -0.89)	<0.001
Incidence	Belize	6.21 (4.78 to 8.57)	6.06 (4.66 to 8.35)	7.66 (5.99 to 9.57)	4.53 (3.55 to 5.66)	-0.36 (-1.05 to 0.33)	0.296
Incidence	Benin	87.64 (70.20 to 108.67)	3.05 (2.45 to 3.79)	214.19 (172.64 to 271.23)	2.85 (2.30 to 3.61)	-0.2 (-0.26 to -0.15)	<0.001
Incidence	Bermuda	0.57 (0.46 to 0.72)	3.60 (2.87 to 4.53)	0.43 (0.32 to 0.57)	3.73 (2.83 to 4.92)	0 (-0.19 to 0.2)	0.996

Incidence	Bhutan	8.89 (6.89 to 11.37)	2.61 (2.02 to 3.34)	8.38 (6.45 to 10.75)	3.29 (2.53 to 4.22)	0.14 (-0.48 to 0.76)	0.624
Incidence	Bolivia (Plurinational State of)	150.34 (123.92 to 184.35)	4.49 (3.70 to 5.50)	161.05 (125.63 to 207.36)	3.54 (2.76 to 4.56)	-0.89 (-1.03 to -0.76)	<0.001
Incidence	Bosnia and Herzegovina	181.30 (136.20 to 237.74)	12.24 (9.19 to 16.04)	54.63 (39.90 to 72.94)	8.21 (6.00 to 10.97)	-0.26 (-1.96 to 1.56)	0.605
Incidence	Botswana	20.66 (16.82 to 25.60)	2.79 (2.27 to 3.46)	28.03 (22.50 to 34.96)	3.06 (2.45 to 3.81)	0.41 (0.28 to 0.53)	<0.001
Incidence	Brazil	4849.75 (3623.44 to 6629.05)	7.22 (5.39 to 9.87)	3527.16 (2656.86 to 4727.97)	5.52 (4.16 to 7.40)	-0.95 (-1.09 to -0.83)	<0.001
Incidence	Brunei Darussalam	11.20 (8.87 to 14.23)	9.74 (7.72 to 12.37)	9.04 (7.04 to 11.70)	7.00 (5.45 to 9.06)	-1.07 (-1.1 to -1.03)	<0.001
Incidence	Bulgaria	291.43 (221.39 to 380.54)	12.33 (9.37 to 16.10)	121.29 (90.81 to 159.78)	9.37 (7.02 to 12.35)	-0.91 (-0.95 to -0.87)	<0.001
Incidence	Burkina Faso	175.27 (139.68 to 219.74)	3.10 (2.47 to 3.88)	666.23 (464.17 to 978.85)	5.20 (3.62 to 7.64)	2.07 (1.7 to 2.72)	<0.001
Incidence	Burundi	89.47 (72.09 to 110.06)	2.84 (2.28 to 3.49)	217.48 (172.02 to 276.76)	2.99 (2.36 to 3.80)	-0.01 (-1.81 to 1.68)	0.993
Incidence	Cabo Verde	5.98 (4.68 to 7.59)	3.07 (2.41 to 3.90)	5.25 (4.08 to 6.82)	2.73 (2.12 to 3.54)	-0.4 (-0.5 to -0.34)	<0.001
Incidence	Cambodia	331.93 (233.21 to 493.98)	5.83 (4.10 to 8.68)	192.50 (149.09 to 251.78)	2.90 (2.25 to 3.80)	-1.66 (-2.21 to -1.02)	<0.001

Incidence	Cameroon	152.41 (121.50 to 189.59)	2.57 (2.05 to 3.19)	647.27 (495.76 to 876.40)	3.83 (2.93 to 5.18)	1.35 (0.71 to 2.15)	<0.001
Incidence	Canada	579.84 (460.06 to 735.27)	7.57 (6.00 to 9.59)	387.83 (302.75 to 503.10)	4.71 (3.68 to 6.11)	-1.51 (-1.54 to -1.48)	<0.001
Incidence	Central African Republic	45.55 (37.31 to 56.62)	3.04 (2.49 to 3.78)	350.07 (214.72 to 565.76)	12.10 (7.42 to 19.55)	2.96 (1.75 to 4.25)	<0.001
Incidence	Chad	245.60 (156.22 to 393.05)	6.97 (4.43 to 11.15)	526.21 (375.16 to 759.34)	4.81 (3.43 to 6.93)	-0.89 (-2.14 to 0.44)	0.176
Incidence	Chile	468.21 (360.79 to 607.77)	8.88 (6.84 to 11.52)	487.64 (355.51 to 680.28)	9.96 (7.26 to 13.90)	0.25 (0.14 to 0.33)	<0.001
Incidence	China	20199.44 (15876.95 to 26345.47)	4.54 (3.57 to 5.92)	10773.59 (8142.92 to 14518.75)	3.22 (2.44 to 4.34)	-1.33 (-1.71 to -0.83)	0.000
Incidence	Colombia	1577.01 (1183.68 to 2126.66)	10.48 (7.86 to 14.13)	863.17 (658.06 to 1118.80)	5.93 (4.52 to 7.68)	-1.78 (-2.3 to -1.2)	<0.001
Incidence	Comoros	7.16 (5.79 to 8.84)	2.71 (2.19 to 3.35)	7.45 (5.89 to 9.52)	2.37 (1.88 to 3.03)	-0.04 (-0.6 to 0.53)	0.884
Incidence	Congo	35.12 (28.31 to 43.98)	2.65 (2.13 to 3.31)	58.44 (46.67 to 74.00)	2.35 (1.88 to 2.98)	-1.09 (-2.3 to 0.08)	0.066
Incidence	Cook Islands	0.37 (0.27 to 0.54)	4.28 (3.16 to 6.28)	0.12 (0.09 to 0.17)	2.39 (1.77 to 3.23)	-0.59 (-1.28 to 0.13)	0.091
Incidence	Costa Rica	84.94 (62.74 to 115.63)	5.99 (4.42 to 8.15)	72.91 (53.74 to 99.45)	5.33 (3.93 to 7.26)	-0.46 (-0.53 to -0.36)	<0.001

Incidence	Coted'Ivoire	197.37 (157.92 to 245.41)	2.85 (2.28 to 3.55)	385.85 (306.51 to 489.43)	2.68 (2.13 to 3.40)	-0.11 (-0.57 to 0.35)	0.621
Incidence	Croatia	138.86 (106.93 to 181.67)	10.47 (8.06 to 13.70)	70.27 (51.36 to 95.02)	8.72 (6.37 to 11.79)	-0.79 (-2.03 to 0.14)	0.081
Incidence	Cuba	182.53 (148.38 to 229.73)	5.00 (4.06 to 6.29)	107.71 (82.21 to 141.13)	4.49 (3.42 to 5.88)	-0.39 (-0.45 to -0.33)	<0.001
Incidence	Cyprus	30.12 (22.28 to 40.12)	11.62 (8.60 to 15.49)	28.09 (20.18 to 40.68)	9.85 (7.08 to 14.27)	-0.53 (-0.56 to -0.49)	<0.001
Incidence	Czechia	363.13 (279.85 to 470.31)	11.93 (9.19 to 15.45)	206.19 (153.74 to 271.63)	9.29 (6.92 to 12.23)	-0.81 (-0.85 to -0.79)	<0.001
Incidence	Democratic People's Republic of Korea	240.09 (189.76 to 307.95)	3.08 (2.44 to 3.95)	143.11 (115.58 to 177.46)	2.17 (1.75 to 2.69)	-1 (-1.3 to -0.68)	<0.001
Incidence	Democratic Republic of the Congo	694.90 (558.69 to 867.60)	3.21 (2.58 to 4.01)	1893.36 (1433.39 to 2604.34)	3.95 (2.99 to 5.44)	0.22 (-0.94 to 1.41)	0.701
Incidence	Denmark	137.97 (102.37 to 189.34)	11.04 (8.19 to 15.14)	119.88 (81.38 to 175.33)	9.26 (6.29 to 13.55)	-0.57 (-0.59 to -0.55)	<0.001
Incidence	Djibouti	17.63 (10.63 to 28.76)	7.86 (4.74 to 12.82)	14.23 (11.46 to 18.19)	2.69 (2.17 to 3.44)	-4.38 (-6.85 to -1.8)	0.004
Incidence	Dominica	1.09 (0.88 to 1.35)	3.32 (2.68 to 4.12)	0.68 (0.54 to 0.86)	3.52 (2.80 to 4.44)	0.56 (0 to 1.15)	0.048
Incidence	Dominican Republic	123.24 (100.57 to 149.55)	3.52 (2.87 to 4.27)	138.79 (111.59 to 173.27)	3.57 (2.87 to 4.45)	0.05 (-0.25 to 0.34)	0.683

Incidence	Ecuador	222.28 (177.61 to 281.84)	4.48 (3.58 to 5.69)	281.50 (218.09 to 370.12)	4.25 (3.30 to 5.59)	-0.37 (-0.66 to -0.09)	0.018
Incidence	Egypt	1517.04 (1239.19 to 1876.30)	5.43 (4.44 to 6.72)	1614.21 (1306.97 to 2015.07)	3.47 (2.81 to 4.33)	-1.07 (-1.34 to -0.79)	<0.001
Incidence	El Salvador	579.61 (341.65 to 969.60)	21.04 (12.40 to 35.19)	151.48 (116.98 to 197.02)	6.36 (4.91 to 8.27)	-4.32 (-5.58 to -3.03)	<0.001
Incidence	Equatorial Guinea	7.16 (5.75 to 8.86)	2.98 (2.40 to 3.69)	16.75 (13.06 to 21.50)	2.17 (1.70 to 2.79)	-1.11 (-1.3 to -0.93)	<0.001
Incidence	Eritrea	2176.67 (910.75 to 4255.35)	110.67 (46.31 to 216.36)	79.94 (63.58 to 100.82)	2.49 (1.98 to 3.13)	-13.36 (-17.76 to -10.41)	<0.001
Incidence	Estonia	48.95 (38.70 to 62.31)	10.66 (8.43 to 13.57)	18.03 (13.65 to 24.23)	6.43 (4.87 to 8.64)	-1.61 (-1.67 to -1.57)	<0.001
Incidence	Eswatini	15.73 (12.58 to 20.34)	3.30 (2.64 to 4.26)	30.43 (20.50 to 47.15)	5.69 (3.83 to 8.81)	0.9 (0.04 to 1.43)	0.039
Incidence	Ethiopia	11544.09 (5471.09 to 21732.19)	39.18 (18.57 to 73.75)	3963.64 (2148.03 to 7419.76)	6.93 (3.76 to 12.97)	-5.52 (-8.75 to -2.76)	0.001
Incidence	Fiji	7.85 (6.14 to 9.99)	2.19 (1.71 to 2.79)	7.93 (6.21 to 10.12)	2.26 (1.77 to 2.89)	-0.11 (-1.7 to 1.18)	0.746
Incidence	Finland	185.20 (133.69 to 264.35)	14.60 (10.54 to 20.83)	142.34 (94.95 to 214.27)	12.39 (8.27 to 18.66)	-0.53 (-0.56 to -0.51)	<0.001
Incidence	France	2063.89 (1555.15 to 2788.26)	12.79 (9.63 to 17.27)	1540.92 (1093.42 to 2242.96)	9.78 (6.94 to 14.24)	-0.84 (-0.92 to -0.79)	<0.001

Incidence	Gabon	14.33 (11.49 to 17.88)	2.82 (2.26 to 3.52)	20.23 (15.94 to 25.92)	2.45 (1.93 to 3.13)	-0.51 (-0.66 to -0.37)	<0.001
Incidence	Gambia	14.00 (10.91 to 17.64)	2.49 (1.94 to 3.14)	28.01 (21.57 to 36.03)	2.21 (1.70 to 2.84)	-0.27 (-0.72 to 0.15)	0.177
Incidence	Georgia	143.49 (109.65 to 188.19)	7.99 (6.10 to 10.47)	66.27 (50.22 to 86.99)	7.13 (5.40 to 9.36)	-0.8 (-1.45 to -0.15)	0.014
Incidence	Germany	2028.97 (1538.50 to 2745.05)	11.71 (8.88 to 15.84)	1490.78 (1035.51 to 2139.13)	9.35 (6.49 to 13.41)	-0.71 (-0.74 to -0.68)	<0.001
Incidence	Ghana	202.46 (159.75 to 252.74)	2.45 (1.93 to 3.06)	388.19 (301.84 to 499.58)	2.38 (1.85 to 3.06)	-0.06 (-0.25 to 0.11)	0.430
Incidence	Greece	360.33 (270.90 to 489.09)	12.82 (9.64 to 17.40)	176.99 (128.27 to 251.28)	9.29 (6.73 to 13.19)	-1.04 (-1.07 to -1.01)	<0.001
Incidence	Greenland	1.39 (1.11 to 1.75)	7.77 (6.20 to 9.80)	0.76 (0.60 to 0.98)	4.95 (3.88 to 6.37)	-1.5 (-1.58 to -1.38)	<0.001
Incidence	Grenada	1.46 (1.16 to 1.81)	3.45 (2.76 to 4.30)	1.11 (0.86 to 1.41)	3.68 (2.85 to 4.70)	0.17 (-0.19 to 0.55)	0.323
Incidence	Guam	1.43 (1.12 to 1.83)	2.63 (2.07 to 3.38)	1.14 (0.88 to 1.49)	2.36 (1.81 to 3.06)	-0.47 (-0.66 to -0.29)	<0.001
Incidence	Guatemala	591.23 (396.60 to 913.13)	12.04 (8.08 to 18.59)	382.02 (295.77 to 486.13)	5.78 (4.47 to 7.35)	-2.07 (-2.53 to -1.65)	<0.001
Incidence	Guinea	105.85 (85.27 to 131.44)	3.22 (2.59 to 4.00)	213.97 (172.83 to 271.80)	2.87 (2.32 to 3.64)	-0.09 (-0.68 to 0.51)	0.758

Incidence	Guinea-Bissau	20.49 (16.80 to 25.45)	3.48 (2.86 to 4.33)	29.54 (23.73 to 37.19)	2.64 (2.12 to 3.32)	-0.99 (-1.59 to -0.39)	0.003
Incidence	Guyana	15.06 (12.40 to 18.36)	3.95 (3.25 to 4.81)	11.90 (9.63 to 14.78)	4.24 (3.43 to 5.27)	0.24 (-0.15 to 0.67)	0.225
Incidence	Haiti	153.61 (128.15 to 182.96)	4.59 (3.83 to 5.47)	541.93 (370.96 to 784.22)	9.66 (6.62 to 13.98)	-0.13 (-1.12 to 0.87)	0.782
Incidence	Honduras	165.14 (129.49 to 208.09)	6.09 (4.77 to 7.67)	232.52 (178.47 to 296.66)	5.34 (4.10 to 6.82)	-0.56 (-1.11 to 0.01)	0.053
Incidence	Hungary	291.14 (225.34 to 368.54)	10.03 (7.76 to 12.70)	161.34 (119.29 to 215.43)	8.60 (6.36 to 11.49)	-0.51 (-0.53 to -0.49)	<0.001
Incidence	Iceland	9.95 (7.35 to 13.64)	11.77 (8.69 to 16.13)	8.31 (5.72 to 12.36)	9.33 (6.42 to 13.87)	-0.81 (-1.14 to -0.48)	<0.001
Incidence	India	21917.88 (16856.98 to 28915.70)	5.34 (4.11 to 7.05)	15737.83 (12096.80 to 20930.71)	3.14 (2.42 to 4.18)	-1.66 (-1.97 to -1.34)	<0.001
Incidence	Indonesia	3216.49 (2613.27 to 3986.65)	3.68 (2.99 to 4.56)	2324.23 (1850.14 to 2938.88)	2.58 (2.05 to 3.26)	-1.27 (-1.73 to -0.79)	<0.001
Incidence	Iran (Islamic Republic of)	4851.10 (3194.85 to 7553.78)	15.40 (10.14 to 23.97)	1153.71 (917.72 to 1460.07)	4.46 (3.55 to 5.65)	-3.17 (-3.99 to -2.2)	<0.001
Incidence	Iraq	802.18 (619.97 to 1042.84)	7.79 (6.02 to 10.13)	915.72 (705.73 to 1174.29)	5.19 (4.00 to 6.66)	-1.49 (-2.25 to -0.83)	<0.001
Incidence	Ireland	151.17 (110.88 to 206.73)	11.38 (8.34 to 15.56)	125.20 (85.49 to 184.92)	9.47 (6.47 to 13.99)	-0.59 (-0.61 to -0.56)	<0.001

Incidence	Israel	232.59 (177.74 to 309.13)	11.59 (8.86 to 15.41)	354.92 (254.37 to 491.86)	10.52 (7.54 to 14.58)	-0.7 (-1.96 to 0.42)	0.146
Incidence	Italy	1903.65 (1394.68 to 2606.26)	13.98 (10.24 to 19.14)	1158.54 (797.31 to 1715.11)	11.06 (7.61 to 16.37)	-0.76 (-0.81 to -0.73)	<0.001
Incidence	Jamaica	49.46 (39.00 to 61.52)	4.53 (3.57 to 5.63)	43.86 (31.79 to 62.50)	5.38 (3.90 to 7.66)	0.53 (-0.35 to 1.04)	0.124
Incidence	Japan	3130.44 (2412.40 to 4025.74)	9.38 (7.23 to 12.07)	1289.74 (963.11 to 1748.49)	6.07 (4.54 to 8.23)	-1.5 (-1.62 to -1.37)	<0.001
Incidence	Jordan	88.34 (70.46 to 110.85)	4.21 (3.36 to 5.29)	172.38 (131.32 to 226.53)	3.50 (2.67 to 4.60)	-0.58 (-0.79 to -0.36)	0.001
Incidence	Kazakhstan	545.37 (432.76 to 689.65)	8.21 (6.51 to 10.38)	393.17 (302.93 to 504.91)	5.85 (4.51 to 7.51)	-1.12 (-1.17 to -1.06)	<0.001
Incidence	Kenya	359.29 (279.68 to 457.91)	2.61 (2.03 to 3.32)	580.62 (459.94 to 738.34)	2.36 (1.87 to 3.00)	-0.04 (-0.53 to 0.47)	0.917
Incidence	Kiribati	0.79 (0.62 to 1.00)	2.16 (1.68 to 2.72)	0.90 (0.71 to 1.15)	1.69 (1.32 to 2.15)	-0.93 (-1.5 to -0.35)	0.004
Incidence	Kuwait	209.86 (102.67 to 399.91)	30.59 (14.97 to 58.30)	41.54 (31.90 to 55.55)	3.79 (2.91 to 5.07)	-8.12 (-10.77 to -6.34)	<0.001
Incidence	Kyrgyzstan	157.56 (125.05 to 201.23)	7.46 (5.92 to 9.53)	138.98 (109.90 to 176.90)	4.92 (3.89 to 6.27)	-1.83 (-2.14 to -1.5)	<0.001
Incidence	Lao People's Democratic Republic	179.89 (100.20 to 335.18)	7.93 (4.42 to 14.78)	67.60 (53.90 to 85.02)	2.26 (1.80 to 2.84)	-1.13 (-1.51 to -0.75)	<0.001

Incidence	Latvia	80.14 (63.96 to 102.58)	10.65 (8.50 to 13.63)	24.10 (18.27 to 31.87)	6.23 (4.72 to 8.23)	-1.74 (-1.78 to -1.71)	<0.001
Incidence	Lebanon	266.02 (157.57 to 449.68)	19.86 (11.77 to 33.58)	67.50 (51.07 to 89.21)	4.05 (3.07 to 5.35)	-4.05 (-5.21 to -2.98)	<0.001
Incidence	Lesotho	25.99 (21.10 to 32.85)	3.10 (2.52 to 3.92)	30.96 (24.66 to 39.58)	3.69 (2.94 to 4.72)	0.56 (0.2 to 0.92)	0.008
Incidence	Liberia	784.23 (356.82 to 1495.31)	57.40 (26.12 to 109.45)	61.78 (47.98 to 80.32)	2.21 (1.72 to 2.88)	-8.56 (-13.37 to - 4.25)	0.001
Incidence	Libya	104.04 (83.55 to 129.47)	4.51 (3.62 to 5.61)	134.38 (104.76 to 178.14)	6.44 (5.02 to 8.53)	1.67 (0.27 to 3.81)	0.022
Incidence	Lithuania	103.39 (81.76 to 132.36)	9.33 (7.38 to 11.95)	33.42 (25.44 to 43.90)	6.24 (4.75 to 8.19)	-1.29 (-1.32 to -1.26)	<0.001
Incidence	Luxembourg	12.13 (9.23 to 16.21)	13.74 (10.46 to 18.37)	13.25 (9.22 to 19.19)	9.82 (6.83 to 14.23)	-1.09 (-1.12 to -1.06)	<0.001
Incidence	Madagascar	184.54 (147.22 to 229.63)	2.74 (2.19 to 3.41)	361.04 (282.40 to 464.60)	2.42 (1.89 to 3.11)	-0.44 (-0.71 to -0.17)	0.002
Incidence	Malawi	153.59 (121.45 to 190.05)	2.76 (2.18 to 3.41)	240.98 (186.58 to 306.95)	2.28 (1.76 to 2.90)	-0.73 (-0.91 to -0.55)	<0.001
Incidence	Malaysia	203.41 (166.67 to 249.17)	2.44 (2.00 to 2.99)	247.15 (196.66 to 312.73)	2.41 (1.92 to 3.05)	-0.09 (-0.32 to 0.09)	0.284
Incidence	Maldives	3.37 (2.68 to 4.23)	2.63 (2.09 to 3.30)	3.11 (2.37 to 4.19)	2.39 (1.82 to 3.21)	-0.15 (-0.77 to 0.5)	0.541

Incidence	Mali	291.91 (207.21 to 423.99)	5.90 (4.19 to 8.57)	629.12 (474.88 to 856.75)	4.42 (3.34 to 6.02)	-0.33 (-1.95 to 1.22)	0.538
Incidence	Malta	12.90 (9.14 to 18.39)	11.19 (7.93 to 15.95)	8.62 (6.00 to 12.72)	10.26 (7.14 to 15.15)	-0.31 (-0.36 to -0.27)	<0.001
Incidence	Marshall Islands	0.60 (0.47 to 0.76)	2.22 (1.75 to 2.83)	0.51 (0.39 to 0.64)	2.19 (1.70 to 2.78)	-0.04 (-0.05 to -0.03)	<0.001
Incidence	Mauritania	36.58 (29.74 to 45.58)	3.23 (2.62 to 4.02)	55.95 (44.20 to 71.06)	2.40 (1.90 to 3.05)	-0.71 (-1 to -0.45)	0.000
Incidence	Mauritius	10.81 (8.54 to 13.56)	2.51 (1.98 to 3.15)	6.83 (5.35 to 8.78)	2.30 (1.80 to 2.96)	-0.18 (-0.32 to -0.04)	0.015
Incidence	Mexico	3751.00 (2846.62 to 4973.87)	8.65 (6.56 to 11.47)	2263.51 (1710.21 to 3050.17)	5.25 (3.96 to 7.07)	-1.5 (-1.65 to -1.37)	<0.001
Incidence	Micronesia (Federated States of)	1.41 (1.11 to 1.77)	2.46 (1.94 to 3.08)	1.01 (0.77 to 1.31)	2.43 (1.87 to 3.16)	-0.01 (-0.49 to 0.5)	0.938
Incidence	Monaco	0.48 (0.34 to 0.68)	9.99 (7.04 to 14.04)	0.61 (0.43 to 0.90)	9.08 (6.30 to 13.28)	-0.3 (-0.32 to -0.29)	<0.001
Incidence	Mongolia	75.78 (62.14 to 92.40)	6.68 (5.47 to 8.14)	78.34 (61.61 to 99.34)	5.97 (4.69 to 7.57)	-0.09 (-0.39 to 0.19)	0.488
Incidence	Montenegro	21.37 (16.10 to 28.12)	9.96 (7.51 to 13.11)	12.63 (9.43 to 17.01)	8.40 (6.27 to 11.31)	-0.54 (-0.57 to -0.5)	<0.001
Incidence	Morocco	601.64 (486.36 to 744.94)	4.82 (3.90 to 5.97)	467.29 (369.64 to 591.45)	3.62 (2.86 to 4.58)	-1 (-1.11 to -0.89)	<0.001

Incidence	Mozambique	632.07 (347.34 to 1106.11)	8.35 (4.59 to 14.61)	657.44 (493.80 to 919.81)	3.70 (2.78 to 5.18)	-2.54 (-3.63 to -1.38)	<0.001
Incidence	Myanmar	924.62 (717.88 to 1190.75)	4.84 (3.76 to 6.23)	2271.42 (1423.04 to 3556.74)	10.94 (6.85 to 17.13)	-0.32 (-1.48 to 0.87)	0.565
Incidence	Namibia	20.93 (16.72 to 26.54)	2.75 (2.19 to 3.48)	29.05 (23.23 to 36.62)	2.71 (2.17 to 3.42)	0.05 (-0.47 to 0.51)	0.757
Incidence	Nauru	0.13 (0.11 to 0.17)	2.58 (2.04 to 3.26)	0.14 (0.11 to 0.18)	2.75 (2.13 to 3.52)	0.22 (0.2 to 0.23)	<0.001
Incidence	Nepal	381.30 (294.77 to 499.77)	3.68 (2.85 to 4.83)	455.66 (345.42 to 607.47)	3.66 (2.77 to 4.88)	0.68 (-0.56 to 2.22)	0.166
Incidence	Netherlands	348.17 (261.71 to 463.91)	9.08 (6.83 to 12.10)	259.35 (184.46 to 370.85)	7.03 (5.00 to 10.05)	-0.79 (-0.96 to -0.63)	<0.001
Incidence	New Zealand	208.47 (158.82 to 280.71)	18.93 (14.42 to 25.49)	203.40 (149.79 to 278.68)	15.60 (11.49 to 21.38)	-0.64 (-0.7 to -0.58)	<0.001
Incidence	Nicaragua	150.38 (115.33 to 192.47)	6.68 (5.13 to 8.55)	129.45 (97.22 to 173.65)	4.98 (3.74 to 6.68)	-0.83 (-1.39 to -0.27)	0.005
Incidence	Niger	206.96 (163.10 to 265.26)	4.27 (3.36 to 5.47)	676.89 (504.84 to 932.12)	4.34 (3.24 to 5.98)	0.53 (0.02 to 1.08)	0.045
Incidence	Nigeria	1520.93 (1198.21 to 1952.83)	3.13 (2.47 to 4.02)	4134.58 (3242.71 to 5192.71)	3.24 (2.54 to 4.06)	0.34 (-0.24 to 0.91)	0.190
Incidence	Niue	0.02 (0.02 to 0.03)	2.41 (1.88 to 3.07)	0.01 (0.01 to 0.02)	2.59 (1.98 to 3.35)	0.08 (-0.48 to 0.65)	0.699

Incidence	North Macedonia	58.49 (45.18 to 76.51)	8.39 (6.48 to 10.98)	35.03 (25.87 to 46.70)	7.84 (5.79 to 10.46)	-0.39 (-0.72 to -0.11)	0.040
Incidence	Northern Mariana Islands	0.44 (0.34 to 0.57)	2.72 (2.11 to 3.51)	0.43 (0.33 to 0.57)	2.84 (2.17 to 3.77)	0.51 (0.2 to 0.82)	0.002
Incidence	Norway	122.70 (92.58 to 164.76)	11.03 (8.32 to 14.81)	100.47 (70.45 to 144.64)	8.09 (5.67 to 11.65)	-1.04 (-1.2 to -0.82)	<0.001
Incidence	Oman	62.56 (50.27 to 78.05)	6.24 (5.02 to 7.79)	71.68 (56.71 to 91.13)	4.80 (3.80 to 6.11)	-0.83 (-0.89 to -0.74)	<0.001
Incidence	Pakistan	1672.87 (1308.90 to 2143.22)	2.74 (2.15 to 3.52)	3056.41 (2405.81 to 3917.29)	2.80 (2.20 to 3.59)	-0.78 (-1.62 to -0.03)	0.043
Incidence	Palau	0.21 (0.16 to 0.27)	3.41 (2.66 to 4.40)	0.14 (0.11 to 0.19)	3.21 (2.45 to 4.21)	-0.2 (-0.21 to -0.19)	<0.001
Incidence	Palestine	155.73 (89.85 to 267.24)	13.05 (7.53 to 22.39)	161.99 (113.72 to 235.56)	6.73 (4.72 to 9.78)	-2.92 (-4.36 to -1.44)	0.001
Incidence	Panama	67.86 (52.07 to 88.79)	6.24 (4.79 to 8.17)	78.19 (58.39 to 105.19)	5.19 (3.87 to 6.98)	-0.71 (-0.84 to -0.58)	<0.001
Incidence	Papua New Guinea	64.74 (50.19 to 86.81)	3.04 (2.36 to 4.08)	178.22 (137.00 to 235.80)	3.64 (2.80 to 4.81)	-0.32 (-1.09 to 0.45)	0.382
Incidence	Paraguay	110.79 (78.43 to 157.20)	5.35 (3.79 to 7.60)	133.59 (99.68 to 179.15)	5.00 (3.73 to 6.71)	-0.2 (-0.27 to -0.12)	<0.001
Incidence	Peru	1249.32 (780.63 to 2016.51)	11.73 (7.33 to 18.93)	462.68 (355.81 to 614.49)	3.70 (2.84 to 4.91)	-4.18 (-4.62 to -3.74)	<0.001

Incidence	Philippines	2278.06 (1615.55 to 3234.54)	7.11 (5.04 to 10.09)	1257.87 (979.07 to 1611.73)	2.81 (2.19 to 3.60)	-2.67 (-4.12 to -0.2)	0.035
Incidence	Poland	1145.56 (884.26 to 1473.31)	9.20 (7.10 to 11.83)	525.13 (390.08 to 698.50)	6.82 (5.07 to 9.07)	-0.97 (-1 to -0.96)	<0.001
Incidence	Portugal	437.34 (341.72 to 564.48)	14.67 (11.46 to 18.94)	134.21 (99.79 to 182.06)	7.00 (5.21 to 9.50)	-2.36 (-2.39 to -2.33)	<0.001
Incidence	Puerto Rico	61.76 (47.66 to 77.65)	4.64 (3.58 to 5.83)	35.32 (26.96 to 45.99)	5.41 (4.13 to 7.04)	0.53 (0.2 to 1.04)	0.005
Incidence	Qatar	6.95 (5.55 to 8.74)	4.54 (3.63 to 5.71)	22.91 (17.76 to 29.65)	3.84 (2.97 to 4.97)	-0.55 (-0.6 to -0.51)	<0.001
Incidence	Republic of Korea	2110.31 (1647.90 to 2689.28)	13.25 (10.35 to 16.88)	589.93 (432.24 to 817.04)	7.04 (5.16 to 9.75)	-2.01 (-2.06 to -1.98)	<0.001
Incidence	Republic of Moldova	155.63 (124.18 to 198.12)	9.84 (7.85 to 12.53)	43.76 (33.85 to 56.61)	6.29 (4.86 to 8.13)	-1.42 (-1.62 to -1.2)	<0.001
Incidence	Romania	925.18 (714.76 to 1206.47)	12.33 (9.53 to 16.08)	352.76 (265.69 to 466.54)	8.73 (6.58 to 11.55)	-1.1 (-1.12 to -1.09)	<0.001
Incidence	Russian Federation	4935.76 (3875.19 to 6227.35)	10.92 (8.58 to 13.78)	2277.22 (1738.98 to 3006.95)	6.74 (5.15 to 8.90)	-1.56 (-1.91 to -1.25)	<0.001
Incidence	Rwanda	680.12 (360.06 to 1224.75)	16.43 (8.70 to 29.59)	151.02 (118.55 to 193.02)	2.35 (1.84 to 3.00)	-6.37 (-11.51 to -3)	0.009
Incidence	Saint Kitts and Nevis	0.67 (0.54 to 0.82)	3.64 (2.93 to 4.47)	0.64 (0.51 to 0.81)	4.53 (3.60 to 5.73)	0.78 (0.49 to 1.12)	<0.001

Incidence	Saint Lucia	2.27 (1.84 to 2.79)	3.40 (2.75 to 4.18)	1.54 (1.21 to 1.94)	3.66 (2.87 to 4.62)	0.02 (-0.31 to 0.39)	0.968
Incidence	Saint Vincent and the Grenadines	1.93 (1.56 to 2.37)	3.59 (2.90 to 4.41)	1.26 (0.98 to 1.59)	3.74 (2.90 to 4.72)	0.02 (-0.36 to 0.4)	0.921
Incidence	Samoa	3.54 (2.54 to 5.17)	3.86 (2.77 to 5.65)	2.12 (1.64 to 2.78)	2.10 (1.62 to 2.75)	-0.61 (-1.41 to 0.25)	0.146
Incidence	San Marino	0.65 (0.48 to 0.89)	10.94 (7.97 to 14.96)	0.60 (0.42 to 0.86)	9.58 (6.73 to 13.79)	-0.42 (-0.44 to -0.4)	<0.001
Incidence	Sao Tome and Principe	2.61 (1.98 to 3.36)	3.74 (2.83 to 4.81)	4.53 (3.50 to 5.99)	4.45 (3.43 to 5.88)	0.33 (-0.13 to 0.53)	0.102
Incidence	Saudi Arabia	533.83 (417.65 to 686.33)	6.50 (5.09 to 8.36)	583.91 (446.11 to 796.11)	5.76 (4.40 to 7.86)	-0.5 (-0.71 to -0.22)	0.001
Incidence	Senegal	138.30 (109.29 to 174.68)	3.11 (2.46 to 3.93)	183.78 (142.31 to 238.25)	2.26 (1.75 to 2.94)	-1.12 (-1.58 to -0.66)	<0.001
Incidence	Serbia	277.35 (217.35 to 357.47)	9.56 (7.49 to 12.32)	150.88 (110.06 to 204.49)	8.05 (5.87 to 10.91)	-0.97 (-1.71 to -0.19)	0.013
Incidence	Seychelles	0.73 (0.59 to 0.92)	2.35 (1.89 to 2.93)	0.64 (0.50 to 0.81)	2.09 (1.64 to 2.67)	-0.39 (-0.76 to -0.02)	0.038
Incidence	Sierra Leone	77.38 (61.80 to 96.69)	3.51 (2.80 to 4.38)	113.00 (90.39 to 142.09)	2.48 (1.99 to 3.12)	-1.52 (-2.6 to -0.42)	0.007
Incidence	Singapore	82.89 (63.93 to 106.99)	9.00 (6.94 to 11.62)	73.81 (54.40 to 102.29)	7.07 (5.21 to 9.80)	-0.79 (-0.83 to -0.76)	<0.001

Incidence	Slovakia	178.58 (137.22 to 232.83)	10.17 (7.82 to 13.26)	95.54 (70.91 to 129.35)	8.52 (6.33 to 11.54)	-0.56 (-0.59 to -0.54)	<0.001
Incidence	Slovenia	69.54 (53.06 to 90.22)	12.49 (9.53 to 16.20)	42.30 (30.86 to 56.69)	10.45 (7.62 to 14.00)	-0.66 (-0.76 to -0.53)	<0.001
Incidence	Solomon Islands	4.66 (3.69 to 5.87)	2.40 (1.89 to 3.02)	9.02 (7.09 to 11.59)	2.73 (2.14 to 3.50)	0.43 (-0.14 to 1.01)	0.137
Incidence	Somalia	697.25 (357.55 to 1279.52)	14.73 (7.56 to 27.04)	832.40 (537.78 to 1314.94)	6.51 (4.21 to 10.29)	-3.53 (-6.83 to 3.33)	0.154
Incidence	South Africa	801.11 (639.01 to 1035.90)	4.55 (3.63 to 5.88)	561.34 (444.37 to 721.93)	2.82 (2.23 to 3.62)	-1.52 (-1.72 to -1.34)	<0.001
Incidence	South Sudan	91.23 (72.51 to 113.77)	2.78 (2.21 to 3.46)	319.48 (221.91 to 471.28)	5.82 (4.04 to 8.59)	4.02 (2.14 to 6.18)	0.000
Incidence	Spain	1248.49 (959.89 to 1602.54)	11.20 (8.61 to 14.38)	762.19 (523.89 to 1115.25)	8.65 (5.95 to 12.66)	-0.83 (-0.92 to -0.76)	<0.001
Incidence	Sri Lanka	2509.25 (1199.24 to 4681.21)	34.66 (16.56 to 64.66)	199.62 (155.27 to 262.69)	2.89 (2.25 to 3.81)	-8.03 (-10.57 to -5.44)	<0.001
Incidence	Sudan	1606.12 (910.01 to 2804.59)	14.54 (8.24 to 25.39)	887.71 (712.41 to 1131.18)	4.15 (3.33 to 5.29)	-4.09 (-5.74 to -2.73)	<0.001
Incidence	Suriname	6.13 (5.03 to 7.49)	3.58 (2.94 to 4.38)	6.65 (5.31 to 8.26)	3.50 (2.80 to 4.35)	-0.04 (-0.16 to 0.09)	0.417
Incidence	Sweden	227.69 (167.90 to 309.16)	10.81 (7.97 to 14.67)	193.51 (134.48 to 280.76)	8.01 (5.57 to 11.62)	-0.95 (-0.98 to -0.94)	<0.001

Incidence	Switzerland	240.15 (177.98 to 329.54)	15.23 (11.28 to 20.89)	191.10 (132.67 to 280.46)	10.84 (7.53 to 15.91)	-1.11 (-1.15 to -1.07)	<0.001
Incidence	Syrian Arab Republic	304.53 (240.93 to 393.95)	4.14 (3.28 to 5.36)	526.14 (335.04 to 825.13)	9.67 (6.16 to 15.17)	2.89 (1.49 to 4.14)	<0.001
Incidence	Taiwan (Province of China)	248.84 (196.31 to 314.45)	3.40 (2.69 to 4.30)	83.23 (66.28 to 106.58)	2.06 (1.64 to 2.63)	-1.96 (-2.32 to -1.59)	<0.001
Incidence	Tajikistan	185.87 (147.52 to 239.77)	6.47 (5.13 to 8.34)	195.73 (152.81 to 249.03)	4.39 (3.43 to 5.59)	-2.62 (-3.79 to -1.48)	<0.001
Incidence	Thailand	923.15 (758.29 to 1135.78)	4.06 (3.33 to 4.99)	445.69 (357.83 to 560.91)	3.27 (2.62 to 4.11)	-0.8 (-1.29 to -0.45)	0.028
Incidence	Timor-Leste	91.06 (43.99 to 169.68)	22.39 (10.82 to 41.73)	21.15 (16.10 to 29.01)	3.09 (2.35 to 4.23)	-10.64 (-14.2 to -7.64)	<0.001
Incidence	Togo	63.59 (51.28 to 79.31)	2.95 (2.38 to 3.68)	105.31 (83.93 to 133.23)	2.52 (2.01 to 3.19)	-0.72 (-1.11 to -0.31)	0.002
Incidence	Tokelau	0.02 (0.01 to 0.02)	2.17 (1.69 to 2.76)	0.01 (0.01 to 0.01)	2.05 (1.54 to 2.69)	-0.2 (-0.23 to -0.17)	<0.001
Incidence	Tonga	1.27 (1.00 to 1.63)	2.37 (1.87 to 3.05)	0.85 (0.65 to 1.11)	1.72 (1.31 to 2.24)	-0.6 (-1.02 to -0.16)	0.012
Incidence	Trinidad and Tobago	20.21 (16.14 to 24.91)	3.89 (3.11 to 4.80)	20.47 (15.38 to 28.00)	5.62 (4.23 to 7.69)	1.32 (0.27 to 2.16)	0.015
Incidence	Tunisia	198.94 (158.72 to 249.49)	4.99 (3.98 to 6.25)	136.34 (105.90 to 176.08)	3.80 (2.95 to 4.91)	-0.98 (-1.3 to -0.46)	0.002

Incidence	Turkey	1245.49<br>(994.06 to 1565.10)	4.67<br>(3.73 to 5.87)	852.44<br>(645.39 to 1141.57)	3.45<br>(2.61 to 4.62)	-0.97<br>(-1.63 to -0.31)	0.010
Incidence	Turkmenistan	122.24<br>(97.84 to 154.33)	6.48<br>(5.19 to 8.19)	89.02<br>(69.13 to 112.68)	4.54<br>(3.53 to 5.75)	-1.41<br>(-1.76 to -1.05)	<0.001
Incidence	Tuvalu	0.11<br>(0.09 to 0.14)	2.59<br>(2.08 to 3.21)	0.11<br>(0.08 to 0.14)	2.19<br>(1.69 to 2.81)	-0.54<br>(-0.57 to -0.52)	<0.001
Incidence	Uganda	469.38<br>(338.82 to 667.43)	4.56<br>(3.29 to 6.48)	668.75<br>(534.87 to 854.63)	2.68<br>(2.14 to 3.43)	-1.18<br>(-2.04 to -0.29)	0.013
Incidence	Ukraine	1567.29<br>(1177.46 to 2117.16)	10.41<br>(7.82 to 14.07)	686.48<br>(531.51 to 886.33)	8.13<br>(6.29 to 10.50)	-1.02<br>(-1.4 to -0.63)	<0.001
Incidence	United Arab Emirates	32.69<br>(26.21 to 41.22)	4.62<br>(3.71 to 5.83)	61.02<br>(47.52 to 79.67)	3.60<br>(2.80 to 4.70)	-0.92<br>(-1.11 to -0.73)	<0.001
Incidence	United Kingdom	1501.07<br>(1128.31 to 2014.83)	10.12<br>(7.61 to 13.58)	1135.94<br>(793.08 to 1651.34)	7.26<br>(5.07 to 10.55)	-1.08<br>(-1.12 to -1.04)	<0.001
Incidence	United Republic of Tanzania	461.40<br>(367.35 to 580.21)	3.09<br>(2.46 to 3.89)	816.11<br>(640.52 to 1043.89)	2.64<br>(2.08 to 3.38)	-0.39<br>(-0.49 to -0.29)	<0.001
Incidence	United States of America	7132.44<br>(5466.40 to 9237.67)	9.63<br>(7.38 to 12.48)	4290.52<br>(3356.78 to 5500.36)	5.28<br>(4.13 to 6.76)	-1.96<br>(-2.04 to -1.9)	<0.001
Incidence	United States Virgin Islands	1.77<br>(1.45 to 2.15)	4.24<br>(3.48 to 5.15)	0.62<br>(0.49 to 0.78)	3.41<br>(2.70 to 4.30)	-0.79<br>(-1.12 to -0.48)	0.000
Incidence	Uruguay	105.85<br>(79.96 to 138.64)	9.83<br>(7.42 to 12.87)	81.21<br>(61.57 to 107.08)	8.99<br>(6.82 to 11.85)	-0.29<br>(-0.31 to -0.27)	<0.001

Incidence	Uzbekistan	668.22 (530.11 to 854.33)	6.26 (4.97 to 8.00)	591.58 (467.86 to 746.36)	4.69 (3.71 to 5.92)	-1.06 (-1.19 to -0.93)	<0.001
Incidence	Vanuatu	1.70 (1.34 to 2.16)	2.04 (1.61 to 2.59)	2.81 (2.23 to 3.56)	1.91 (1.51 to 2.41)	-0.33 (-0.92 to 0.26)	0.247
Incidence	Venezuela (Bolivarian Republic of)	715.86 (552.48 to 907.48)	7.89 (6.09 to 10.00)	587.17 (451.44 to 753.49)	6.71 (5.16 to 8.61)	-0.77 (-1.22 to -0.3)	0.003
Incidence	Viet Nam	914.13 (749.41 to 1117.90)	2.72 (2.23 to 3.32)	840.47 (665.72 to 1065.29)	2.66 (2.11 to 3.37)	-0.08 (-0.37 to 0.24)	0.497
Incidence	Yemen	382.30 (309.67 to 476.61)	4.55 (3.68 to 5.67)	4684.47 (2418.07 to 8685.07)	26.81 (13.84 to 49.71)	5.67 (3.97 to 7.92)	<0.001
Incidence	Zambia	145.10 (117.56 to 182.23)	3.09 (2.50 to 3.88)	256.50 (201.31 to 330.52)	2.45 (1.93 to 3.16)	-0.55 (-0.67 to -0.43)	<0.001
Incidence	Zimbabwe	153.17 (124.29 to 191.98)	2.54 (2.06 to 3.19)	188.58 (152.53 to 234.22)	2.36 (1.91 to 2.93)	-0.11 (-0.37 to 0.15)	0.361

AAPC average annual percentage change.

Supplementary Table 15 The prevalence of spinal cord injury and their AAPCs from 1990 to 2021 among children and adolescents at national level							
	**Location**	**Case (n), 1990**	**Rate (per 100, 000), 1990**	**Case (n), 2021**	**Rate (per 100, 000), 2021**	**AAPC, 1990-2021**	***P***
Prevalence	Afghanistan	33662.98 (6614.73 to 117784.09)	604.04 (118.69 to 2113.49)	36789.31 (20028.88 to 68825.75)	206.95 (112.67 to 387.17)	-3.63 (-3.81 to -3.43)	<0.001
Prevalence	Albania	1314.39 (1158.71 to 1511.27)	90.90 (80.14 to 104.52)	547.73 (472.00 to 645.34)	88.76 (76.48 to 104.57)	-0.19 (-0.34 to -0.06)	0.009
Prevalence	Algeria	7210.34 (6419.29 to 8181.11)	53.34 (47.49 to 60.53)	5503.99 (4805.67 to 6463.98)	33.46 (29.22 to 39.30)	-1.49 (-1.56 to -1.43)	<0.001
Prevalence	American Samoa	4.96 (4.38 to 5.75)	20.71 (18.31 to 24.04)	8.39 (5.73 to 13.16)	43.85 (29.94 to 68.78)	2.35 (2.09 to 2.62)	<0.001
Prevalence	Andorra	16.56 (13.83 to 19.92)	123.03 (102.78 to 148.00)	15.74 (13.05 to 19.21)	109.45 (90.75 to 133.60)	-0.38 (-0.41 to -0.36)	<0.001
Prevalence	Angola	6257.00 (3507.96 to 11123.01)	108.51 (60.84 to 192.91)	4041.74 (3607.05 to 4487.16)	21.64 (19.31 to 24.03)	-4.97 (-5.2 to -4.61)	<0.001
Prevalence	Antigua and Barbuda	9.36 (8.31 to 10.48)	39.16 (34.79 to 43.87)	10.00 (8.92 to 11.48)	42.80 (38.18 to 49.14)	0.29 (0.19 to 0.4)	<0.001
Prevalence	Argentina	11503.55 (10252.05 to 13065.80)	88.31 (78.70 to 100.30)	11140.46 (9681.02 to 13054.52)	81.25 (70.61 to 95.21)	-0.28 (-0.33 to -0.25)	<0.001

Prevalence	Armenia	3314.52 (1908.48 to 5926.22)	249.62 (143.73 to 446.31)	974.59 (609.12 to 1588.34)	127.71 (79.82 to 208.14)	-1.53 (-2.15 to -0.66)	0.002
Prevalence	Australia	8188.35 (6905.26 to 9653.92)	158.32 (133.51 to 186.65)	8424.07 (6969.79 to 10089.65)	135.04 (111.73 to 161.74)	-0.5 (-0.53 to -0.48)	<0.001
Prevalence	Austria	2471.21 (2115.61 to 2873.90)	131.92 (112.94 to 153.42)	2018.13 (1695.34 to 2398.32)	115.00 (96.61 to 136.66)	-0.45 (-0.48 to -0.41)	<0.001
Prevalence	Azerbaijan	1737.85 (1542.45 to 1995.37)	55.52 (49.28 to 63.75)	1978.09 (1597.65 to 2515.50)	64.92 (52.44 to 82.56)	0.74 (0.28 to 1.02)	0.005
Prevalence	Bahamas	39.49 (35.35 to 44.60)	36.68 (32.84 to 41.43)	62.40 (48.78 to 82.87)	54.14 (42.32 to 71.90)	1.45 (1.3 to 1.59)	<0.001
Prevalence	Bahrain	73.12 (63.97 to 84.40)	36.54 (31.97 to 42.18)	135.40 (115.83 to 160.53)	33.55 (28.71 to 39.78)	-0.29 (-0.34 to -0.24)	<0.001
Prevalence	Bangladesh	18133.38 (14828.86 to 23055.01)	30.04 (24.57 to 38.20)	15904.61 (13511.72 to 18861.50)	26.08 (22.15 to 30.93)	-0.56 (-0.88 to -0.35)	<0.001
Prevalence	Barbados	31.56 (27.84 to 35.91)	37.17 (32.78 to 42.30)	25.22 (22.30 to 29.27)	37.99 (33.59 to 44.10)	0.07 (0.03 to 0.12)	0.002
Prevalence	Belarus	2537.22 (2215.89 to 2922.45)	80.85 (70.61 to 93.13)	1235.74 (1058.29 to 1442.58)	61.02 (52.25 to 71.23)	-0.9 (-0.95 to -0.87)	<0.001

Prevalence	Belgium	3248.90 (2752.41 to 3853.98)	131.44 (111.36 to 155.92)	2689.31 (2197.45 to 3268.64)	105.70 (86.37 to 128.47)	-0.67 (-0.77 to -0.6)	<0.001
Prevalence	Belize	42.87 (38.33 to 49.13)	41.79 (37.37 to 47.90)	67.44 (58.93 to 78.09)	39.89 (34.86 to 46.19)	-0.18 (-0.27 to -0.08)	0.002
Prevalence	Benin	723.91 (651.77 to 794.41)	25.23 (22.71 to 27.68)	1873.11 (1667.62 to 2111.70)	24.92 (22.19 to 28.09)	-0.03 (-0.06 to -0.02)	<0.001
Prevalence	Bermuda	5.93 (5.25 to 6.78)	37.27 (33.01 to 42.61)	4.73 (4.17 to 5.54)	41.11 (36.26 to 48.16)	0.32 (0.28 to 0.34)	<0.001
Prevalence	Bhutan	82.13 (71.94 to 98.67)	24.11 (21.12 to 28.97)	72.95 (62.49 to 85.96)	28.61 (24.51 to 33.71)	0.64 (0.5 to 0.76)	<0.001
Prevalence	Bolivia (Plurinational State of)	1422.77 (1261.00 to 1606.52)	42.49 (37.65 to 47.97)	1530.85 (1315.77 to 1787.70)	33.67 (28.94 to 39.32)	-0.75 (-0.77 to -0.73)	<0.001
Prevalence	Bosnia and Herzegovina	1600.28 (1363.15 to 1920.90)	108.00 (92.00 to 129.64)	538.98 (461.44 to 633.82)	81.04 (69.38 to 95.30)	-0.75 (-1.05 to -0.39)	0.002
Prevalence	Botswana	168.12 (151.00 to 187.45)	22.70 (20.39 to 25.31)	240.87 (218.40 to 265.58)	26.27 (23.82 to 28.96)	0.48 (0.46 to 0.49)	<0.001
Prevalence	Brazil	44124.00 (37147.66 to 52117.57)	65.67 (55.29 to 77.56)	35197.96 (28429.29 to 43794.44)	55.07 (44.48 to 68.52)	-0.56 (-0.6 to -0.53)	<0.001

Prevalence	Brunei Darussalam	93.05 (82.89 to 104.31)	80.89 (72.06 to 90.69)	92.81 (81.25 to 106.70)	71.87 (62.92 to 82.62)	-0.4 (-0.41 to -0.38)	<0.001
Prevalence	Bulgaria	2852.18 (2451.71 to 3244.73)	120.69 (103.74 to 137.30)	1166.88 (1008.39 to 1329.15)	90.16 (77.91 to 102.69)	-0.94 (-0.96 to -0.93)	<0.001
Prevalence	Burkina Faso	1510.80 (1347.58 to 1690.27)	26.71 (23.82 to 29.88)	4312.50 (3695.95 to 5090.29)	33.68 (28.86 to 39.75)	0.81 (0.77 to 0.85)	<0.001
Prevalence	Burundi	704.99 (630.65 to 784.89)	22.34 (19.99 to 24.88)	2168.29 (1766.62 to 2705.40)	29.81 (24.28 to 37.19)	0.87 (0.63 to 1.07)	<0.001
Prevalence	Cabo Verde	58.21 (52.61 to 64.14)	29.93 (27.05 to 32.98)	53.93 (47.62 to 60.94)	27.98 (24.71 to 31.62)	-0.21 (-0.24 to -0.19)	<0.001
Prevalence	Cambodia	6646.12 (2541.62 to 17578.94)	116.73 (44.64 to 308.76)	1622.73 (1386.12 to 1935.48)	24.47 (20.90 to 29.19)	-4.8 (-4.87 to -4.66)	<0.001
Prevalence	Cameroon	1509.53 (1301.75 to 1769.75)	25.43 (21.93 to 29.81)	5177.02 (4293.94 to 6384.65)	30.63 (25.40 to 37.77)	0.62 (0.52 to 0.7)	<0.001
Prevalence	Canada	4784.45 (4269.18 to 5387.75)	62.42 (55.70 to 70.29)	3631.60 (3224.03 to 4174.92)	44.13 (39.18 to 50.74)	-1.1 (-1.12 to -1.08)	<0.001
Prevalence	Central African Republic	365.80 (332.01 to 405.31)	24.43 (22.18 to 27.07)	1712.64 (1163.05 to 2591.82)	59.18 (40.19 to 89.56)	2.93 (2.5 to 3.19)	<0.001

Prevalence	Chad	2193.33 (1376.84 to 3658.72)	62.22 (39.06 to 103.79)	3091.54 (2687.22 to 3644.38)	28.23 (24.54 to 33.28)	-2.56 (-2.74 to -2.38)	<0.001
Prevalence	Chile	4676.19 (4133.38 to 5375.23)	88.66 (78.36 to 101.91)	5196.76 (4383.68 to 6161.26)	106.19 (89.58 to 125.90)	0.6 (0.55 to 0.64)	<0.001
Prevalence	China	232136.18 (206376.43 to 262069.94)	52.16 (46.37 to 58.89)	116642.53 (101054.42 to 136259.91)	34.89 (30.23 to 40.76)	-1.28 (-1.34 to -1.22)	<0.001
Prevalence	Colombia	15679.59 (12797.62 to 19522.15)	104.16 (85.02 to 129.69)	8804.82 (7475.12 to 10304.71)	60.48 (51.34 to 70.78)	-1.78 (-1.83 to -1.72)	<0.001
Prevalence	Comoros	71.94 (64.72 to 79.92)	27.22 (24.49 to 30.24)	93.95 (79.86 to 114.41)	29.92 (25.44 to 36.44)	0.47 (0.34 to 0.58)	<0.001
Prevalence	Congo	321.88 (290.68 to 355.43)	24.26 (21.91 to 26.79)	524.57 (465.01 to 584.07)	21.09 (18.70 to 23.49)	-0.47 (-0.67 to -0.26)	0.000
Prevalence	Cook Islands	2.76 (2.25 to 3.49)	32.03 (26.10 to 40.57)	1.20 (1.00 to 1.45)	23.22 (19.33 to 27.98)	-1.13 (-1.36 to -0.89)	<0.001
Prevalence	Costa Rica	853.59 (724.11 to 1003.70)	60.19 (51.06 to 70.78)	799.86 (673.22 to 963.00)	58.43 (49.18 to 70.34)	-0.09 (-0.12 to -0.07)	<0.001
Prevalence	Coted'Ivoire	1648.39 (1473.08 to 1822.13)	23.83 (21.29 to 26.34)	3705.35 (3292.68 to 4146.93)	25.76 (22.89 to 28.83)	0.25 (0.21 to 0.28)	<0.001

Prevalence	Croatia	1449.78 (1262.78 to 1651.44)	109.30 (95.20 to 124.50)	738.64 (632.42 to 866.53)	91.67 (78.49 to 107.55)	-0.56 (-0.64 to -0.49)	<0.001
Prevalence	Cuba	1804.57 (1596.71 to 2052.23)	49.41 (43.72 to 56.19)	1120.15 (975.79 to 1303.23)	46.65 (40.64 to 54.27)	-0.17 (-0.23 to -0.13)	<0.001
Prevalence	Cyprus	282.87 (244.78 to 332.55)	109.19 (94.49 to 128.37)	279.75 (234.20 to 332.19)	98.13 (82.15 to 116.52)	-0.34 (-0.37 to -0.31)	<0.001
Prevalence	Czechia	3707.84 (3237.95 to 4209.52)	121.81 (106.38 to 138.29)	2162.87 (1894.77 to 2468.74)	97.40 (85.33 to 111.18)	-0.71 (-0.75 to -0.68)	<0.001
Prevalence	Democratic People's Republic of Korea	2412.06 (2188.87 to 2663.78)	30.97 (28.10 to 34.20)	1345.44 (1208.01 to 1506.29)	20.38 (18.30 to 22.81)	-1.34 (-1.38 to -1.3)	<0.001
Prevalence	Democratic Republic of the Congo	5664.55 (5116.03 to 6307.93)	26.16 (23.63 to 29.14)	13819.58 (11543.55 to 16870.11)	28.85 (24.10 to 35.21)	0.33 (0.18 to 0.47)	<0.001
Prevalence	Denmark	1433.84 (1221.21 to 1683.17)	114.68 (97.67 to 134.62)	1341.60 (1116.44 to 1621.44)	103.66 (86.26 to 125.28)	-0.34 (-0.37 to -0.32)	<0.001
Prevalence	Djibouti	65.94 (58.35 to 75.45)	29.40 (26.02 to 33.64)	137.11 (122.50 to 151.67)	25.95 (23.18 to 28.70)	-0.45 (-0.55 to -0.31)	<0.001
Prevalence	Dominica	10.82 (9.55 to 12.20)	33.01 (29.15 to 37.24)	10.95 (8.46 to 14.79)	56.36 (43.57 to 76.11)	1.79 (1.65 to 1.88)	<0.001

Prevalence	Dominican Republic	1233.39 (1106.16 to 1385.56)	35.22 (31.59 to 39.56)	1336.02 (1191.02 to 1515.46)	34.34 (30.61 to 38.95)	-0.08 (-0.14 to -0.04)	0.000
Prevalence	Ecuador	2851.10 (2267.90 to 3691.74)	57.51 (45.75 to 74.47)	2561.80 (2228.20 to 3003.34)	38.72 (33.68 to 45.39)	-1.26 (-1.33 to -1.18)	<0.001
Prevalence	Egypt	14263.19 (12768.79 to 15875.33)	51.08 (45.73 to 56.85)	15500.98 (13437.02 to 17792.70)	33.30 (28.87 to 38.22)	-1.36 (-1.37 to -1.34)	<0.001
Prevalence	El Salvador	8246.91 (4544.10 to 15497.70)	299.30 (164.92 to 562.45)	1352.51 (1165.20 to 1581.78)	56.78 (48.92 to 66.40)	-5.28 (-5.4 to -5.13)	<0.001
Prevalence	Equatorial Guinea	58.31 (52.83 to 64.32)	24.30 (22.01 to 26.80)	163.73 (144.10 to 187.81)	21.26 (18.71 to 24.39)	-0.43 (-0.45 to -0.41)	<0.001
Prevalence	Eritrea	10174.47 (4415.18 to 20870.68)	517.31 (224.49 to 1061.16)	758.26 (677.40 to 840.13)	23.57 (21.06 to 26.12)	-9.87 (-10.03 to -9.66)	<0.001
Prevalence	Estonia	434.96 (383.25 to 490.03)	94.70 (83.44 to 106.69)	168.96 (145.51 to 195.11)	60.28 (51.91 to 69.61)	-1.44 (-1.47 to -1.42)	<0.001
Prevalence	Eswatini	133.12 (120.32 to 144.91)	27.90 (25.22 to 30.37)	136.65 (123.23 to 152.73)	25.54 (23.03 to 28.55)	-0.3 (-0.37 to -0.26)	<0.001
Prevalence	Ethiopia	19549.42 (13394.99 to 30086.19)	66.34 (45.46 to 102.10)	15936.54 (13652.14 to 18974.76)	27.86 (23.87 to 33.17)	-3.24 (-3.49 to -2.95)	<0.001

Prevalence	Fiji	81.20 (69.47 to 95.50)	22.64 (19.37 to 26.62)	77.43 (65.03 to 92.59)	22.08 (18.55 to 26.41)	-0.05 (-0.1 to 0)	0.040
Prevalence	Finland	1934.81 (1610.21 to 2316.97)	152.50 (126.91 to 182.62)	1671.48 (1364.01 to 2043.81)	145.53 (118.76 to 177.95)	-0.15 (-0.2 to -0.12)	<0.001
Prevalence	France	19614.18 (16764.35 to 22834.66)	121.51 (103.86 to 141.46)	16648.93 (13903.18 to 20007.70)	105.70 (88.26 to 127.02)	-0.45 (-0.47 to -0.42)	<0.001
Prevalence	Gabon	132.64 (118.99 to 145.61)	26.10 (23.41 to 28.65)	184.93 (164.06 to 208.13)	22.36 (19.84 to 25.17)	-0.49 (-0.52 to -0.48)	<0.001
Prevalence	Gambia	162.27 (135.69 to 204.85)	28.88 (24.15 to 36.46)	279.35 (245.38 to 314.81)	22.05 (19.37 to 24.85)	-0.92 (-0.97 to -0.86)	<0.001
Prevalence	Georgia	1399.92 (1204.14 to 1611.42)	77.91 (67.02 to 89.68)	579.16 (492.07 to 681.39)	62.32 (52.95 to 73.32)	-0.62 (-0.78 to -0.45)	<0.001
Prevalence	Germany	19415.11 (16421.76 to 22648.62)	112.03 (94.76 to 130.69)	16406.15 (13701.55 to 19667.45)	102.88 (85.92 to 123.33)	-0.28 (-0.31 to -0.26)	<0.001
Prevalence	Ghana	1798.75 (1605.10 to 2011.33)	21.78 (19.43 to 24.35)	3655.42 (3207.31 to 4175.28)	22.41 (19.66 to 25.59)	0.09 (0.07 to 0.12)	<0.001
Prevalence	Greece	3694.47 (3171.66 to 4278.73)	131.45 (112.85 to 152.24)	1857.74 (1539.60 to 2241.40)	97.50 (80.81 to 117.64)	-0.96 (-0.98 to -0.94)	<0.001

Prevalence	Greenland	12.27 (11.12 to 13.58)	68.56 (62.16 to 75.90)	6.61 (5.83 to 7.67)	42.99 (37.90 to 49.92)	-1.48 (-1.53 to -1.45)	<0.001
Prevalence	Grenada	23.69 (17.12 to 36.61)	56.19 (40.61 to 86.84)	11.48 (10.09 to 13.26)	38.13 (33.52 to 44.05)	-1.38 (-1.47 to -1.25)	<0.001
Prevalence	Guam	12.33 (10.76 to 14.35)	22.78 (19.87 to 26.50)	10.24 (8.88 to 11.85)	21.13 (18.31 to 24.44)	-0.23 (-0.26 to -0.21)	<0.001
Prevalence	Guatemala	5720.26 (3850.80 to 9607.91)	116.48 (78.42 to 195.65)	3877.43 (3339.27 to 4519.20)	58.62 (50.48 to 68.32)	-2.22 (-2.28 to -2.14)	<0.001
Prevalence	Guinea	872.25 (782.87 to 960.29)	26.53 (23.82 to 29.21)	2065.28 (1836.89 to 2301.49)	27.67 (24.61 to 30.83)	0.14 (0.05 to 0.24)	0.009
Prevalence	Guinea-Bissau	167.39 (151.06 to 183.14)	28.46 (25.69 to 31.14)	263.02 (238.41 to 291.50)	23.51 (21.31 to 26.06)	-0.62 (-0.72 to -0.51)	<0.001
Prevalence	Guyana	151.13 (135.57 to 167.90)	39.61 (35.53 to 44.00)	108.51 (96.56 to 122.87)	38.66 (34.40 to 43.77)	-0.08 (-0.15 to -0.05)	<0.001
Prevalence	Haiti	1374.69 (1253.85 to 1508.45)	41.08 (37.47 to 45.08)	11457.64 (5978.20 to 21627.56)	204.32 (106.61 to 385.67)	4.93 (3.63 to 5.99)	<0.001
Prevalence	Honduras	1553.77 (1381.90 to 1788.17)	57.26 (50.93 to 65.90)	2269.45 (1986.67 to 2649.81)	52.17 (45.67 to 60.91)	-0.32 (-0.46 to -0.15)	<0.001

Prevalence	Hungary	3180.08 (2805.25 to 3603.25)	109.57 (96.66 to 124.15)	1763.63 (1517.69 to 2046.09)	94.04 (80.93 to 109.10)	-0.53 (-0.57 to -0.5)	<0.001
Prevalence	Iceland	98.87 (83.93 to 117.39)	116.99 (99.31 to 138.90)	90.69 (75.14 to 109.20)	101.78 (84.33 to 122.55)	-0.46 (-0.5 to -0.41)	<0.001
Prevalence	India	157045.83 (135404.52 to 183756.14)	38.27 (32.99 to 44.77)	149739.81 (127498.82 to 180107.06)	29.91 (25.47 to 35.98)	-0.82 (-0.86 to -0.79)	<0.001
Prevalence	Indonesia	38597.93 (32482.53 to 51248.16)	44.13 (37.14 to 58.59)	27735.46 (24220.94 to 32185.92)	30.74 (26.85 to 35.68)	-1.16 (-1.25 to -1.07)	<0.001
Prevalence	Iran (Islamic Republic of)	28758.95 (24531.97 to 34920.85)	91.27 (77.86 to 110.83)	9976.76 (8769.99 to 11452.92)	38.59 (33.92 to 44.30)	-2.72 (-2.79 to -2.66)	<0.001
Prevalence	Iraq	12997.71 (8330.78 to 20593.94)	126.21 (80.89 to 199.97)	19086.81 (11704.93 to 32717.55)	108.22 (66.37 to 185.51)	-0.45 (-0.85 to -0.09)	0.022
Prevalence	Ireland	1629.14 (1401.30 to 1894.93)	122.60 (105.45 to 142.60)	1422.74 (1169.26 to 1710.99)	107.65 (88.47 to 129.46)	-0.42 (-0.43 to -0.41)	<0.001
Prevalence	Israel	2242.66 (1942.69 to 2606.30)	111.78 (96.83 to 129.91)	3198.86 (2665.57 to 3848.25)	94.83 (79.02 to 114.08)	-0.63 (-0.7 to -0.56)	<0.001

Prevalence	Italy	18991.90 (15981.94 to 22381.40)	139.44 (117.34 to 164.33)	12189.55 (9898.43 to 14869.55)	116.32 (94.45 to 141.89)	-0.59 (-0.61 to -0.57)	<0.001
Prevalence	Jamaica	488.19 (438.56 to 542.86)	44.69 (40.14 to 49.69)	374.03 (321.86 to 445.81)	45.87 (39.47 to 54.68)	0.07 (0.02 to 0.12)	0.009
Prevalence	Japan	34885.76 (30777.89 to 39633.85)	104.56 (92.25 to 118.79)	14411.32 (12489.20 to 16850.72)	67.87 (58.82 to 79.36)	-1.41 (-1.43 to -1.39)	<0.001
Prevalence	Jordan	817.56 (718.30 to 949.33)	38.99 (34.26 to 45.28)	1709.19 (1449.74 to 2057.78)	34.70 (29.44 to 41.78)	-0.36 (-0.39 to -0.34)	<0.001
Prevalence	Kazakhstan	5091.53 (4472.79 to 5785.00)	76.64 (67.33 to 87.08)	3466.70 (3019.63 to 4030.62)	51.56 (44.91 to 59.94)	-1.27 (-1.31 to -1.24)	<0.001
Prevalence	Kenya	3422.94 (3074.97 to 3754.72)	24.82 (22.30 to 27.22)	6156.81 (5450.56 to 6868.96)	25.03 (22.16 to 27.92)	-0.01 (-0.05 to 0.03)	0.642
Prevalence	Kiribati	5.95 (5.33 to 6.58)	16.24 (14.53 to 17.94)	7.32 (6.46 to 8.22)	13.70 (12.08 to 15.38)	-0.61 (-0.71 to -0.5)	<0.001
Prevalence	Kuwait	381.63 (319.24 to 472.16)	55.64 (46.54 to 68.83)	410.16 (355.83 to 483.33)	37.44 (32.48 to 44.12)	-1.33 (-1.5 to -1.2)	<0.001
Prevalence	Kyrgyzstan	1469.96 (1272.11 to 1683.58)	69.62 (60.25 to 79.74)	1202.14 (1048.42 to 1395.66)	42.59 (37.14 to 49.44)	-1.59 (-1.66 to -1.53)	<0.001

Prevalence	Lao People's Democratic Republic	642.66 (573.13 to 743.77)	28.33 (25.27 to 32.79)	585.21 (516.41 to 660.26)	19.56 (17.26 to 22.07)	-1.22 (-1.3 to -1.15)	<0.001
Prevalence	Latvia	701.27 (621.31 to 785.39)	93.20 (82.58 to 104.38)	229.03 (199.37 to 271.47)	59.17 (51.51 to 70.13)	-1.45 (-1.49 to -1.41)	<0.001
Prevalence	Lebanon	4610.51 (1991.91 to 11924.45)	344.29 (148.75 to 890.46)	564.15 (480.68 to 683.75)	33.86 (28.85 to 41.04)	-7.37 (-7.71 to -6.94)	<0.001
Prevalence	Lesotho	205.05 (187.13 to 224.26)	24.49 (22.35 to 26.78)	233.63 (213.47 to 253.32)	27.85 (25.45 to 30.20)	0.4 (0.38 to 0.44)	<0.001
Prevalence	Liberia	737.92 (525.34 to 1090.51)	54.01 (38.45 to 79.82)	600.65 (529.02 to 688.07)	21.53 (18.96 to 24.66)	-2.99 (-3.17 to -2.79)	<0.001
Prevalence	Libya	1437.45 (1163.85 to 1920.89)	62.27 (50.42 to 83.22)	3272.32 (2439.22 to 4672.22)	156.76 (116.85 to 223.82)	3.34 (2.8 to 3.85)	<0.001
Prevalence	Lithuania	941.95 (832.79 to 1062.02)	85.04 (75.19 to 95.88)	320.38 (277.00 to 367.27)	59.80 (51.70 to 68.55)	-1.13 (-1.17 to -1.11)	<0.001
Prevalence	Luxembourg	107.20 (92.04 to 125.07)	121.49 (104.31 to 141.75)	134.56 (110.87 to 163.20)	99.73 (82.18 to 120.96)	-0.63 (-0.67 to -0.61)	<0.001
Prevalence	Madagascar	1578.89 (1417.21 to 1747.55)	23.47 (21.07 to 25.98)	3139.70 (2776.73 to 3536.51)	21.01 (18.58 to 23.67)	-0.36 (-0.38 to -0.34)	<0.001

Prevalence	Malawi	1266.28 (1124.60 to 1416.26)	22.72 (20.18 to 25.41)	2316.88 (2054.34 to 2606.36)	21.88 (19.40 to 24.61)	-0.12 (-0.15 to -0.1)	<0.001
Prevalence	Malaysia	1704.05 (1546.09 to 1885.94)	20.43 (18.53 to 22.61)	2061.37 (1799.26 to 2360.98)	20.10 (17.54 to 23.02)	-0.05 (-0.08 to -0.03)	<0.001
Prevalence	Maldives	34.19 (29.57 to 40.80)	26.64 (23.04 to 31.79)	38.65 (31.14 to 49.34)	29.66 (23.90 to 37.86)	0.35 (0.19 to 0.56)	<0.001
Prevalence	Mali	1380.18 (1230.46 to 1543.91)	27.91 (24.88 to 31.22)	5037.79 (4234.45 to 6150.13)	35.39 (29.75 to 43.21)	0.79 (0.65 to 0.91)	<0.001
Prevalence	Malta	138.37 (116.18 to 164.86)	120.06 (100.80 to 143.05)	90.48 (74.61 to 110.88)	107.77 (88.87 to 132.06)	-0.34 (-0.38 to -0.31)	<0.001
Prevalence	Marshall Islands	4.73 (4.17 to 5.37)	17.66 (15.56 to 20.05)	4.11 (3.62 to 4.68)	17.83 (15.71 to 20.30)	0.03 (0.02 to 0.05)	<0.001
Prevalence	Mauritania	290.10 (263.40 to 320.25)	25.58 (23.23 to 28.24)	540.58 (481.00 to 601.71)	23.18 (20.62 to 25.80)	-0.33 (-0.36 to -0.32)	<0.001
Prevalence	Mauritius	105.58 (94.65 to 119.32)	24.51 (21.97 to 27.70)	66.81 (58.03 to 76.06)	22.50 (19.55 to 25.62)	-0.26 (-0.29 to -0.24)	<0.001
Prevalence	Mexico	37058.43 (31988.30 to 42734.58)	85.45 (73.76 to 98.54)	22224.48 (19103.96 to 26604.75)	51.50 (44.27 to 61.66)	-1.54 (-1.73 to -1.4)	<0.001
Prevalence	Micronesia (Federated States of)	12.46 (11.04 to 13.95)	21.67 (19.21 to 24.26)	10.52 (8.63 to 13.36)	25.39 (20.82 to 32.24)	0.54 (0.47 to 0.63)	<0.001

Prevalence	Monaco	4.97 (4.18 to 6.00)	102.61 (86.21 to 123.87)	6.61 (5.48 to 8.05)	97.71 (80.87 to 118.97)	-0.15 (-0.18 to -0.12)	<0.001
Prevalence	Mongolia	648.97 (577.74 to 732.74)	57.17 (50.90 to 64.55)	636.70 (554.79 to 740.38)	48.50 (42.26 to 56.40)	-0.54 (-0.6 to -0.49)	<0.001
Prevalence	Montenegro	211.87 (182.67 to 243.62)	98.77 (85.15 to 113.57)	124.18 (107.10 to 144.40)	82.57 (71.21 to 96.02)	-0.58 (-0.6 to -0.56)	<0.001
Prevalence	Morocco	6168.98 (5502.20 to 6984.16)	49.43 (44.09 to 55.96)	4261.61 (3710.03 to 5010.40)	33.00 (28.73 to 38.79)	-1.3 (-1.31 to -1.28)	<0.001
Prevalence	Mozambique	11640.17 (5519.44 to 23347.67)	153.75 (72.91 to 308.39)	4803.52 (4200.83 to 5514.92)	27.06 (23.66 to 31.06)	-5.61 (-5.73 to -5.43)	<0.001
Prevalence	Myanmar	10315.95 (6301.42 to 21753.55)	53.98 (32.97 to 113.83)	17820.65 (12042.42 to 27165.83)	85.81 (57.99 to 130.81)	1.63 (1.19 to 2.09)	<0.001
Prevalence	Namibia	548.08 (328.88 to 923.10)	71.96 (43.18 to 121.19)	252.86 (227.96 to 278.10)	23.60 (21.28 to 25.96)	-3.43 (-3.52 to -3.3)	<0.001
Prevalence	Nauru	1.09 (0.97 to 1.24)	20.99 (18.65 to 23.78)	1.17 (1.03 to 1.35)	22.78 (20.04 to 26.34)	0.28 (0.26 to 0.29)	<0.001
Prevalence	Nepal	3384.22 (2857.46 to 4111.80)	32.70 (27.61 to 39.73)	6062.63 (4377.36 to 8816.88)	48.68 (35.15 to 70.79)	1.27 (0.96 to 1.48)	<0.001

Prevalence	Netherlands	3626.21 (3121.93 to 4242.27)	94.60 (81.45 to 110.68)	2918.85 (2457.40 to 3520.58)	79.08 (66.58 to 95.38)	-0.57 (-0.6 to -0.54)	<0.001
Prevalence	New Zealand	1943.06 (1665.19 to 2219.47)	176.47 (151.23 to 201.57)	2153.92 (1790.90 to 2542.08)	165.24 (137.39 to 195.02)	-0.21 (-0.25 to -0.19)	<0.001
Prevalence	Nicaragua	6557.05 (3053.53 to 12988.67)	291.39 (135.70 to 577.21)	1377.56 (1177.90 to 1635.41)	53.00 (45.32 to 62.92)	-5.31 (-5.46 to -5.06)	<0.001
Prevalence	Niger	1314.84 (1162.38 to 1476.51)	27.10 (23.96 to 30.43)	4669.79 (4012.63 to 5474.39)	29.96 (25.74 to 35.12)	0.3 (0.24 to 0.35)	<0.001
Prevalence	Nigeria	13338.36 (11954.81 to 14843.79)	27.49 (24.64 to 30.59)	36700.98 (31854.35 to 42179.59)	28.72 (24.93 to 33.01)	0.15 (0.11 to 0.19)	<0.001
Prevalence	Niue	0.21 (0.19 to 0.25)	20.90 (18.14 to 24.17)	0.16 (0.13 to 0.20)	30.10 (25.02 to 37.54)	1.14 (1.01 to 1.29)	<0.001
Prevalence	North Macedonia	594.46 (522.30 to 681.78)	85.30 (74.94 to 97.83)	357.03 (305.49 to 419.02)	79.95 (68.41 to 93.83)	-0.22 (-0.26 to -0.19)	<0.001
Prevalence	Northern Mariana Islands	4.00 (3.46 to 4.72)	24.65 (21.32 to 29.13)	4.26 (3.61 to 5.11)	28.40 (24.07 to 34.09)	0.49 (0.39 to 0.58)	<0.001
Prevalence	Norway	1283.57 (1103.69 to 1474.77)	115.37 (99.20 to 132.56)	1121.35 (949.03 to 1346.96)	90.33 (76.44 to 108.50)	-0.77 (-0.8 to -0.74)	<0.001

Prevalence	Oman	548.55 (495.27 to 612.44)	54.73 (49.41 to 61.10)	588.19 (516.91 to 678.02)	39.43 (34.65 to 45.45)	-1.04 (-1.07 to -1.02)	<0.001
Prevalence	Pakistan	13927.34 (12397.79 to 15809.71)	22.85 (20.34 to 25.93)	33790.87 (27859.69 to 43203.31)	30.94 (25.51 to 39.55)	0.98 (0.86 to 1.12)	<0.001
Prevalence	Palau	1.86 (1.62 to 2.21)	30.24 (26.21 to 35.81)	1.25 (1.06 to 1.51)	28.15 (23.95 to 34.21)	-0.24 (-0.26 to -0.22)	<0.001
Prevalence	Palestine	2923.75 (1440.46 to 5409.38)	244.96 (120.68 to 453.21)	1576.99 (1102.81 to 2344.79)	65.49 (45.80 to 97.38)	-4.18 (-4.37 to -3.97)	<0.001
Prevalence	Panama	914.49 (660.23 to 1673.91)	84.13 (60.74 to 153.99)	812.42 (695.89 to 961.17)	53.90 (46.17 to 63.77)	-1.39 (-1.43 to -1.34)	<0.001
Prevalence	Papua New Guinea	502.24 (439.75 to 580.05)	23.59 (20.66 to 27.25)	1255.90 (1089.30 to 1447.97)	25.64 (22.23 to 29.56)	0.13 (-0.01 to 0.3)	0.065
Prevalence	Paraguay	1006.39 (811.86 to 1236.76)	48.64 (39.24 to 59.77)	1095.10 (901.37 to 1370.45)	41.00 (33.75 to 51.31)	-0.54 (-0.57 to -0.52)	<0.001
Prevalence	Peru	7904.27 (5984.08 to 11408.22)	74.22 (56.19 to 107.12)	4400.67 (3772.30 to 5129.72)	35.18 (30.15 to 41.00)	-2.4 (-2.45 to -2.33)	<0.001
Prevalence	Philippines	15878.40 (11421.97 to 24752.38)	49.54 (35.64 to 77.22)	14260.12 (11420.72 to 18095.59)	31.85 (25.51 to 40.41)	-1.55 (-1.69 to -1.41)	<0.001

Prevalence	Poland	10793.05 (9455.90 to 12334.10)	86.69 (75.95 to 99.07)	4819.66 (4150.13 to 5615.04)	62.60 (53.91 to 72.94)	-1.06 (-1.08 to -1.03)	<0.001
Prevalence	Portugal	4329.20 (3801.77 to 4839.80)	145.23 (127.53 to 162.36)	1488.22 (1285.41 to 1722.70)	77.64 (67.06 to 89.88)	-2 (-2.02 to -1.98)	<0.001
Prevalence	Puerto Rico	644.72 (566.08 to 743.08)	48.40 (42.50 to 55.78)	390.99 (313.32 to 552.57)	59.83 (47.95 to 84.56)	0.69 (0.58 to 0.77)	<0.001
Prevalence	Qatar	59.58 (53.13 to 67.78)	38.92 (34.71 to 44.28)	188.96 (162.43 to 224.95)	31.65 (27.20 to 37.67)	-0.66 (-0.69 to -0.64)	<0.001
Prevalence	Republic of Korea	21472.43 (18906.68 to 23956.55)	134.82 (118.71 to 150.41)	6654.32 (5716.77 to 7800.27)	79.38 (68.19 to 93.05)	-1.71 (-1.74 to -1.69)	<0.001
Prevalence	Republic of Moldova	1512.85 (1333.82 to 1710.59)	95.67 (84.35 to 108.18)	449.15 (395.11 to 518.90)	64.51 (56.75 to 74.53)	-1.26 (-1.29 to -1.23)	<0.001
Prevalence	Romania	9550.38 (8327.66 to 10809.59)	127.29 (110.99 to 144.07)	3638.86 (3140.09 to 4172.00)	90.05 (77.71 to 103.25)	-1.12 (-1.14 to -1.09)	<0.001
Prevalence	Russian Federation	43572.19 (38475.75 to 49048.04)	96.43 (85.15 to 108.55)	19283.94 (16608.73 to 22768.69)	57.07 (49.15 to 67.38)	-1.68 (-1.73 to -1.64)	<0.001
Prevalence	Rwanda	1318.52 (1131.26 to 1602.17)	31.86 (27.33 to 38.71)	1587.52 (1407.74 to 1789.45)	24.66 (21.87 to 27.79)	-0.89 (-1.58 to -0.41)	0.000

Prevalence	Saint Kitts and Nevis	6.80 (6.08 to 7.62)	37.03 (33.09 to 41.45)	6.07 (5.35 to 6.98)	43.17 (38.05 to 49.61)	0.51 (0.46 to 0.54)	<0.001
Prevalence	Saint Lucia	23.23 (20.68 to 26.42)	34.78 (30.97 to 39.56)	16.16 (14.34 to 18.69)	38.52 (34.17 to 44.55)	0.31 (0.28 to 0.33)	<0.001
Prevalence	Saint Vincent and the Grenadines	20.10 (17.91 to 22.70)	37.39 (33.32 to 42.23)	12.86 (11.37 to 14.63)	38.15 (33.73 to 43.40)	0.07 (0.02 to 0.11)	0.002
Prevalence	Samoa	19.14 (16.82 to 21.73)	20.90 (18.36 to 23.72)	40.44 (26.22 to 66.09)	40.06 (25.97 to 65.48)	1.98 (1.73 to 2.27)	<0.001
Prevalence	San Marino	7.11 (5.96 to 8.49)	118.86 (99.58 to 141.91)	6.60 (5.47 to 8.06)	105.71 (87.66 to 129.11)	-0.38 (-0.4 to -0.37)	<0.001
Prevalence	Sao Tome and Principe	25.68 (22.31 to 29.33)	36.75 (31.93 to 41.97)	32.18 (27.95 to 37.24)	31.61 (27.46 to 36.58)	-0.5 (-0.52 to -0.48)	<0.001
Prevalence	Saudi Arabia	4665.99 (4041.36 to 5460.29)	56.82 (49.22 to 66.50)	4727.12 (4061.94 to 5640.59)	46.65 (40.08 to 55.66)	-0.65 (-0.68 to -0.62)	<0.001
Prevalence	Senegal	1025.13 (909.81 to 1152.17)	23.05 (20.46 to 25.91)	1781.50 (1560.13 to 2060.90)	21.95 (19.22 to 25.40)	-0.14 (-0.19 to -0.11)	<0.001
Prevalence	Serbia	2790.52 (2461.98 to 3163.54)	96.19 (84.86 to 109.05)	1636.68 (1400.13 to 1949.53)	87.34 (74.72 to 104.04)	-0.33 (-0.6 to -0.1)	0.007
Prevalence	Seychelles	6.98 (6.22 to 7.87)	22.35 (19.92 to 25.20)	5.71 (5.01 to 6.47)	18.71 (16.40 to 21.18)	-0.58 (-0.65 to -0.51)	<0.001

Prevalence	Sierra Leone	620.99 (557.70 to 686.01)	28.15 (25.28 to 31.10)	1262.83 (1091.51 to 1458.04)	27.75 (23.99 to 32.04)	0 (-0.17 to 0.14)	0.891
Prevalence	Singapore	841.37 (734.45 to 945.36)	91.36 (79.75 to 102.65)	748.61 (645.41 to 879.24)	71.75 (61.86 to 84.27)	-0.81 (-0.86 to -0.76)	<0.001
Prevalence	Slovakia	1676.54 (1440.45 to 1925.62)	95.52 (82.07 to 109.71)	893.61 (761.51 to 1053.36)	79.71 (67.93 to 93.96)	-0.61 (-0.63 to -0.59)	<0.001
Prevalence	Slovenia	733.22 (639.18 to 829.23)	131.69 (114.80 to 148.94)	433.12 (370.54 to 501.08)	106.99 (91.53 to 123.78)	-0.67 (-0.7 to -0.64)	<0.001
Prevalence	Solomon Islands	53.99 (42.32 to 72.58)	27.74 (21.75 to 37.29)	93.26 (73.66 to 123.27)	28.20 (22.27 to 37.27)	-0.04 (-0.16 to 0.09)	0.567
Prevalence	Somalia	2987.68 (1271.05 to 10297.96)	63.13 (26.86 to 217.61)	6869.53 (4622.98 to 10498.42)	53.73 (36.16 to 82.12)	-1.59 (-1.98 to -1.02)	0.002
Prevalence	South Africa	8670.56 (7150.83 to 13455.49)	49.20 (40.58 to 76.36)	5201.62 (4728.17 to 5680.94)	26.12 (23.74 to 28.52)	-2.02 (-2.06 to -1.97)	<0.001
Prevalence	South Sudan	1813.45 (1225.26 to 2876.25)	55.19 (37.29 to 87.53)	3001.98 (2017.80 to 4615.12)	54.71 (36.77 to 84.10)	0.22 (-0.01 to 0.42)	0.065
Prevalence	Spain	12195.03 (10670.03 to 13778.53)	109.40 (95.72 to 123.61)	8535.25 (7071.43 to 10403.20)	96.91 (80.29 to 118.12)	-0.39 (-0.42 to -0.35)	<0.001

Prevalence	Sri Lanka	6305.88 (3822.51 to 10601.67)	87.10 (52.80 to 146.43)	4673.68 (3133.93 to 6847.39)	67.74 (45.42 to 99.24)	-0.82 (-1.23 to -0.46)	<0.001
Prevalence	Sudan	7184.99 (5457.04 to 10249.45)	65.05 (49.40 to 92.79)	9178.89 (7602.53 to 11265.47)	42.94 (35.57 to 52.70)	-1.38 (-1.49 to -1.25)	<0.001
Prevalence	Suriname	127.17 (70.01 to 329.38)	74.38 (40.94 to 192.63)	66.01 (58.46 to 74.57)	34.77 (30.79 to 39.28)	-2.27 (-2.35 to -2.15)	<0.001
Prevalence	Sweden	2390.52 (2045.43 to 2783.18)	113.45 (97.08 to 132.09)	2104.48 (1758.26 to 2514.59)	87.13 (72.80 to 104.11)	-0.83 (-0.88 to -0.79)	<0.001
Prevalence	Switzerland	2218.03 (1858.67 to 2601.46)	140.63 (117.85 to 164.94)	2042.67 (1693.97 to 2452.29)	115.89 (96.11 to 139.13)	-0.62 (-0.67 to -0.57)	<0.001
Prevalence	Syrian Arab Republic	3668.18 (2717.60 to 5943.74)	49.89 (36.96 to 80.85)	24376.99 (11162.74 to 50189.58)	448.10 (205.19 to 922.59)	7.57 (5.87 to 8.52)	<0.001
Prevalence	Taiwan (Province of China)	2418.88 (2140.08 to 2752.89)	33.09 (29.28 to 37.66)	922.54 (829.12 to 1021.05)	22.80 (20.50 to 25.24)	-1.27 (-1.39 to -1.16)	<0.001
Prevalence	Tajikistan	1553.56 (1336.34 to 1777.92)	54.05 (46.49 to 61.85)	1708.32 (1488.59 to 1985.86)	38.34 (33.41 to 44.57)	-0.8 (-1.19 to -0.36)	0.001

Prevalence	Thailand	7661.96 (6845.34 to 8567.77)	33.68 (30.09 to 37.67)	3935.38 (3409.21 to 4569.78)	28.83 (24.98 to 33.48)	-0.53 (-0.57 to -0.5)	<0.001
Prevalence	Timor-Leste	672.37 (327.82 to 1273.36)	165.34 (80.61 to 313.12)	119.57 (104.94 to 134.40)	17.44 (15.31 to 19.61)	-7.14 (-7.36 to -6.82)	<0.001
Prevalence	Togo	562.78 (499.89 to 623.98)	26.14 (23.22 to 28.99)	990.94 (888.38 to 1105.90)	23.73 (21.28 to 26.49)	-0.33 (-0.42 to -0.23)	<0.001
Prevalence	Tokelau	0.14 (0.13 to 0.16)	18.84 (16.65 to 21.68)	0.09 (0.08 to 0.11)	18.47 (15.88 to 21.57)	-0.07 (-0.12 to -0.02)	0.004
Prevalence	Tonga	10.60 (9.27 to 12.24)	19.86 (17.38 to 22.94)	8.80 (7.54 to 10.39)	17.75 (15.20 to 20.96)	-0.5 (-0.62 to -0.42)	<0.001
Prevalence	Trinidad and Tobago	194.17 (174.46 to 219.31)	37.38 (33.58 to 42.22)	171.33 (145.08 to 210.91)	47.07 (39.86 to 57.95)	0.92 (0.81 to 1.01)	<0.001
Prevalence	Tunisia	2112.68 (1845.60 to 2548.44)	52.95 (46.25 to 63.87)	1283.70 (1112.19 to 1507.92)	35.80 (31.02 to 42.06)	-1.25 (-1.28 to -1.22)	<0.001
Prevalence	Turkey	11514.63 (10059.65 to 13305.52)	43.16 (37.70 to 49.87)	8929.98 (7649.46 to 10780.39)	36.14 (30.95 to 43.62)	-0.61 (-0.67 to -0.55)	<0.001
Prevalence	Turkmenistan	1156.03 (1012.74 to 1328.53)	61.32 (53.72 to 70.47)	831.39 (725.88 to 961.41)	42.41 (37.02 to 49.04)	-1.17 (-1.21 to -1.13)	<0.001

Prevalence	Tuvalu	0.86 (0.77 to 0.95)	20.05 (18.08 to 22.16)	0.86 (0.75 to 1.00)	17.51 (15.17 to 20.28)	-0.46 (-0.49 to -0.43)	<0.001
Prevalence	Uganda	9428.56 (4786.51 to 18402.16)	91.56 (46.48 to 178.70)	6142.98 (5447.53 to 6888.81)	24.63 (21.84 to 27.62)	-4.19 (-4.32 to -4.04)	<0.001
Prevalence	Ukraine	13908.72 (11922.16 to 16031.59)	92.41 (79.21 to 106.51)	5664.59 (4822.41 to 6594.11)	67.07 (57.10 to 78.08)	-1.04 (-1.12 to -0.98)	<0.001
Prevalence	United Arab Emirates	281.94 (250.69 to 320.56)	39.88 (35.46 to 45.34)	526.81 (460.69 to 617.13)	31.08 (27.18 to 36.41)	-0.8 (-0.86 to -0.75)	<0.001
Prevalence	United Kingdom	14832.78 (12724.88 to 17228.68)	99.99 (85.78 to 116.14)	12036.17 (10067.19 to 14576.13)	76.89 (64.31 to 93.11)	-0.83 (-0.88 to -0.8)	<0.001
Prevalence	United Republic of Tanzania	3908.07 (3507.47 to 4376.32)	26.19 (23.50 to 29.33)	7933.06 (7049.56 to 8997.22)	25.71 (22.84 to 29.16)	-0.07 (-0.09 to -0.05)	<0.001
Prevalence	United States of America	62268.71 (55373.15 to 70284.42)	84.10 (74.78 to 94.92)	38446.02 (34397.99 to 43732.09)	47.28 (42.30 to 53.78)	-1.87 (-1.93 to -1.82)	<0.001
Prevalence	United States Virgin Islands	17.02 (15.34 to 18.99)	40.80 (36.77 to 45.53)	6.70 (5.98 to 7.56)	36.75 (32.84 to 41.47)	-0.36 (-0.39 to -0.32)	<0.001
Prevalence	Uruguay	964.96 (850.10 to 1113.67)	89.59 (78.93 to 103.40)	739.79 (642.15 to 869.89)	81.90 (71.09 to 96.30)	-0.3 (-0.32 to -0.28)	<0.001

Prevalence	Uzbekistan	6249.67 (5416.58 to 7188.17)	58.54 (50.74 to 67.33)	5241.56 (4570.58 to 6050.16)	41.57 (36.25 to 47.98)	-1.09 (-1.14 to -1.04)	<0.001
Prevalence	Vanuatu	23.79 (17.53 to 33.88)	28.51 (21.01 to 40.60)	26.98 (23.16 to 31.84)	18.30 (15.70 to 21.59)	-1.44 (-1.61 to -1.27)	<0.001
Prevalence	Venezuela (Bolivarian Republic of)	6786.51 (5977.20 to 7846.42)	74.77 (65.86 to 86.45)	5452.46 (4720.33 to 6316.03)	62.30 (53.94 to 72.17)	-0.7 (-0.83 to -0.59)	<0.001
Prevalence	Viet Nam	8251.60 (7436.55 to 9238.69)	24.51 (22.09 to 27.44)	7328.59 (6399.73 to 8390.83)	23.18 (20.24 to 26.54)	-0.19 (-0.23 to -0.16)	<0.001
Prevalence	Yemen	5008.01 (4078.27 to 6811.88)	59.56 (48.50 to 81.02)	27980.64 (17288.08 to 47569.18)	160.14 (98.95 to 272.26)	3.68 (3.22 to 4.02)	<0.001
Prevalence	Zambia	1204.44 (1078.02 to 1331.72)	25.62 (22.93 to 28.33)	2332.80 (2083.01 to 2629.09)	22.31 (19.93 to 25.15)	-0.45 (-0.47 to -0.43)	<0.001
Prevalence	Zimbabwe	1382.78 (1247.17 to 1520.65)	22.96 (20.71 to 25.25)	1659.71 (1483.55 to 1841.29)	20.79 (18.58 to 23.06)	-0.31 (-0.34 to -0.29)	<0.001

AAPC average annual percentage change.

Supplementary Table 16 The YLDs of spinal cord injury and their AAPCs from 1990 to 2021 among children and adolescents at national level							
	**Location**	**Case (n), 1990**	**Rate (per 100, 000), 1990**	**Case (n), 2021**	**Rate (per 100, 000), 2021**	**AAPC, 1990-2021**	***P***
YLDs	Afghanistan	13623.13 (2703.37 to 46601.02)	244.45 (48.51 to 836.20)	14259.82 (6984.09 to 26402.39)	80.22 (39.29 to 148.52)	-3.76 (-3.94 to -3.58)	<0.001
YLDs	Albania	458.23 (318.36 to 618.63)	31.69 (22.02 to 42.79)	160.29 (104.26 to 221.01)	25.97 (16.89 to 35.81)	-0.78 (-0.99 to -0.64)	<0.001
YLDs	Algeria	2621.01 (1726.86 to 3539.30)	19.39 (12.78 to 26.18)	1733.76 (1129.45 to 2530.16)	10.54 (6.87 to 15.38)	-1.98 (-2.07 to -1.9)	<0.001
YLDs	American Samoa	1.82 (1.14 to 2.69)	7.59 (4.79 to 11.23)	2.91 (1.71 to 5.00)	15.20 (8.94 to 26.16)	2.17 (1.89 to 2.43)	<0.001
YLDs	Andorra	4.87 (3.15 to 6.99)	36.20 (23.41 to 51.91)	4.62 (2.98 to 6.53)	32.15 (20.70 to 45.39)	-0.4 (-0.44 to -0.37)	<0.001
YLDs	Angola	2616.65 (1372.88 to 4804.19)	45.38 (23.81 to 83.32)	1545.91 (1039.34 to 2177.77)	8.28 (5.57 to 11.66)	-5.16 (-5.37 to -4.84)	<0.001
YLDs	Antigua and Barbuda	3.23 (2.16 to 4.51)	13.54 (9.03 to 18.88)	3.19 (2.01 to 4.62)	13.67 (8.60 to 19.78)	0.03 (-0.12 to 0.2)	0.6034793
YLDs	Argentina	4106.41 (2779.70 to 5521.41)	31.52 (21.34 to 42.38)	3610.23 (2463.51 to 5057.62)	26.33 (17.97 to 36.89)	-0.61 (-0.66 to -0.57)	<0.001

YLDs	Armenia	1128.39 (592.87 to 2125.96)	84.98 (44.65 to 160.11)	299.04 (164.93 to 519.92)	39.19 (21.61 to 68.13)	-2.01 (-3.11 to -1.29)	<0.001
YLDs	Australia	2399.52 (1562.17 to 3430.64)	46.39 (30.20 to 66.33)	2476.63 (1628.93 to 3466.90)	39.70 (26.11 to 55.57)	-0.49 (-0.52 to -0.46)	<0.001
YLDs	Austria	732.67 (490.44 to 1006.10)	39.11 (26.18 to 53.71)	581.68 (384.25 to 800.94)	33.15 (21.90 to 45.64)	-0.55 (-0.59 to -0.52)	<0.001
YLDs	Azerbaijan	634.41 (422.63 to 870.15)	20.27 (13.50 to 27.80)	651.41 (414.87 to 999.50)	21.38 (13.62 to 32.81)	0.42 (-0.06 to 0.76)	0.06678664
YLDs	Bahamas	14.07 (9.35 to 20.02)	13.07 (8.69 to 18.60)	20.52 (13.00 to 30.98)	17.80 (11.27 to 26.88)	1.26 (0.99 to 1.42)	<0.001
YLDs	Bahrain	25.67 (16.60 to 36.50)	12.83 (8.30 to 18.24)	39.10 (24.24 to 58.44)	9.69 (6.01 to 14.48)	-0.92 (-0.98 to -0.84)	<0.001
YLDs	Bangladesh	7310.72 (4291.49 to 11048.68)	12.11 (7.11 to 18.30)	5569.26 (3482.40 to 8113.64)	9.13 (5.71 to 13.30)	-1.03 (-1.27 to -0.71)	<0.001
YLDs	Barbados	10.95 (6.98 to 15.57)	12.90 (8.22 to 18.34)	7.87 (4.88 to 11.31)	11.85 (7.35 to 17.03)	-0.23 (-0.29 to -0.17)	<0.001
YLDs	Belarus	828.20 (544.59 to 1144.08)	26.39 (17.35 to 36.46)	351.44 (227.64 to 500.19)	17.35 (11.24 to 24.70)	-1.31 (-1.4 to -1.26)	<0.001

YLDs	Belgium	959.64 (647.19 to 1375.66)	38.82 (26.18 to 55.66)	781.27 (502.96 to 1116.67)	30.71 (19.77 to 43.89)	-0.73 (-0.81 to -0.66)	<0.001
YLDs	Belize	15.59 (10.25 to 21.42)	15.20 (9.99 to 20.88)	22.92 (14.66 to 32.87)	13.56 (8.67 to 19.44)	-0.38 (-0.48 to -0.28)	<0.001
YLDs	Benin	292.98 (193.34 to 404.50)	10.21 (6.74 to 14.10)	714.23 (448.29 to 1035.91)	9.50 (5.96 to 13.78)	-0.23 (-0.27 to -0.2)	<0.001
YLDs	Bermuda	1.92 (1.20 to 2.74)	12.09 (7.57 to 17.23)	1.33 (0.86 to 1.91)	11.56 (7.45 to 16.58)	-0.14 (-0.16 to -0.11)	<0.001
YLDs	Bhutan	33.02 (20.86 to 47.55)	9.70 (6.13 to 13.96)	25.73 (16.75 to 37.30)	10.09 (6.57 to 14.63)	0.19 (0.03 to 0.32)	0.02159568
YLDs	Bolivia (Plurinational State of)	576.17 (378.94 to 803.11)	17.21 (11.32 to 23.98)	550.56 (350.30 to 783.41)	12.11 (7.70 to 17.23)	-1.14 (-1.17 to -1.11)	<0.001
YLDs	Bosnia and Herzegovina	544.75 (375.12 to 754.56)	36.76 (25.32 to 50.92)	157.49 (101.20 to 215.93)	23.68 (15.22 to 32.47)	-1.19 (-1.58 to -0.77)	<0.001
YLDs	Botswana	65.89 (42.29 to 90.64)	8.90 (5.71 to 12.24)	88.30 (57.44 to 126.12)	9.63 (6.26 to 13.75)	0.26 (0.22 to 0.29)	<0.001
YLDs	Brazil	16163.16 (11100.41 to 21514.43)	24.05 (16.52 to 32.02)	11501.85 (7514.23 to 15871.84)	18.00 (11.76 to 24.83)	-0.92 (-0.96 to -0.89)	<0.001

YLDs	Brunei Darussalam	33.78 (21.86 to 45.39)	29.37 (19.00 to 39.46)	30.55 (20.95 to 41.14)	23.66 (16.23 to 31.86)	-0.69 (-0.73 to -0.66)	<0.001
YLDs	Bulgaria	931.23 (629.89 to 1250.59)	39.40 (26.65 to 52.92)	352.22 (242.04 to 488.74)	27.21 (18.70 to 37.76)	-1.18 (-1.21 to -1.15)	<0.001
YLDs	Burkina Faso	602.99 (417.86 to 850.29)	10.66 (7.39 to 15.03)	1676.31 (1071.15 to 2432.30)	13.09 (8.36 to 18.99)	0.68 (0.62 to 0.73)	<0.001
YLDs	Burundi	295.40 (189.25 to 413.85)	9.36 (6.00 to 13.12)	858.34 (548.83 to 1234.85)	11.80 (7.54 to 16.97)	0.7 (0.27 to 1.1)	0.00439912
YLDs	Cabo Verde	21.55 (13.64 to 30.98)	11.08 (7.02 to 15.93)	17.91 (11.32 to 26.01)	9.29 (5.87 to 13.50)	-0.58 (-0.62 to -0.55)	<0.001
YLDs	Cambodia	2705.97 (983.16 to 7315.43)	47.53 (17.27 to 128.49)	597.59 (383.48 to 833.79)	9.01 (5.78 to 12.57)	-5.23 (-5.34 to -5.07)	<0.001
YLDs	Cameroon	597.54 (392.51 to 841.96)	10.06 (6.61 to 14.18)	1952.73 (1230.54 to 2844.84)	11.55 (7.28 to 16.83)	0.5 (0.44 to 0.57)	<0.001
YLDs	Canada	1392.16 (910.06 to 1971.54)	18.16 (11.87 to 25.72)	1059.13 (653.74 to 1527.92)	12.87 (7.94 to 18.57)	-1.11 (-1.14 to -1.09)	<0.001
YLDs	Central African Republic	155.09 (100.99 to 214.52)	10.36 (6.75 to 14.33)	725.14 (422.68 to 1202.93)	25.06 (14.61 to 41.57)	2.86 (2.62 to 3.06)	<0.001

YLDs	Chad	902.03 (492.08 to 1570.00)	25.59 (13.96 to 44.54)	1233.78 (781.95 to 1733.02)	11.27 (7.14 to 15.83)	-2.72 (-2.89 to -2.54)	<0.001
YLDs	Chile	1635.48 (1115.41 to 2251.92)	31.01 (21.15 to 42.69)	1565.16 (995.38 to 2162.09)	31.98 (20.34 to 44.18)	0.11 (0.06 to 0.16)	0.00039992
YLDs	China	85418.93 (59423.17 to 110191.82)	19.19 (13.35 to 24.76)	33874.54 (22733.04 to 46017.78)	10.13 (6.80 to 13.77)	-2.03 (-2.09 to -1.97)	<0.001
YLDs	Colombia	5716.11 (3796.35 to 8169.86)	37.97 (25.22 to 54.27)	2760.69 (1844.32 to 3833.95)	18.96 (12.67 to 26.33)	-2.25 (-2.33 to -2.18)	<0.001
YLDs	Comoros	29.34 (19.27 to 40.20)	11.10 (7.29 to 15.21)	35.29 (22.18 to 51.18)	11.24 (7.07 to 16.30)	0.1 (-0.05 to 0.23)	0.16956609
YLDs	Congo	132.11 (84.79 to 184.29)	9.96 (6.39 to 13.89)	197.90 (128.54 to 283.36)	7.96 (5.17 to 11.39)	-0.72 (-1.27 to -0.04)	0.03959208
YLDs	Cook Islands	0.96 (0.59 to 1.45)	11.20 (6.88 to 16.85)	0.36 (0.22 to 0.53)	6.99 (4.18 to 10.27)	-1.51 (-1.85 to -1.16)	<0.001
YLDs	Costa Rica	285.25 (191.91 to 401.44)	20.11 (13.53 to 28.31)	239.74 (156.57 to 338.67)	17.51 (11.44 to 24.74)	-0.45 (-0.47 to -0.42)	<0.001
YLDs	Coted'Ivoire	652.39 (432.79 to 907.44)	9.43 (6.26 to 13.12)	1388.43 (876.69 to 1936.61)	9.65 (6.09 to 13.46)	0.06 (0.01 to 0.09)	0.0139972

YLDs	Croatia	436.88 (287.65 to 613.97)	32.94 (21.69 to 46.29)	206.26 (138.96 to 288.52)	25.60 (17.25 to 35.81)	-0.84 (-0.92 to -0.76)	<0.001
YLDs	Cuba	595.81 (384.50 to 836.13)	16.31 (10.53 to 22.89)	335.30 (218.62 to 483.29)	13.96 (9.10 to 20.13)	-0.51 (-0.57 to -0.45)	<0.001
YLDs	Cyprus	89.15 (60.95 to 119.57)	34.41 (23.53 to 46.16)	81.28 (53.31 to 116.16)	28.51 (18.70 to 40.75)	-0.6 (-0.63 to -0.57)	<0.001
YLDs	Czechia	1166.91 (776.91 to 1565.20)	38.34 (25.52 to 51.42)	604.17 (405.47 to 833.94)	27.21 (18.26 to 37.56)	-1.1 (-1.15 to -1.05)	<0.001
YLDs	Democratic People's Republic of Korea	883.80 (590.02 to 1242.68)	11.35 (7.57 to 15.95)	453.16 (273.71 to 660.20)	6.86 (4.15 to 10.00)	-1.61 (-1.64 to -1.57)	<0.001
YLDs	Democratic Republic of the Congo	2299.12 (1464.58 to 3209.58)	10.62 (6.76 to 14.82)	5315.02 (3303.74 to 7962.96)	11.09 (6.90 to 16.62)	0.18 (-0.09 to 0.46)	0.15276945
YLDs	Denmark	432.70 (290.09 to 601.96)	34.61 (23.20 to 48.15)	395.00 (261.09 to 566.62)	30.52 (20.17 to 43.78)	-0.43 (-0.46 to -0.4)	<0.001
YLDs	Djibouti	26.66 (17.49 to 37.41)	11.89 (7.80 to 16.68)	52.37 (33.87 to 72.71)	9.91 (6.41 to 13.76)	-0.62 (-0.76 to -0.46)	<0.001
YLDs	Dominica	3.91 (2.57 to 5.51)	11.94 (7.84 to 16.81)	3.77 (2.21 to 5.67)	19.43 (11.39 to 29.17)	1.61 (1.52 to 1.69)	<0.001

YLDs	Dominican Republic	471.22 (318.05 to 647.58)	13.46 (9.08 to 18.49)	464.74 (289.17 to 651.00)	11.94 (7.43 to 16.73)	-0.38 (-0.44 to -0.32)	<0.001
YLDs	Ecuador	1077.28 (672.44 to 1541.47)	21.73 (13.56 to 31.09)	852.58 (547.76 to 1213.63)	12.89 (8.28 to 18.34)	-1.65 (-1.81 to -1.55)	<0.001
YLDs	Egypt	5427.99 (3619.85 to 7396.49)	19.44 (12.96 to 26.49)	5048.19 (3137.38 to 7374.02)	10.84 (6.74 to 15.84)	-1.87 (-1.91 to -1.84)	<0.001
YLDs	El Salvador	3099.48 (1623.55 to 5939.77)	112.49 (58.92 to 215.57)	434.54 (273.35 to 595.13)	18.24 (11.48 to 24.98)	-5.75 (-5.84 to -5.65)	<0.001
YLDs	Equatorial Guinea	24.49 (16.03 to 34.31)	10.21 (6.68 to 14.30)	58.08 (34.68 to 82.82)	7.54 (4.50 to 10.75)	-0.97 (-1 to -0.94)	<0.001
YLDs	Eritrea	4240.47 (1838.07 to 8770.27)	215.60 (93.46 to 445.92)	303.35 (187.47 to 431.51)	9.43 (5.83 to 13.41)	-9.73 (-9.89 to -9.47)	<0.001
YLDs	Estonia	139.38 (95.81 to 194.19)	30.34 (20.86 to 42.28)	47.49 (31.03 to 66.68)	16.94 (11.07 to 23.79)	-1.84 (-1.87 to -1.81)	<0.001
YLDs	Eswatini	52.39 (33.57 to 72.10)	10.98 (7.04 to 15.11)	51.80 (34.03 to 73.31)	9.68 (6.36 to 13.70)	-0.44 (-0.51 to -0.37)	<0.001

YLDs	Ethiopia	8488.30 (5039.31 to 13915.24)	28.81 (17.10 to 47.22)	6084.59 (3873.77 to 8569.84)	10.64 (6.77 to 14.98)	-3.75 (-4.01 to -3.45)	<0.001
YLDs	Fiji	30.84 (19.04 to 44.09)	8.60 (5.31 to 12.29)	28.30 (17.67 to 41.91)	8.07 (5.04 to 11.95)	-0.17 (-0.23 to -0.11)	<0.001
YLDs	Finland	589.31 (397.30 to 830.83)	46.45 (31.31 to 65.48)	487.91 (333.39 to 681.89)	42.48 (29.03 to 59.37)	-0.28 (-0.34 to -0.23)	<0.001
YLDs	France	5929.45 (3966.22 to 8299.39)	36.73 (24.57 to 51.42)	4885.37 (3176.60 to 6803.77)	31.01 (20.17 to 43.19)	-0.54 (-0.57 to -0.52)	<0.001
YLDs	Gabon	52.55 (34.77 to 73.10)	10.34 (6.84 to 14.38)	66.60 (42.20 to 96.44)	8.05 (5.10 to 11.66)	-0.81 (-0.87 to -0.76)	<0.001
YLDs	Gambia	64.39 (40.16 to 95.11)	11.46 (7.15 to 16.93)	104.03 (66.40 to 149.75)	8.21 (5.24 to 11.82)	-1.09 (-1.13 to -1.05)	<0.001
YLDs	Georgia	469.15 (316.61 to 633.64)	26.11 (17.62 to 35.26)	184.96 (118.72 to 263.58)	19.90 (12.78 to 28.36)	-0.9 (-1.08 to -0.7)	<0.001
YLDs	Germany	5869.16 (3859.91 to 8153.58)	33.87 (22.27 to 47.05)	4780.28 (3137.32 to 6810.20)	29.98 (19.67 to 42.71)	-0.38 (-0.42 to -0.35)	<0.001
YLDs	Ghana	719.70 (469.25 to 1002.59)	8.71 (5.68 to 12.14)	1357.87 (866.38 to 1944.88)	8.32 (5.31 to 11.92)	-0.14 (-0.2 to -0.1)	<0.001

YLDs	Greece	1077.17 (728.68 to 1457.18)	38.33 (25.93 to 51.85)	543.88 (344.47 to 780.37)	28.55 (18.08 to 40.96)	-0.96 (-0.99 to -0.93)	<0.001
YLDs	Greenland	4.32 (2.93 to 5.91)	24.16 (16.35 to 33.02)	2.10 (1.36 to 2.91)	13.63 (8.82 to 18.93)	-1.82 (-1.85 to -1.79)	<0.001
YLDs	Grenada	8.73 (5.30 to 14.36)	20.70 (12.56 to 34.05)	3.80 (2.50 to 5.39)	12.62 (8.31 to 17.90)	-1.58 (-1.66 to -1.47)	<0.001
YLDs	Guam	4.05 (2.53 to 5.89)	7.49 (4.68 to 10.88)	3.28 (2.00 to 4.94)	6.76 (4.13 to 10.20)	-0.35 (-0.38 to -0.33)	<0.001
YLDs	Guatemala	2271.05 (1327.40 to 3992.79)	46.25 (27.03 to 81.31)	1361.39 (883.86 to 1851.46)	20.58 (13.36 to 27.99)	-2.61 (-2.67 to -2.54)	<0.001
YLDs	Guinea	357.55 (232.42 to 493.79)	10.88 (7.07 to 15.02)	814.21 (533.14 to 1149.86)	10.91 (7.14 to 15.40)	-0.14 (-0.26 to -0.02)	0.0219956
YLDs	Guinea-Bissau	69.89 (47.65 to 95.29)	11.88 (8.10 to 16.20)	104.61 (71.35 to 146.66)	9.35 (6.38 to 13.11)	-1.05 (-1.27 to -0.81)	<0.001
YLDs	Guyana	59.78 (37.98 to 83.91)	15.67 (9.95 to 21.99)	39.56 (25.43 to 55.69)	14.09 (9.06 to 19.84)	-0.34 (-0.4 to -0.3)	<0.001

YLDs	Haiti	580.58 (396.95 to 786.05)	17.35 (11.86 to 23.49)	4486.26 (2092.96 to 8783.62)	80.00 (37.32 to 156.63)	4.73 (3.96 to 5.64)	<0.001
YLDs	Honduras	607.04 (387.54 to 834.89)	22.37 (14.28 to 30.77)	820.52 (543.39 to 1158.82)	18.86 (12.49 to 26.64)	-0.5 (-0.77 to -0.2)	0.00119976
YLDs	Hungary	1023.52 (674.99 to 1408.93)	35.27 (23.26 to 48.55)	495.67 (322.16 to 714.17)	26.43 (17.18 to 38.08)	-0.98 (-1.02 to -0.94)	<0.001
YLDs	Iceland	28.70 (18.22 to 40.02)	33.96 (21.56 to 47.36)	26.42 (17.54 to 37.63)	29.65 (19.69 to 42.23)	-0.46 (-0.52 to -0.4)	<0.001
YLDs	India	62015.98 (41853.00 to 80675.38)	15.11 (10.20 to 19.66)	53185.42 (35474.97 to 69657.11)	10.62 (7.09 to 13.92)	-1.16 (-1.2 to -1.13)	<0.001
YLDs	Indonesia	15085.43 (10127.36 to 22281.55)	17.25 (11.58 to 25.48)	9970.93 (6933.73 to 13069.33)	11.05 (7.69 to 14.49)	-1.45 (-1.58 to -1.32)	<0.001
YLDs	Iran (Islamic Republic of)	10218.05 (7166.07 to 13973.15)	32.43 (22.74 to 44.35)	2990.43 (1953.81 to 3968.58)	11.57 (7.56 to 15.35)	-3.29 (-3.35 to -3.23)	<0.001
YLDs	Iraq	4675.76 (2673.96 to 7815.37)	45.40 (25.96 to 75.89)	5971.85 (3270.01 to 10341.45)	33.86 (18.54 to 58.64)	-0.91 (-1.21 to -0.62)	<0.001
YLDs	Ireland	487.83 (318.84 to 671.54)	36.71 (23.99 to 50.54)	417.04 (267.42 to 604.16)	31.56 (20.23 to 45.71)	-0.48 (-0.51 to -0.46)	<0.001

YLDs	Israel	698.04 (471.41 to 963.64)	34.79 (23.50 to 48.03)	940.52 (619.76 to 1339.53)	27.88 (18.37 to 39.71)	-0.81 (-0.89 to -0.75)	<0.001
YLDs	Italy	5660.74 (3857.65 to 7744.46)	41.56 (28.32 to 56.86)	3616.41 (2292.08 to 4954.29)	34.51 (21.87 to 47.28)	-0.6 (-0.62 to -0.58)	<0.001
YLDs	Jamaica	168.15 (110.35 to 227.53)	15.39 (10.10 to 20.83)	121.54 (78.92 to 176.01)	14.91 (9.68 to 21.59)	-0.13 (-0.18 to -0.09)	<0.001
YLDs	Japan	10109.59 (6827.09 to 13685.87)	30.30 (20.46 to 41.02)	4163.05 (2886.40 to 5570.48)	19.61 (13.59 to 26.23)	-1.41 (-1.43 to -1.39)	<0.001
YLDs	Jordan	284.21 (193.34 to 406.62)	13.56 (9.22 to 19.39)	514.67 (317.40 to 790.29)	10.45 (6.44 to 16.05)	-0.83 (-0.88 to -0.78)	<0.001
YLDs	Kazakhstan	1780.97 (1183.89 to 2452.14)	26.81 (17.82 to 36.91)	1099.64 (717.26 to 1601.61)	16.35 (10.67 to 23.82)	-1.57 (-1.64 to -1.53)	<0.001
YLDs	Kenya	1327.79 (913.70 to 1675.78)	9.63 (6.62 to 12.15)	2297.31 (1580.99 to 2999.39)	9.34 (6.43 to 12.19)	-0.13 (-0.16 to -0.1)	<0.001
YLDs	Kiribati	2.54 (1.58 to 3.58)	6.92 (4.32 to 9.78)	2.97 (1.84 to 4.11)	5.55 (3.44 to 7.69)	-0.79 (-0.93 to -0.64)	<0.001

YLDs	Kuwait	124.83 (81.64 to 186.05)	18.20 (11.90 to 27.12)	114.66 (72.18 to 168.16)	10.47 (6.59 to 15.35)	-2.06 (-2.32 to -1.83)	<0.001
YLDs	Kyrgyzstan	532.73 (355.65 to 727.15)	25.23 (16.84 to 34.44)	397.91 (247.96 to 559.61)	14.10 (8.78 to 19.82)	-1.87 (-1.95 to -1.81)	<0.001
YLDs	Lao People's Democratic Republic	270.91 (175.94 to 386.17)	11.94 (7.76 to 17.02)	223.58 (137.60 to 314.86)	7.47 (4.60 to 10.53)	-1.51 (-1.58 to -1.44)	<0.001
YLDs	Latvia	227.07 (155.81 to 309.13)	30.18 (20.71 to 41.09)	67.28 (43.92 to 97.99)	17.38 (11.35 to 25.31)	-1.76 (-1.82 to -1.71)	<0.001
YLDs	Lebanon	1568.10 (680.69 to 4046.45)	117.10 (50.83 to 302.17)	160.53 (105.07 to 232.80)	9.63 (6.31 to 13.97)	-7.78 (-7.98 to -7.47)	<0.001
YLDs	Lesotho	82.03 (53.49 to 114.34)	9.80 (6.39 to 13.66)	91.78 (55.85 to 124.57)	10.94 (6.66 to 14.85)	0.32 (0.25 to 0.37)	<0.001
YLDs	Liberia	309.46 (185.96 to 510.49)	22.65 (13.61 to 37.37)	224.58 (136.52 to 321.85)	8.05 (4.89 to 11.54)	-3.31 (-3.59 to -3.02)	<0.001
YLDs	Libya	499.61 (315.95 to 732.66)	21.64 (13.69 to 31.74)	1012.97 (637.09 to 1577.46)	48.52 (30.52 to 75.57)	2.92 (2.56 to 3.25)	<0.001
YLDs	Lithuania	302.22 (198.89 to 417.15)	27.29 (17.96 to 37.66)	94.21 (60.45 to 133.48)	17.58 (11.28 to 24.91)	-1.4 (-1.45 to -1.36)	<0.001

YLDs	Luxembourg	32.61 (21.46 to 44.21)	36.95 (24.33 to 50.10)	39.47 (25.97 to 56.30)	29.25 (19.25 to 41.73)	-0.75 (-0.78 to -0.71)	<0.001
YLDs	Madagascar	646.44 (412.97 to 888.92)	9.61 (6.14 to 13.22)	1226.71 (752.65 to 1755.59)	8.21 (5.04 to 11.75)	-0.5 (-0.54 to -0.48)	<0.001
YLDs	Malawi	512.90 (323.27 to 715.95)	9.20 (5.80 to 12.85)	906.65 (612.56 to 1278.85)	8.56 (5.78 to 12.08)	-0.27 (-0.32 to -0.24)	<0.001
YLDs	Malaysia	631.85 (412.23 to 882.86)	7.57 (4.94 to 10.58)	668.15 (423.13 to 960.37)	6.52 (4.13 to 9.36)	-0.49 (-0.54 to -0.44)	<0.001
YLDs	Maldives	13.00 (8.41 to 18.48)	10.13 (6.56 to 14.40)	11.95 (7.31 to 17.80)	9.17 (5.61 to 13.66)	-0.26 (-0.49 to -0.06)	0.01479704
YLDs	Mali	560.50 (365.33 to 764.38)	11.33 (7.39 to 15.46)	1929.23 (1257.11 to 2751.58)	13.55 (8.83 to 19.33)	0.59 (0.4 to 0.78)	<0.001
YLDs	Malta	42.09 (27.27 to 57.34)	36.52 (23.66 to 49.75)	26.38 (16.92 to 37.36)	31.42 (20.15 to 44.50)	-0.47 (-0.52 to -0.43)	<0.001
YLDs	Marshall Islands	1.90 (1.24 to 2.62)	7.08 (4.62 to 9.77)	1.57 (0.94 to 2.33)	6.79 (4.06 to 10.10)	-0.13 (-0.19 to -0.09)	<0.001

YLDs	Mauritania	117.49 (75.70 to 165.97)	10.36 (6.68 to 14.64)	192.43 (123.94 to 270.54)	8.25 (5.31 to 11.60)	-0.74 (-0.77 to -0.71)	<0.001
YLDs	Mauritius	37.32 (24.26 to 52.46)	8.66 (5.63 to 12.18)	21.50 (13.14 to 31.21)	7.24 (4.43 to 10.51)	-0.6 (-0.64 to -0.57)	<0.001
YLDs	Mexico	13743.30 (9567.43 to 17722.54)	31.69 (22.06 to 40.87)	7361.87 (4972.53 to 9735.34)	17.06 (11.52 to 22.56)	-1.95 (-2.12 to -1.8)	<0.001
YLDs	Micronesia (Federated States of)	5.07 (3.29 to 6.93)	8.82 (5.73 to 12.06)	3.94 (2.40 to 5.74)	9.50 (5.79 to 13.84)	0.27 (0.16 to 0.38)	<0.001
YLDs	Monaco	1.46 (0.93 to 2.07)	30.12 (19.13 to 42.73)	1.95 (1.31 to 2.75)	28.80 (19.38 to 40.65)	-0.14 (-0.17 to -0.11)	<0.001
YLDs	Mongolia	255.73 (166.06 to 351.26)	22.53 (14.63 to 30.95)	222.71 (145.11 to 313.38)	16.97 (11.05 to 23.87)	-0.92 (-0.99 to -0.86)	<0.001
YLDs	Montenegro	63.41 (41.85 to 88.01)	29.56 (19.51 to 41.03)	35.37 (22.98 to 50.70)	23.52 (15.28 to 33.71)	-0.74 (-0.78 to -0.71)	<0.001
YLDs	Morocco	2321.81 (1512.28 to 3203.35)	18.60 (12.12 to 25.67)	1418.41 (910.18 to 1993.48)	10.98 (7.05 to 15.43)	-1.71 (-1.75 to -1.67)	<0.001

YLDs	Mozambique	4731.23 (2225.40 to 9968.60)	62.49 (29.39 to 131.67)	1890.93 (1202.19 to 2681.82)	10.65 (6.77 to 15.11)	-5.53 (-5.61 to - 5.43)	<0.001
YLDs	Myanmar	4171.36 (2105.31 to 8817.38)	21.83 (11.02 to 46.14)	6495.01 (3728.13 to 10953.50)	31.27 (17.95 to 52.74)	1.16 (0.35 to 1.83)	0.009998
YLDs	Namibia	216.01 (111.61 to 394.71)	28.36 (14.65 to 51.82)	91.68 (59.06 to 127.93)	8.56 (5.51 to 11.94)	-3.78 (-3.86 to - 3.69)	<0.001
YLDs	Nauru	0.43 (0.28 to 0.60)	8.20 (5.29 to 11.52)	0.43 (0.27 to 0.62)	8.41 (5.27 to 12.14)	0.08 (0.04 to 0.12)	<0.001
YLDs	Nepal	1359.11 (863.00 to 1970.63)	13.13 (8.34 to 19.04)	2171.61 (1261.42 to 3372.89)	17.44 (10.13 to 27.08)	1.02 (0.67 to 1.25)	<0.001
YLDs	Netherlands	1058.49 (702.04 to 1465.84)	27.61 (18.32 to 38.24)	855.92 (558.01 to 1218.28)	23.19 (15.12 to 33.01)	-0.55 (-0.59 to - 0.51)	<0.001
YLDs	New Zealand	586.20 (395.49 to 798.41)	53.24 (35.92 to 72.51)	639.40 (418.60 to 879.05)	49.05 (32.11 to 67.44)	-0.27 (-0.32 to - 0.24)	<0.001
YLDs	Nicaragua	2414.93 (1093.89 to 4887.95)	107.32 (48.61 to 217.22)	461.36 (296.05 to 641.20)	17.75 (11.39 to 24.67)	-5.61 (-5.71 to - 5.43)	<0.001

YLDs	Niger	539.80 (335.63 to 767.19)	11.13 (6.92 to 15.81)	1846.72 (1192.37 to 2682.07)	11.85 (7.65 to 17.21)	0.21 (0.16 to 0.27)	<0.001
YLDs	Nigeria	5224.94 (3607.03 to 6703.27)	10.77 (7.43 to 13.82)	13583.22 (9378.14 to 18131.56)	10.63 (7.34 to 14.19)	-0.04 (-0.08 to 0)	0.0579884
YLDs	Niue	0.08 (0.05 to 0.12)	7.76 (4.85 to 11.34)	0.05 (0.03 to 0.08)	10.18 (6.25 to 14.93)	0.76 (0.64 to 0.88)	<0.001
YLDs	North Macedonia	200.90 (141.42 to 276.69)	28.83 (20.29 to 39.70)	105.44 (67.94 to 143.26)	23.61 (15.21 to 32.08)	-0.66 (-0.7 to -0.63)	<0.001
YLDs	Northern Mariana Islands	1.36 (0.83 to 1.97)	8.39 (5.11 to 12.13)	1.38 (0.88 to 2.00)	9.23 (5.84 to 13.35)	0.34 (0.24 to 0.43)	<0.001
YLDs	Norway	379.77 (254.99 to 513.41)	34.14 (22.92 to 46.15)	327.38 (218.46 to 450.57)	26.37 (17.60 to 36.29)	-0.84 (-0.87 to -0.81)	<0.001
YLDs	Oman	192.51 (130.89 to 259.97)	19.21 (13.06 to 25.94)	173.50 (104.80 to 258.71)	11.63 (7.02 to 17.34)	-1.62 (-1.67 to -1.57)	<0.001
YLDs	Pakistan	5500.34 (3717.68 to 7287.24)	9.02 (6.10 to 11.95)	12640.87 (8561.22 to 18113.46)	11.57 (7.84 to 16.58)	0.71 (0.48 to 0.88)	<0.001
YLDs	Palau	0.67 (0.41 to 0.97)	10.88 (6.68 to 15.78)	0.43 (0.27 to 0.65)	9.71 (6.02 to 14.75)	-0.41 (-0.44 to -0.38)	<0.001

YLDs	Palestine	1038.48 (480.64 to 2041.74)	87.01 (40.27 to 171.06)	492.69 (293.18 to 800.26)	20.46 (12.18 to 33.24)	-4.57 (-4.75 to -4.4)	<0.001
YLDs	Panama	321.04 (190.06 to 620.09)	29.53 (17.48 to 57.04)	251.09 (162.28 to 368.06)	16.66 (10.77 to 24.42)	-1.79 (-1.85 to -1.72)	<0.001
YLDs	Papua New Guinea	198.53 (128.56 to 276.86)	9.33 (6.04 to 13.01)	478.12 (305.65 to 697.72)	9.76 (6.24 to 14.24)	0.03 (-0.27 to 0.35)	0.84663067
YLDs	Paraguay	363.45 (231.90 to 521.15)	17.57 (11.21 to 25.19)	360.82 (231.30 to 523.39)	13.51 (8.66 to 19.60)	-0.86 (-0.88 to -0.83)	<0.001
YLDs	Peru	2998.84 (1858.73 to 4738.36)	28.16 (17.45 to 44.49)	1369.59 (859.76 to 1989.36)	10.95 (6.87 to 15.90)	-3.02 (-3.09 to -2.94)	<0.001
YLDs	Philippines	5937.57 (3730.02 to 9943.52)	18.52 (11.64 to 31.02)	5113.96 (3386.72 to 7067.26)	11.42 (7.56 to 15.78)	-1.69 (-1.82 to -1.57)	<0.001
YLDs	Poland	3622.22 (2459.64 to 4748.32)	29.10 (19.76 to 38.14)	1372.54 (903.13 to 1839.17)	17.83 (11.73 to 23.89)	-1.59 (-1.62 to -1.57)	<0.001
YLDs	Portugal	1375.50 (926.07 to 1841.03)	46.14 (31.07 to 61.76)	432.83 (297.91 to 623.48)	22.58 (15.54 to 32.53)	-2.3 (-2.35 to -2.26)	<0.001
YLDs	Puerto Rico	210.46 (139.10 to 285.33)	15.80 (10.44 to 21.42)	110.66 (68.23 to 176.31)	16.93 (10.44 to 26.98)	0.2 (0.11 to 0.26)	<0.001

YLDs	Qatar	20.09 (12.95 to 28.10)	13.12 (8.46 to 18.36)	53.07 (32.95 to 78.17)	8.89 (5.52 to 13.09)	-1.28 (-1.34 to -1.24)	<0.001
YLDs	Republic of Korea	7355.69 (4999.30 to 10057.93)	46.18 (31.39 to 63.15)	1948.32 (1239.07 to 2714.11)	23.24 (14.78 to 32.38)	-2.19 (-2.23 to -2.16)	<0.001
YLDs	Republic of Moldova	514.41 (350.33 to 688.20)	32.53 (22.15 to 43.52)	137.32 (88.66 to 189.01)	19.72 (12.73 to 27.15)	-1.6 (-1.64 to -1.57)	<0.001
YLDs	Romania	3282.60 (2230.98 to 4406.38)	43.75 (29.74 to 58.73)	1060.64 (696.95 to 1495.23)	26.25 (17.25 to 37.00)	-1.65 (-1.68 to -1.62)	<0.001
YLDs	Russian Federation	14417.98 (10011.07 to 19155.37)	31.91 (22.16 to 42.39)	5637.57 (3851.65 to 7672.63)	16.68 (11.40 to 22.71)	-2.05 (-2.12 to -2.01)	<0.001
YLDs	Rwanda	565.12 (369.75 to 828.65)	13.65 (8.93 to 20.02)	598.47 (370.54 to 866.38)	9.30 (5.76 to 13.46)	-1.27 (-2.59 to 0.06)	0.05718856
YLDs	Saint Kitts and Nevis	2.59 (1.71 to 3.66)	14.10 (9.29 to 19.92)	2.04 (1.33 to 2.91)	14.49 (9.43 to 20.73)	0.07 (-0.01 to 0.13)	0.07038592
YLDs	Saint Lucia	8.41 (5.55 to 11.63)	12.59 (8.32 to 17.41)	5.34 (3.47 to 7.47)	12.72 (8.28 to 17.81)	0.03 (-0.02 to 0.07)	0.20475905

YLDs	Saint Vincent and the Grenadines	7.33 (5.03 to 9.96)	13.63 (9.36 to 18.54)	4.38 (2.74 to 6.40)	13.01 (8.14 to 18.99)	-0.16 (-0.2 to -0.11)	<0.001
YLDs	Samoa	7.29 (4.44 to 10.88)	7.95 (4.84 to 11.88)	14.09 (8.04 to 23.39)	13.96 (7.96 to 23.17)	1.5 (1.16 to 1.87)	<0.001
YLDs	San Marino	2.09 (1.38 to 2.98)	34.86 (23.08 to 49.84)	1.93 (1.25 to 2.76)	30.96 (19.97 to 44.17)	-0.38 (-0.41 to -0.35)	<0.001
YLDs	Sao Tome and Principe	10.09 (6.41 to 13.93)	14.44 (9.18 to 19.93)	11.42 (7.42 to 16.13)	11.21 (7.29 to 15.85)	-0.82 (-0.86 to -0.78)	<0.001
YLDs	Saudi Arabia	1721.52 (1173.50 to 2352.91)	20.96 (14.29 to 28.65)	1423.10 (937.01 to 2024.86)	14.04 (9.25 to 19.98)	-1.29 (-1.36 to -1.24)	<0.001
YLDs	Senegal	405.35 (247.22 to 566.88)	9.12 (5.56 to 12.75)	666.21 (416.82 to 957.62)	8.21 (5.14 to 11.80)	-0.33 (-0.38 to -0.28)	<0.001
YLDs	Serbia	921.38 (622.92 to 1244.50)	31.76 (21.47 to 42.90)	465.10 (304.25 to 659.01)	24.82 (16.24 to 35.17)	-0.86 (-1.14 to -0.57)	<0.001
YLDs	Seychelles	2.54 (1.59 to 3.57)	8.13 (5.07 to 11.44)	1.89 (1.11 to 2.77)	6.19 (3.62 to 9.07)	-0.89 (-0.99 to -0.81)	<0.001
YLDs	Sierra Leone	247.34 (161.91 to 344.62)	11.21 (7.34 to 15.62)	480.26 (306.66 to 695.23)	10.55 (6.74 to 15.28)	-0.22 (-0.42 to -0.02)	0.0339932

YLDs	Singapore	270.45 (185.42 to 366.43)	29.37 (20.13 to 39.79)	219.70 (143.38 to 318.56)	21.06 (13.74 to 30.53)	-1.1 (-1.15 to -1.06)	<0.001
YLDs	Slovakia	541.50 (350.23 to 752.13)	30.85 (19.95 to 42.85)	251.04 (176.13 to 352.09)	22.39 (15.71 to 31.41)	-1.02 (-1.05 to -1)	<0.001
YLDs	Slovenia	219.66 (147.54 to 292.35)	39.45 (26.50 to 52.51)	121.43 (78.17 to 170.59)	30.00 (19.31 to 42.14)	-0.88 (-0.91 to -0.85)	<0.001
YLDs	Solomon Islands	21.90 (13.35 to 33.82)	11.25 (6.86 to 17.38)	35.77 (20.82 to 55.40)	10.81 (6.29 to 16.75)	-0.21 (-0.33 to -0.07)	0.0079984
YLDs	Somalia	1253.66 (453.86 to 4189.62)	26.49 (9.59 to 88.53)	2841.91 (1720.70 to 4715.21)	22.23 (13.46 to 36.88)	-1.36 (-1.66 to -1.02)	<0.001
YLDs	South Africa	3196.44 (2069.36 to 5078.94)	18.14 (11.74 to 28.82)	1820.87 (1226.24 to 2361.99)	9.14 (6.16 to 11.86)	-2.2 (-2.23 to -2.16)	<0.001
YLDs	South Sudan	730.12 (411.97 to 1225.48)	22.22 (12.54 to 37.29)	1161.54 (700.89 to 2007.40)	21.17 (12.77 to 36.58)	0.22 (-0.1 to 0.57)	0.16196761
YLDs	Spain	3594.90 (2378.37 to 4886.40)	32.25 (21.34 to 43.84)	2478.36 (1552.45 to 3512.64)	28.14 (17.63 to 39.88)	-0.42 (-0.48 to -0.36)	<0.001
YLDs	Sri Lanka	2327.98 (1179.01 to 4238.49)	32.15 (16.28 to 58.54)	1435.18 (868.89 to 2320.57)	20.80 (12.59 to 33.63)	-1.45 (-1.82 to -1.13)	<0.001

YLDs	Sudan	2813.92 (1743.66 to 4245.40)	25.47 (15.79 to 38.43)	3199.69 (2026.72 to 4781.97)	14.97 (9.48 to 22.37)	-1.76 (-1.92 to -1.62)	<0.001
YLDs	Suriname	47.69 (22.89 to 119.40)	27.89 (13.38 to 69.83)	23.37 (14.59 to 33.88)	12.31 (7.69 to 17.85)	-2.61 (-2.68 to -2.52)	<0.001
YLDs	Sweden	701.84 (465.13 to 980.38)	33.31 (22.08 to 46.53)	615.60 (404.81 to 893.07)	25.49 (16.76 to 36.98)	-0.85 (-0.89 to -0.8)	<0.001
YLDs	Switzerland	648.60 (431.84 to 898.21)	41.12 (27.38 to 56.95)	595.89 (391.33 to 841.54)	33.81 (22.20 to 47.74)	-0.61 (-0.66 to -0.56)	<0.001
YLDs	Syrian Arab Republic	1330.05 (789.38 to 2241.10)	18.09 (10.74 to 30.48)	7455.32 (3202.49 to 15094.27)	137.04 (58.87 to 277.46)	6.84 (5.48 to 7.71)	<0.001
YLDs	Taiwan (Province of China)	771.28 (478.32 to 1101.24)	10.55 (6.54 to 15.07)	260.61 (165.36 to 384.88)	6.44 (4.09 to 9.51)	-1.65 (-1.77 to -1.56)	<0.001
YLDs	Tajikistan	581.40 (375.55 to 791.56)	20.23 (13.06 to 27.54)	610.65 (396.14 to 874.72)	13.71 (8.89 to 19.63)	-0.67 (-1.27 to -0.05)	0.03839232
YLDs	Thailand	2709.98 (1912.52 to 3773.60)	11.91 (8.41 to 16.59)	1201.43 (724.33 to 1748.86)	8.80 (5.31 to 12.81)	-1.01 (-1.07 to -0.96)	<0.001

YLDs	Timor-Leste	272.43 (126.58 to 548.11)	66.99 (31.13 to 134.78)	45.31 (26.56 to 65.70)	6.61 (3.87 to 9.59)	-7.53 (-7.97 to -7.09)	<0.001
YLDs	Togo	222.92 (141.33 to 314.12)	10.36 (6.57 to 14.59)	374.33 (238.94 to 538.30)	8.97 (5.72 to 12.89)	-0.48 (-0.56 to -0.39)	<0.001
YLDs	Tokelau	0.06 (0.03 to 0.08)	7.31 (4.57 to 10.61)	0.03 (0.02 to 0.05)	6.38 (4.07 to 9.25)	-0.49 (-0.55 to -0.43)	<0.001
YLDs	Tonga	3.91 (2.32 to 5.60)	7.33 (4.34 to 10.49)	3.03 (1.78 to 4.71)	6.12 (3.58 to 9.50)	-0.71 (-0.81 to -0.63)	<0.001
YLDs	Trinidad and Tobago	71.13 (46.44 to 100.73)	13.69 (8.94 to 19.39)	56.30 (35.57 to 79.04)	15.47 (9.77 to 21.71)	0.57 (0.45 to 0.7)	<0.001
YLDs	Tunisia	730.89 (473.79 to 1050.00)	18.32 (11.87 to 26.32)	382.51 (239.74 to 561.96)	10.67 (6.69 to 15.67)	-1.71 (-1.75 to -1.68)	<0.001
YLDs	Turkey	4204.82 (2811.45 to 5951.29)	15.76 (10.54 to 22.30)	2623.51 (1596.32 to 3782.34)	10.62 (6.46 to 15.31)	-1.19 (-1.27 to -1.09)	<0.001
YLDs	Turkmenistan	424.44 (283.57 to 574.20)	22.51 (15.04 to 30.46)	281.74 (184.66 to 391.82)	14.37 (9.42 to 19.99)	-1.44 (-1.49 to -1.38)	<0.001

YLDs	Tuvalu	0.35 (0.23 to 0.48)	8.13 (5.35 to 11.34)	0.32 (0.20 to 0.47)	6.47 (4.04 to 9.54)	-0.77 (-0.83 to -0.72)	<0.001
YLDs	Uganda	3748.52 (1746.00 to 7457.88)	36.40 (16.96 to 72.42)	2326.76 (1528.56 to 3314.82)	9.33 (6.13 to 13.29)	-4.32 (-4.4 to -4.22)	<0.001
YLDs	Ukraine	4477.64 (2921.29 to 6260.44)	29.75 (19.41 to 41.59)	1736.88 (1124.26 to 2451.30)	20.57 (13.31 to 29.03)	-1.19 (-1.27 to -1.11)	<0.001
YLDs	United Arab Emirates	100.68 (68.25 to 137.61)	14.24 (9.65 to 19.46)	164.31 (104.98 to 234.77)	9.69 (6.19 to 13.85)	-1.2 (-1.29 to -1.16)	<0.001
YLDs	United Kingdom	4458.67 (2990.52 to 6039.63)	30.06 (20.16 to 40.71)	3526.03 (2403.25 to 4781.96)	22.52 (15.35 to 30.55)	-0.91 (-0.96 to -0.88)	<0.001
YLDs	United Republic of Tanzania	1542.21 (1004.78 to 2188.61)	10.33 (6.73 to 14.67)	2992.29 (1896.34 to 4375.44)	9.70 (6.15 to 14.18)	-0.2 (-0.24 to -0.17)	<0.001
YLDs	United States of America	18271.02 (12580.25 to 24676.70)	24.68 (16.99 to 33.33)	11064.32 (7646.44 to 14922.06)	13.61 (9.40 to 18.35)	-1.94 (-1.99 to -1.89)	<0.001
YLDs	United States Virgin Islands	5.88 (3.85 to 8.47)	14.10 (9.22 to 20.31)	2.16 (1.40 to 3.10)	11.84 (7.69 to 16.99)	-0.61 (-0.66 to -0.57)	<0.001

YLDs	Uruguay	334.24 (226.05 to 458.62)	31.03 (20.99 to 42.58)	231.66 (156.85 to 315.90)	25.65 (17.36 to 34.97)	-0.63 (-0.66 to -0.59)	<0.001
YLDs	Uzbekistan	2248.82 (1478.76 to 3109.75)	21.06 (13.85 to 29.13)	1789.13 (1128.91 to 2483.01)	14.19 (8.95 to 19.69)	-1.28 (-1.35 to -1.22)	<0.001
YLDs	Vanuatu	9.41 (5.07 to 15.43)	11.28 (6.07 to 18.48)	10.48 (6.31 to 14.90)	7.11 (4.28 to 10.11)		
YLDs	Venezuela (Bolivarian Republic of)	2476.32 (1677.03 to 3311.64)	27.28 (18.48 to 36.49)	1808.70 (1190.41 to 2508.64)	20.67 (13.60 to 28.66)	-0.99 (-1.14 to -0.88)	<0.001
YLDs	Viet Nam	3084.79 (1931.07 to 4377.08)	9.16 (5.74 to 13.00)	2369.41 (1438.82 to 3510.31)	7.49 (4.55 to 11.10)	-0.65 (-0.73 to -0.59)	<0.001
YLDs	Yemen	1941.04 (1256.08 to 2899.48)	23.09 (14.94 to 34.48)	10123.64 (5139.74 to 18198.16)	57.94 (29.42 to 104.16)	3.04 (2.63 to 3.28)	<0.001
YLDs	Zambia	492.14 (315.94 to 681.56)	10.47 (6.72 to 14.50)	884.87 (573.19 to 1259.99)	8.46 (5.48 to 12.05)	-0.7 (-0.74 to -0.66)	<0.001
YLDs	Zimbabwe	533.64 (350.20 to 738.99)	8.86 (5.81 to 12.27)	654.58 (441.33 to 931.06)	8.20 (5.53 to 11.66)	-0.23 (-0.31 to -0.18)	<0.001

YLDs years lived with disability, *AAPC* average annual percentage change.

.

Supplementary Table 17 Joinpoint regression analysis of incidence of spinal cord injury from 1990 to 2021 among children and adolescents at national level

Measure	Location	Year	APC (95% CI)	*P*
Incidence	Afghanistan	1990-1998	9.3 (-0.77 to 20.45)	0.056
Incidence	Afghanistan	1998-2003	-27.86 (-45.93 to 13.56)	0.058
Incidence	Afghanistan	2003-2007	30.62 (-23.26 to 55.95)	0.064
Incidence	Afghanistan	2007-2021	8.59 (-7.41 to 12.77)	0.089
Incidence	Albania	1990-2021	-0.47 (-1.06 to 0.14)	0.118
Incidence	Algeria	1990-1997	8.41 (4.53 to 13.12)	0.010
Incidence	Algeria	1997-2005	-6.69 (-14.14 to -3.7)	0.025
Incidence	Algeria	2005-2021	-2.85 (-3.68 to 0.29)	0.058
Incidence	American Samoa	1990-2021	-0.07 (-0.74 to 0.63)	0.734
Incidence	Andorra	1990-2002	-0.42 (-0.48 to -0.38)	<0.001
Incidence	Andorra	2002-2010	0.19 (0.11 to 0.3)	0.003
Incidence	Andorra	2010-2018	-1.66 (-1.74 to -1.59)	<0.001
Incidence	Andorra	2018-2021	0.25 (-0.06 to 0.52)	0.077
Incidence	Angola	1990-1993	40.53 (-10.99 to 140.26)	0.062
Incidence	Angola	1993-1996	-41.29 (-52.57 to 60.58)	0.058
Incidence	Angola	1996-1999	109.66 (-38.5 to 155.97)	0.058
Incidence	Angola	1999-2003	-42.19 (-56.24 to -4.95)	0.023
Incidence	Angola	2003-2021	-1.65 (-2.9 to -0.33)	0.026

Incidence	Antigua and Barbuda	1990-1997	3.93 (-0.1 to 15.23)	0.062
Incidence	Antigua and Barbuda	1997-2021	-0.71 (-3.66 to 0.24)	0.063
Incidence	Argentina	1990-2000	0.18 (0.01 to 0.33)	0.043
Incidence	Argentina	2000-2005	2.35 (1.83 to 3.18)	0.000
Incidence	Argentina	2005-2012	-0.49 (-0.84 to -0.13)	0.023
Incidence	Argentina	2012-2019	-2.74 (-3.2 to -2.53)	<0.001
Incidence	Argentina	2019-2021	0.72 (-0.78 to 1.8)	0.247
Incidence	Armenia	1990-2021	-1.46 (-2.26 to -0.64)	0.002
Incidence	Australia	1990-1995	-0.85 (-0.98 to -0.75)	<0.001
Incidence	Australia	1995-2008	-0.07 (-0.1 to -0.03)	0.009
Incidence	Australia	2008-2011	-0.48 (-0.63 to -0.28)	0.001
Incidence	Australia	2011-2014	-2.49 (-2.64 to -2.29)	<0.001
Incidence	Australia	2014-2018	-1.71 (-1.84 to -1.46)	<0.001
Incidence	Australia	2018-2021	-0.15 (-0.36 to 0.06)	0.102
Incidence	Austria	1990-1993	-2.55 (-3.14 to -2.24)	<0.001
Incidence	Austria	1993-1996	-1.1 (-1.83 to -0.12)	0.021
Incidence	Austria	1996-1999	0.21 (-0.38 to 0.43)	0.268
Incidence	Austria	1999-2010	-0.38 (-1.18 to -0.34)	0.001
Incidence	Austria	2010-2018	-2.29 (-2.38 to -2.2)	<0.001

Incidence	Austria	2018-2021	-0.13 (-0.49 to 0.31)	0.355
Incidence	Azerbaijan	1990-2021	-1.2 (-1.96 to -0.46)	0.002
Incidence	Bahamas	1990-2021	0.17 (-0.33 to 0.66)	0.461
Incidence	Bahrain	1990-1996	3.35 (0.78 to 10.81)	0.011
Incidence	Bahrain	1996-2021	-0.83 (-1.45 to -0.52)	0.003
Incidence	Bangladesh	1990-2021	-0.18 (-0.96 to 0.63)	0.590
Incidence	Barbados	1990-1992	26.1 (10.35 to 39.17)	0.002
Incidence	Barbados	1992-1995	-9.27 (-12.41 to -1.9)	0.000
Incidence	Barbados	1995-2021	-0.25 (-0.57 to 0.36)	0.285
Incidence	Belarus	1990-2002	0.71 (0.67 to 0.74)	<0.001
Incidence	Belarus	2002-2007	-0.59 (-0.68 to -0.46)	0.001
Incidence	Belarus	2007-2010	-2 (-2.28 to -1.75)	<0.001
Incidence	Belarus	2010-2016	-3.49 (-3.62 to -3.41)	<0.001
Incidence	Belarus	2016-2019	-2.16 (-2.43 to -1.99)	<0.001
Incidence	Belarus	2019-2021	1.59 (1.22 to 1.94)	<0.001
Incidence	Belgium	1990-1996	-0.56 (-1.11 to -0.2)	0.008
Incidence	Belgium	1996-2005	2.46 (2.22 to 2.76)	<0.001
Incidence	Belgium	2005-2011	-0.56 (-0.98 to -0.16)	0.009
Incidence	Belgium	2011-2018	-6.08 (-6.4 to -5.82)	<0.001

Incidence	Belgium	2018-2021	-0.31 (-1.39 to 0.73)	0.419
Incidence	Belize	1990-2021	-0.36 (-1.05 to 0.33)	0.296
Incidence	Benin	1990-2003	-0.34 (-0.5 to -0.25)	0.004
Incidence	Benin	2003-2010	0.19 (-0.06 to 0.9)	0.110
Incidence	Benin	2010-2014	-1.79 (-2.43 to -1.24)	0.018
Incidence	Benin	2014-2019	-0.39 (-0.82 to 0.09)	0.085
Incidence	Benin	2019-2021	3 (1.72 to 3.94)	<0.001
Incidence	Bermuda	1990-2003	1.3 (0.74 to 2.32)	<0.001
Incidence	Bermuda	2003-2021	-0.92 (-1.54 to -0.54)	<0.001
Incidence	Bhutan	1990-2021	0.14 (-0.48 to 0.76)	0.624
Incidence	Bolivia (Plurinational State of)	1990-2021	-0.89 (-1.03 to -0.76)	<0.001
Incidence	Bosnia and Herzegovina	1990-1993	135.12 (67.17 to 348.95)	0.004
Incidence	Bosnia and Herzegovina	1993-1996	-56.28 (-65.9 to -31.44)	0.003
Incidence	Bosnia and Herzegovina	1996-2021	-0.65 (-1.9 to 0.78)	0.332
Incidence	Botswana	1990-2006	1.37 (1.08 to 1.71)	<0.001
Incidence	Botswana	2006-2021	-0.59 (-0.99 to -0.29)	0.000
Incidence	Brazil	1990-1996	1.84 (1.1 to 2.91)	0.002
Incidence	Brazil	1996-2007	-0.35 (-1.53 to -0.09)	0.017
Incidence	Brazil	2007-2010	2.88 (0.93 to 3.85)	0.003

Incidence	Brazil	2010-2013	-2.89 (-3.88 to -1.12)	0.002
Incidence	Brazil	2013-2019	1.58 (0.97 to 3.07)	0.000
Incidence	Brazil	2019-2021	-20.43 (-22.51 to -18.96)	<0.001
Incidence	Brunei Darussalam	1990-2010	-1 (-1.05 to -0.94)	<0.001
Incidence	Brunei Darussalam	2010-2014	-2.28 (-2.87 to -1.72)	<0.001
Incidence	Brunei Darussalam	2014-2021	-0.56 (-0.78 to -0.22)	0.007
Incidence	Bulgaria	1990-2010	-0.79 (-0.84 to -0.74)	0.001
Incidence	Bulgaria	2010-2018	-1.6 (-2.01 to -1.42)	<0.001
Incidence	Bulgaria	2018-2021	0.17 (-0.58 to 1.2)	0.591
Incidence	Burkina Faso	1990-2016	-0.12 (-0.4 to 0.16)	0.340
Incidence	Burkina Faso	2016-2021	14.22 (9.87 to 20)	<0.001
Incidence	Burundi	1990-1993	3.32 (-27.09 to 26.85)	0.777
Incidence	Burundi	1993-1996	225.16 (89.64 to 344.26)	0.005
Incidence	Burundi	1996-2003	-3.05 (-50.82 to 32.94)	0.795
Incidence	Burundi	2003-2006	-67.34 (-75.8 to 7.4)	0.094
Incidence	Burundi	2006-2021	-0.43 (-5.96 to 4.79)	0.803
Incidence	Cabo Verde	1990-1997	-0.93 (-1.83 to -0.54)	0.000
Incidence	Cabo Verde	1997-2009	0.1 (-0.11 to 1.35)	0.312
Incidence	Cabo Verde	2009-2021	-0.6 (-1.24 to -0.38)	0.002

Incidence	Cambodia	1990-1996	10.91 (5.73 to 20.48)	<0.001
Incidence	Cambodia	1996-1999	-28.8 (-32.96 to -17.52)	<0.001
Incidence	Cambodia	1999-2021	-0.54 (-1.04 to -0.01)	0.047
Incidence	Cameroon	1990-2012	0.19 (-0.37 to 0.77)	0.432
Incidence	Cameroon	2012-2019	9.25 (-0.17 to 22)	0.054
Incidence	Cameroon	2019-2021	-11.61 (-21.41 to 5.88)	0.167
Incidence	Canada	1990-1994	-2.23 (-2.67 to -1.94)	<0.001
Incidence	Canada	1994-2004	-1.24 (-1.34 to -1.14)	<0.001
Incidence	Canada	2004-2008	-0.44 (-0.72 to -0.11)	0.005
Incidence	Canada	2008-2017	-2.42 (-2.51 to -2.35)	<0.001
Incidence	Canada	2017-2021	-0.45 (-0.72 to -0.22)	0.024
Incidence	Central African Republic	1990-2021	2.96 (1.75 to 4.25)	<0.001
Incidence	Chad	1990-2021	-0.89 (-2.14 to 0.44)	0.176
Incidence	Chile	1990-1995	0.42 (-1.27 to 1.16)	0.354
Incidence	Chile	1995-2001	2.99 (2.3 to 4.83)	0.002
Incidence	Chile	2001-2010	0.87 (0.17 to 1.35)	0.029
Incidence	Chile	2010-2021	-1.8 (-2.18 to -1.5)	<0.001
Incidence	China	1990-2011	-2.52 (-3.2 to -2.07)	<0.001
Incidence	China	2011-2021	1.21 (-0.28 to 4.35)	0.100

Incidence	Colombia	1990-2002	1.12<br>(-2.93 to 3.6)	0.154
Incidence	Colombia	2002-2007	-7.85<br>(-13.86 to 4.66)	0.063
Incidence	Colombia	2007-2021	-1.99<br>(-3.26 to 2.55)	0.170
Incidence	Comoros	1990-2021	-0.04<br>(-0.6 to 0.53)	0.884
Incidence	Congo	1990-2021	-1.09<br>(-2.3 to 0.08)	0.066
Incidence	Cook Islands	1990-2021	-0.59<br>(-1.28 to 0.13)	0.091
Incidence	Costa Rica	1990-2010	-0.03<br>(-0.14 to 0.09)	0.611
Incidence	Costa Rica	2010-2015	-2.16<br>(-3.47 to -1.32)	0.024
Incidence	Costa Rica	2015-2021	-0.45<br>(-1.03 to 0.85)	0.278
Incidence	Coted'Ivoire	1990-2021	-0.11<br>(-0.57 to 0.35)	0.621
Incidence	Croatia	1990-1992	79.51<br>(27.58 to 124.13)	0.004
Incidence	Croatia	1992-1995	-30.62<br>(-39.71 to -12.92)	0.003
Incidence	Croatia	1995-2021	-1.21<br>(-1.88 to -0.39)	0.011
Incidence	Cuba	1990-1996	-0.47<br>(-1.28 to -0.13)	0.030
Incidence	Cuba	1996-2005	0.04<br>(-0.32 to 0.86)	0.648
Incidence	Cuba	2005-2010	0.81<br>(-2.29 to 1.58)	0.174
Incidence	Cuba	2010-2016	-2.08<br>(-2.89 to -0.76)	0.032
Incidence	Cuba	2016-2021	-0.18<br>(-0.78 to 0.89)	0.400
Incidence	Cyprus	1990-2006	0.54<br>(0.5 to 0.6)	0.001

Incidence	Cyprus	2006-2010	-0.06 (-1.36 to 0.24)	0.632
Incidence	Cyprus	2010-2018	-2.85 (-3.03 to -2.72)	<0.001
Incidence	Cyprus	2018-2021	-0.53 (-1.12 to 0.03)	0.056
Incidence	Czechia	1990-2000	-0.3 (-0.37 to -0.23)	<0.001
Incidence	Czechia	2000-2005	1.85 (1.7 to 2)	<0.001
Incidence	Czechia	2005-2010	-0.76 (-0.97 to -0.56)	0.000
Incidence	Czechia	2010-2015	-2.19 (-2.41 to -1.97)	<0.001
Incidence	Czechia	2015-2019	-4.33 (-4.57 to -4.1)	<0.001
Incidence	Czechia	2019-2021	0.56 (-0.03 to 1.05)	0.059
Incidence	Democratic People's Republic of Korea	1990-2021	-1 (-1.3 to -0.68)	<0.001
Incidence	Democratic Republic of the Congo	1990-2021	0.22 (-0.94 to 1.41)	0.701
Incidence	Denmark	1990-1997	-0.5 (-0.63 to -0.42)	0.000
Incidence	Denmark	1997-2001	0.03 (-0.26 to 0.46)	0.733
Incidence	Denmark	2001-2008	0.81 (0.73 to 0.95)	0.005
Incidence	Denmark	2008-2011	-0.84 (-1.1 to -0.64)	0.003
Incidence	Denmark	2011-2018	-2.48 (-2.57 to -2.4)	<0.001
Incidence	Denmark	2018-2021	0.08 (-0.14 to 0.29)	0.433
Incidence	Djibouti	1990-1995	-20.85 (-50.7 to -3.29)	0.003
Incidence	Djibouti	1995-2021	-0.84 (-1.69 to 0.63)	0.158

Incidence	Dominica	1990-2021	0.56<br>(0 to 1.15)	0.048
Incidence	Dominican Republic	1990-2004	1.27<br>(0.53 to 2.99)	<0.001
Incidence	Dominican Republic	2004-2021	-0.94<br>(-2.1 to -0.38)	0.001
Incidence	Ecuador	1990-2008	0.57<br>(0.09 to 1.55)	0.022
Incidence	Ecuador	2008-2021	-1.66<br>(-3.71 to -0.9)	<0.001
Incidence	Egypt	1990-2021	-1.07<br>(-1.34 to -0.79)	<0.001
Incidence	El Salvador	1990-1992	-46.87<br>(-56.94 to -28.25)	<0.001
Incidence	El Salvador	1992-2021	-0.36<br>(-0.78 to 0.09)	0.118
Incidence	Equatorial Guinea	1990-2021	-1.11<br>(-1.3 to -0.93)	<0.001
Incidence	Eritrea	1990-1992	-88.22<br>(-94.79 to -80.14)	<0.001
Incidence	Eritrea	1992-2021	-0.57<br>(-1.41 to 0.27)	0.182
Incidence	Estonia	1990-1997	-0.43<br>(-0.64 to -0.18)	0.008
Incidence	Estonia	1997-2009	-1.78<br>(-1.9 to -1.67)	0.006
Incidence	Estonia	2009-2014	-4.26<br>(-4.77 to -3.58)	<0.001
Incidence	Estonia	2014-2018	-1.38<br>(-3.61 to -0.92)	<0.001
Incidence	Estonia	2018-2021	0.5<br>(-0.29 to 1.57)	0.180
Incidence	Eswatini	1990-2019	-0.22<br>(-0.57 to 0.03)	0.068
Incidence	Eswatini	2019-2021	18.7<br>(0.99 to 28.63)	0.019
Incidence	Ethiopia	1990-1992	-68.52<br>(-81.51 to -49.34)	<0.001

Incidence	Ethiopia	1992-2019	-1.4<br>(-1.97 to -0.89)	0.004
Incidence	Ethiopia	2019-2021	59.31<br>(12.14 to 141.01)	0.009
Incidence	Fiji	1990-2000	2.39<br>(-0.27 to 19.4)	0.089
Incidence	Fiji	2000-2021	-1.28<br>(-12.59 to -0.35)	0.028
Incidence	Finland	1990-1995	1.67<br>(1.3 to 1.87)	<0.001
Incidence	Finland	1995-2004	2.43<br>(2.34 to 2.56)	<0.001
Incidence	Finland	2004-2007	-0.23<br>(-0.59 to 0.06)	0.071
Incidence	Finland	2007-2011	-1.85<br>(-2.08 to -1.68)	<0.001
Incidence	Finland	2011-2018	-5.09<br>(-5.19 to -4.99)	<0.001
Incidence	Finland	2018-2021	-0.55<br>(-0.9 to -0.27)	0.000
Incidence	France	1990-1995	-1.28<br>(-1.91 to -0.92)	0.002
Incidence	France	1995-2009	-0.05<br>(-0.17 to 0.08)	0.346
Incidence	France	2009-2019	-1.94<br>(-2.32 to -1.74)	0.009
Incidence	France	2019-2021	0.23<br>(-1.4 to 1.09)	0.823
Incidence	Gabon	1990-2021	-0.51<br>(-0.66 to -0.37)	<0.001
Incidence	Gambia	1990-2000	1.31<br>(-0.07 to 7.16)	0.065
Incidence	Gambia	2000-2021	-1.01<br>(-2.68 to -0.55)	0.004
Incidence	Georgia	1990-2021	-0.8<br>(-1.45 to -0.15)	0.014
Incidence	Germany	1990-2006	-0.21<br>(-0.25 to -0.09)	0.035

Incidence	Germany	2006-2010	-0.52 (-2.31 to -0.27)	<0.001
Incidence	Germany	2010-2018	-2.01 (-2.29 to -1.79)	<0.001
Incidence	Germany	2018-2021	-0.1 (-0.56 to 0.61)	0.503
Incidence	Ghana	1990-2001	0.81 (0.21 to 2.9)	0.009
Incidence	Ghana	2001-2021	-0.54 (-1.11 to -0.29)	0.002
Incidence	Greece	1990-1996	-0.49 (-0.86 to -0.34)	0.006
Incidence	Greece	1996-1999	0.25 (-0.19 to 0.44)	0.247
Incidence	Greece	1999-2010	-0.91 (-1.01 to -0.83)	0.016
Incidence	Greece	2010-2015	-2.97 (-3.27 to -0.98)	<0.001
Incidence	Greece	2015-2018	-1.54 (-2.86 to -1.03)	<0.001
Incidence	Greece	2018-2021	-0.12 (-0.55 to 0.53)	0.546
Incidence	Greenland	1990-2003	-1.22 (-1.47 to -0.53)	0.019
Incidence	Greenland	2003-2009	-2.92 (-4.77 to -2.1)	0.002
Incidence	Greenland	2009-2021	-1.08 (-1.33 to -0.6)	0.012
Incidence	Grenada	1990-2021	0.17 (-0.19 to 0.55)	0.323
Incidence	Guam	1990-1996	-0.83 (-3.81 to 0.31)	0.152
Incidence	Guam	1996-2004	0.98 (-2.05 to 3.63)	0.131
Incidence	Guam	2004-2021	-1.02 (-1.46 to -0.21)	0.042
Incidence	Guatemala	1990-1996	-7.07 (-12.63 to -4.65)	<0.001

Incidence	Guatemala	1996-2016	-0.97<br>(-1.41 to -0.53)	0.004
Incidence	Guatemala	2016-2019	10.83<br>(5.59 to 13.79)	0.004
Incidence	Guatemala	2019-2021	-14.86<br>(-20.75 to -6.81)	0.004
Incidence	Guinea	1990-2021	-0.09<br>(-0.68 to 0.51)	0.758
Incidence	Guinea-Bissau	1990-2021	-0.99<br>(-1.59 to -0.39)	0.003
Incidence	Guyana	1990-2008	1.21<br>(0.51 to 3.97)	0.017
Incidence	Guyana	2008-2021	-1.1<br>(-5.52 to 0.11)	0.064
Incidence	Haiti	1990-2021	-0.13<br>(-1.12 to 0.87)	0.782
Incidence	Honduras	1990-2021	-0.56<br>(-1.11 to 0.01)	0.053
Incidence	Hungary	1990-2000	-0.07<br>(-0.14 to -0.01)	0.039
Incidence	Hungary	2000-2010	0.35<br>(0.03 to 0.45)	0.043
Incidence	Hungary	2010-2015	-2.45<br>(-2.65 to 0.34)	0.130
Incidence	Hungary	2015-2018	-1.93<br>(-2.42 to -1.5)	<0.001
Incidence	Hungary	2018-2021	-0.12<br>(-0.43 to 0.37)	0.410
Incidence	Iceland	1990-2021	-0.81<br>(-1.14 to -0.48)	<0.001
Incidence	India	1990-1995	-3.63<br>(-8.35 to -1.55)	0.006
Incidence	India	1995-2001	1.42<br>(-0.54 to 5.85)	0.144
Incidence	India	2001-2015	-2.88<br>(-6.09 to -2.34)	0.023
Incidence	India	2015-2021	-0.12<br>(-1.87 to 4.61)	0.941

Incidence	Indonesia	1990-2021	-1.27<br>(-1.73 to -0.79)	<0.001
Incidence	Iran (Islamic Republic of)	1990-1992	-21.82<br>(-32.08 to -3.21)	0.010
Incidence	Iran (Islamic Republic of)	1992-2003	0.88<br>(-0.79 to 7.39)	0.200
Incidence	Iran (Islamic Republic of)	2003-2021	-3.29<br>(-4.4 to -2.52)	0.012
Incidence	Iraq	1990-2002	-1.83<br>(-3.49 to -0.18)	0.025
Incidence	Iraq	2002-2005	120.41<br>(70.06 to 153.66)	<0.001
Incidence	Iraq	2005-2011	-25.05<br>(-43.68 to -17.66)	<0.001
Incidence	Iraq	2011-2014	61.7<br>(16.49 to 89.45)	0.002
Incidence	Iraq	2014-2021	-28.27<br>(-34.79 to -23.38)	<0.001
Incidence	Ireland	1990-1992	-0.33<br>(-0.6 to 0.19)	0.271
Incidence	Ireland	1992-2005	0.23<br>(-0.24 to 0.38)	0.065
Incidence	Ireland	2005-2010	-0.36<br>(-2.04 to -0.21)	0.001
Incidence	Ireland	2010-2018	-2.34<br>(-2.43 to -2.23)	<0.001
Incidence	Ireland	2018-2021	0.03<br>(-0.29 to 0.3)	0.786
Incidence	Israel	1990-2006	1.84<br>(0 to 9.64)	0.051
Incidence	Israel	2006-2021	-3.33<br>(-12.44 to -1.21)	0.002
Incidence	Italy	1990-1998	-1.27<br>(-1.37 to -1.03)	<0.001
Incidence	Italy	1998-2010	-1.47<br>(-1.71 to -1.4)	0.004
Incidence	Italy	2010-2015	1.68<br>(-1.37 to 1.86)	0.097

Incidence	Italy	2015-2019	-1.11<br>(-1.53 to 1.6)	0.103
Incidence	Italy	2019-2021	0.19<br>(-0.79 to 0.65)	0.557
Incidence	Jamaica	1990-1994	-4.56<br>(-11.26 to -0.8)	0.015
Incidence	Jamaica	1994-2010	0.16<br>(-0.36 to 6.52)	0.334
Incidence	Jamaica	2010-2016	-3.42<br>(-11.79 to -0.65)	0.036
Incidence	Jamaica	2016-2019	27.47<br>(9.69 to 35.11)	0.041
Incidence	Jamaica	2019-2021	-9.24<br>(-22.17 to 8.22)	0.139
Incidence	Japan	1990-2008	-1.08<br>(-1.38 to -0.92)	0.002
Incidence	Japan	2008-2011	1.99<br>(-0.39 to 3.54)	0.102
Incidence	Japan	2011-2014	-7.21<br>(-8.68 to -4.72)	0.010
Incidence	Japan	2014-2021	-1.52<br>(-2.16 to -0.38)	0.024
Incidence	Jordan	1990-2005	-0.07<br>(-0.53 to 2)	0.881
Incidence	Jordan	2005-2021	-1.05<br>(-2.93 to -0.61)	0.028
Incidence	Kazakhstan	1990-1995	-0.18<br>(-0.64 to 0.63)	0.571
Incidence	Kazakhstan	1995-1998	-1.64<br>(-2.13 to 0.08)	0.056
Incidence	Kazakhstan	1998-2005	0.1<br>(-1.15 to 0.82)	0.373
Incidence	Kazakhstan	2005-2010	-1.41<br>(-3.27 to -0.87)	<0.001
Incidence	Kazakhstan	2010-2015	-3.6<br>(-4.34 to -2.17)	0.006
Incidence	Kazakhstan	2015-2021	-0.7<br>(-1.09 to -0.16)	0.024

Incidence	Kenya	1990-2001	2.36 (0.72 to 7.58)	0.005
Incidence	Kenya	2001-2021	-1.33 (-2.75 to -0.69)	0.000
Incidence	Kiribati	1990-2021	-0.93 (-1.5 to -0.35)	0.004
Incidence	Kuwait	1990-1992	-69.75 (-80.97 to -47.88)	<0.001
Incidence	Kuwait	1992-2021	-0.8 (-1.21 to -0.35)	0.002
Incidence	Kyrgyzstan	1990-2021	-1.83 (-2.14 to -1.5)	<0.001
Incidence	Lao People's Democratic Republic	1990-2021	-1.13 (-1.51 to -0.75)	<0.001
Incidence	Latvia	1990-2000	-0.67 (-0.77 to -0.58)	0.007
Incidence	Latvia	2000-2005	-2.58 (-2.98 to -2.23)	<0.001
Incidence	Latvia	2005-2009	-1.7 (-2.2 to -1.29)	<0.001
Incidence	Latvia	2009-2015	-4.01 (-4.3 to -3.82)	<0.001
Incidence	Latvia	2015-2018	-1.42 (-3.37 to -1.03)	0.001
Incidence	Latvia	2018-2021	0.37 (-0.16 to 1.21)	0.100
Incidence	Lebanon	1990-2001	-11.22 (-16.78 to -7.73)	<0.001
Incidence	Lebanon	2001-2021	0.14 (-1.16 to 1.96)	0.776
Incidence	Lesotho	1990-1995	-0.75 (-7.38 to 2.11)	0.412
Incidence	Lesotho	1995-1998	16.34 (7.14 to 21.69)	0.021
Incidence	Lesotho	1998-2001	-9.23 (-12.63 to -1.03)	0.021
Incidence	Lesotho	2001-2021	0.24 (-0.23 to 1.04)	0.216

Incidence	Liberia	1990-1998	-22.65 (-73.05 to -6.3)	0.004
Incidence	Liberia	1998-2021	-3.07 (-5.46 to 5)	0.140
Incidence	Libya	1990-2006	-0.42 (-2.82 to 1.55)	0.624
Incidence	Libya	2006-2016	19.42 (12.85 to 55.82)	0.003
Incidence	Libya	2016-2021	-21.24 (-52.92 to -3.6)	0.019
Incidence	Lithuania	1990-1995	-0.59 (-1.02 to -0.42)	<0.001
Incidence	Lithuania	1995-2001	-0.09 (-0.23 to 0.27)	0.565
Incidence	Lithuania	2001-2010	-0.8 (-0.89 to -0.72)	0.000
Incidence	Lithuania	2010-2015	-4.28 (-4.46 to -4.15)	<0.001
Incidence	Lithuania	2015-2018	-2.18 (-2.71 to -1.85)	<0.001
Incidence	Lithuania	2018-2021	-0.32 (-0.67 to 0.32)	0.148
Incidence	Luxembourg	1990-1995	-1.14 (-1.33 to -0.86)	0.000
Incidence	Luxembourg	1995-1999	-2.5 (-2.95 to -2.18)	<0.001
Incidence	Luxembourg	1999-2003	-1.3 (-1.77 to -0.55)	0.007
Incidence	Luxembourg	2003-2010	0 (-0.2 to 0.36)	0.882
Incidence	Luxembourg	2010-2017	-1.71 (-2.01 to -1.52)	<0.001
Incidence	Luxembourg	2017-2021	-0.21 (-0.61 to 0.37)	0.216
Incidence	Madagascar	1990-2021	-0.44 (-0.71 to -0.17)	0.002
Incidence	Malawi	1990-2021	-0.73 (-0.91 to -0.55)	<0.001

Incidence	Malaysia	1990-1996	1.28 (-0.07 to 4.83)	0.062
Incidence	Malaysia	1996-2021	-0.41 (-0.95 to -0.23)	0.020
Incidence	Maldives	1990-2021	-0.15 (-0.77 to 0.5)	0.541
Incidence	Mali	1990-1995	-12.17 (-36.22 to 0.83)	0.056
Incidence	Mali	1995-1998	84.81 (20.08 to 136.91)	0.022
Incidence	Mali	1998-2005	-22.1 (-49.64 to -10.9)	0.021
Incidence	Mali	2005-2021	2.86 (0.06 to 6.6)	0.046
Incidence	Malta	1990-2000	0.64 (0.49 to 0.75)	<0.001
Incidence	Malta	2000-2005	1.99 (1.59 to 2.7)	<0.001
Incidence	Malta	2005-2011	0.11 (-0.15 to 0.34)	0.334
Incidence	Malta	2011-2019	-3.32 (-3.48 to -3.19)	<0.001
Incidence	Malta	2019-2021	0.28 (-0.72 to 0.94)	0.432
Incidence	Marshall Islands	1990-1998	0.95 (0.91 to 1)	<0.001
Incidence	Marshall Islands	1998-2003	0.2 (0.12 to 0.35)	0.002
Incidence	Marshall Islands	2003-2010	-0.24 (-0.3 to -0.18)	0.006
Incidence	Marshall Islands	2010-2015	-1.49 (-1.58 to -1.42)	<0.001
Incidence	Marshall Islands	2015-2018	-0.5 (-0.9 to -0.35)	0.002
Incidence	Marshall Islands	2018-2021	0.26 (0.1 to 0.54)	0.003
Incidence	Mauritania	1990-2005	0.15 (-0.41 to 1.52)	0.533

Incidence	Mauritania	2005-2021	-1.52 (-2.86 to -1.01)	<0.001
Incidence	Mauritius	1990-2021	-0.18 (-0.32 to -0.04)	0.015
Incidence	Mexico	1990-1995	-7.19 (-7.92 to -6.46)	<0.001
Incidence	Mexico	1995-1999	4.71 (3.6 to 6.2)	0.001
Incidence	Mexico	1999-2005	0.8 (-0.74 to 1.52)	0.192
Incidence	Mexico	2005-2010	7.24 (6.35 to 8.62)	<0.001
Incidence	Mexico	2010-2019	-7.38 (-7.83 to -7.02)	<0.001
Incidence	Mexico	2019-2021	0.65 (-2.58 to 2.7)	0.573
Incidence	Micronesia (Federated States of)	1990-2021	-0.01 (-0.49 to 0.5)	0.938
Incidence	Monaco	1990-1994	-0.39 (-0.74 to -0.22)	<0.001
Incidence	Monaco	1994-1998	0.12 (-0.12 to 0.55)	0.224
Incidence	Monaco	1998-2010	0.59 (0.55 to 0.65)	0.035
Incidence	Monaco	2010-2018	-1.92 (-1.97 to -1.86)	<0.001
Incidence	Monaco	2018-2021	0.02 (-0.18 to 0.19)	0.755
Incidence	Mongolia	1990-2021	-0.09 (-0.39 to 0.19)	0.488
Incidence	Montenegro	1990-2005	0.24 (0.18 to 0.32)	0.004
Incidence	Montenegro	2005-2010	-1.06 (-1.44 to -0.33)	0.023
Incidence	Montenegro	2010-2015	-2.11 (-2.76 to -1.77)	<0.001
Incidence	Montenegro	2015-2021	-0.71 (-0.96 to -0.36)	0.008

Incidence	Morocco	1990-2002	-0.13 (-0.48 to 0.39)	0.574
Incidence	Morocco	2002-2021	-1.54 (-1.78 to -1.36)	<0.001
Incidence	Mozambique	1990-1993	-33.26 (-46.11 to -21.58)	<0.001
Incidence	Mozambique	1993-2017	-0.46 (-0.89 to -0.06)	0.026
Incidence	Mozambique	2017-2021	14.09 (6.87 to 28.53)	<0.001
Incidence	Myanmar	1990-2021	-0.32 (-1.48 to 0.87)	0.565
Incidence	Namibia	1990-2000	2.06 (0.29 to 9.14)	0.016
Incidence	Namibia	2000-2021	-0.89 (-2.7 to -0.34)	0.007
Incidence	Nauru	1990-2000	1.5 (1.45 to 1.54)	<0.001
Incidence	Nauru	2000-2005	0.77 (0.67 to 0.91)	<0.001
Incidence	Nauru	2005-2010	-0.63 (-0.75 to -0.47)	0.035
Incidence	Nauru	2010-2015	-1.55 (-1.73 to -1.38)	<0.001
Incidence	Nauru	2015-2018	-0.53 (-1.33 to -0.33)	0.002
Incidence	Nauru	2018-2021	0.2 (-0.01 to 0.56)	0.062
Incidence	Nepal	1990-2004	9.06 (0.36 to 19.07)	0.049
Incidence	Nepal	2004-2007	-26.69 (-35.04 to 17.19)	0.082
Incidence	Nepal	2007-2021	-0.52 (-3.68 to 15.23)	0.952
Incidence	Netherlands	1990-1999	-2.16 (-3.25 to -1.74)	0.000
Incidence	Netherlands	1999-2012	-0.93 (-1.29 to -0.48)	0.010

Incidence	Netherlands	2012-2021	0.78<br>(0.21 to 2.04)	0.015
Incidence	New Zealand	1990-2000	1.57<br>(1.33 to 1.89)	0.000
Incidence	New Zealand	2000-2006	-0.52<br>(-1.31 to 0.35)	0.128
Incidence	New Zealand	2006-2021	-2.13<br>(-2.31 to -1.99)	<0.001
Incidence	Nicaragua	1990-2021	-0.83<br>(-1.39 to -0.27)	0.005
Incidence	Niger	1990-2011	-1.13<br>(-1.93 to -0.57)	<0.001
Incidence	Niger	2011-2021	4.09<br>(2.05 to 8.46)	<0.001
Incidence	Nigeria	1990-2000	2.19<br>(0.52 to 10.57)	0.004
Incidence	Nigeria	2000-2021	-0.53<br>(-3.5 to 0.03)	0.066
Incidence	Niue	1990-2021	0.08<br>(-0.48 to 0.65)	0.699
Incidence	North Macedonia	1990-1998	-0.19<br>(-3.48 to 1.33)	0.542
Incidence	North Macedonia	1998-2001	6.18<br>(-2.56 to 8.37)	0.117
Incidence	North Macedonia	2001-2021	-1.43<br>(-1.98 to -0.39)	0.044
Incidence	Northern Mariana Islands	1990-2021	0.51<br>(0.2 to 0.82)	0.002
Incidence	Norway	1990-2002	-0.74<br>(-1.42 to -0.32)	0.018
Incidence	Norway	2002-2011	1.18<br>(0.38 to 5.46)	0.032
Incidence	Norway	2011-2014	-7.26<br>(-8.98 to -3.54)	0.034
Incidence	Norway	2014-2021	-1.64<br>(-2.53 to 0.26)	0.069
Incidence	Oman	1990-1999	-1.21<br>(-1.46 to -1.02)	<0.001

Incidence	Oman	1999-2009	0.08 (-0.11 to 0.41)	0.308
Incidence	Oman	2009-2018	-1.96 (-2.32 to -1.76)	<0.001
Incidence	Oman	2018-2021	0.73 (-0.21 to 2.14)	0.102
Incidence	Pakistan	1990-2004	-0.58 (-3.32 to 1.12)	0.426
Incidence	Pakistan	2004-2010	19.64 (7.99 to 52.24)	0.001
Incidence	Pakistan	2010-2021	-10.64 (-15.92 to -6.98)	0.001
Incidence	Palau	1990-1996	-0.93 (-0.99 to -0.88)	<0.001
Incidence	Palau	1996-2000	0.1 (-0.39 to 0.19)	0.114
Incidence	Palau	2000-2005	0.75 (0.4 to 0.83)	0.003
Incidence	Palau	2005-2010	-0.48 (-0.54 to 0.77)	0.078
Incidence	Palau	2010-2015	-0.63 (-0.76 to -0.03)	0.044
Incidence	Palau	2015-2021	0.15 (0.09 to 0.23)	0.009
Incidence	Palestine	1990-1998	-12.4 (-20.33 to -5.55)	0.021
Incidence	Palestine	1998-2001	105.5 (8.95 to 144)	0.037
Incidence	Palestine	2001-2008	1.63 (-23.5 to 33.12)	0.991
Incidence	Palestine	2008-2013	-37.47 (-54.91 to -12.13)	0.033
Incidence	Palestine	2013-2021	2.69 (-2.39 to 9.53)	0.246
Incidence	Panama	1990-2006	-0.33 (-0.57 to 0.3)	0.142
Incidence	Panama	2006-2021	-1.11 (-1.84 to -0.84)	<0.001

Incidence	Papua New Guinea	1990-2021	-0.32 (-1.09 to 0.45)	0.382
Incidence	Paraguay	1990-1998	0.5 (0.19 to 1.6)	0.001
Incidence	Paraguay	1998-2011	-0.03 (-0.83 to 0.12)	0.619
Incidence	Paraguay	2011-2018	-1.88 (-2.68 to -1.5)	<0.001
Incidence	Paraguay	2018-2021	1.11 (0.05 to 2.66)	0.042
Incidence	Peru	1990-1994	-21.03 (-26.85 to -17.13)	<0.001
Incidence	Peru	1994-2000	-3.99 (-7.33 to -2.29)	<0.001
Incidence	Peru	2000-2021	-0.63 (-0.94 to -0.2)	0.018
Incidence	Philippines	1990-1993	-22.42 (-42.98 to -0.39)	0.034
Incidence	Philippines	1993-2021	-0.28 (-3.06 to 4.41)	0.820
Incidence	Poland	1990-1999	0.13 (0.06 to 0.21)	0.009
Incidence	Poland	1999-2008	-0.6 (-0.66 to -0.48)	0.016
Incidence	Poland	2008-2011	-1.13 (-1.33 to -0.85)	0.000
Incidence	Poland	2011-2014	-3.96 (-4.18 to -3.69)	<0.001
Incidence	Poland	2014-2018	-2.53 (-2.71 to -2.21)	<0.001
Incidence	Poland	2018-2021	-0.11 (-0.42 to 0.16)	0.316
Incidence	Portugal	1990-1993	-0.62 (-0.91 to -0.14)	0.026
Incidence	Portugal	1993-1996	-1.91 (-2.59 to -1.64)	<0.001
Incidence	Portugal	1996-2014	-3.28 (-3.32 to -3.25)	<0.001

Incidence	Portugal	2014-2018	-1.42 (-2.31 to -1.23)	<0.001
Incidence	Portugal	2018-2021	-0.2 (-0.65 to 0.47)	0.373
Incidence	Puerto Rico	1990-1997	1.87 (0.21 to 7.43)	0.026
Incidence	Puerto Rico	1997-2016	-0.51 (-3.41 to -0.08)	0.040
Incidence	Puerto Rico	2016-2019	10.43 (-0.32 to 14.95)	0.059
Incidence	Puerto Rico	2019-2021	-8.02 (-14.17 to 3.47)	0.102
Incidence	Qatar	1990-1995	0.64 (0.38 to 1)	<0.001
Incidence	Qatar	1995-2000	-0.74 (-1.24 to -0.48)	<0.001
Incidence	Qatar	2000-2005	1.93 (1.64 to 2.3)	<0.001
Incidence	Qatar	2005-2011	-0.8 (-1.07 to -0.54)	0.000
Incidence	Qatar	2011-2019	-2.61 (-2.87 to -2.46)	<0.001
Incidence	Qatar	2019-2021	0 (-1.14 to 0.64)	0.927
Incidence	Republic of Korea	1990-1998	-0.79 (-0.92 to -0.62)	0.012
Incidence	Republic of Korea	1998-2001	-3.04 (-3.51 to -1.64)	0.002
Incidence	Republic of Korea	2001-2005	-4.52 (-5.22 to -4.08)	<0.001
Incidence	Republic of Korea	2005-2010	-1.62 (-1.95 to -0.78)	<0.001
Incidence	Republic of Korea	2010-2017	-2.5 (-3.33 to -2.3)	<0.001
Incidence	Republic of Korea	2017-2021	-0.77 (-1.35 to 0.22)	0.072
Incidence	Republic of Moldova	1990-1992	9.97 (4.08 to 14.71)	0.002

Incidence	Republic of Moldova	1992-1995	-6.37 (-7.92 to -3.47)	0.001
Incidence	Republic of Moldova	1995-2021	-1.66 (-1.81 to -1.47)	0.002
Incidence	Romania	1990-1995	0.31 (0.21 to 0.41)	0.000
Incidence	Romania	1995-2004	-1.66 (-1.72 to -1.6)	0.002
Incidence	Romania	2004-2010	-0.98 (-1.12 to -0.8)	<0.001
Incidence	Romania	2010-2014	-2.12 (-2.37 to -1.89)	<0.001
Incidence	Romania	2014-2018	-1.43 (-1.68 to -1.13)	<0.001
Incidence	Romania	2018-2021	-0.22 (-0.48 to 0.2)	0.145
Incidence	Russian Federation	1990-1997	1.57 (-2.75 to 3.18)	0.275
Incidence	Russian Federation	1997-2000	6.69 (-5.39 to 9.16)	0.241
Incidence	Russian Federation	2000-2017	-4.55 (-7.79 to -3.9)	0.010
Incidence	Russian Federation	2017-2021	0.01 (-3.46 to 6.5)	0.962
Incidence	Rwanda	1990-2002	-13.53 (-58.05 to -5.92)	0.000
Incidence	Rwanda	2002-2021	-1.55 (-3.74 to 10.19)	0.378
Incidence	Saint Kitts and Nevis	1990-2012	1.73 (1.34 to 2.42)	0.001
Incidence	Saint Kitts and Nevis	2012-2021	-1.51 (-6.59 to 0.06)	0.056
Incidence	Saint Lucia	1990-2010	0.92 (0.42 to 2.13)	0.002
Incidence	Saint Lucia	2010-2021	-1.58 (-5.81 to -0.35)	0.009
Incidence	Saint Vincent and the Grenadines	1990-2021	0.02 (-0.36 to 0.4)	0.921

Incidence	Samoa	1990-2021	-0.61 (-1.41 to 0.25)	0.146
Incidence	San Marino	1990-1998	-0.32 (-0.41 to -0.25)	0.001
Incidence	San Marino	1998-2011	0.09 (0.05 to 0.14)	0.026
Incidence	San Marino	2011-2015	-2.08 (-2.29 to -0.2)	0.046
Incidence	San Marino	2015-2018	-1.39 (-1.86 to -0.98)	<0.001
Incidence	San Marino	2018-2021	0.34 (0.07 to 0.67)	0.013
Incidence	Sao Tome and Principe	1990-2001	-1.67 (-4.48 to -0.93)	0.007
Incidence	Sao Tome and Principe	2001-2019	-0.07 (-0.49 to 0.9)	0.719
Incidence	Sao Tome and Principe	2019-2021	16.16 (6.74 to 19.57)	0.000
Incidence	Saudi Arabia	1990-2016	-0.12 (-0.29 to 0.39)	0.328
Incidence	Saudi Arabia	2016-2021	-2.4 (-6.53 to -0.46)	0.003
Incidence	Senegal	1990-2021	-1.12 (-1.58 to -0.66)	<0.001
Incidence	Serbia	1990-2021	-0.97 (-1.71 to -0.19)	0.013
Incidence	Seychelles	1990-2021	-0.39 (-0.76 to -0.02)	0.038
Incidence	Sierra Leone	1990-1998	31.38 (20.45 to 47.28)	<0.001
Incidence	Sierra Leone	1998-2002	-47.3 (-59.92 to -31.31)	<0.001
Incidence	Sierra Leone	2002-2021	-0.5 (-1.73 to 0.86)	0.490
Incidence	Singapore	1990-1994	-1.51 (-2.09 to -1.17)	<0.001
Incidence	Singapore	1994-1999	0.18 (-0.09 to 0.7)	0.121

Incidence	Singapore	1999-2003	-1.58 (-2.15 to -1.22)	<0.001
Incidence	Singapore	2003-2010	0.34 (0.17 to 0.6)	0.003
Incidence	Singapore	2010-2015	-2.03 (-2.52 to -1.69)	<0.001
Incidence	Singapore	2015-2021	-0.88 (-1.1 to -0.44)	0.006
Incidence	Slovakia	1990-1998	0.27 (0.14 to 0.39)	0.026
Incidence	Slovakia	1998-2005	-0.55 (-0.84 to 0.35)	0.100
Incidence	Slovakia	2005-2010	-0.27 (-0.6 to -0.02)	0.038
Incidence	Slovakia	2010-2015	-2.19 (-2.44 to -0.3)	0.002
Incidence	Slovakia	2015-2019	-0.88 (-2.18 to -0.71)	<0.001
Incidence	Slovakia	2019-2021	0.08 (-0.58 to 0.48)	0.830
Incidence	Slovenia	1990-1995	-1.35 (-2.18 to -0.69)	0.002
Incidence	Slovenia	1995-2005	1.74 (1.48 to 2.68)	0.004
Incidence	Slovenia	2005-2010	-0.04 (-2.26 to 1.02)	0.827
Incidence	Slovenia	2010-2018	-3.61 (-4.96 to -3.28)	<0.001
Incidence	Slovenia	2018-2021	-0.45 (-1.82 to 1.68)	0.392
Incidence	Solomon Islands	1990-2021	0.43 (-0.14 to 1.01)	0.137
Incidence	Somalia	1990-1993	-52.51 (-79.24 to 1.71)	0.065
Incidence	Somalia	1993-2021	4.09 (1.63 to 7.28)	0.031
Incidence	South Africa	1990-1992	2.42 (-2.12 to 5.53)	0.205

Incidence	South Africa	1992-1995	-4.33<br>(-5.52 to -0.15)	0.045
Incidence	South Africa	1995-2009	-0.86<br>(-2.51 to -0.17)	0.036
Incidence	South Africa	2009-2018	-3.32<br>(-5.12 to -2.73)	0.000
Incidence	South Africa	2018-2021	1.14<br>(-1.35 to 4.49)	0.295
Incidence	South Sudan	1990-2021	4.02<br>(2.14 to 6.18)	0.000
Incidence	Spain	1990-1995	0.22<br>(-0.18 to 0.8)	0.212
Incidence	Spain	1995-2000	-2.05<br>(-2.99 to -1.59)	<0.001
Incidence	Spain	2000-2009	0.13<br>(-0.12 to 0.49)	0.229
Incidence	Spain	2009-2018	-2.25<br>(-2.7 to -1.99)	<0.001
Incidence	Spain	2018-2021	0.86<br>(-0.47 to 2.58)	0.151
Incidence	Sri Lanka	1990-2003	-15.56<br>(-26.07 to -8.97)	0.018
Incidence	Sri Lanka	2003-2009	49.62<br>(20.39 to 186.57)	0.028
Incidence	Sri Lanka	2009-2013	-47.39<br>(-67.85 to -19.51)	0.029
Incidence	Sri Lanka	2013-2021	-3.01<br>(-11.47 to 17.38)	0.681
Incidence	Sudan	1990-1994	-22.52<br>(-47.51 to -11.5)	0.008
Incidence	Sudan	1994-1997	26.01<br>(4 to 38.41)	0.023
Incidence	Sudan	1997-2021	-3.94<br>(-5.17 to -3.05)	0.007
Incidence	Suriname	1990-2010	0.31<br>(0.14 to 0.67)	0.001
Incidence	Suriname	2010-2021	-0.68<br>(-1.9 to -0.25)	0.002

Incidence	Sweden	1990-2000	-0.67 (-0.74 to -0.62)	<0.001
Incidence	Sweden	2000-2008	0.3 (0.22 to 0.44)	<0.001
Incidence	Sweden	2008-2011	-0.68 (-0.9 to -0.36)	<0.001
Incidence	Sweden	2011-2014	-4.27 (-4.51 to -4.01)	<0.001
Incidence	Sweden	2014-2018	-2.52 (-2.68 to -2.27)	<0.001
Incidence	Sweden	2018-2021	0.01 (-0.27 to 0.26)	0.970
Incidence	Switzerland	1990-1994	0.59 (-0.16 to 0.96)	0.094
Incidence	Switzerland	1994-2000	1.59 (1.39 to 2.03)	0.004
Incidence	Switzerland	2000-2005	-2.13 (-2.61 to -1.89)	0.003
Incidence	Switzerland	2005-2010	-1.16 (-1.49 to -0.65)	<0.001
Incidence	Switzerland	2010-2017	-3.73 (-3.95 to -3.54)	<0.001
Incidence	Switzerland	2017-2021	-0.76 (-1.16 to -0.38)	0.025
Incidence	Syrian Arab Republic	1990-2010	1.14 (0.33 to 1.9)	0.008
Incidence	Syrian Arab Republic	2010-2013	246.86 (179.65 to 291.71)	0.001
Incidence	Syrian Arab Republic	2013-2018	-10.51 (-23.58 to 14.83)	0.220
Incidence	Syrian Arab Republic	2018-2021	-56.82 (-69.78 to -45.24)	<0.001
Incidence	Taiwan (Province of China)	1990-2021	-1.96 (-2.32 to -1.59)	<0.001
Incidence	Tajikistan	1990-2021	-2.62 (-3.79 to -1.48)	<0.001
Incidence	Thailand	1990-2002	-0.69 (-4.08 to 0.53)	0.134

Incidence	Thailand	2002-2005	8.43 (-2.83 to 11.64)	0.077
Incidence	Thailand	2005-2021	-2.52 (-4.36 to -1.13)	0.041
Incidence	Timor-Leste	1990-1996	-40.19 (-63.92 to -27.55)	<0.001
Incidence	Timor-Leste	1996-1999	187.22 (66.91 to 342.15)	0.000
Incidence	Timor-Leste	1999-2004	-50.66 (-75.63 to -37.91)	0.000
Incidence	Timor-Leste	2004-2021	-0.22 (-2.76 to 2.93)	0.939
Incidence	Togo	1990-2021	-0.72 (-1.11 to -0.31)	0.002
Incidence	Tokelau	1990-1994	-0.59 (-1.04 to -0.28)	0.001
Incidence	Tokelau	1994-1999	1.5 (1.24 to 1.84)	<0.001
Incidence	Tokelau	1999-2004	-0.61 (-0.98 to -0.35)	0.000
Incidence	Tokelau	2004-2009	1.42 (1.21 to 1.71)	<0.001
Incidence	Tokelau	2009-2015	-2.45 (-2.62 to -2.29)	<0.001
Incidence	Tokelau	2015-2021	-0.06 (-0.25 to 0.13)	0.480
Incidence	Tonga	1990-2021	-0.6 (-1.02 to -0.16)	0.012
Incidence	Trinidad and Tobago	1990-2011	1.16 (0.11 to 2.47)	0.046
Incidence	Trinidad and Tobago	2011-2016	-4.7 (-13.83 to 2.61)	0.103
Incidence	Trinidad and Tobago	2016-2019	32.26 (-4.05 to 45.16)	0.085
Incidence	Trinidad and Tobago	2019-2021	-19.4 (-33.5 to 9.59)	0.108
Incidence	Tunisia	1990-2015	-0.45 (-0.88 to 0.94)	0.202

Incidence	Tunisia	2015-2021	-3.15 (-8.93 to -0.62)	0.038
Incidence	Turkey	1990-1994	12.37 (3.79 to 29.53)	0.003
Incidence	Turkey	1994-2002	-7.45 (-18.13 to -4.59)	0.003
Incidence	Turkey	2002-2016	1.3 (0.17 to 8.47)	0.031
Incidence	Turkey	2016-2021	-6.41 (-16.66 to -1.3)	0.014
Incidence	Turkmenistan	1990-2021	-1.41 (-1.76 to -1.05)	<0.001
Incidence	Tuvalu	1990-1993	1.82 (1.34 to 2.09)	<0.001
Incidence	Tuvalu	1993-2006	-0.05 (-0.1 to -0.01)	0.024
Incidence	Tuvalu	2006-2009	-3.34 (-3.55 to -3.06)	<0.001
Incidence	Tuvalu	2009-2016	-1.58 (-1.73 to -1.42)	<0.001
Incidence	Tuvalu	2016-2021	-0.07 (-0.28 to 0.24)	0.384
Incidence	Uganda	1990-2021	-1.18 (-2.04 to -0.29)	0.013
Incidence	Ukraine	1990-2021	-1.02 (-1.4 to -0.63)	<0.001
Incidence	United Arab Emirates	1990-1999	0.74 (-0.33 to 3.25)	0.087
Incidence	United Arab Emirates	1999-2008	-0.79 (-2.48 to 0.91)	0.144
Incidence	United Arab Emirates	2008-2021	-2.14 (-4.18 to -0.88)	0.037
Incidence	United Kingdom	1990-1995	-1.24 (-1.69 to -1.01)	<0.001
Incidence	United Kingdom	1995-2001	-0.28 (-0.79 to -0.02)	0.033
Incidence	United Kingdom	2001-2010	0.18 (0.06 to 0.49)	0.023

Incidence	United Kingdom	2010-2015	-3.86 (-4.22 to -3.62)	0.023
Incidence	United Kingdom	2015-2018	-2.36 (-3.42 to -1.38)	<0.001
Incidence	United Kingdom	2018-2021	-0.17 (-0.67 to 0.64)	0.573
Incidence	United Republic of Tanzania	1990-2021	-0.39 (-0.49 to -0.29)	<0.001
Incidence	United States Virgin Islands	1990-1999	1.88 (0.46 to 5.22)	0.010
Incidence	United States Virgin Islands	1999-2021	-1.86 (-2.44 to -1.45)	<0.001
Incidence	United States of America	1990-1996	-0.85 (-1.17 to -0.48)	0.004
Incidence	United States of America	1996-2000	-7.34 (-7.81 to -6.76)	0.005
Incidence	United States of America	2000-2005	-1.12 (-1.64 to -0.4)	0.025
Incidence	United States of America	2005-2015	-2.27 (-2.65 to -2.1)	0.000
Incidence	United States of America	2015-2019	1.46 (0.76 to 2.61)	0.040
Incidence	United States of America	2019-2021	-1.54 (-2.77 to -0.16)	0.042
Incidence	Uruguay	1990-1994	0.62 (0.46 to 0.88)	<0.001
Incidence	Uruguay	1994-2000	-0.02 (-0.14 to 0.1)	0.673
Incidence	Uruguay	2000-2005	-0.72 (-0.97 to -0.59)	0.002
Incidence	Uruguay	2005-2011	0.29 (0.21 to 0.41)	0.003
Incidence	Uruguay	2011-2019	-1.32 (-1.38 to -1.26)	<0.001
Incidence	Uruguay	2019-2021	0.58 (0.2 to 0.84)	0.008
Incidence	Uzbekistan	1990-2005	-0.09 (-0.42 to 0.37)	0.623

Incidence	Uzbekistan	2005-2021	-1.97 (-2.36 to -1.67)	<0.001
Incidence	Vanuatu	1990-2021	-0.33 (-0.92 to 0.26)	0.247
Incidence	Venezuela (Bolivarian Republic of)	1990-2021	-0.77 (-1.22 to -0.3)	0.003
Incidence	Viet Nam	1990-2009	0.59 (0.18 to 2.14)	0.016
Incidence	Viet Nam	2009-2021	-1.14 (-4.51 to -0.26)	0.019
Incidence	Yemen	1990-2005	-0.8 (-1.93 to 0.08)	0.071
Incidence	Yemen	2005-2014	9.92 (6.11 to 13.02)	0.022
Incidence	Yemen	2014-2018	49.44 (27.75 to 86.54)	0.006
Incidence	Yemen	2018-2021	-18.92 (-47.76 to 11.6)	0.092
Incidence	Zambia	1990-2004	-0.33 (-1.13 to -0.12)	0.014
Incidence	Zambia	2004-2007	1.48 (-1.16 to 2.3)	0.114
Incidence	Zambia	2007-2017	-1.98 (-3.11 to -1.61)	0.008
Incidence	Zambia	2017-2021	0.79 (-0.55 to 3.22)	0.222
Incidence	Zimbabwe	1990-2021	-0.11 (-0.37 to 0.15)	0.361

APC annual percentage change, *CI* confidence interval.

Supplementary Table 18 Joinpoint regression analysis of prevalence of spinal cord injury from 1990 to 2021 among children and adolescents at national level

Measure	Location	Year	APC (95% CI)	P
Prevalence	Afghanistan	1990-2005	-13.07 (-13.36 to -12.71)	<0.001
Prevalence	Afghanistan	2005-2009	6.97 (5.97 to 8.19)	0.003
Prevalence	Afghanistan	2009-2013	2.14 (0.51 to 3.26)	0.017
Prevalence	Afghanistan	2013-2019	10.01 (9.28 to 11.64)	<0.001
Prevalence	Afghanistan	2019-2021	1.42 (-1.65 to 5.28)	0.264
Prevalence	Albania	1990-1995	-0.21 (-2.33 to 0.89)	0.620
Prevalence	Albania	1995-1998	10.35 (6.58 to 11.87)	<0.001
Prevalence	Albania	1998-2008	-0.4 (-0.93 to 0.23)	0.158
Prevalence	Albania	2008-2021	-2.32 (-2.78 to -2.01)	<0.001
Prevalence	Algeria	1990-1993	-0.4 (-1.14 to 0.31)	0.185
Prevalence	Algeria	1993-1999	4.4 (3.99 to 4.9)	<0.001
Prevalence	Algeria	1999-2004	-1.48 (-2.07 to 0.99)	0.084
Prevalence	Algeria	2004-2009	-3.64 (-4.62 to -2.75)	<0.001
Prevalence	Algeria	2009-2017	-5.45 (-5.81 to -5.22)	<0.001
Prevalence	Algeria	2017-2021	-0.11 (-0.66 to 0.4)	0.639
Prevalence	American Samoa	1990-2001	0.38 (-0.08 to 0.72)	0.088
Prevalence	American Samoa	2001-2008	2.87 (2.18 to 4.06)	<0.001
Prevalence	American Samoa	2008-2011	30.4 (23.58 to 33.74)	<0.001

Prevalence	American Samoa	2011-2021	-3.1 (-4.53 to -1.89)	<0.001
Prevalence	Andorra	1990-2000	-0.9 (-0.99 to -0.83)	<0.001
Prevalence	Andorra	2000-2005	0.15 (-0.37 to 0.42)	0.243
Prevalence	Andorra	2005-2010	0.88 (0.7 to 1.18)	0.003
Prevalence	Andorra	2010-2015	-1.59 (-1.88 to -1.43)	0.014
Prevalence	Andorra	2015-2019	-0.62 (-1.06 to -0.28)	0.000
Prevalence	Andorra	2019-2021	1.4 (0.73 to 1.8)	0.001
Prevalence	Angola	1990-2001	-2.23 (-3.62 to -0.42)	0.024
Prevalence	Angola	2001-2014	-8.8 (-9.68 to -8.16)	0.006
Prevalence	Angola	2014-2018	-3.76 (-6.72 to -2.32)	<0.001
Prevalence	Angola	2018-2021	0.67 (-0.98 to 3.07)	0.345
Prevalence	Antigua and Barbuda	1990-1996	1.03 (0.51 to 1.48)	0.003
Prevalence	Antigua and Barbuda	1996-1999	11.3 (8.46 to 12.26)	<0.001
Prevalence	Antigua and Barbuda	1999-2013	-0.47 (-0.74 to -0.19)	0.004
Prevalence	Antigua and Barbuda	2013-2018	-4.63 (-6.29 to -3.8)	<0.001
Prevalence	Antigua and Barbuda	2018-2021	0.35 (-1.13 to 2.71)	0.525
Prevalence	Argentina	1990-2000	0.26 (0.16 to 0.36)	0.006
Prevalence	Argentina	2000-2005	2.75 (2.11 to 3.05)	0.012
Prevalence	Argentina	2005-2010	0.05 (-0.18 to 1.92)	0.511

Prevalence	Argentina	2010-2013	-2.35<br>(-2.86 to -0.28)	0.005
Prevalence	Argentina	2013-2019	-3.51<br>(-3.81 to -3.32)	<0.001
Prevalence	Argentina	2019-2021	1.68<br>(0.83 to 2.36)	0.003
Prevalence	Armenia	1990-2005	-2.84<br>(-4.02 to -1.41)	0.011
Prevalence	Armenia	2005-2008	-27.71<br>(-29.86 to -18.39)	0.017
Prevalence	Armenia	2008-2014	-3.49<br>(-7.54 to -2.73)	<0.001
Prevalence	Armenia	2014-2019	-0.33<br>(-1.73 to 2.1)	0.552
Prevalence	Armenia	2019-2021	78.34<br>(65 to 103.83)	<0.001
Prevalence	Australia	1990-1995	-0.67<br>(-0.88 to -0.52)	<0.001
Prevalence	Australia	1995-2005	0.54<br>(0.47 to 0.62)	0.013
Prevalence	Australia	2005-2010	-0.31<br>(-0.53 to -0.07)	0.044
Prevalence	Australia	2010-2016	-2.21<br>(-2.42 to -1.99)	0.001
Prevalence	Australia	2016-2019	-1.23<br>(-2.05 to -1.03)	<0.001
Prevalence	Australia	2019-2021	0.56<br>(-0.04 to 0.98)	0.061
Prevalence	Austria	1990-1992	-2.28<br>(-2.77 to -1.43)	<0.001
Prevalence	Austria	1992-1995	-1.03<br>(-1.28 to 1.37)	0.238
Prevalence	Austria	1995-2002	1.25<br>(0.52 to 1.6)	0.002
Prevalence	Austria	2002-2010	0.55<br>(-2.02 to 0.68)	0.072
Prevalence	Austria	2010-2018	-2.42<br>(-2.53 to -2.3)	<0.001

Prevalence	Austria	2018-2021	0.11<br>(-0.3 to 0.48)	0.455
Prevalence	Azerbaijan	1990-1994	9.56<br>(5.78 to 14.4)	<0.001
Prevalence	Azerbaijan	1994-2019	-2.18<br>(-2.4 to -1.95)	<0.001
Prevalence	Azerbaijan	2019-2021	23.02<br>(14.05 to 27.69)	<0.001
Prevalence	Bahamas	1990-1993	-0.99<br>(-2.5 to 0.06)	0.066
Prevalence	Bahamas	1993-2009	0.76<br>(0.67 to 0.94)	0.000
Prevalence	Bahamas	2009-2014	-1.5<br>(-2.65 to -0.93)	0.002
Prevalence	Bahamas	2014-2018	1.14<br>(0.36 to 2.27)	0.007
Prevalence	Bahamas	2018-2021	13.68<br>(11.84 to 15.53)	<0.001
Prevalence	Bahrain	1990-1994	3.57<br>(3.05 to 4.19)	<0.001
Prevalence	Bahrain	1994-1998	0.29<br>(-0.22 to 1.15)	0.218
Prevalence	Bahrain	1998-2002	-1.49<br>(-2.29 to -1.04)	0.017
Prevalence	Bahrain	2002-2005	1.76<br>(0.8 to 2.36)	0.017
Prevalence	Bahrain	2005-2010	-3.76<br>(-4.41 to -3.36)	0.005
Prevalence	Bahrain	2010-2021	-0.39<br>(-0.55 to -0.23)	0.003
Prevalence	Bangladesh	1990-1992	53.84<br>(43.96 to 59.98)	<0.001
Prevalence	Bangladesh	1992-2008	-3.36<br>(-3.86 to -2.58)	<0.001
Prevalence	Bangladesh	2008-2011	-11.87<br>(-13.13 to -8.69)	<0.001
Prevalence	Bangladesh	2011-2021	-1.08<br>(-1.44 to -0.64)	0.001

Prevalence	Barbados	1990-1994	2.47<br>(2.11 to 2.84)	<0.001
Prevalence	Barbados	1994-1997	-1.51<br>(-1.86 to -0.93)	0.001
Prevalence	Barbados	1997-2004	0.05<br>(-0.2 to 0.31)	0.488
Prevalence	Barbados	2004-2010	0.85<br>(0.62 to 1.4)	0.007
Prevalence	Barbados	2010-2015	-1.47<br>(-2.07 to -1.12)	0.004
Prevalence	Barbados	2015-2021	-0.16<br>(-0.41 to 0.28)	0.285
Prevalence	Belarus	1990-2001	1.49<br>(1.39 to 1.6)	<0.001
Prevalence	Belarus	2001-2005	-0.36<br>(-0.76 to 0.24)	0.140
Prevalence	Belarus	2005-2009	-2.52<br>(-3.02 to -2.01)	0.002
Prevalence	Belarus	2009-2015	-4.52<br>(-5.2 to -4.28)	<0.001
Prevalence	Belarus	2015-2018	-2.54<br>(-3.82 to -1.08)	<0.001
Prevalence	Belarus	2018-2021	0.97<br>(0.31 to 1.96)	0.010
Prevalence	Belgium	1990-1996	-0.57<br>(-1.19 to -0.13)	0.020
Prevalence	Belgium	1996-2005	3.74<br>(3.45 to 4.16)	<0.001
Prevalence	Belgium	2005-2010	0.63<br>(-0.52 to 1.29)	0.130
Prevalence	Belgium	2010-2018	-6.48<br>(-6.84 to -6.19)	<0.001
Prevalence	Belgium	2018-2021	-0.01<br>(-1.31 to 1.55)	0.985
Prevalence	Belize	1990-1995	3.02<br>(2.06 to 4.1)	0.003
Prevalence	Belize	1995-2003	-0.45<br>(-1.13 to 1.61)	0.236

Prevalence	Belize	2003-2012	-1.49 (-2.13 to -0.47)	0.040
Prevalence	Belize	2012-2016	-2.75 (-3.86 to -0.3)	0.036
Prevalence	Belize	2016-2021	1.58 (0.83 to 3.09)	0.014
Prevalence	Benin	1990-2011	0.17 (0.15 to 0.18)	<0.001
Prevalence	Benin	2011-2015	-1.5 (-1.74 to -1.17)	0.012
Prevalence	Benin	2015-2019	-0.19 (-0.76 to 0.06)	0.062
Prevalence	Benin	2019-2021	1.16 (0.57 to 1.51)	0.000
Prevalence	Bermuda	1990-1995	0.27 (0 to 0.43)	0.050
Prevalence	Bermuda	1995-2004	1.26 (0.98 to 1.37)	<0.001
Prevalence	Bermuda	2004-2010	0.53 (0.4 to 1.3)	<0.001
Prevalence	Bermuda	2010-2015	-1.35 (-1.59 to 0.56)	0.158
Prevalence	Bermuda	2015-2019	-0.16 (-1.43 to 0)	0.053
Prevalence	Bermuda	2019-2021	0.7 (0.02 to 1.06)	0.040
Prevalence	Bhutan	1990-1999	2.75 (2.13 to 3.21)	0.000
Prevalence	Bhutan	1999-2002	11.23 (7.31 to 12.78)	<0.001
Prevalence	Bhutan	2002-2012	-0.5 (-1.23 to 0.34)	0.191
Prevalence	Bhutan	2012-2021	-3.45 (-4.31 to -2.88)	<0.001
Prevalence	Bolivia (Plurinational State of)	1990-2002	-0.17 (-0.21 to -0.13)	0.007
Prevalence	Bolivia (Plurinational State of)	2002-2008	-0.54 (-0.65 to -0.4)	0.001

Prevalence	Bolivia (Plurinational State of)	2008-2011	-1.37 (-1.54 to -0.91)	0.000
Prevalence	Bolivia (Plurinational State of)	2011-2014	-2.59 (-2.77 to -2.31)	<0.001
Prevalence	Bolivia (Plurinational State of)	2014-2018	-1.54 (-1.73 to -1.22)	<0.001
Prevalence	Bolivia (Plurinational State of)	2018-2021	0.09 (-0.18 to 0.42)	0.361
Prevalence	Bosnia and Herzegovina	1990-1995	25.47 (18.36 to 33.23)	<0.001
Prevalence	Bosnia and Herzegovina	1995-2014	-6.41 (-7.37 to -5.64)	<0.001
Prevalence	Bosnia and Herzegovina	2014-2021	-1.55 (-3.08 to 2.18)	0.181
Prevalence	Botswana	1990-2004	1.1 (1.08 to 1.12)	<0.001
Prevalence	Botswana	2004-2010	0.51 (0.46 to 0.58)	<0.001
Prevalence	Botswana	2010-2016	-0.76 (-0.84 to -0.72)	0.012
Prevalence	Botswana	2016-2019	-0.18 (-0.5 to -0.08)	0.001
Prevalence	Botswana	2019-2021	0.78 (0.55 to 0.98)	<0.001
Prevalence	Brazil	1990-1994	2.81 (2.52 to 3.17)	<0.001
Prevalence	Brazil	1994-1998	0.82 (0.46 to 1.3)	0.002
Prevalence	Brazil	1998-2004	-0.36 (-0.63 to -0.12)	0.007
Prevalence	Brazil	2004-2011	-1.29 (-1.52 to -1.14)	<0.001
Prevalence	Brazil	2011-2016	-3.53 (-3.78 to -3.3)	0.000
Prevalence	Brazil	2016-2021	-0.5 (-0.77 to -0.26)	0.018
Prevalence	Brunei Darussalam	1990-1994	-0.46 (-0.66 to -0.31)	<0.001

Prevalence	Brunei Darussalam	1994-2000	0.48<br>(0.39 to 0.69)	0.000
Prevalence	Brunei Darussalam	2000-2008	0.2<br>(0.1 to 0.28)	0.009
Prevalence	Brunei Darussalam	2008-2011	-0.38<br>(-0.57 to -0.17)	0.004
Prevalence	Brunei Darussalam	2011-2015	-2.36<br>(-2.51 to -2.24)	<0.001
Prevalence	Brunei Darussalam	2015-2021	-0.7<br>(-0.79 to -0.61)	<0.001
Prevalence	Bulgaria	1990-1995	-0.04<br>(-0.15 to 0.13)	0.476
Prevalence	Bulgaria	1995-2005	-0.45<br>(-0.5 to -0.41)	<0.001
Prevalence	Bulgaria	2005-2010	-1.63<br>(-1.72 to -1.51)	<0.001
Prevalence	Bulgaria	2010-2014	-2.79<br>(-3.01 to -2.62)	<0.001
Prevalence	Bulgaria	2014-2018	-1.7<br>(-1.84 to -1.46)	<0.001
Prevalence	Bulgaria	2018-2021	0.55<br>(0.34 to 0.73)	<0.001
Prevalence	Burkina Faso	1990-1999	-0.3<br>(-0.48 to -0.19)	0.000
Prevalence	Burkina Faso	1999-2015	0.19<br>(0.13 to 0.25)	0.001
Prevalence	Burkina Faso	2015-2018	1.28<br>(0.8 to 1.56)	<0.001
Prevalence	Burkina Faso	2018-2021	7.21<br>(6.66 to 7.73)	<0.001
Prevalence	Burundi	1990-1993	4.28<br>(2.7 to 5.83)	<0.001
Prevalence	Burundi	1993-1996	110.26<br>(101.4 to 116.86)	<0.001
Prevalence	Burundi	1996-2005	5.27<br>(2.78 to 7.98)	0.002
Prevalence	Burundi	2005-2018	-16.17<br>(-18.02 to -15.23)	0.001

Prevalence	Burundi	2018-2021	-8.2<br>(-12.06 to -2.77)	0.004
Prevalence	Cabo Verde	1990-1994	-0.87<br>(-1.02 to -0.73)	<0.001
Prevalence	Cabo Verde	1994-2002	0.53<br>(0.46 to 0.6)	0.002
Prevalence	Cabo Verde	2002-2010	-0.04<br>(-0.11 to 0.04)	0.256
Prevalence	Cabo Verde	2010-2015	-1.62<br>(-1.79 to -1.49)	0.006
Prevalence	Cabo Verde	2015-2019	-0.2<br>(-0.71 to -0.04)	0.036
Prevalence	Cabo Verde	2019-2021	1<br>(0.45 to 1.31)	0.002
Prevalence	Cambodia	1990-1998	-7.56<br>(-7.96 to -6.75)	<0.001
Prevalence	Cambodia	1998-2001	-10.75<br>(-11.37 to -9.63)	<0.001
Prevalence	Cambodia	2001-2005	-5.03<br>(-5.69 to -4.55)	<0.001
Prevalence	Cambodia	2005-2009	-3.25<br>(-3.8 to -2.76)	<0.001
Prevalence	Cambodia	2009-2017	-2.42<br>(-2.54 to -2.22)	<0.001
Prevalence	Cambodia	2017-2021	-0.47<br>(-0.76 to -0.09)	0.041
Prevalence	Cameroon	1990-2013	-0.4<br>(-0.49 to -0.33)	<0.001
Prevalence	Cameroon	2013-2021	3.64<br>(3.15 to 4.37)	<0.001
Prevalence	Canada	1990-1994	-1.66<br>(-1.81 to -1.51)	<0.001
Prevalence	Canada	1994-2001	-0.27<br>(-0.36 to -0.16)	0.004
Prevalence	Canada	2001-2010	-0.83<br>(-0.9 to -0.77)	0.000
Prevalence	Canada	2010-2015	-2.74<br>(-2.91 to -2.63)	<0.001

Prevalence	Canada	2015-2018	-1.64<br>(-2.18 to -1.07)	<0.001
Prevalence	Canada	2018-2021	0.18<br>(-0.1 to 0.61)	0.160
Prevalence	Central African Republic	1990-1996	0.31<br>(-1.19 to 1)	0.474
Prevalence	Central African Republic	1996-2012	2.21<br>(1.95 to 2.55)	<0.001
Prevalence	Central African Republic	2012-2015	23.63<br>(16.49 to 27)	<0.001
Prevalence	Central African Republic	2015-2021	-1.83<br>(-5.54 to 0.38)	0.107
Prevalence	Chad	1990-1998	-6.55<br>(-8.18 to -5.52)	0.006
Prevalence	Chad	1998-2005	-2.61<br>(-3.87 to -1.61)	0.002
Prevalence	Chad	2005-2008	7.99<br>(-1.89 to 9.26)	0.065
Prevalence	Chad	2008-2013	-5.42<br>(-6.88 to 7.18)	0.065
Prevalence	Chad	2013-2019	-1.15<br>(-4.88 to -0.67)	0.002
Prevalence	Chad	2019-2021	1.99<br>(-0.4 to 3.33)	0.100
Prevalence	Chile	1990-1995	0.89<br>(0.66 to 1.12)	0.001
Prevalence	Chile	1995-2000	4.34<br>(4.07 to 4.71)	<0.001
Prevalence	Chile	2000-2004	2.91<br>(2.11 to 3.37)	<0.001
Prevalence	Chile	2004-2010	0.57<br>(0.27 to 0.8)	0.020
Prevalence	Chile	2010-2019	-2.62<br>(-2.76 to -2.51)	<0.001
Prevalence	Chile	2019-2021	1<br>(0.07 to 1.59)	0.040
Prevalence	China	1990-1993	-1.67<br>(-2.7 to -0.89)	<0.001

Prevalence	China	1993-2001	-0.28<br>(-0.46 to 0.12)	0.100
Prevalence	China	2001-2004	-7.32<br>(-7.79 to -6.8)	<0.001
Prevalence	China	2004-2007	-4.31<br>(-4.71 to -2.53)	0.001
Prevalence	China	2007-2018	-0.55<br>(-0.69 to -0.41)	0.003
Prevalence	China	2018-2021	3.26<br>(2.36 to 5.08)	<0.001
Prevalence	Colombia	1990-1995	-1.85<br>(-2.7 to -1.34)	<0.001
Prevalence	Colombia	1995-2002	0.23<br>(-0.05 to 0.72)	0.088
Prevalence	Colombia	2002-2006	-4.06<br>(-4.88 to -3.34)	<0.001
Prevalence	Colombia	2006-2011	-1.64<br>(-2.02 to -0.77)	<0.001
Prevalence	Colombia	2011-2017	-3.15<br>(-3.85 to -2.85)	<0.001
Prevalence	Colombia	2017-2021	-0.94<br>(-1.49 to -0.07)	0.041
Prevalence	Comoros	1990-1995	-1.32<br>(-2.29 to -0.74)	<0.001
Prevalence	Comoros	1995-1998	3.98<br>(2.21 to 4.95)	<0.001
Prevalence	Comoros	1998-2004	-1.85<br>(-3.08 to -1.34)	<0.001
Prevalence	Comoros	2004-2009	0.61<br>(-0.08 to 1.98)	0.087
Prevalence	Comoros	2009-2016	-1.4<br>(-2.08 to -1.03)	<0.001
Prevalence	Comoros	2016-2021	5.66<br>(4.69 to 6.56)	<0.001
Prevalence	Congo	1990-1996	1.21<br>(0.04 to 2.36)	0.046
Prevalence	Congo	1996-1999	102.91<br>(76.25 to 121.75)	0.014

Prevalence	Congo	1999-2002	-25.42 (-33.07 to -11.36)	0.029
Prevalence	Congo	2002-2016	-9.88 (-10.97 to -7.5)	0.010
Prevalence	Congo	2016-2021	-0.1 (-1.95 to 2.46)	0.863
Prevalence	Cook Islands	1990-1996	-0.67 (-2.16 to 0.51)	0.205
Prevalence	Cook Islands	1996-1999	25.05 (16.63 to 28.12)	<0.001
Prevalence	Cook Islands	1999-2014	-3.14 (-3.85 to -2.29)	0.001
Prevalence	Cook Islands	2014-2017	-14.63 (-16.32 to -10.39)	<0.001
Prevalence	Cook Islands	2017-2021	-0.76 (-2.34 to 1.84)	0.336
Prevalence	Costa Rica	1990-2001	1.13 (1.06 to 1.22)	<0.001
Prevalence	Costa Rica	2001-2008	0.41 (0.29 to 0.78)	0.006
Prevalence	Costa Rica	2008-2011	-0.43 (-1.06 to 0.31)	0.084
Prevalence	Costa Rica	2011-2015	-3.03 (-3.44 to -2.5)	0.002
Prevalence	Costa Rica	2015-2018	-1.67 (-2.66 to -0.87)	<0.001
Prevalence	Costa Rica	2018-2021	0.2 (-0.24 to 0.87)	0.161
Prevalence	Coted'Ivoire	1990-2001	0.41 (0.31 to 0.51)	<0.001
Prevalence	Coted'Ivoire	2001-2004	4.33 (3.84 to 4.72)	<0.001
Prevalence	Coted'Ivoire	2004-2009	-1.8 (-2.1 to -1.51)	<0.001
Prevalence	Coted'Ivoire	2009-2012	2.7 (2.14 to 3.17)	<0.001
Prevalence	Coted'Ivoire	2012-2016	-2.86 (-3.27 to -2.5)	<0.001

Prevalence	Coted'Ivoire	2016-2021	0.63<br>(0.31 to 0.92)	0.000
Prevalence	Croatia	1990-1992	24.72<br>(22.7 to 26.86)	<0.001
Prevalence	Croatia	1992-1995	-0.5<br>(-2.37 to 0.68)	0.308
Prevalence	Croatia	1995-1998	-3.75<br>(-4.54 to -0.9)	0.000
Prevalence	Croatia	1998-2010	-1.51<br>(-4 to -1.2)	0.001
Prevalence	Croatia	2010-2018	-3.78<br>(-4.26 to -3.42)	<0.001
Prevalence	Croatia	2018-2021	0.13<br>(-0.91 to 1.63)	0.716
Prevalence	Cuba	1990-1996	-1.2<br>(-1.44 to -0.96)	<0.001
Prevalence	Cuba	1996-2009	1.56<br>(1.48 to 1.65)	<0.001
Prevalence	Cuba	2009-2018	-2.08<br>(-2.23 to -1.95)	<0.001
Prevalence	Cuba	2018-2021	0.25<br>(-0.53 to 1.44)	0.415
Prevalence	Cyprus	1990-1993	0.4<br>(-0.15 to 0.82)	0.139
Prevalence	Cyprus	1993-1999	1.18<br>(0.94 to 1.55)	<0.001
Prevalence	Cyprus	1999-2006	1.72<br>(1.5 to 2.04)	0.003
Prevalence	Cyprus	2006-2010	0.3<br>(-0.11 to 0.57)	0.068
Prevalence	Cyprus	2010-2018	-3.77<br>(-3.87 to -3.67)	<0.001
Prevalence	Cyprus	2018-2021	-0.41<br>(-0.75 to -0.13)	0.007
Prevalence	Czechia	1990-2000	-0.12<br>(-0.21 to -0.03)	0.010
Prevalence	Czechia	2000-2004	2.52<br>(2.23 to 2.78)	<0.001

Prevalence	Czechia	2004-2008	-0.39 (-0.75 to -0.03)	0.044
Prevalence	Czechia	2008-2015	-2.47 (-2.63 to -2.29)	<0.001
Prevalence	Czechia	2015-2019	-3.69 (-4.11 to -3.37)	<0.001
Prevalence	Czechia	2019-2021	1.6 (0.93 to 2.19)	<0.001
Prevalence	Democratic People's Republic of Korea	1990-1994	-4.79 (-5.09 to -4.49)	<0.001
Prevalence	Democratic People's Republic of Korea	1994-1999	-1.73 (-2.03 to -1.43)	<0.001
Prevalence	Democratic People's Republic of Korea	1999-2011	0.06 (-0.02 to 0.15)	0.124
Prevalence	Democratic People's Republic of Korea	2011-2016	-2.42 (-2.8 to -2.14)	<0.001
Prevalence	Democratic People's Republic of Korea	2016-2021	-0.4 (-0.74 to 0.02)	0.056
Prevalence	Democratic Republic of the Congo	1990-1995	-0.08 (-0.8 to 0.62)	0.705
Prevalence	Democratic Republic of the Congo	1995-1998	36.4 (30.97 to 39.73)	0.010
Prevalence	Democratic Republic of the Congo	1998-2001	-9.33 (-11.79 to -4.61)	0.026
Prevalence	Democratic Republic of the Congo	2001-2016	-4.08 (-4.46 to -2.93)	0.008
Prevalence	Democratic Republic of the Congo	2016-2021	1.93 (0.55 to 4.28)	0.012
Prevalence	Denmark	1990-1998	-1.12 (-1.23 to -1.04)	<0.001
Prevalence	Denmark	1998-2001	0.42 (-0.42 to 1.28)	0.090
Prevalence	Denmark	2001-2009	1.78 (1.69 to 1.93)	0.001
Prevalence	Denmark	2009-2012	-0.53 (-0.91 to -0.2)	0.026
Prevalence	Denmark	2012-2018	-2.47 (-2.63 to -2.35)	<0.001

Prevalence	Denmark	2018-2021	-0.09 (-0.42 to 0.2)	0.525
Prevalence	Djibouti	1990-1993	14.47 (9.54 to 16.13)	<0.001
Prevalence	Djibouti	1993-2015	-2.35 (-2.51 to -2.18)	<0.001
Prevalence	Djibouti	2015-2021	-0.34 (-0.89 to 0.66)	0.291
Prevalence	Dominica	1990-1994	-0.67 (-1.6 to -0.09)	0.027
Prevalence	Dominica	1994-2011	1.57 (1.5 to 1.66)	<0.001
Prevalence	Dominica	2011-2014	-2.82 (-3.31 to -1.81)	<0.001
Prevalence	Dominica	2014-2018	9.08 (8.11 to 9.97)	<0.001
Prevalence	Dominica	2018-2021	1.66 (-0.89 to 3.8)	0.103
Prevalence	Dominican Republic	1990-1995	0.04 (-0.5 to 0.37)	0.817
Prevalence	Dominican Republic	1995-2010	1.28 (0.74 to 1.52)	0.016
Prevalence	Dominican Republic	2010-2015	-3.19 (-3.57 to 1.37)	0.244
Prevalence	Dominican Republic	2015-2019	-1.4 (-3.35 to -1.06)	<0.001
Prevalence	Dominican Republic	2019-2021	0.07 (-1.08 to 0.77)	0.954
Prevalence	Ecuador	1990-2004	-0.56 (-0.72 to -0.35)	0.008
Prevalence	Ecuador	2004-2007	-3.96 (-4.59 to -2.59)	0.011
Prevalence	Ecuador	2007-2011	-0.89 (-1.51 to 0.2)	0.112
Prevalence	Ecuador	2011-2015	-2.86 (-3.85 to -2.11)	0.002
Prevalence	Ecuador	2015-2021	-0.69 (-1.08 to 0.14)	0.077

Prevalence	Egypt	1990-1997	-1.09<br>(-1.16 to -1.03)	<0.001
Prevalence	Egypt	1997-2008	-1.72<br>(-1.76 to -1.68)	<0.001
Prevalence	Egypt	2008-2013	-2.46<br>(-2.65 to -1.76)	<0.001
Prevalence	Egypt	2013-2016	-2.07<br>(-2.3 to -1.94)	<0.001
Prevalence	Egypt	2016-2019	0.08<br>(-0.1 to 0.26)	0.302
Prevalence	Egypt	2019-2021	1.46<br>(1.15 to 1.74)	<0.001
Prevalence	El Salvador	1990-1997	-13.2<br>(-14.75 to -12.4)	<0.001
Prevalence	El Salvador	1997-2001	-8.61<br>(-10.11 to -6.85)	<0.001
Prevalence	El Salvador	2001-2005	-3.85<br>(-4.73 to -2.87)	<0.001
Prevalence	El Salvador	2005-2011	-0.47<br>(-0.81 to 0.07)	0.059
Prevalence	El Salvador	2011-2017	-2.86<br>(-3.32 to -2.55)	<0.001
Prevalence	El Salvador	2017-2021	0.7<br>(0.2 to 1.32)	0.018
Prevalence	Equatorial Guinea	1990-1996	-0.29<br>(-0.49 to -0.19)	<0.001
Prevalence	Equatorial Guinea	1996-1999	0.61<br>(0.23 to 0.81)	<0.001
Prevalence	Equatorial Guinea	1999-2003	-0.93<br>(-1.24 to -0.72)	<0.001
Prevalence	Equatorial Guinea	2003-2010	-0.21<br>(-0.31 to -0.02)	0.044
Prevalence	Equatorial Guinea	2010-2016	-1.79<br>(-1.91 to -1.67)	<0.001
Prevalence	Equatorial Guinea	2016-2021	0.53<br>(0.36 to 0.69)	<0.001
Prevalence	Eritrea	1990-1998	-13.92<br>(-15.86 to -12.87)	<0.001

Prevalence	Eritrea	1998-2001	0.58<br>(-6.5 to 3.95)	0.949
Prevalence	Eritrea	2001-2004	-21.88<br>(-25.06 to -15.59)	0.003
Prevalence	Eritrea	2004-2014	-10.38<br>(-11.86 to -8.48)	<0.001
Prevalence	Eritrea	2014-2019	-3.92<br>(-5.49 to -2.94)	<0.001
Prevalence	Eritrea	2019-2021	-0.1<br>(-1.3 to 0.77)	0.695
Prevalence	Estonia	1990-1996	0.17<br>(0.04 to 0.32)	0.008
Prevalence	Estonia	1996-2001	-0.73<br>(-0.95 to -0.53)	<0.001
Prevalence	Estonia	2001-2008	-1.88<br>(-1.98 to -1.78)	<0.001
Prevalence	Estonia	2008-2014	-4.15<br>(-4.26 to -4.05)	<0.001
Prevalence	Estonia	2014-2017	-1.87<br>(-2.13 to -1.6)	<0.001
Prevalence	Estonia	2017-2021	0.49<br>(0.27 to 0.72)	<0.001
Prevalence	Eswatini	1990-1997	-1.28<br>(-1.89 to -1.05)	0.007
Prevalence	Eswatini	1997-2004	-0.59<br>(-1.08 to -0.18)	0.007
Prevalence	Eswatini	2004-2011	0.12<br>(-0.62 to 0.76)	0.206
Prevalence	Eswatini	2011-2014	-1.13<br>(-1.5 to 0.24)	0.101
Prevalence	Eswatini	2014-2019	-0.18<br>(-0.69 to 0.36)	0.337
Prevalence	Eswatini	2019-2021	3.68<br>(2.43 to 4.43)	<0.001
Prevalence	Ethiopia	1990-1998	-6.85<br>(-9.23 to -4.73)	0.019
Prevalence	Ethiopia	1998-2001	18.29<br>(-8.27 to 23.02)	0.147

Prevalence	Ethiopia	2001-2004	-13.52 (-17.17 to 13.52)	0.147
Prevalence	Ethiopia	2004-2015	-5.26 (-8.59 to -3.59)	0.006
Prevalence	Ethiopia	2015-2019	-0.91 (-4.66 to 0.57)	0.141
Prevalence	Ethiopia	2019-2021	5.72 (2.04 to 8.11)	0.001
Prevalence	Fiji	1990-2002	1.21 (1.08 to 1.34)	<0.001
Prevalence	Fiji	2002-2011	-0.79 (-1.03 to -0.54)	0.000
Prevalence	Fiji	2011-2015	-2.97 (-3.81 to -2.37)	<0.001
Prevalence	Fiji	2015-2021	0.56 (0.22 to 0.98)	0.004
Prevalence	Finland	1990-1995	2.18 (1.43 to 2.49)	<0.001
Prevalence	Finland	1995-2004	2.78 (2.65 to 3.08)	0.018
Prevalence	Finland	2004-2010	-0.58 (-0.78 to -0.42)	0.000
Prevalence	Finland	2010-2018	-4.69 (-4.83 to -4.56)	<0.001
Prevalence	Finland	2018-2021	0.56 (0.06 to 0.98)	0.030
Prevalence	France	1990-1994	-0.98 (-1.39 to -0.74)	<0.001
Prevalence	France	1994-2000	0.02 (-0.25 to 0.23)	0.783
Prevalence	France	2000-2006	0.64 (0.48 to 1.02)	0.002
Prevalence	France	2006-2010	-0.05 (-0.71 to 0.16)	0.555
Prevalence	France	2010-2017	-1.93 (-2.07 to -1.82)	<0.001
Prevalence	France	2017-2021	-0.01 (-0.26 to 0.24)	0.950

Prevalence	Gabon	1990-1994	-1.4 (-1.67 to -1.24)	<0.001
Prevalence	Gabon	1994-2003	-0.31 (-0.39 to -0.24)	<0.001
Prevalence	Gabon	2003-2010	0.08 (0 to 0.2)	0.054
Prevalence	Gabon	2010-2015	-1.81 (-1.93 to -1.71)	0.000
Prevalence	Gabon	2015-2019	-0.21 (-0.49 to -0.06)	0.017
Prevalence	Gabon	2019-2021	1.26 (0.76 to 1.6)	<0.001
Prevalence	Gambia	1990-1995	-3.25 (-4.23 to -2.83)	<0.001
Prevalence	Gambia	1995-1998	-1.18 (-2.37 to 0.34)	0.070
Prevalence	Gambia	1998-2001	1.1 (-0.21 to 1.47)	0.057
Prevalence	Gambia	2001-2011	-0.3 (-0.48 to -0.19)	0.012
Prevalence	Gambia	2011-2015	-2.07 (-2.63 to -1.56)	0.011
Prevalence	Gambia	2015-2021	-0.09 (-0.35 to 0.33)	0.378
Prevalence	Georgia	1990-1994	7.73 (5.62 to 10.06)	<0.001
Prevalence	Georgia	1994-1998	-3.9 (-6.13 to -1.57)	0.006
Prevalence	Georgia	1998-2010	-0.41 (-0.8 to 0.85)	0.276
Prevalence	Georgia	2010-2015	-3.96 (-6.07 to -2.5)	0.015
Prevalence	Georgia	2015-2021	-1.34 (-2.33 to 1.26)	0.183
Prevalence	Germany	1990-1992	-0.32 (-0.59 to 0.2)	0.200
Prevalence	Germany	1992-2008	0.83 (0.8 to 0.87)	<0.001

Prevalence	Germany	2008-2011	-0.71<br>(-1.06 to -0.31)	0.013
Prevalence	Germany	2011-2018	-2.49<br>(-2.61 to -2.4)	<0.001
Prevalence	Germany	2018-2021	-0.52<br>(-0.8 to -0.28)	0.012
Prevalence	Ghana	1990-1994	0.09<br>(-0.24 to 0.31)	0.358
Prevalence	Ghana	1994-2002	1.07<br>(0.97 to 1.2)	<0.001
Prevalence	Ghana	2002-2011	0.2<br>(0.12 to 0.27)	0.001
Prevalence	Ghana	2011-2015	-2.41<br>(-2.56 to -2.26)	0.003
Prevalence	Ghana	2015-2019	-0.14<br>(-0.47 to 0.02)	0.082
Prevalence	Ghana	2019-2021	1.33<br>(0.71 to 1.78)	<0.001
Prevalence	Greece	1990-2000	-0.33<br>(-0.39 to -0.26)	0.009
Prevalence	Greece	2000-2005	-1.2<br>(-1.47 to -0.45)	0.002
Prevalence	Greece	2005-2010	-0.82<br>(-1.01 to -0.58)	<0.001
Prevalence	Greece	2010-2015	-2.77<br>(-3 to -2.64)	<0.001
Prevalence	Greece	2015-2018	-1.28<br>(-1.86 to -0.82)	<0.001
Prevalence	Greece	2018-2021	0.45<br>(0.15 to 0.93)	0.012
Prevalence	Greenland	1990-2001	-0.94<br>(-1 to -0.82)	0.010
Prevalence	Greenland	2001-2006	-1.47<br>(-1.92 to -1.18)	<0.001
Prevalence	Greenland	2006-2010	-3.48<br>(-4.08 to -3.1)	<0.001
Prevalence	Greenland	2010-2013	-0.86<br>(-1.35 to -0.45)	<0.001

Prevalence	Greenland	2013-2019	-1.99 (-2.72 to -1.84)	<0.001
Prevalence	Greenland	2019-2021	0.2 (-0.98 to 0.81)	0.575
Prevalence	Grenada	1990-1998	-4.37 (-5.14 to -3.81)	0.007
Prevalence	Grenada	1998-2003	-0.77 (-2.18 to -0.23)	0.026
Prevalence	Grenada	2003-2006	2.35 (1.16 to 2.89)	0.036
Prevalence	Grenada	2006-2011	0.17 (-0.4 to 0.76)	0.476
Prevalence	Grenada	2011-2015	-1.94 (-2.64 to -1.34)	0.012
Prevalence	Grenada	2015-2021	-0.56 (-0.91 to 0.33)	0.119
Prevalence	Guam	1990-1995	-0.68 (-0.8 to -0.54)	0.001
Prevalence	Guam	1995-2005	1.27 (1.16 to 1.34)	0.029
Prevalence	Guam	2005-2010	0.36 (0.18 to 1.28)	0.002
Prevalence	Guam	2010-2015	-2.8 (-2.99 to 0.35)	0.101
Prevalence	Guam	2015-2018	-1.03 (-2.85 to -0.82)	<0.001
Prevalence	Guam	2018-2021	-0.3 (-0.7 to 0.18)	0.178
Prevalence	Guatemala	1990-1997	-5.93 (-6.5 to -5.52)	<0.001
Prevalence	Guatemala	1997-2002	-3.48 (-4.27 to -3.06)	<0.001
Prevalence	Guatemala	2002-2005	-1.07 (-3 to -0.36)	0.004
Prevalence	Guatemala	2005-2011	0.07 (-0.19 to 0.49)	0.360
Prevalence	Guatemala	2011-2017	-2.17 (-2.38 to -1.97)	0.001

Prevalence	Guatemala	2017-2021	1.76<br>(1.47 to 2.04)	<0.001
Prevalence	Guinea	1990-1998	-0.52<br>(-1 to -0.14)	0.020
Prevalence	Guinea	1998-2002	7.38<br>(5.72 to 9.44)	0.020
Prevalence	Guinea	2002-2005	-3.92<br>(-5.24 to -0.97)	0.038
Prevalence	Guinea	2005-2010	0.34<br>(-0.52 to 2.56)	0.259
Prevalence	Guinea	2010-2015	-2.23<br>(-4.02 to -1.32)	0.009
Prevalence	Guinea	2015-2021	0.29<br>(-0.38 to 2.22)	0.264
Prevalence	Guinea-Bissau	1990-1997	0.33<br>(-0.15 to 0.78)	0.138
Prevalence	Guinea-Bissau	1997-2000	19.63<br>(16.59 to 22.11)	0.012
Prevalence	Guinea-Bissau	2000-2003	-10.31<br>(-12.59 to -6.92)	0.032
Prevalence	Guinea-Bissau	2003-2007	-1.23<br>(-2.93 to 0.92)	0.252
Prevalence	Guinea-Bissau	2007-2018	-3.5<br>(-4.56 to -3.22)	<0.001
Prevalence	Guinea-Bissau	2018-2021	0.47<br>(-1.06 to 2.85)	0.490
Prevalence	Guyana	1990-2001	-0.21<br>(-0.34 to -0.1)	0.003
Prevalence	Guyana	2001-2006	1.95<br>(1.24 to 2.48)	0.003
Prevalence	Guyana	2006-2009	4.61<br>(4.04 to 5.04)	0.001
Prevalence	Guyana	2009-2019	-2.3<br>(-2.51 to -2.18)	0.004
Prevalence	Guyana	2019-2021	-0.1<br>(-1.49 to 0.53)	0.594
Prevalence	Haiti	1990-2009	0.49<br>(0.02 to 0.92)	0.043

Prevalence	Haiti	2009-2012	116.16 (72.93 to 139.9)	<0.001
Prevalence	Haiti	2012-2021	-9.65 (-17.14 to -4.31)	0.001
Prevalence	Honduras	1990-1997	0.99 (0.31 to 1.61)	0.006
Prevalence	Honduras	1997-2000	19.55 (13.74 to 21.45)	<0.001
Prevalence	Honduras	2000-2015	-2.45 (-2.89 to -1.87)	<0.001
Prevalence	Honduras	2015-2018	-10.65 (-12.01 to -7.59)	<0.001
Prevalence	Honduras	2018-2021	0.25 (-1.6 to 3.31)	0.695
Prevalence	Hungary	1990-2010	0.39 (0.35 to 0.42)	<0.001
Prevalence	Hungary	2010-2018	-3.02 (-3.16 to -2.91)	<0.001
Prevalence	Hungary	2018-2021	0.15 (-0.47 to 0.7)	0.477
Prevalence	Iceland	1990-1994	-0.16 (-0.81 to 0.17)	0.294
Prevalence	Iceland	1994-1997	1.92 (1.2 to 2.33)	0.001
Prevalence	Iceland	1997-2002	-0.24 (-0.86 to 0.06)	0.106
Prevalence	Iceland	2002-2010	0.47 (0.33 to 0.82)	0.012
Prevalence	Iceland	2010-2017	-3.05 (-3.27 to -2.87)	<0.001
Prevalence	Iceland	2017-2021	-0.04 (-0.5 to 0.41)	0.855
Prevalence	India	1990-1998	0.07 (-0.35 to 0.23)	0.513
Prevalence	India	1998-2002	0.7 (-0.15 to 1.08)	0.061
Prevalence	India	2002-2010	-0.99 (-1.21 to -0.51)	0.040

Prevalence	India	2010-2015	-3.24 (-3.68 to -1.02)	<0.001
Prevalence	India	2015-2018	-1.59 (-3.15 to -0.82)	<0.001
Prevalence	India	2018-2021	0.15 (-0.45 to 1.01)	0.481
Prevalence	Indonesia	1990-2003	-2.07 (-2.31 to -1.83)	<0.001
Prevalence	Indonesia	2003-2006	12.2 (10.7 to 13.21)	<0.001
Prevalence	Indonesia	2006-2021	-2.85 (-3.09 to -2.61)	<0.001
Prevalence	Iran (Islamic Republic of)	1990-2005	-0.83 (-0.93 to -0.7)	0.013
Prevalence	Iran (Islamic Republic of)	2005-2009	-4.53 (-5.02 to -2.66)	0.000
Prevalence	Iran (Islamic Republic of)	2009-2015	-6.7 (-7.55 to -6.25)	<0.001
Prevalence	Iran (Islamic Republic of)	2015-2018	-4.21 (-6.2 to -2.31)	<0.001
Prevalence	Iran (Islamic Republic of)	2018-2021	0.02 (-0.95 to 1.71)	0.921
Prevalence	Iraq	1990-1992	-21.33 (-23.99 to -16.58)	<0.001
Prevalence	Iraq	1992-2002	-3.46 (-3.77 to -3.09)	<0.001
Prevalence	Iraq	2002-2008	20.82 (19.45 to 21.87)	<0.001
Prevalence	Iraq	2008-2012	-8.73 (-13.46 to -4.22)	0.000
Prevalence	Iraq	2012-2017	8.73 (5.38 to 15.74)	<0.001
Prevalence	Iraq	2017-2021	-11.63 (-15.57 to -7.84)	<0.001
Prevalence	Ireland	1990-1997	0.37 (0.33 to 0.41)	<0.001
Prevalence	Ireland	1997-2005	-0.38 (-0.41 to -0.34)	0.002

Prevalence	Ireland	2005-2010	-0.65 (-0.75 to -0.57)	<0.001
Prevalence	Ireland	2010-2014	-1.65 (-1.8 to -1.56)	<0.001
Prevalence	Ireland	2014-2018	-1.08 (-1.17 to -0.91)	<0.001
Prevalence	Ireland	2018-2021	0.58 (0.43 to 0.71)	<0.001
Prevalence	Israel	1990-2000	-0.39 (-0.69 to -0.15)	0.009
Prevalence	Israel	2000-2003	4 (2.17 to 4.78)	0.009
Prevalence	Israel	2003-2009	0.17 (-0.88 to 0.86)	0.731
Prevalence	Israel	2009-2021	-2.34 (-2.62 to -2.12)	0.000
Prevalence	Italy	1990-1997	-1.8 (-1.91 to -1.73)	<0.001
Prevalence	Italy	1997-2004	-1.41 (-1.55 to -1.3)	<0.001
Prevalence	Italy	2004-2010	-1.07 (-1.16 to -0.9)	0.000
Prevalence	Italy	2010-2015	2.2 (2.1 to 2.3)	<0.001
Prevalence	Italy	2015-2019	-0.49 (-0.67 to -0.36)	<0.001
Prevalence	Italy	2019-2021	0.96 (0.51 to 1.28)	<0.001
Prevalence	Jamaica	1990-1996	-1.09 (-1.56 to -0.85)	0.001
Prevalence	Jamaica	1996-2003	0.19 (-0.31 to 0.49)	0.235
Prevalence	Jamaica	2003-2010	0.98 (0.78 to 1.41)	0.004
Prevalence	Jamaica	2010-2017	-2.22 (-2.4 to -2.06)	0.000
Prevalence	Jamaica	2017-2021	4.2 (3.72 to 4.58)	<0.001

Prevalence	Japan	1990-2003	-1.16 (-1.22 to -1.11)	<0.001
Prevalence	Japan	2003-2011	-0.59 (-1.05 to -0.46)	0.000
Prevalence	Japan	2011-2015	-3.57 (-3.84 to -0.65)	<0.001
Prevalence	Japan	2015-2018	-2.84 (-3.34 to -2.33)	<0.001
Prevalence	Japan	2018-2021	-0.27 (-0.65 to 0.17)	0.137
Prevalence	Jordan	1990-1999	0.04 (-0.04 to 0.16)	0.294
Prevalence	Jordan	1999-2007	-0.34 (-0.52 to -0.24)	<0.001
Prevalence	Jordan	2007-2015	-1.48 (-1.61 to -1.37)	0.001
Prevalence	Jordan	2015-2021	0.51 (0.34 to 0.66)	0.000
Prevalence	Kazakhstan	1990-1998	-0.3 (-0.49 to -0.17)	0.003
Prevalence	Kazakhstan	1998-2004	0.57 (0.38 to 0.89)	0.006
Prevalence	Kazakhstan	2004-2009	-2.13 (-2.38 to -1.83)	0.008
Prevalence	Kazakhstan	2009-2015	-4.27 (-4.56 to -4.05)	<0.001
Prevalence	Kazakhstan	2015-2018	-1.85 (-3.19 to -0.79)	<0.001
Prevalence	Kazakhstan	2018-2021	0.65 (0.09 to 1.68)	0.026
Prevalence	Kenya	1990-1996	0.38 (-0.28 to 0.61)	0.114
Prevalence	Kenya	1996-2001	1.28 (0.96 to 1.88)	0.005
Prevalence	Kenya	2001-2006	-0.43 (-1.06 to -0.14)	0.011
Prevalence	Kenya	2006-2009	2.38 (1.54 to 2.82)	0.005

Prevalence	Kenya	2009-2014	-2.53 (-3.01 to -2.23)	0.003
Prevalence	Kenya	2014-2021	-0.13 (-0.33 to 0.15)	0.174
Prevalence	Kiribati	1990-1993	3.02 (0.06 to 4.67)	0.045
Prevalence	Kiribati	1993-1996	9.56 (5.7 to 11.47)	0.034
Prevalence	Kiribati	1996-2000	-4.75 (-6.86 to -2.29)	0.006
Prevalence	Kiribati	2000-2011	-1.54 (-1.85 to -0.26)	0.036
Prevalence	Kiribati	2011-2016	-3.39 (-4.93 to -2.51)	0.007
Prevalence	Kiribati	2016-2021	-0.29 (-1.12 to 1.62)	0.503
Prevalence	Kuwait	1990-1992	42.07 (37.39 to 47.21)	<0.001
Prevalence	Kuwait	1992-1996	-12.5 (-14.71 to -10.59)	<0.001
Prevalence	Kuwait	1996-2001	-2.59 (-3.68 to -0.22)	0.036
Prevalence	Kuwait	2001-2006	-5.44 (-6.85 to -4.57)	<0.001
Prevalence	Kuwait	2006-2018	-1.69 (-1.89 to -1.54)	<0.001
Prevalence	Kuwait	2018-2021	1.1 (-0.02 to 2.92)	0.052
Prevalence	Kyrgyzstan	1990-2005	-0.98 (-1.1 to -0.72)	0.020
Prevalence	Kyrgyzstan	2005-2011	-1.73 (-2.96 to -1.24)	<0.001
Prevalence	Kyrgyzstan	2011-2016	-4.84 (-5.88 to -4.19)	0.004
Prevalence	Kyrgyzstan	2016-2021	0.06 (-0.54 to 0.91)	0.792
Prevalence	Lao People's Democratic Republic	1990-1999	-1.7 (-2.05 to -1.43)	<0.001

Prevalence	Lao People's Democratic Republic	1999-2009	-0.43 (-0.65 to -0.09)	0.022
Prevalence	Lao People's Democratic Republic	2009-2017	-2.52 (-3 to -2.22)	<0.001
Prevalence	Lao People's Democratic Republic	2017-2021	0.51 (-0.31 to 1.77)	0.202
Prevalence	Latvia	1990-2000	0.55 (0.45 to 0.64)	0.004
Prevalence	Latvia	2000-2007	-1.77 (-1.89 to -1.03)	0.037
Prevalence	Latvia	2007-2010	-2.91 (-3.94 to -2)	<0.001
Prevalence	Latvia	2010-2015	-5.37 (-5.81 to -5.09)	<0.001
Prevalence	Latvia	2015-2018	-1.46 (-3.36 to -0.93)	0.011
Prevalence	Latvia	2018-2021	0.92 (0.34 to 1.89)	0.004
Prevalence	Lebanon	1990-1996	-9.46 (-12.26 to -5.29)	0.002
Prevalence	Lebanon	1996-2001	-23.3 (-28.15 to -20.83)	<0.001
Prevalence	Lebanon	2001-2005	-7.16 (-8.94 to -5.37)	<0.001
Prevalence	Lebanon	2005-2008	4.56 (2.65 to 5.93)	<0.001
Prevalence	Lebanon	2008-2012	-4.78 (-6.32 to -3.7)	<0.001
Prevalence	Lebanon	2012-2021	-1.02 (-1.37 to -0.55)	0.003
Prevalence	Lesotho	1990-1997	1.21 (1.06 to 1.33)	<0.001
Prevalence	Lesotho	1997-2000	4.73 (4.38 to 5.1)	<0.001
Prevalence	Lesotho	2000-2003	-1.64 (-2.01 to -1.2)	<0.001
Prevalence	Lesotho	2003-2010	-0.18 (-0.31 to 0.3)	0.190

Prevalence	Lesotho	2010-2017	-0.76<br>(-1.22 to -0.62)	<0.001
Prevalence	Lesotho	2017-2021	0.45<br>(0.16 to 0.83)	0.005
Prevalence	Liberia	1990-1996	19.44<br>(17.27 to 21.59)	<0.001
Prevalence	Liberia	1996-2000	-16.38<br>(-20.71 to -12.98)	<0.001
Prevalence	Liberia	2000-2004	8.49<br>(3.62 to 15.16)	<0.001
Prevalence	Liberia	2004-2008	-16.85<br>(-21.19 to -13.23)	<0.001
Prevalence	Liberia	2008-2017	-8.57<br>(-9.19 to -7.78)	<0.001
Prevalence	Liberia	2017-2021	-1.8<br>(-2.71 to -0.91)	0.008
Prevalence	Libya	1990-1994	-4.43<br>(-11.98 to 0.71)	0.104
Prevalence	Libya	1994-1997	6.96<br>(-4.95 to 10.34)	0.152
Prevalence	Libya	1997-2005	-3.89<br>(-5.95 to 16.34)	0.141
Prevalence	Libya	2005-2012	16.16<br>(3.23 to 18.17)	0.033
Prevalence	Libya	2012-2021	2.99<br>(0.41 to 5.1)	0.039
Prevalence	Lithuania	1990-1993	-0.93<br>(-1.63 to -0.57)	<0.001
Prevalence	Lithuania	1993-2002	0.45<br>(0.36 to 0.57)	<0.001
Prevalence	Lithuania	2002-2010	-0.6<br>(-0.71 to -0.48)	0.002
Prevalence	Lithuania	2010-2015	-4.53<br>(-4.79 to -4.38)	0.000
Prevalence	Lithuania	2015-2018	-2.75<br>(-3.33 to -2.31)	<0.001
Prevalence	Lithuania	2018-2021	-0.08<br>(-0.48 to 0.42)	0.660

Prevalence	Luxembourg	1990-1999	-1.04 (-1.24 to -0.94)	<0.001
Prevalence	Luxembourg	1999-2002	-0.15 (-0.85 to 0.79)	0.531
Prevalence	Luxembourg	2002-2010	0.89 (-0.15 to 1.11)	0.054
Prevalence	Luxembourg	2010-2018	-2.06 (-2.21 to -1.96)	<0.001
Prevalence	Luxembourg	2018-2021	-0.1 (-0.58 to 0.73)	0.582
Prevalence	Madagascar	1990-2000	-1.17 (-1.21 to -1.12)	<0.001
Prevalence	Madagascar	2000-2007	0.27 (0.1 to 0.33)	0.023
Prevalence	Madagascar	2007-2010	0.85 (0.57 to 1.01)	<0.001
Prevalence	Madagascar	2010-2013	-1.5 (-1.66 to -1.23)	<0.001
Prevalence	Madagascar	2013-2019	-0.44 (-0.53 to -0.29)	0.000
Prevalence	Madagascar	2019-2021	1.65 (1.2 to 1.96)	<0.001
Prevalence	Malawi	1990-1992	0.09 (-0.35 to 0.39)	0.655
Prevalence	Malawi	1992-2002	-0.74 (-0.82 to -0.7)	<0.001
Prevalence	Malawi	2002-2011	0.02 (-0.04 to 0.1)	0.315
Prevalence	Malawi	2011-2014	-1.03 (-1.18 to -0.68)	0.029
Prevalence	Malawi	2014-2019	0.68 (0.38 to 0.78)	0.038
Prevalence	Malawi	2019-2021	1.49 (1.02 to 1.79)	<0.001
Prevalence	Malaysia	1990-1995	0.52 (0.25 to 0.65)	0.006
Prevalence	Malaysia	1995-1998	1.62 (1.21 to 1.79)	<0.001

Prevalence	Malaysia	1998-2010	0.53<br>(0.48 to 0.56)	<0.001
Prevalence	Malaysia	2010-2018	-1.89<br>(-1.96 to -1.82)	<0.001
Prevalence	Malaysia	2018-2021	-0.01<br>(-0.36 to 0.34)	0.965
Prevalence	Maldives	1990-1993	-3.89<br>(-6.35 to -2.36)	<0.001
Prevalence	Maldives	1993-2003	0.5<br>(0.22 to 0.89)	0.009
Prevalence	Maldives	2003-2006	23.36<br>(18.04 to 25.32)	0.022
Prevalence	Maldives	2006-2015	-3.81<br>(-5.86 to -3.04)	0.044
Prevalence	Maldives	2015-2021	-1.7<br>(-2.8 to 1.3)	0.130
Prevalence	Mali	1990-1996	0.95<br>(0.4 to 1.47)	0.001
Prevalence	Mali	1996-1999	33.93<br>(31.08 to 36.09)	<0.001
Prevalence	Mali	1999-2012	-5.82<br>(-6.52 to -5.29)	0.000
Prevalence	Mali	2012-2017	-1.54<br>(-3.12 to 0.11)	0.060
Prevalence	Mali	2017-2021	4.27<br>(2.81 to 6.96)	<0.001
Prevalence	Malta	1990-2000	1.19<br>(1.08 to 1.29)	<0.001
Prevalence	Malta	2000-2005	3.24<br>(2.94 to 3.65)	<0.001
Prevalence	Malta	2005-2009	0.2<br>(-0.12 to 0.78)	0.179
Prevalence	Malta	2009-2012	-1.94<br>(-3 to -1.44)	<0.001
Prevalence	Malta	2012-2019	-4.88<br>(-5.04 to -4.74)	<0.001
Prevalence	Malta	2019-2021	0.89<br>(0.23 to 1.48)	0.006

Prevalence	Marshall Islands	1990-1997	1.35<br>(1.29 to 1.44)	<0.001
Prevalence	Marshall Islands	1997-2000	0.66<br>(0.22 to 1.07)	0.004
Prevalence	Marshall Islands	2000-2010	-0.15<br>(-0.19 to -0.11)	0.000
Prevalence	Marshall Islands	2010-2015	-1.78<br>(-1.86 to -1.71)	<0.001
Prevalence	Marshall Islands	2015-2018	-0.59<br>(-0.79 to -0.41)	0.011
Prevalence	Marshall Islands	2018-2021	0.63<br>(0.45 to 0.88)	<0.001
Prevalence	Mauritania	1990-2002	0.06<br>(-0.03 to 0.1)	0.121
Prevalence	Mauritania	2002-2006	0.43<br>(0.19 to 0.65)	0.006
Prevalence	Mauritania	2006-2011	-0.64<br>(-0.81 to -0.47)	0.012
Prevalence	Mauritania	2011-2015	-2.38<br>(-2.65 to -2.18)	<0.001
Prevalence	Mauritania	2015-2018	-0.66<br>(-1.44 to -0.33)	0.035
Prevalence	Mauritania	2018-2021	0.68<br>(0.37 to 1.24)	0.000
Prevalence	Mauritius	1990-1997	0.05<br>(-0.03 to 0.12)	0.142
Prevalence	Mauritius	1997-2002	-0.91<br>(-1.08 to -0.77)	0.010
Prevalence	Mauritius	2002-2005	0.08<br>(-0.48 to 0.55)	0.401
Prevalence	Mauritius	2005-2009	0.88<br>(0.62 to 1.12)	0.027
Prevalence	Mauritius	2009-2019	-0.79<br>(-0.87 to -0.74)	0.002
Prevalence	Mauritius	2019-2021	0.17<br>(-0.31 to 0.44)	0.378
Prevalence	Mexico	1990-1995	-6.38<br>(-7.23 to -5.53)	<0.001

Prevalence	Mexico	1995-2000	4.53<br>(3.63 to 6.16)	0.002
Prevalence	Mexico	2000-2005	0.8<br>(-1.42 to 1.81)	0.289
Prevalence	Mexico	2005-2010	7.43<br>(6.47 to 8.99)	0.000
Prevalence	Mexico	2010-2019	-8.42<br>(-8.94 to -8.03)	<0.001
Prevalence	Mexico	2019-2021	1.01<br>(-2.79 to 3.27)	0.442
Prevalence	Micronesia (Federated States of)	1990-2001	0.25<br>(0.05 to 0.43)	0.016
Prevalence	Micronesia (Federated States of)	2001-2004	17.34<br>(16.25 to 18.39)	<0.001
Prevalence	Micronesia (Federated States of)	2004-2021	-1.97<br>(-2.19 to -1.74)	<0.001
Prevalence	Monaco	1990-1993	-0.94<br>(-1.6 to -0.48)	<0.001
Prevalence	Monaco	1993-1997	0.19<br>(-0.21 to 1.02)	0.159
Prevalence	Monaco	1997-2010	1.11<br>(1.05 to 1.19)	0.028
Prevalence	Monaco	2010-2019	-2.05<br>(-2.13 to -1.98)	<0.001
Prevalence	Monaco	2019-2021	0.89<br>(0.27 to 1.3)	0.012
Prevalence	Mongolia	1990-1993	-1.1<br>(-2.35 to -0.42)	0.006
Prevalence	Mongolia	1993-2006	1.43<br>(1.32 to 1.56)	<0.001
Prevalence	Mongolia	2006-2010	-0.92<br>(-1.51 to 1.31)	0.078
Prevalence	Mongolia	2010-2015	-4.66<br>(-5.31 to -1.16)	<0.001
Prevalence	Mongolia	2015-2018	-1.92<br>(-4.48 to -1.13)	<0.001
Prevalence	Mongolia	2018-2021	0.53<br>(-0.33 to 1.7)	0.137

Prevalence	Montenegro	1990-1993	-0.62 (-0.86 to -0.44)	<0.001
Prevalence	Montenegro	1993-2005	0.42 (0.39 to 0.45)	<0.001
Prevalence	Montenegro	2005-2011	-0.94 (-1 to 0.39)	0.066
Prevalence	Montenegro	2011-2015	-2.24 (-2.42 to -0.97)	<0.001
Prevalence	Montenegro	2015-2018	-1.81 (-2.13 to -1.51)	<0.001
Prevalence	Montenegro	2018-2021	-0.34 (-0.56 to -0.04)	0.044
Prevalence	Morocco	1990-1993	-1.22 (-1.55 to -1.01)	<0.001
Prevalence	Morocco	1993-2000	-0.55 (-0.61 to -0.46)	<0.001
Prevalence	Morocco	2000-2010	-1.32 (-1.36 to -1.28)	<0.001
Prevalence	Morocco	2010-2015	-3.06 (-3.2 to -2.99)	<0.001
Prevalence	Morocco	2015-2018	-2.44 (-2.65 to -2.28)	<0.001
Prevalence	Morocco	2018-2021	1.11 (0.91 to 1.32)	<0.001
Prevalence	Mozambique	1990-2002	-12.97 (-13.37 to -12.51)	<0.001
Prevalence	Mozambique	2002-2006	-5.27 (-6.37 to -4.66)	<0.001
Prevalence	Mozambique	2006-2014	-1.12 (-1.63 to -0.92)	0.000
Prevalence	Mozambique	2014-2018	0.2 (-0.39 to 0.89)	0.392
Prevalence	Mozambique	2018-2021	6.09 (5.18 to 6.94)	<0.001
Prevalence	Myanmar	1990-2007	-2.61 (-3.21 to -1.99)	<0.001
Prevalence	Myanmar	2007-2010	54.36 (39.77 to 61.06)	<0.001

Prevalence	Myanmar	2010-2021	-3.14<br>(-5.14 to -1.5)	0.001
Prevalence	Namibia	1990-1998	-8.64<br>(-9.56 to -8.04)	<0.001
Prevalence	Namibia	1998-2003	-4.16<br>(-6.65 to -3.6)	<0.001
Prevalence	Namibia	2003-2006	-2.36<br>(-3.09 to -0.63)	0.032
Prevalence	Namibia	2006-2011	0.47<br>(0.12 to 0.94)	0.025
Prevalence	Namibia	2011-2016	-1.7<br>(-2.23 to -1.35)	0.002
Prevalence	Namibia	2016-2021	-0.24<br>(-0.54 to 0.3)	0.169
Prevalence	Nauru	1990-2000	1.53<br>(1.48 to 1.58)	<0.001
Prevalence	Nauru	2000-2005	0.83<br>(0.71 to 0.97)	<0.001
Prevalence	Nauru	2005-2010	-0.65<br>(-0.76 to -0.48)	0.032
Prevalence	Nauru	2010-2015	-1.57<br>(-1.76 to -1.41)	<0.001
Prevalence	Nauru	2015-2018	-0.38<br>(-1.28 to -0.16)	0.030
Prevalence	Nauru	2018-2021	0.51<br>(0.26 to 0.95)	0.002
Prevalence	Nepal	1990-1996	0.98<br>(-1.59 to 1.97)	0.229
Prevalence	Nepal	1996-2001	3.54<br>(2.48 to 19.84)	0.001
Prevalence	Nepal	2001-2004	21.91<br>(-4.2 to 23.88)	0.068
Prevalence	Nepal	2004-2014	-5.39<br>(-6.28 to -4.56)	0.000
Prevalence	Nepal	2014-2017	10.03<br>(5.21 to 12.46)	0.002
Prevalence	Nepal	2017-2021	-4.09<br>(-8.72 to -1.93)	0.002

Prevalence	Netherlands	1990-1993	-3.02<br>(-3.55 to -2.69)	<0.001
Prevalence	Netherlands	1993-2000	-2.07<br>(-2.2 to -1.94)	<0.001
Prevalence	Netherlands	2000-2003	-0.78<br>(-1.52 to -0.1)	0.018
Prevalence	Netherlands	2003-2016	0.3<br>(0.25 to 0.34)	0.002
Prevalence	Netherlands	2016-2019	1.66<br>(1.28 to 1.86)	0.001
Prevalence	Netherlands	2019-2021	-0.17<br>(-0.68 to 0.34)	0.494
Prevalence	New Zealand	1990-1994	2<br>(1.41 to 2.28)	<0.001
Prevalence	New Zealand	1994-2001	2.77<br>(2.64 to 3.04)	<0.001
Prevalence	New Zealand	2001-2006	0.27<br>(0.06 to 0.47)	0.036
Prevalence	New Zealand	2006-2014	-2.25<br>(-2.37 to -2.11)	<0.001
Prevalence	New Zealand	2014-2019	-3.31<br>(-3.65 to -3.09)	<0.001
Prevalence	New Zealand	2019-2021	-0.03<br>(-0.73 to 0.48)	0.909
Prevalence	Nicaragua	1990-1992	-14.58<br>(-17.18 to -9.66)	<0.001
Prevalence	Nicaragua	1992-2002	-8.8<br>(-9.1 to -7.73)	<0.001
Prevalence	Nicaragua	2002-2006	-4.28<br>(-5.23 to -3.08)	0.000
Prevalence	Nicaragua	2006-2010	-1.14<br>(-1.84 to -0.33)	0.004
Prevalence	Nicaragua	2010-2018	-3.16<br>(-3.41 to -2.96)	<0.001
Prevalence	Nicaragua	2018-2021	0.78<br>(0.16 to 1.33)	0.029
Prevalence	Niger	1990-1999	-0.33<br>(-0.58 to -0.13)	0.025

Prevalence	Niger	1999-2005	-1.15 (-1.73 to -0.11)	0.034
Prevalence	Niger	2005-2010	0.65 (-1.18 to 1.34)	0.120
Prevalence	Niger	2010-2014	-0.97 (-1.45 to 0.74)	0.123
Prevalence	Niger	2014-2017	3.59 (-0.84 to 4.07)	0.124
Prevalence	Niger	2017-2021	2.35 (1.33 to 2.95)	<0.001
Prevalence	Nigeria	1990-1998	0.1 (-0.16 to 0.26)	0.388
Prevalence	Nigeria	1998-2001	2.19 (1.3 to 2.54)	<0.001
Prevalence	Nigeria	2001-2021	-0.13 (-0.2 to -0.07)	<0.001
Prevalence	Niue	1990-2003	1.32 (1.08 to 1.54)	0.024
Prevalence	Niue	2003-2006	16.81 (1.35 to 18.37)	0.002
Prevalence	Niue	2006-2015	-2.54 (-4.42 to 15.56)	0.092
Prevalence	Niue	2015-2021	-0.9 (-1.88 to 1.31)	0.232
Prevalence	North Macedonia	1990-1996	0.6 (0.31 to 0.8)	0.003
Prevalence	North Macedonia	1996-2003	1.69 (1.51 to 2.02)	<0.001
Prevalence	North Macedonia	2003-2010	0.22 (0.05 to 0.39)	0.028
Prevalence	North Macedonia	2010-2017	-3.05 (-3.21 to -2.91)	<0.001
Prevalence	North Macedonia	2017-2021	-0.56 (-0.92 to -0.19)	0.038
Prevalence	Northern Mariana Islands	1990-1994	-2.79 (-4.64 to -1.84)	0.001
Prevalence	Northern Mariana Islands	1994-2003	-0.44 (-1.04 to 0.65)	0.117

Prevalence	Northern Mariana Islands	2003-2006	6.59 (-1.19 to 7.41)	0.141
Prevalence	Northern Mariana Islands	2006-2009	2.04 (0.37 to 6.43)	0.012
Prevalence	Northern Mariana Islands	2009-2021	0.44 (-0.57 to 0.75)	0.077
Prevalence	Norway	1990-1999	-1.21 (-1.3 to -1.14)	<0.001
Prevalence	Norway	1999-2008	1.34 (1.24 to 1.45)	0.025
Prevalence	Norway	2008-2011	0 (-0.28 to 0.63)	0.807
Prevalence	Norway	2011-2015	-4 (-4.39 to -3.71)	0.012
Prevalence	Norway	2015-2018	-2.91 (-3.47 to -2.23)	<0.001
Prevalence	Norway	2018-2021	0.08 (-0.35 to 0.62)	0.611
Prevalence	Oman	1990-1995	-0.98 (-1.21 to -0.82)	<0.001
Prevalence	Oman	1995-2003	0.03 (-0.07 to 0.16)	0.505
Prevalence	Oman	2003-2009	-0.83 (-1 to -0.69)	0.000
Prevalence	Oman	2009-2016	-3.06 (-3.2 to -2.97)	<0.001
Prevalence	Oman	2016-2019	-1.34 (-1.77 to -1.11)	<0.001
Prevalence	Oman	2019-2021	1.53 (0.95 to 2.01)	<0.001
Prevalence	Pakistan	1990-1997	1.36 (0.91 to 2.66)	0.000
Prevalence	Pakistan	1997-2004	0.07 (-0.98 to 0.54)	0.848
Prevalence	Pakistan	2004-2007	14.95 (12.17 to 16.6)	0.001
Prevalence	Pakistan	2007-2012	5.09 (1.5 to 7.34)	0.026

Prevalence	Pakistan	2012-2021	-5<br>(-5.88 to -4.29)	0.001
Prevalence	Palau	1990-1995	-2.22<br>(-2.39 to -2.07)	<0.001
Prevalence	Palau	1995-1998	-0.12<br>(-1.02 to 0.26)	0.285
Prevalence	Palau	1998-2005	1.26<br>(1.16 to 1.42)	0.009
Prevalence	Palau	2005-2008	-0.09<br>(-0.52 to 0.59)	0.400
Prevalence	Palau	2008-2016	-0.93<br>(-1.07 to -0.84)	0.007
Prevalence	Palau	2016-2021	0.64<br>(0.47 to 0.82)	0.001
Prevalence	Palestine	1990-1993	-7.58<br>(-10.47 to -2.89)	0.003
Prevalence	Palestine	1993-1999	-14.44<br>(-15.87 to -13.63)	0.001
Prevalence	Palestine	1999-2003	18.94<br>(16.83 to 21.65)	0.016
Prevalence	Palestine	2003-2010	6.53<br>(5.35 to 8.25)	0.001
Prevalence	Palestine	2010-2015	-12.4<br>(-14.28 to 3.57)	0.068
Prevalence	Palestine	2015-2021	-9.93<br>(-10.78 to -7.83)	<0.001
Prevalence	Panama	1990-1994	-4.72<br>(-4.98 to -4.33)	<0.001
Prevalence	Panama	1994-2004	-1.01<br>(-1.11 to -0.94)	<0.001
Prevalence	Panama	2004-2010	-0.51<br>(-0.64 to -0.29)	0.004
Prevalence	Panama	2010-2015	-1.92<br>(-2.15 to -1.77)	<0.001
Prevalence	Panama	2015-2019	-0.95<br>(-1.17 to -0.65)	<0.001
Prevalence	Panama	2019-2021	1.31<br>(0.83 to 1.64)	<0.001

Prevalence	Papua New Guinea	1990-1997	2.95<br>(2.14 to 3.59)	<0.001
Prevalence	Papua New Guinea	1997-2000	19.01<br>(12.38 to 21.14)	<0.001
Prevalence	Papua New Guinea	2000-2015	-2.63<br>(-3.16 to -1.9)	0.002
Prevalence	Papua New Guinea	2015-2018	-9.42<br>(-10.78 to -6.3)	<0.001
Prevalence	Papua New Guinea	2018-2021	0.42<br>(-1.17 to 3.03)	0.547
Prevalence	Paraguay	1990-1994	-0.51<br>(-0.94 to -0.27)	0.001
Prevalence	Paraguay	1994-2000	0.77<br>(0.63 to 1.07)	<0.001
Prevalence	Paraguay	2000-2011	0.08<br>(0.01 to 0.14)	0.028
Prevalence	Paraguay	2011-2014	-3.71<br>(-3.93 to -3.46)	0.000
Prevalence	Paraguay	2014-2018	-2.28<br>(-2.49 to -1.87)	<0.001
Prevalence	Paraguay	2018-2021	0.05<br>(-0.32 to 0.46)	0.711
Prevalence	Peru	1990-1992	1.65<br>(0.31 to 2.91)	0.034
Prevalence	Peru	1992-1997	-5.81<br>(-6.33 to -5.4)	<0.001
Prevalence	Peru	1997-2004	-3.52<br>(-3.64 to -3.37)	<0.001
Prevalence	Peru	2004-2010	-1.23<br>(-1.37 to -1.04)	<0.001
Prevalence	Peru	2010-2018	-1.9<br>(-2.1 to -1.79)	<0.001
Prevalence	Peru	2018-2021	-0.24<br>(-0.71 to 0.51)	0.204
Prevalence	Philippines	1990-2000	-4.24<br>(-4.77 to -3.78)	0.001
Prevalence	Philippines	2000-2012	-1.38<br>(-1.64 to -1.1)	0.000

Prevalence	Philippines	2012-2018	2.75 (2.04 to 4.31)	0.005
Prevalence	Philippines	2018-2021	-1.54 (-4.14 to 0.13)	0.075
Prevalence	Poland	1990-1997	0.75 (0.64 to 0.95)	<0.001
Prevalence	Poland	1997-2001	0.31 (-0.85 to 0.51)	0.206
Prevalence	Poland	2001-2010	-1 (-1.09 to -0.91)	0.001
Prevalence	Poland	2010-2015	-4.31 (-4.54 to -4.18)	<0.001
Prevalence	Poland	2015-2018	-2.88 (-3.23 to -2.61)	<0.001
Prevalence	Poland	2018-2021	0.14 (-0.2 to 0.47)	0.286
Prevalence	Portugal	1990-1992	-1.17 (-1.45 to -0.9)	<0.001
Prevalence	Portugal	1992-1995	-2.06 (-2.54 to -1.92)	<0.001
Prevalence	Portugal	1995-2001	-3.29 (-3.35 to -3.23)	<0.001
Prevalence	Portugal	2001-2014	-2.05 (-2.08 to -2.02)	<0.001
Prevalence	Portugal	2014-2019	-1.49 (-1.65 to -1.3)	<0.001
Prevalence	Portugal	2019-2021	0.23 (-0.22 to 0.52)	0.164
Prevalence	Puerto Rico	1990-2011	0.35 (0.31 to 0.4)	0.004
Prevalence	Puerto Rico	2011-2016	-1.5 (-1.88 to 0.32)	0.067
Prevalence	Puerto Rico	2016-2019	6.15 (-1.35 to 6.86)	0.078
Prevalence	Puerto Rico	2019-2021	1.81 (0.02 to 4.72)	0.049
Prevalence	Qatar	1990-1995	0.31 (0.15 to 0.5)	<0.001

Prevalence	Qatar	1995-2000	-0.49<br>(-0.67 to -0.34)	<0.001
Prevalence	Qatar	2000-2005	1.33<br>(1.2 to 1.46)	<0.001
Prevalence	Qatar	2005-2009	-0.81<br>(-1 to -0.66)	<0.001
Prevalence	Qatar	2009-2019	-2.45<br>(-2.51 to -2.39)	<0.001
Prevalence	Qatar	2019-2021	0.86<br>(0.39 to 1.23)	0.001
Prevalence	Republic of Korea	1990-1999	-1.21<br>(-1.28 to -1.15)	<0.001
Prevalence	Republic of Korea	1999-2004	-2.81<br>(-2.93 to -2.48)	<0.001
Prevalence	Republic of Korea	2004-2010	-0.63<br>(-1.23 to -0.5)	0.000
Prevalence	Republic of Korea	2010-2015	-3.33<br>(-3.62 to -0.69)	<0.001
Prevalence	Republic of Korea	2015-2018	-2.46<br>(-3.23 to -1.78)	<0.001
Prevalence	Republic of Korea	2018-2021	-0.06<br>(-0.46 to 0.52)	0.766
Prevalence	Republic of Moldova	1990-1993	0.79<br>(0.38 to 1.12)	<0.001
Prevalence	Republic of Moldova	1993-2002	-0.68<br>(-0.77 to -0.6)	<0.001
Prevalence	Republic of Moldova	2002-2009	-1.75<br>(-1.89 to -1.61)	<0.001
Prevalence	Republic of Moldova	2009-2015	-3.29<br>(-3.5 to -3.14)	<0.001
Prevalence	Republic of Moldova	2015-2018	-1.76<br>(-2.25 to -1.38)	<0.001
Prevalence	Republic of Moldova	2018-2021	0.75<br>(0.37 to 1.22)	<0.001
Prevalence	Romania	1990-1995	1.14<br>(1.01 to 1.3)	<0.001
Prevalence	Romania	1995-2001	-1.59<br>(-1.84 to -1.47)	0.017

Prevalence	Romania	2001-2006	-1.16 (-1.38 to -0.83)	<0.001
Prevalence	Romania	2006-2014	-2.27 (-2.47 to -2.17)	<0.001
Prevalence	Romania	2014-2018	-1.57 (-1.98 to -1.15)	<0.001
Prevalence	Romania	2018-2021	-0.11 (-0.47 to 0.51)	0.504
Prevalence	Russian Federation	1990-2002	1.57 (1.48 to 1.65)	<0.001
Prevalence	Russian Federation	2002-2007	-3.58 (-3.89 to -3.35)	0.003
Prevalence	Russian Federation	2007-2015	-6.1 (-6.3 to -5.93)	<0.001
Prevalence	Russian Federation	2015-2018	-2.36 (-3.45 to -1.79)	0.002
Prevalence	Russian Federation	2018-2021	1.52 (0.82 to 2.85)	0.000
Prevalence	Rwanda	1990-1993	16.52 (4.22 to 25.38)	0.012
Prevalence	Rwanda	1993-1996	197.92 (125.12 to 234.29)	<0.001
Prevalence	Rwanda	1996-2010	-16 (-19.58 to -5.12)	0.024
Prevalence	Rwanda	2010-2014	-30.84 (-39.64 to -21.16)	0.019
Prevalence	Rwanda	2014-2021	-1.34 (-3.06 to 0.83)	0.150
Prevalence	Saint Kitts and Nevis	1990-1996	0.35 (0.11 to 0.51)	0.016
Prevalence	Saint Kitts and Nevis	1996-1999	2.73 (2.21 to 2.99)	<0.001
Prevalence	Saint Kitts and Nevis	1999-2010	1.74 (1.65 to 1.81)	<0.001
Prevalence	Saint Kitts and Nevis	2010-2015	-0.8 (-0.99 to -0.51)	0.003
Prevalence	Saint Kitts and Nevis	2015-2019	-2.45 (-2.95 to -2.11)	<0.001

Prevalence	Saint Kitts and Nevis	2019-2021	0.23 (-0.62 to 0.84)	0.493
Prevalence	Saint Lucia	1990-1993	0.32 (-0.27 to 0.7)	0.132
Prevalence	Saint Lucia	1993-2001	1.47 (1.39 to 1.65)	<0.001
Prevalence	Saint Lucia	2001-2005	0.64 (0.32 to 0.91)	<0.001
Prevalence	Saint Lucia	2005-2011	1.47 (1.36 to 1.67)	<0.001
Prevalence	Saint Lucia	2011-2017	-2.03 (-2.18 to -1.91)	<0.001
Prevalence	Saint Lucia	2017-2021	-0.54 (-0.8 to -0.18)	0.014
Prevalence	Saint Vincent and the Grenadines	1990-1993	0.18 (-0.62 to 0.58)	0.593
Prevalence	Saint Vincent and the Grenadines	1993-1996	1.88 (1.3 to 2.23)	0.011
Prevalence	Saint Vincent and the Grenadines	1996-2000	-0.68 (-1.17 to -0.33)	0.011
Prevalence	Saint Vincent and the Grenadines	2000-2008	1.1 (0.95 to 1.26)	0.006
Prevalence	Saint Vincent and the Grenadines	2008-2018	-0.97 (-1.17 to -0.88)	0.012
Prevalence	Saint Vincent and the Grenadines	2018-2021	-0.07 (-0.64 to 0.7)	0.801
Prevalence	Samoa	1990-1992	6.04 (2.21 to 7.8)	0.001
Prevalence	Samoa	1992-2003	-0.99 (-1.75 to -0.71)	0.000
Prevalence	Samoa	2003-2008	1.74 (0.82 to 3.38)	0.001
Prevalence	Samoa	2008-2011	41.6 (37.94 to 45.44)	0.001
Prevalence	Samoa	2011-2021	-5.16 (-6.3 to -3.95)	0.001
Prevalence	San Marino	1990-2000	-0.84 (-0.91 to -0.78)	<0.001

Prevalence	San Marino	2000-2004	-0.28<br>(-0.64 to 0.16)	0.104
Prevalence	San Marino	2004-2011	0.61<br>(0.52 to 0.74)	0.023
Prevalence	San Marino	2011-2014	-1.68<br>(-1.85 to -1.43)	0.044
Prevalence	San Marino	2014-2018	-1.08<br>(-1.25 to -0.82)	<0.001
Prevalence	San Marino	2018-2021	0.96<br>(0.7 to 1.2)	<0.001
Prevalence	Sao Tome and Principe	1990-1995	-1.18<br>(-1.29 to -1.07)	<0.001
Prevalence	Sao Tome and Principe	1995-2000	-2.41<br>(-2.56 to -2.3)	<0.001
Prevalence	Sao Tome and Principe	2000-2005	-1.27<br>(-1.37 to -1.12)	<0.001
Prevalence	Sao Tome and Principe	2005-2014	0.14<br>(0.03 to 0.19)	0.027
Prevalence	Sao Tome and Principe	2014-2019	0.58<br>(0.4 to 0.82)	<0.001
Prevalence	Sao Tome and Principe	2019-2021	2.5<br>(2.02 to 2.8)	<0.001
Prevalence	Saudi Arabia	1990-1996	0.34<br>(0.21 to 0.51)	<0.001
Prevalence	Saudi Arabia	1996-1999	-2.02<br>(-2.25 to -1.51)	<0.001
Prevalence	Saudi Arabia	1999-2006	-0.42<br>(-0.59 to -0.26)	<0.001
Prevalence	Saudi Arabia	2006-2009	1.45<br>(1.11 to 1.7)	<0.001
Prevalence	Saudi Arabia	2009-2018	-1.9<br>(-2.01 to -1.81)	<0.001
Prevalence	Saudi Arabia	2018-2021	-0.06<br>(-0.53 to 0.78)	0.728
Prevalence	Senegal	1990-1996	1.77<br>(1.17 to 1.98)	<0.001
Prevalence	Senegal	1996-1999	2.83<br>(1.54 to 3.21)	0.046

Prevalence	Senegal	1999-2005	-1.82 (-2.27 to -1.53)	<0.001
Prevalence	Senegal	2005-2011	-0.86 (-1.15 to -0.27)	0.006
Prevalence	Senegal	2011-2015	-2.64 (-3.18 to -2.19)	0.016
Prevalence	Senegal	2015-2021	0.59 (0.33 to 0.85)	0.006
Prevalence	Serbia	1990-1992	8.54 (3.45 to 12.8)	0.002
Prevalence	Serbia	1992-1997	-3.8 (-5.76 to -2.8)	0.002
Prevalence	Serbia	1997-2000	18.44 (12.26 to 21.6)	0.002
Prevalence	Serbia	2000-2004	-6.19 (-8.95 to -3.15)	0.004
Prevalence	Serbia	2004-2018	-2.5 (-3.64 to -0.62)	0.031
Prevalence	Serbia	2018-2021	1.04 (-2.22 to 4.5)	0.452
Prevalence	Seychelles	1990-1996	-1.29 (-1.69 to -0.97)	0.004
Prevalence	Seychelles	1996-1999	3.32 (1.93 to 3.95)	0.034
Prevalence	Seychelles	1999-2002	-0.43 (-1.11 to 0.68)	0.405
Prevalence	Seychelles	2002-2009	1.02 (-1.99 to 2.06)	0.063
Prevalence	Seychelles	2009-2018	-2.87 (-3.26 to -2.62)	<0.001
Prevalence	Seychelles	2018-2021	0.14 (-0.87 to 1.78)	0.697
Prevalence	Sierra Leone	1990-1999	16.95 (14.96 to 18.22)	<0.001
Prevalence	Sierra Leone	1999-2015	-9.1 (-9.54 to -8.46)	<0.001
Prevalence	Sierra Leone	2015-2021	1.94 (0.98 to 2.77)	0.000

Prevalence	Singapore	1990-1993	-1.52 (-2.41 to -0.78)	<0.001
Prevalence	Singapore	1993-2003	-0.3 (-0.44 to 0.05)	0.059
Prevalence	Singapore	2003-2008	2.2 (1.84 to 2.89)	0.009
Prevalence	Singapore	2008-2011	-1.13 (-1.98 to -0.17)	0.039
Prevalence	Singapore	2011-2015	-4.2 (-4.96 to -3.75)	<0.001
Prevalence	Singapore	2015-2021	-1.31 (-1.6 to -0.9)	<0.001
Prevalence	Slovakia	1990-2001	0.88 (0.83 to 0.93)	<0.001
Prevalence	Slovakia	2001-2007	-0.47 (-0.54 to -0.1)	0.035
Prevalence	Slovakia	2007-2010	-1.09 (-1.39 to -0.79)	<0.001
Prevalence	Slovakia	2010-2014	-3.48 (-3.79 to -3.29)	<0.001
Prevalence	Slovakia	2014-2018	-2.02 (-2.26 to -1.69)	<0.001
Prevalence	Slovakia	2018-2021	0 (-0.31 to 0.32)	0.993
Prevalence	Slovenia	1990-1995	-0.03 (-0.22 to 0.17)	0.671
Prevalence	Slovenia	1995-2003	1.81 (1.71 to 1.99)	0.002
Prevalence	Slovenia	2003-2006	0.45 (-0.41 to 1.32)	0.134
Prevalence	Slovenia	2006-2010	-1.31 (-1.95 to -1.07)	<0.001
Prevalence	Slovenia	2010-2018	-3.98 (-4.09 to -3.88)	<0.001
Prevalence	Slovenia	2018-2021	0.46 (0.13 to 0.77)	0.008
Prevalence	Solomon Islands	1990-2003	-1.05 (-1.31 to -0.62)	0.002

Prevalence	Solomon Islands	2003-2006	-4.79<br>(-5.62 to -3.26)	<0.001
Prevalence	Solomon Islands	2006-2009	8.16<br>(6.09 to 9.3)	<0.001
Prevalence	Solomon Islands	2009-2021	0.31<br>(-0.12 to 0.66)	0.144
Prevalence	Somalia	1990-2003	-6.59<br>(-10.7 to -1.91)	0.043
Prevalence	Somalia	2003-2006	-3.49<br>(-7.23 to 5.02)	0.199
Prevalence	Somalia	2006-2012	6.8<br>(-3.49 to 9.72)	0.074
Prevalence	Somalia	2012-2016	-0.61<br>(-3.99 to 6.51)	0.379
Prevalence	Somalia	2016-2019	7.48<br>(0.36 to 10.18)	0.042
Prevalence	Somalia	2019-2021	-4.48<br>(-9.65 to 2.61)	0.119
Prevalence	South Africa	1990-1995	-1.66<br>(-2.03 to -0.77)	0.012
Prevalence	South Africa	1995-2004	-2.44<br>(-2.75 to -2.33)	<0.001
Prevalence	South Africa	2004-2010	-1.93<br>(-2.02 to -1.8)	<0.001
Prevalence	South Africa	2010-2015	-3.62<br>(-3.71 to -3.53)	<0.001
Prevalence	South Africa	2015-2018	-1.41<br>(-1.56 to -1.25)	<0.001
Prevalence	South Africa	2018-2021	0.52<br>(0.32 to 0.71)	<0.001
Prevalence	South Sudan	1990-1996	-6.5<br>(-7.44 to -5.52)	<0.001
Prevalence	South Sudan	1996-1999	36.8<br>(33.8 to 39.84)	0.004
Prevalence	South Sudan	1999-2005	-9.33<br>(-11.31 to -7.93)	0.004
Prevalence	South Sudan	2005-2012	-1.57<br>(-3.15 to -0.35)	0.018

Prevalence	South Sudan	2012-2015	8.08<br>(3.97 to 10.2)	0.006
Prevalence	South Sudan	2015-2021	-0.08<br>(-3.23 to 1.21)	0.721
Prevalence	Spain	1990-1995	0.07<br>(-0.11 to 0.26)	0.321
Prevalence	Spain	1995-2001	-1.55<br>(-1.7 to -1.41)	0.003
Prevalence	Spain	2001-2010	0.47<br>(0.36 to 0.56)	0.020
Prevalence	Spain	2010-2014	-2.08<br>(-2.43 to -1.61)	0.037
Prevalence	Spain	2014-2018	-1<br>(-1.42 to -0.41)	0.018
Prevalence	Spain	2018-2021	1.76<br>(1.27 to 2.42)	<0.001
Prevalence	Sri Lanka	1990-1992	14.4<br>(3.52 to 21.07)	0.000
Prevalence	Sri Lanka	1992-2000	0.08<br>(-6.63 to 1.73)	0.987
Prevalence	Sri Lanka	2000-2003	-11.57<br>(-13.86 to 10.12)	0.068
Prevalence	Sri Lanka	2003-2010	12.49<br>(9.92 to 14.49)	0.032
Prevalence	Sri Lanka	2010-2015	-11.81<br>(-15.17 to -9.26)	0.009
Prevalence	Sri Lanka	2015-2021	-5.78<br>(-7.38 to -2.83)	0.009
Prevalence	Sudan	1990-1992	1.83<br>(-1.05 to 4.42)	0.224
Prevalence	Sudan	1992-1996	-3.89<br>(-5.89 to -2.79)	0.039
Prevalence	Sudan	1996-1999	3.75<br>(1.03 to 5.16)	0.032
Prevalence	Sudan	1999-2002	-0.98<br>(-2.71 to 4.23)	0.240
Prevalence	Sudan	2002-2005	4.87<br>(-3.63 to 6.15)	0.147

Prevalence	Sudan	2005-2021	-3.28 (-3.49 to -2.98)	<0.001
Prevalence	Suriname	1990-1999	-3.52 (-3.93 to -2.08)	0.011
Prevalence	Suriname	1999-2005	-4.67 (-5.06 to -4.35)	0.001
Prevalence	Suriname	2005-2011	-0.1 (-0.22 to 0.02)	0.097
Prevalence	Suriname	2011-2014	-2.03 (-2.24 to -1.66)	0.003
Prevalence	Suriname	2014-2018	-1.11 (-1.38 to -0.73)	<0.001
Prevalence	Suriname	2018-2021	0.3 (-0.01 to 0.78)	0.058
Prevalence	Sweden	1990-1995	-1.07 (-1.64 to -0.76)	0.003
Prevalence	Sweden	1995-2007	0.74 (0.63 to 0.9)	0.012
Prevalence	Sweden	2007-2010	-0.68 (-1.95 to 0.17)	0.074
Prevalence	Sweden	2010-2015	-4.48 (-5.2 to -4.16)	0.002
Prevalence	Sweden	2015-2018	-2.13 (-3.7 to -0.99)	<0.001
Prevalence	Sweden	2018-2021	0.74 (0.04 to 1.9)	0.046
Prevalence	Switzerland	1990-1994	0.62 (-0.14 to 1.08)	0.074
Prevalence	Switzerland	1994-2000	2.76 (2.5 to 3.17)	<0.001
Prevalence	Switzerland	2000-2010	-0.77 (-0.9 to -0.64)	<0.001
Prevalence	Switzerland	2010-2018	-3.65 (-3.82 to -3.48)	<0.001
Prevalence	Switzerland	2018-2021	-0.17 (-0.84 to 0.46)	0.497
Prevalence	Syrian Arab Republic	1990-1998	-2.94 (-10.58 to -0.43)	0.026

Prevalence	Syrian Arab Republic	1998-2010	0.49<br>(-0.2 to 4.9)	0.136
Prevalence	Syrian Arab Republic	2010-2016	54.54<br>(45.68 to 70.15)	0.015
Prevalence	Syrian Arab Republic	2016-2021	-3.35<br>(-17.55 to 8.33)	0.405
Prevalence	Taiwan (Province of China)	1990-1998	-0.13<br>(-1.68 to 0.36)	0.420
Prevalence	Taiwan (Province of China)	1998-2001	4.04<br>(1.45 to 5.09)	0.010
Prevalence	Taiwan (Province of China)	2001-2012	-1.15<br>(-1.69 to -0.71)	0.004
Prevalence	Taiwan (Province of China)	2012-2021	-4.09<br>(-4.72 to -3.63)	0.001
Prevalence	Tajikistan	1990-1995	29.03<br>(20.37 to 40.62)	<0.001
Prevalence	Tajikistan	1995-2014	-7.19<br>(-8.47 to -6.26)	<0.001
Prevalence	Tajikistan	2014-2021	-1.49<br>(-3.41 to 2.41)	0.251
Prevalence	Thailand	1990-2003	-1.18<br>(-1.25 to -1.11)	<0.001
Prevalence	Thailand	2003-2006	5.81<br>(5.38 to 6.2)	<0.001
Prevalence	Thailand	2006-2012	-0.28<br>(-0.58 to 0.1)	0.111
Prevalence	Thailand	2012-2021	-1.81<br>(-2.03 to -1.64)	<0.001
Prevalence	Timor-Leste	1990-1997	-3.71<br>(-6.65 to -1.69)	0.010
Prevalence	Timor-Leste	1997-2000	23.46<br>(11.58 to 27.63)	0.016
Prevalence	Timor-Leste	2000-2011	-13.1<br>(-13.75 to -11.22)	0.020
Prevalence	Timor-Leste	2011-2014	-19.01<br>(-20.72 to -15.08)	<0.001
Prevalence	Timor-Leste	2014-2019	-9.02<br>(-10 to -7.78)	<0.001

Prevalence	Timor-Leste	2019-2021	-0.73<br>(-2.57 to 0.65)	0.269
Prevalence	Togo	1990-1995	0.49<br>(-0.09 to 1.63)	0.096
Prevalence	Togo	1995-2003	-1.05<br>(-2.28 to -0.78)	0.004
Prevalence	Togo	2003-2006	3.63<br>(2.08 to 4.36)	0.004
Prevalence	Togo	2006-2016	-1.63<br>(-2.17 to -1.42)	0.001
Prevalence	Togo	2016-2021	0.28<br>(-0.49 to 2.08)	0.272
Prevalence	Tokelau	1990-1995	-0.31<br>(-0.77 to 0.05)	0.078
Prevalence	Tokelau	1995-1999	2.79<br>(2.23 to 3.49)	<0.001
Prevalence	Tokelau	1999-2004	-0.96<br>(-1.54 to -0.59)	<0.001
Prevalence	Tokelau	2004-2010	1.68<br>(1.39 to 2.12)	<0.001
Prevalence	Tokelau	2010-2015	-3.8<br>(-4.11 to -3.51)	<0.001
Prevalence	Tokelau	2015-2021	0.43<br>(0.14 to 0.7)	0.006
Prevalence	Tonga	1990-2007	-0.91<br>(-1.11 to -0.74)	<0.001
Prevalence	Tonga	2007-2010	6.83<br>(3.77 to 7.87)	<0.001
Prevalence	Tonga	2010-2021	-1.8<br>(-2.22 to -1.46)	<0.001
Prevalence	Trinidad and Tobago	1990-2000	1.72<br>(1.49 to 1.98)	<0.001
Prevalence	Trinidad and Tobago	2000-2008	0.1<br>(-0.38 to 0.53)	0.619
Prevalence	Trinidad and Tobago	2008-2017	-2.12<br>(-2.44 to -1.86)	<0.001
Prevalence	Trinidad and Tobago	2017-2021	7.75<br>(6.83 to 8.59)	<0.001

Prevalence	Tunisia	1990-1997	-1.26<br>(-1.43 to -1.14)	<0.001
Prevalence	Tunisia	1997-2004	-0.6<br>(-0.72 to -0.36)	0.002
Prevalence	Tunisia	2004-2011	-1.66<br>(-1.78 to -1.49)	<0.001
Prevalence	Tunisia	2011-2014	-2.93<br>(-3.14 to -2.5)	<0.001
Prevalence	Tunisia	2014-2019	-1.54<br>(-1.7 to -1.29)	<0.001
Prevalence	Tunisia	2019-2021	1.26<br>(0.66 to 1.69)	0.001
Prevalence	Turkey	1990-1992	1.09<br>(0.23 to 2.3)	0.011
Prevalence	Turkey	1992-1995	3.4<br>(0.31 to 3.95)	0.028
Prevalence	Turkey	1995-2000	-0.07<br>(-1.3 to 0.35)	0.578
Prevalence	Turkey	2000-2014	-1.48<br>(-1.76 to -1.4)	<0.001
Prevalence	Turkey	2014-2017	-0.22<br>(-1.01 to 0.16)	0.214
Prevalence	Turkey	2017-2021	-2.26<br>(-3.14 to -1.84)	<0.001
Prevalence	Turkmenistan	1990-1993	3.81<br>(3.45 to 4.24)	<0.001
Prevalence	Turkmenistan	1993-2003	-1.38<br>(-1.63 to -1.28)	0.000
Prevalence	Turkmenistan	2003-2009	-0.96<br>(-1.35 to -0.54)	<0.001
Prevalence	Turkmenistan	2009-2015	-3.49<br>(-3.84 to -1.01)	<0.001
Prevalence	Turkmenistan	2015-2018	-1.78<br>(-3.5 to -1.21)	<0.001
Prevalence	Turkmenistan	2018-2021	-0.38<br>(-0.94 to 0.42)	0.237
Prevalence	Tuvalu	1990-1994	1.61<br>(1.3 to 1.83)	<0.001

Prevalence	Tuvalu	1994-2006	0.1 (0.03 to 0.15)	0.013
Prevalence	Tuvalu	2006-2009	-2.66 (-2.87 to -1.75)	0.026
Prevalence	Tuvalu	2009-2016	-1.88 (-2.05 to -1.4)	0.005
Prevalence	Tuvalu	2016-2021	-0.09 (-0.34 to 0.34)	0.396
Prevalence	Uganda	1990-1995	-10.7 (-13.91 to -9.48)	<0.001
Prevalence	Uganda	1995-2001	-7.95 (-8.47 to -6.79)	0.008
Prevalence	Uganda	2001-2005	1.36 (0.31 to 2.02)	0.015
Prevalence	Uganda	2005-2013	-3.15 (-3.56 to -2.93)	0.001
Prevalence	Uganda	2013-2017	-1.89 (-2.5 to -1.01)	<0.001
Prevalence	Uganda	2017-2021	0.34 (-0.01 to 0.91)	0.060
Prevalence	Ukraine	1990-2000	0.58 (0.37 to 0.78)	0.008
Prevalence	Ukraine	2000-2008	-2.61 (-2.9 to -1.89)	0.035
Prevalence	Ukraine	2008-2013	-4.44 (-5.31 to -2.9)	0.003
Prevalence	Ukraine	2013-2016	1.84 (-3.79 to 2.63)	0.062
Prevalence	Ukraine	2016-2019	-1.45 (-2.29 to -0.14)	0.045
Prevalence	Ukraine	2019-2021	2.28 (0.51 to 3.61)	0.030
Prevalence	United Arab Emirates	1990-2000	1.19 (1.07 to 1.33)	0.001
Prevalence	United Arab Emirates	2000-2005	-2.2 (-2.66 to -1.75)	0.011
Prevalence	United Arab Emirates	2005-2008	1.91 (1.24 to 2.39)	0.014

Prevalence	United Arab Emirates	2008-2013	-4.93 (-5.55 to -4.59)	0.012
Prevalence	United Arab Emirates	2013-2018	-1.57 (-2.87 to -1.08)	<0.001
Prevalence	United Arab Emirates	2018-2021	0.67 (-0.28 to 1.9)	0.120
Prevalence	United Kingdom	1990-1995	-1.15 (-1.55 to -0.89)	<0.001
Prevalence	United Kingdom	1995-2008	0.62 (0.54 to 0.71)	0.012
Prevalence	United Kingdom	2008-2011	-1.2 (-1.87 to -0.31)	0.038
Prevalence	United Kingdom	2011-2015	-4.37 (-4.93 to -3.94)	<0.001
Prevalence	United Kingdom	2015-2018	-2.54 (-3.74 to -1.35)	<0.001
Prevalence	United Kingdom	2018-2021	0.38 (-0.18 to 1.29)	0.104
Prevalence	United Republic of Tanzania	1990-1994	-0.88 (-1.22 to -0.71)	<0.001
Prevalence	United Republic of Tanzania	1994-1998	-0.37 (-0.61 to 0.13)	0.104
Prevalence	United Republic of Tanzania	1998-2006	0.18 (0.09 to 0.56)	0.006
Prevalence	United Republic of Tanzania	2006-2010	0.84 (0.42 to 1.05)	0.049
Prevalence	United Republic of Tanzania	2010-2015	-1.03 (-1.13 to -0.9)	0.041
Prevalence	United Republic of Tanzania	2015-2021	0.54 (0.44 to 0.63)	0.001
Prevalence	United States Virgin Islands	1990-1993	-0.18 (-0.92 to 0.45)	0.301
Prevalence	United States Virgin Islands	1993-1996	1.03 (-0.07 to 1.32)	0.058
Prevalence	United States Virgin Islands	1996-2008	0.05 (-0.09 to 0.22)	0.222
Prevalence	United States Virgin Islands	2008-2011	-0.65 (-2.98 to -0.24)	0.015

Prevalence	United States Virgin Islands	2011-2015	-3.25 (-3.55 to -0.26)	0.036
Prevalence	United States Virgin Islands	2015-2021	0.18 (-0.07 to 0.42)	0.090
Prevalence	United States of America	1990-1996	-0.41 (-0.66 to -0.15)	0.003
Prevalence	United States of America	1996-1999	-9.68 (-10.17 to -9.24)	<0.001
Prevalence	United States of America	1999-2002	-3.69 (-4.07 to -3.01)	<0.001
Prevalence	United States of America	2002-2015	-1.37 (-1.46 to -1.26)	<0.001
Prevalence	United States of America	2015-2019	1.69 (1.34 to 2.5)	0.002
Prevalence	United States of America	2019-2021	-1.49 (-2.56 to -0.28)	0.006
Prevalence	Uruguay	1990-2001	0.21 (0.17 to 0.27)	0.003
Prevalence	Uruguay	2001-2004	-0.33 (-0.48 to 0)	0.050
Prevalence	Uruguay	2004-2011	0.44 (0.35 to 0.6)	0.018
Prevalence	Uruguay	2011-2015	-2.56 (-2.83 to -2.42)	0.026
Prevalence	Uruguay	2015-2018	-1.71 (-2.02 to -1.39)	<0.001
Prevalence	Uruguay	2018-2021	0.61 (0.3 to 0.96)	0.000
Prevalence	Uzbekistan	1990-1995	-0.05 (-0.72 to 0.3)	0.709
Prevalence	Uzbekistan	1995-2004	1.04 (0.88 to 1.28)	0.001
Prevalence	Uzbekistan	2004-2010	-1.49 (-1.72 to -1.18)	0.009
Prevalence	Uzbekistan	2010-2015	-4.8 (-5.24 to -4.47)	<0.001
Prevalence	Uzbekistan	2015-2019	-2.45 (-3.49 to -1.81)	<0.001

Prevalence	Uzbekistan	2019-2021	0.29 (-0.92 to 1.06)	0.558
Prevalence	Vanuatu	1990-1998	-1.66 (-2.87 to -0.83)	<0.001
Prevalence	Vanuatu	1998-2001	8.91 (4.1 to 11.5)	<0.001
Prevalence	Vanuatu	2001-2008	-4.55 (-7.93 to -3.55)	<0.001
Prevalence	Vanuatu	2008-2021	-1.87 (-2.15 to -1.51)	0.001
Prevalence	Venezuela (Bolivarian Republic of)	1990-1998	0.46 (-0.24 to 0.93)	0.125
Prevalence	Venezuela (Bolivarian Republic of)	1998-2001	9.47 (6.03 to 10.76)	0.000
Prevalence	Venezuela (Bolivarian Republic of)	2001-2012	-1.65 (-2.11 to -1.06)	0.003
Prevalence	Venezuela (Bolivarian Republic of)	2012-2021	-3.73 (-4.56 to -3.24)	<0.001
Prevalence	Viet Nam	1990-1995	-0.03 (-0.26 to 0.2)	0.763
Prevalence	Viet Nam	1995-1999	3.13 (2.73 to 3.62)	0.001
Prevalence	Viet Nam	1999-2004	1.07 (0.61 to 1.63)	<0.001
Prevalence	Viet Nam	2004-2010	0.06 (-0.62 to 0.26)	0.662
Prevalence	Viet Nam	2010-2017	-3.07 (-3.27 to -2.91)	<0.001
Prevalence	Viet Nam	2017-2021	-0.51 (-0.89 to -0.1)	0.041
Prevalence	Yemen	1990-2005	-2.91 (-3.18 to -2.59)	<0.001
Prevalence	Yemen	2005-2009	10.19 (8.93 to 11.37)	<0.001
Prevalence	Yemen	2009-2015	3.23 (1.97 to 4.23)	0.002
Prevalence	Yemen	2015-2021	17.86 (14.92 to 22.71)	<0.001

Prevalence	Zambia	1990-1996	-0.92<br>(-1.08 to -0.81)	<0.001
Prevalence	Zambia	1996-2005	-0.3<br>(-0.38 to -0.23)	<0.001
Prevalence	Zambia	2005-2010	0.64<br>(0.52 to 0.85)	0.004
Prevalence	Zambia	2010-2015	-1.96<br>(-2.15 to -1.85)	0.004
Prevalence	Zambia	2015-2018	-0.77<br>(-1.29 to -0.42)	0.030
Prevalence	Zambia	2018-2021	1.08<br>(0.78 to 1.58)	<0.001
Prevalence	Zimbabwe	1990-2002	-0.14<br>(-0.2 to -0.07)	0.013
Prevalence	Zimbabwe	2002-2006	-1.38<br>(-1.83 to -0.94)	0.013
Prevalence	Zimbabwe	2006-2009	0.99<br>(0.13 to 1.3)	0.048
Prevalence	Zimbabwe	2009-2014	-1.72<br>(-2.08 to -1.43)	0.048
Prevalence	Zimbabwe	2014-2017	-0.27<br>(-1.48 to 0.48)	0.180
Prevalence	Zimbabwe	2017-2021	1.03<br>(0.72 to 1.74)	<0.001

APC annual percentage change, *CI* confidence interval.

Supplementary Table 19 Joinpoint regression analysis of YLDs of spinal cord injury from 1990 to 2021 among children and adolescents at national level

Measure	Location	Year	APC (95% CI)	P
YLDs	Afghanistan	1990-2005	-12.94<br>(-13.22 to -12.6)	<0.001
YLDs	Afghanistan	2005-2009	6.45<br>(5.44 to 7.69)	0.003
YLDs	Afghanistan	2009-2013	1.65<br>(-0.25 to 2.81)	0.072
YLDs	Afghanistan	2013-2019	9.7<br>(8.99 to 11.38)	<0.001
YLDs	Afghanistan	2019-2021	0.97<br>(-2.04 to 4.62)	0.375
YLDs	Albania	1990-1995	-0.59<br>(-3.17 to 0.67)	0.246
YLDs	Albania	1995-1998	9.43<br>(4.97 to 10.95)	0.020
YLDs	Albania	1998-2009	-1.59<br>(-2.01 to -0.01)	0.050
YLDs	Albania	2009-2021	-2.53<br>(-4.85 to -2.17)	<0.001
YLDs	Algeria	1990-1993	-0.91<br>(-2.66 to 0.14)	0.077
YLDs	Algeria	1993-1998	4.48<br>(3.9 to 5.4)	<0.001
YLDs	Algeria	1998-2002	-0.9<br>(-1.76 to 0.49)	0.124
YLDs	Algeria	2002-2009	-3.72<br>(-4.23 to -3.25)	<0.001

YLDs	Algeria	2009-2017	-5.83<br>(-6.29 to -5.53)	<0.001
YLDs	Algeria	2017-2021	-0.76<br>(-1.55 to 0.08)	0.057
YLDs	American Samoa	1990-2001	0.12<br>(-1.14 to 0.73)	0.764
YLDs	American Samoa	2001-2008	3.31<br>(2.04 to 5.79)	<0.001
YLDs	American Samoa	2008-2011	28.43<br>(20.56 to 31.73)	0.011
YLDs	American Samoa	2011-2021	-3.19<br>(-4.32 to -2.15)	0.004
YLDs	Andorra	1990-2000	-0.94<br>(-1.14 to -0.83)	0.001
YLDs	Andorra	2000-2005	0.05<br>(-0.53 to 0.6)	0.773
YLDs	Andorra	2005-2009	1.4<br>(0.83 to 1.89)	0.015
YLDs	Andorra	2009-2018	-1.33<br>(-1.48 to -1.22)	<0.001
YLDs	Andorra	2018-2021	1.06<br>(0.5 to 2.13)	0.002
YLDs	Angola	1990-1994	1.56<br>(-0.6 to 6.9)	0.128
YLDs	Angola	1994-1997	-10.01<br>(-12.99 to -6.03)	0.014

YLDs	Angola	1997-2000	8.05 (2.76 to 11.69)	0.015
YLDs	Angola	2000-2005	-11.54 (-14.56 to -9.62)	<0.001
YLDs	Angola	2005-2015	-8.54 (-9.15 to -6.13)	0.003
YLDs	Angola	2015-2021	-1.88 (-3.07 to 0.09)	0.057
YLDs	Antigua and Barbuda	1990-1996	0.91 (0.09 to 1.55)	0.037
YLDs	Antigua and Barbuda	1996-1999	10.45 (7.35 to 11.58)	0.005
YLDs	Antigua and Barbuda	1999-2013	-0.77 (-1.05 to -0.46)	0.014
YLDs	Antigua and Barbuda	2013-2018	-4.54 (-6.73 to -3.39)	0.011
YLDs	Antigua and Barbuda	2018-2021	-0.06 (-2.14 to 3.28)	0.932
YLDs	Argentina	1990-2000	-0.24 (-0.35 to -0.12)	<0.001
YLDs	Argentina	2000-2005	2.69 (2.44 to 2.98)	<0.001
YLDs	Argentina	2005-2011	-0.44 (-0.65 to -0.26)	0.004
YLDs	Argentina	2011-2019	-3.58 (-3.74 to -3.45)	<0.001

YLDs	Argentina	2019-2021	0.99 (0.01 to 1.63)	0.049
YLDs	Armenia	1990-2006	-4.26 (-5.24 to -2.91)	<0.001
YLDs	Armenia	2006-2009	-27.41 (-30.38 to -18.69)	<0.001
YLDs	Armenia	2009-2019	-1.39 (-3.01 to 0.66)	0.121
YLDs	Armenia	2019-2021	79.35 (46.17 to 98.61)	<0.001
YLDs	Australia	1990-1995	-0.57 (-0.86 to -0.36)	0.001
YLDs	Australia	1995-2005	0.5 (0.42 to 0.6)	0.005
YLDs	Australia	2005-2011	-0.48 (-0.65 to -0.3)	0.012
YLDs	Australia	2011-2015	-2.47 (-2.79 to -2.21)	<0.001
YLDs	Australia	2015-2019	-1.32 (-1.74 to -0.99)	<0.001
YLDs	Australia	2019-2021	0.42 (-0.21 to 0.82)	0.146
YLDs	Austria	1990-1993	-2.57 (-3.29 to -2.16)	<0.001
YLDs	Austria	1993-1996	-0.49 (-1.43 to 0.58)	0.072

YLDs	Austria	1996-1999	1.7<br>(1.15 to 1.99)	<0.001
YLDs	Austria	1999-2010	0.67<br>(0.57 to 0.73)	0.020
YLDs	Austria	2010-2017	-2.52<br>(-2.66 to -2.39)	<0.001
YLDs	Austria	2017-2021	-0.61<br>(-0.94 to -0.12)	0.024
YLDs	Azerbaijan	1990-1994	9.63<br>(6.01 to 14.18)	<0.001
YLDs	Azerbaijan	1994-2019	-2.56<br>(-2.83 to -2.32)	<0.001
YLDs	Azerbaijan	2019-2021	22.91<br>(12.06 to 27.86)	<0.001
YLDs	Bahamas	1990-2018	0.08<br>(-0.06 to 0.22)	0.243
YLDs	Bahamas	2018-2021	12.92<br>(8.71 to 20.51)	<0.001
YLDs	Bahrain	1990-1993	3.67<br>(2.66 to 5.02)	<0.001
YLDs	Bahrain	1993-1998	0.24<br>(-0.34 to 0.87)	0.298
YLDs	Bahrain	1998-2002	-2.59<br>(-3.64 to -1.97)	0.004
YLDs	Bahrain	2002-2005	1.27<br>(0.04 to 2.02)	0.047
YLDs	Bahrain	2005-2009	-4.81<br>(-6 to -4.2)	0.002

YLDs	Bahrain	2009-2021	-1.17 (-1.33 to -0.99)	0.000
YLDs	Bangladesh	1990-1992	53.7 (46.7 to 61.7)	<0.001
YLDs	Bangladesh	1992-2009	-4.11 (-4.42 to -3.65)	<0.001
YLDs	Bangladesh	2009-2012	-11.64 (-12.98 to -8.36)	<0.001
YLDs	Bangladesh	2012-2021	-1.06 (-1.76 to -0.04)	0.042
YLDs	Barbados	1990-1994	1.73 (1.18 to 2.41)	<0.001
YLDs	Barbados	1994-1998	-1.66 (-2.41 to -1.04)	0.001
YLDs	Barbados	1998-2012	0.03 (-0.06 to 0.17)	0.440
YLDs	Barbados	2012-2016	-1.65 (-2.33 to -1.11)	0.006
YLDs	Barbados	2016-2021	-0.23 (-0.61 to 0.77)	0.443
YLDs	Belarus	1990-2001	1.34 (1.19 to 1.53)	0.002
YLDs	Belarus	2001-2005	-0.69 (-1.43 to 0.6)	0.116
YLDs	Belarus	2005-2009	-3.23 (-3.99 to -2.26)	0.003

YLDs	Belarus	2009-2015	-5.44 (-6.41 to -5.06)	<0.001
YLDs	Belarus	2015-2019	-2.38 (-4.11 to -1.47)	<0.001
YLDs	Belarus	2019-2021	1.76 (0.01 to 2.79)	0.050
YLDs	Belgium	1990-1994	-1.33 (-2.48 to -0.65)	<0.001
YLDs	Belgium	1994-1998	1.76 (0.57 to 2.98)	0.018
YLDs	Belgium	1998-2005	4 (3.62 to 4.74)	0.001
YLDs	Belgium	2005-2010	0.5 (-0.08 to 0.96)	0.066
YLDs	Belgium	2010-2018	-6.48 (-6.71 to -6.26)	<0.001
YLDs	Belgium	2018-2021	-0.26 (-1.09 to 0.51)	0.470
YLDs	Belize	1990-1995	2.69 (1.92 to 3.7)	<0.001
YLDs	Belize	1995-2003	-0.47 (-0.96 to 0.05)	0.071
YLDs	Belize	2003-2017	-2.04 (-2.3 to -1.87)	<0.001
YLDs	Belize	2017-2021	1.95 (0.79 to 3.76)	0.002
YLDs	Benin	1990-2010	-0.04 (-0.08 to 0)	0.032

YLDs	Benin	2010-2019	-1.09 (-1.24 to -0.97)	<0.001
YLDs	Benin	2019-2021	1.75 (0.92 to 2.29)	<0.001
YLDs	Bermuda	1990-1998	-0.38 (-0.53 to -0.26)	<0.001
YLDs	Bermuda	1998-2004	0.8 (0.62 to 1.15)	<0.001
YLDs	Bermuda	2004-2011	-0.03 (-0.22 to 0.12)	0.683
YLDs	Bermuda	2011-2015	-1.68 (-2.13 to -1.34)	<0.001
YLDs	Bermuda	2015-2021	0.17 (0 to 0.39)	0.046
YLDs	Bhutan	1990-1999	2.44 (1.68 to 2.93)	0.000
YLDs	Bhutan	1999-2002	10.38 (6.74 to 11.92)	<0.001
YLDs	Bhutan	2002-2012	-1.06 (-1.68 to -0.31)	0.018
YLDs	Bhutan	2012-2021	-3.8 (-4.84 to -3.21)	<0.001
YLDs	Bolivia (Plurinational State of)	1990-2000	-0.51 (-0.59 to -0.4)	0.000
YLDs	Bolivia (Plurinational State of)	2000-2009	-1.06 (-1.2 to -0.94)	<0.001
YLDs	Bolivia (Plurinational State of)	2009-2018	-2.2 (-2.33 to -2.09)	<0.001

YLDs	Bolivia (Plurinational State of)	2018-2021	-0.26 (-0.76 to 0.54)	0.153
YLDs	Bosnia and Herzegovina	1990-1995	29.79 (23.95 to 35.42)	<0.001
YLDs	Bosnia and Herzegovina	1995-2000	-11.88 (-17.65 to -5.36)	0.036
YLDs	Bosnia and Herzegovina	2000-2015	-6.27 (-7.82 to -4.52)	0.012
YLDs	Bosnia and Herzegovina	2015-2021	-1.19 (-4.04 to 5.98)	0.459
YLDs	Botswana	1990-2002	1.2 (1.12 to 1.3)	<0.001
YLDs	Botswana	2002-2009	0.33 (0.07 to 0.52)	0.018
YLDs	Botswana	2009-2018	-1.06 (-1.35 to -0.93)	0.001
YLDs	Botswana	2018-2021	0.35 (-0.33 to 1.21)	0.236
YLDs	Brazil	1990-1994	2.31 (2 to 2.76)	<0.001
YLDs	Brazil	1994-1997	0.57 (-0.45 to 1.34)	0.134
YLDs	Brazil	1997-2005	-0.68 (-0.98 to -0.54)	0.000
YLDs	Brazil	2005-2011	-1.69 (-2.08 to -1.49)	<0.001
YLDs	Brazil	2011-2016	-3.76 (-4.08 to -3.47)	<0.001

YLDs	Brazil	2016-2021	-0.93<br>(-1.2 to -0.64)	0.002
YLDs	Brunei Darussalam	1990-1994	-0.88<br>(-1.54 to -0.55)	0.003
YLDs	Brunei Darussalam	1994-2010	-0.09<br>(-0.21 to 0.28)	0.128
YLDs	Brunei Darussalam	2010-2014	-2.31<br>(-2.63 to 0.05)	0.078
YLDs	Brunei Darussalam	2014-2019	-1.31<br>(-2.48 to -1.11)	<0.001
YLDs	Brunei Darussalam	2019-2021	-0.32<br>(-1.14 to 0.16)	0.169
YLDs	Bulgaria	1990-2003	-0.32<br>(-0.38 to -0.27)	<0.001
YLDs	Bulgaria	2003-2010	-1.72<br>(-1.86 to -1.55)	<0.001
YLDs	Bulgaria	2010-2014	-3.53<br>(-3.94 to -3.15)	<0.001
YLDs	Bulgaria	2014-2019	-1.76<br>(-1.99 to -1.51)	<0.001
YLDs	Bulgaria	2019-2021	1.46<br>(0.78 to 1.97)	0.001
YLDs	Burkina Faso	1990-2000	-0.49<br>(-0.75 to -0.32)	<0.001
YLDs	Burkina Faso	2000-2017	0.14<br>(0.06 to 0.24)	0.001

YLDs	Burkina Faso	2017-2021	6.07<br>(5.6 to 6.45)	<0.001
YLDs	Burundi	1990-1993	5.62<br>(1.18 to 10.26)	0.012
YLDs	Burundi	1993-1996	107.82<br>(98.33 to 116.61)	<0.001
YLDs	Burundi	1996-2005	4.84<br>(2.32 to 7.41)	0.008
YLDs	Burundi	2005-2018	-16.25<br>(-18.93 to -15.18)	0.016
YLDs	Burundi	2018-2021	-8.39<br>(-13.8 to -0.93)	0.033
YLDs	Cabo Verde	1990-1994	-0.93<br>(-1.53 to -0.58)	<0.001
YLDs	Cabo Verde	1994-2002	0.14<br>(0.01 to 0.57)	0.030
YLDs	Cabo Verde	2002-2009	-0.4<br>(-0.67 to -0.24)	<0.001
YLDs	Cabo Verde	2009-2015	-2.03<br>(-2.28 to -1.82)	<0.001
YLDs	Cabo Verde	2015-2021	-0.07<br>(-0.25 to 0.19)	0.398
YLDs	Cambodia	1990-1993	-9.94<br>(-12.69 to -7.55)	<0.001
YLDs	Cambodia	1993-1997	-6.17<br>(-10.64 to -4.6)	<0.001

YLDs	Cambodia	1997-2001	-10.83 (-11.64 to -5.48)	<0.001
YLDs	Cambodia	2001-2006	-5.4 (-6.03 to -3.09)	<0.001
YLDs	Cambodia	2006-2016	-3 (-3.22 to -2.74)	<0.001
YLDs	Cambodia	2016-2021	-1.11 (-1.55 to -0.51)	0.011
YLDs	Cameroon	1990-2003	-0.25 (-0.4 to 0.06)	0.070
YLDs	Cameroon	2003-2013	-0.77 (-1.45 to -0.55)	0.023
YLDs	Cameroon	2013-2021	3.36 (3.02 to 3.82)	<0.001
YLDs	Canada	1990-1993	-1.86 (-2.48 to -1.46)	<0.001
YLDs	Canada	1993-2006	-0.52 (-0.57 to -0.46)	0.000
YLDs	Canada	2006-2011	-1.4 (-1.69 to -1.11)	<0.001
YLDs	Canada	2011-2017	-2.52 (-2.71 to -2.38)	<0.001
YLDs	Canada	2017-2021	0.04 (-0.18 to 0.25)	0.700
YLDs	Central African Republic	1990-1995	-0.04 (-3.15 to 1.25)	0.893

YLDs	Central African Republic	1995-2012	2.13 (1.89 to 2.57)	<0.001
YLDs	Central African Republic	2012-2015	24.03 (20.99 to 26.08)	0.004
YLDs	Central African Republic	2015-2021	-2.12 (-3.55 to -0.78)	0.006
YLDs	Chad	1990-1998	-6.22 (-8.57 to -5.34)	<0.001
YLDs	Chad	1998-2004	-3.16 (-4.55 to 0.23)	0.053
YLDs	Chad	2004-2008	5.5 (2.9 to 8.25)	0.023
YLDs	Chad	2008-2013	-5.61 (-8.08 to -4.01)	0.008
YLDs	Chad	2013-2021	-0.91 (-1.61 to 0.07)	0.063
YLDs	Chile	1990-1995	0.29 (0.01 to 0.58)	0.042
YLDs	Chile	1995-2000	3.78 (3.48 to 4.3)	0.000
YLDs	Chile	2000-2004	2.25 (1.41 to 2.82)	0.000
YLDs	Chile	2004-2010	0.07 (-0.24 to 0.29)	0.534
YLDs	Chile	2010-2019	-3.12 (-3.25 to -3)	<0.001

YLDs	Chile	2019-2021	1.33 (0.29 to 2.01)	0.030
YLDs	China	1990-1993	-2.18 (-3.17 to -1.46)	<0.001
YLDs	China	1993-2001	-1.04 (-1.21 to -0.66)	<0.001
YLDs	China	2001-2005	-8.03 (-8.49 to -7.66)	<0.001
YLDs	China	2005-2008	-3.88 (-4.56 to -2.37)	<0.001
YLDs	China	2008-2018	-1.17 (-1.32 to -0.98)	0.000
YLDs	China	2018-2021	2.87 (2.1 to 3.53)	<0.001
YLDs	Colombia	1990-1995	-2.33 (-3.61 to -1.79)	0.000
YLDs	Colombia	1995-2003	-0.78 (-1.01 to -0.17)	0.027
YLDs	Colombia	2003-2006	-5.04 (-5.68 to -3.88)	<0.001
YLDs	Colombia	2006-2011	-2.19 (-2.62 to -1.07)	<0.001
YLDs	Colombia	2011-2018	-3.52 (-4.5 to -3.25)	<0.001
YLDs	Colombia	2018-2021	-0.28 (-1.37 to 1.49)	0.457

YLDs	Comoros	1990-1995	-1.34<br>(-2.84 to -0.54)	0.014
YLDs	Comoros	1995-1998	4.18<br>(1.89 to 5.36)	0.026
YLDs	Comoros	1998-2003	-2.21<br>(-3.67 to -1.34)	0.028
YLDs	Comoros	2003-2016	-0.8<br>(-1.1 to 0.02)	0.052
YLDs	Comoros	2016-2019	6.21<br>(-1.34 to 7.26)	0.125
YLDs	Comoros	2019-2021	0.56<br>(-1.83 to 4.23)	0.572
YLDs	Congo	1990-1996	2.43<br>(-1.36 to 5.82)	0.146
YLDs	Congo	1996-1999	99.02<br>(55.15 to 118.1)	0.024
YLDs	Congo	1999-2002	-24.97<br>(-32.66 to -10.76)	0.047
YLDs	Congo	2002-2015	-10.6<br>(-12.4 to -6.87)	0.030
YLDs	Congo	2015-2021	-1.88<br>(-5.57 to 5.7)	0.374
YLDs	Cook Islands	1990-1996	-1.01<br>(-3.37 to 0.68)	0.200
YLDs	Cook Islands	1996-1999	24.69<br>(15.87 to 28)	0.000

YLDs	Cook Islands	1999-2015	-3.96 (-4.45 to -3.37)	0.001
YLDs	Cook Islands	2015-2018	-14.87 (-17.19 to -10.29)	0.000
YLDs	Cook Islands	2018-2021	1.98 (-2.17 to 10.21)	0.244
YLDs	Costa Rica	1990-1994	0.46 (-0.01 to 0.69)	0.054
YLDs	Costa Rica	1994-2000	0.93 (0.77 to 1.29)	0.021
YLDs	Costa Rica	2000-2010	0.08 (0 to 0.15)	0.053
YLDs	Costa Rica	2010-2015	-3.23 (-3.43 to -3.12)	0.002
YLDs	Costa Rica	2015-2018	-1.98 (-2.38 to -1.58)	<0.001
YLDs	Costa Rica	2018-2021	0.13 (-0.17 to 0.57)	0.299
YLDs	Coted'Ivoire	1990-2001	0.55 (0.42 to 0.66)	<0.001
YLDs	Coted'Ivoire	2001-2004	3.29 (2.49 to 3.68)	<0.001
YLDs	Coted'Ivoire	2004-2009	-1.86 (-2.21 to -1.56)	<0.001
YLDs	Coted'Ivoire	2009-2012	2.42 (1.79 to 2.91)	<0.001
YLDs	Coted'Ivoire	2012-2016	-3.08 (-3.89 to -2.66)	<0.001

YLDs	Coted'Ivoire	2016-2021	0.15 (-0.21 to 0.64)	0.340
YLDs	Croatia	1990-1992	25.13 (23.32 to 27.19)	<0.001
YLDs	Croatia	1992-1995	-1.69 (-3.1 to -0.82)	0.000
YLDs	Croatia	1995-1998	-4.47 (-5.06 to -1.72)	<0.001
YLDs	Croatia	1998-2010	-1.8 (-2.51 to -1.54)	<0.001
YLDs	Croatia	2010-2018	-3.7 (-4.17 to -3.38)	<0.001
YLDs	Croatia	2018-2021	-0.05 (-1.12 to 1.73)	0.897
YLDs	Cuba	1990-1996	-1.55 (-2.04 to -1.21)	<0.001
YLDs	Cuba	1996-2010	1.06 (0.96 to 1.18)	<0.001
YLDs	Cuba	2010-2016	-2.84 (-3.28 to -2.53)	<0.001
YLDs	Cuba	2016-2021	-0.79 (-1.27 to 0.01)	0.051
YLDs	Cyprus	1990-1994	-0.19 (-0.84 to 0.12)	0.199
YLDs	Cyprus	1994-2000	0.8 (0.43 to 1.11)	0.001

YLDs	Cyprus	2000-2006	1.43<br>(1.21 to 1.92)	0.003
YLDs	Cyprus	2006-2010	0.34<br>(-0.08 to 0.61)	0.070
YLDs	Cyprus	2010-2018	-3.86<br>(-3.97 to -3.75)	<0.001
YLDs	Cyprus	2018-2021	-0.33<br>(-0.72 to -0.02)	0.041
YLDs	Czechia	1990-1999	-1.3<br>(-1.59 to -1.12)	0.010
YLDs	Czechia	1999-2005	1.78<br>(-0.98 to 2.28)	0.084
YLDs	Czechia	2005-2008	-0.76<br>(-1.52 to 1.64)	0.222
YLDs	Czechia	2008-2015	-2.4<br>(-2.62 to -2.07)	<0.001
YLDs	Czechia	2015-2018	-4.2<br>(-4.7 to -3.51)	<0.001
YLDs	Czechia	2018-2021	-0.27<br>(-0.97 to 1.1)	0.326
YLDs	Democratic People's Republic of Korea	1990-1993	-5.22<br>(-5.62 to -4.81)	<0.001
YLDs	Democratic People's Republic of Korea	1993-1998	-2.44<br>(-2.63 to -2.23)	<0.001
YLDs	Democratic People's Republic of Korea	1998-2010	-0.22<br>(-0.29 to -0.16)	0.000

YLDs	Democratic People's Republic of Korea	2010-2016	-2.44 (-2.68 to -2.26)	<0.001
YLDs	Democratic People's Republic of Korea	2016-2021	-0.86 (-1.16 to -0.43)	0.004
YLDs	Democratic Republic of the Congo	1990-1995	0.55 (-1.36 to 2.27)	0.401
YLDs	Democratic Republic of the Congo	1995-1998	34.82 (26.14 to 38.74)	0.014
YLDs	Democratic Republic of the Congo	1998-2001	-9.01 (-11.47 to -4.48)	0.038
YLDs	Democratic Republic of the Congo	2001-2016	-4.26 (-4.77 to -2.76)	0.018
YLDs	Democratic Republic of the Congo	2016-2021	1.37 (-0.75 to 6.24)	0.174
YLDs	Denmark	1990-1997	-1.52 (-1.82 to -1.38)	<0.001
YLDs	Denmark	1997-2000	-0.43 (-0.93 to 1.38)	0.195
YLDs	Denmark	2000-2009	1.72 (1.62 to 1.91)	0.012
YLDs	Denmark	2009-2012	-0.62 (-1.09 to -0.02)	0.043
YLDs	Denmark	2012-2018	-2.49 (-2.73 to -2.34)	<0.001
YLDs	Denmark	2018-2021	0.1 (-0.35 to 0.57)	0.527

YLDs	Djibouti	1990-1993	14.5<br>(10.14 to 16.21)	<0.001
YLDs	Djibouti	1993-2016	-2.5<br>(-2.68 to -2.37)	<0.001
YLDs	Djibouti	2016-2021	-0.28<br>(-1.3 to 1.95)	0.647
YLDs	Dominica	1990-1994	-0.87<br>(-2.04 to -0.16)	0.016
YLDs	Dominica	1994-2011	1.36<br>(1.27 to 1.47)	<0.001
YLDs	Dominica	2011-2014	-2.51<br>(-3.08 to -1.4)	<0.001
YLDs	Dominica	2014-2018	9.58<br>(8.89 to 10.25)	<0.001
YLDs	Dominica	2018-2021	0.39<br>(-0.65 to 1.36)	0.330
YLDs	Dominican Republic	1990-1995	-0.65<br>(-1.3 to -0.25)	0.003
YLDs	Dominican Republic	1995-2010	0.86<br>(0.77 to 0.96)	<0.001
YLDs	Dominican Republic	2010-2018	-2.46<br>(-2.75 to -2.29)	0.002
YLDs	Dominican Republic	2018-2021	-0.43<br>(-1.38 to 0.94)	0.359
YLDs	Ecuador	1990-2003	-1.2<br>(-1.47 to -0.89)	0.006

YLDs	Ecuador	2003-2018	-2.39 (-3.09 to -2.17)	0.008
YLDs	Ecuador	2018-2021	0.13 (-2 to 2.42)	0.896
YLDs	Egypt	1990-2003	-1.85 (-1.92 to -1.76)	<0.001
YLDs	Egypt	2003-2016	-2.39 (-2.5 to -2.31)	<0.001
YLDs	Egypt	2016-2021	-0.56 (-0.9 to -0.1)	0.024
YLDs	El Salvador	1990-1997	-13.68 (-14.69 to -13.2)	<0.001
YLDs	El Salvador	1997-2000	-10.3 (-12.48 to -7.65)	<0.001
YLDs	El Salvador	2000-2004	-5.71 (-6.5 to -3.67)	0.001
YLDs	El Salvador	2004-2011	-1.08 (-1.43 to -0.34)	0.022
YLDs	El Salvador	2011-2017	-2.83 (-3.94 to -2.38)	0.000
YLDs	El Salvador	2017-2021	0.11 (-0.66 to 1.4)	0.725
YLDs	Equatorial Guinea	1990-2000	-0.44 (-0.51 to -0.33)	0.002
YLDs	Equatorial Guinea	2000-2003	-1.98 (-2.21 to -1.46)	0.002
YLDs	Equatorial Guinea	2003-2011	-1.06 (-1.17 to -0.67)	<0.001

YLDs	Equatorial Guinea	2011-2015	-2.59 (-3.17 to -2.23)	0.002
YLDs	Equatorial Guinea	2015-2021	-0.13 (-0.33 to 0.16)	0.175
YLDs	Eritrea	1990-1992	-4.41 (-10.12 to 0.63)	0.072
YLDs	Eritrea	1992-1997	-17.47 (-20.06 to -16.38)	0.000
YLDs	Eritrea	1997-2000	0.99 (-4.03 to 4.07)	0.545
YLDs	Eritrea	2000-2005	-17.44 (-21.59 to -15.47)	<0.001
YLDs	Eritrea	2005-2015	-9.79 (-10.74 to -8.69)	<0.001
YLDs	Eritrea	2015-2021	-2.67 (-3.41 to -1.67)	<0.001
YLDs	Estonia	1990-1997	-0.16 (-0.29 to 0.03)	0.079
YLDs	Estonia	1997-2001	-0.89 (-1.6 to -0.63)	<0.001
YLDs	Estonia	2001-2008	-3.01 (-3.18 to -2.86)	<0.001
YLDs	Estonia	2008-2014	-4.54 (-4.72 to -4.35)	<0.001
YLDs	Estonia	2014-2018	-1.39 (-1.85 to -1.14)	<0.001

YLDs	Estonia	2018-2021	0.68 (0.21 to 1.48)	0.009
YLDs	Eswatini	1990-1993	-1.65 (-2.6 to -0.85)	0.000
YLDs	Eswatini	1993-2004	-0.73 (-0.92 to 0.17)	0.077
YLDs	Eswatini	2004-2010	0.15 (-1.29 to 0.95)	0.421
YLDs	Eswatini	2010-2019	-0.95 (-1.32 to -0.66)	0.002
YLDs	Eswatini	2019-2021	3.62 (2.17 to 4.51)	<0.001
YLDs	Ethiopia	1990-1998	-7.28 (-8.64 to -6.01)	0.002
YLDs	Ethiopia	1998-2001	18.24 (8.97 to 22.54)	0.024
YLDs	Ethiopia	2001-2004	-14.34 (-17.52 to -7.33)	0.024
YLDs	Ethiopia	2004-2016	-5.46 (-6.29 to -4.02)	0.003
YLDs	Ethiopia	2016-2021	1.15 (-0.82 to 5.14)	0.234
YLDs	Fiji	1990-2001	1.14 (0.98 to 1.34)	<0.001
YLDs	Fiji	2001-2010	-0.54 (-0.83 to -0.26)	0.001

YLDs	Fiji	2010-2015	-2.68<br>(-3.7 to -2.11)	<0.001
YLDs	Fiji	2015-2021	0.13<br>(-0.29 to 0.7)	0.500
YLDs	Finland	1990-1995	1.41<br>(0.66 to 1.84)	0.009
YLDs	Finland	1995-2004	2.84<br>(2.66 to 3.1)	<0.001
YLDs	Finland	2004-2010	-0.62<br>(-0.86 to -0.35)	0.004
YLDs	Finland	2010-2018	-4.72<br>(-4.9 to -4.55)	<0.001
YLDs	Finland	2018-2021	0.48<br>(-0.13 to 1.01)	0.094
YLDs	France	1990-1994	-1.7<br>(-2 to -1.5)	<0.001
YLDs	France	1994-2001	-0.03<br>(-0.2 to 0.09)	0.517
YLDs	France	2001-2006	0.85<br>(0.64 to 1.27)	<0.001
YLDs	France	2006-2010	-0.12<br>(-0.61 to 0.09)	0.230
YLDs	France	2010-2018	-1.84<br>(-1.93 to -1.75)	<0.001
YLDs	France	2018-2021	0.42<br>(0.12 to 0.71)	0.011
YLDs	Gabon	1990-1995	-1.44<br>(-2.56 to -0.96)	<0.001

YLDs	Gabon	1995-2010	-0.37 (-0.46 to -0.2)	0.010
YLDs	Gabon	2010-2016	-2.01 (-2.91 to -1.64)	0.000
YLDs	Gabon	2016-2021	-0.07 (-0.58 to 1.03)	0.710
YLDs	Gambia	1990-1996	-3.2 (-3.4 to -2.97)	<0.001
YLDs	Gambia	1996-2002	0.32 (0.14 to 0.61)	0.011
YLDs	Gambia	2002-2005	-1.4 (-1.78 to -0.75)	0.005
YLDs	Gambia	2005-2010	0.17 (-0.15 to 0.81)	0.170
YLDs	Gambia	2010-2015	-2.32 (-2.81 to -1.95)	0.001
YLDs	Gambia	2015-2021	-0.21 (-0.47 to 0.12)	0.127
YLDs	Georgia	1990-1994	7.63 (5.49 to 10.36)	<0.001
YLDs	Georgia	1994-1998	-4.5 (-6.94 to -2.15)	0.000
YLDs	Georgia	1998-2009	-0.42 (-0.93 to 2.15)	0.440
YLDs	Georgia	2009-2021	-2.82 (-3.44 to -2.35)	0.003

YLDs	Germany	1990-1992	-1.44 (-1.85 to -0.78)	<0.001
YLDs	Germany	1992-2000	0.53 (0.36 to 0.65)	<0.001
YLDs	Germany	2000-2008	0.95 (0.85 to 1.2)	0.001
YLDs	Germany	2008-2011	-0.93 (-1.4 to -0.15)	0.035
YLDs	Germany	2011-2019	-2.36 (-2.48 to -2.28)	<0.001
YLDs	Germany	2019-2021	0.57 (0 to 1.02)	0.050
YLDs	Ghana	1990-1993	-0.85 (-1.82 to -0.21)	0.012
YLDs	Ghana	1993-2002	0.92 (0.73 to 1.23)	0.001
YLDs	Ghana	2002-2011	0.08 (-0.17 to 0.85)	0.318
YLDs	Ghana	2011-2014	-3.05 (-3.36 to 0.23)	0.122
YLDs	Ghana	2014-2019	-0.65 (-3.21 to -0.37)	0.001
YLDs	Ghana	2019-2021	0.87 (-0.33 to 1.52)	0.156
YLDs	Greece	1990-2000	-0.36 (-0.51 to -0.25)	0.021
YLDs	Greece	2000-2004	-1.23 (-1.5 to -0.21)	0.003

YLDs	Greece	2004-2010	-0.89 (-1.12 to -0.64)	<0.001
YLDs	Greece	2010-2015	-2.72 (-2.97 to -2.56)	<0.001
YLDs	Greece	2015-2019	-0.86 (-1.35 to -0.65)	<0.001
YLDs	Greece	2019-2021	0.69 (0.07 to 1.09)	0.032
YLDs	Greenland	1990-1995	-1.03 (-1.23 to -0.61)	0.002
YLDs	Greenland	1995-2005	-1.53 (-1.67 to -1.46)	<0.001
YLDs	Greenland	2005-2010	-3.6 (-3.81 to -3.4)	<0.001
YLDs	Greenland	2010-2014	-1.34 (-1.63 to -0.94)	<0.001
YLDs	Greenland	2014-2018	-2.68 (-3.13 to -2.36)	<0.001
YLDs	Greenland	2018-2021	-0.58 (-1.01 to 0.11)	0.069
YLDs	Grenada	1990-1993	-2.7 (-3.87 to -0.82)	0.028
YLDs	Grenada	1993-1998	-5.63 (-6.95 to -5.11)	0.001
YLDs	Grenada	1998-2003	-0.53 (-1.96 to 0.28)	0.092

YLDs	Grenada	2003-2009	1.2<br>(0.63 to 2.33)	0.029
YLDs	Grenada	2009-2021	-1.39<br>(-1.57 to -1.22)	0.001
YLDs	Guam	1990-1994	-0.55<br>(-1.01 to -0.3)	<0.001
YLDs	Guam	1994-2000	0.07<br>(-0.08 to 0.43)	0.281
YLDs	Guam	2000-2005	1.47<br>(1.25 to 1.78)	<0.001
YLDs	Guam	2005-2010	0.36<br>(0.14 to 0.53)	0.014
YLDs	Guam	2010-2015	-2.67<br>(-2.82 to -2.52)	<0.001
YLDs	Guam	2015-2021	-0.78<br>(-0.93 to -0.64)	<0.001
YLDs	Guatemala	1990-1997	-6.5<br>(-6.99 to -6.14)	<0.001
YLDs	Guatemala	1997-2003	-3.89<br>(-4.28 to -3.43)	<0.001
YLDs	Guatemala	2003-2012	-0.41<br>(-0.6 to -0.14)	0.024
YLDs	Guatemala	2012-2017	-2.45<br>(-3.23 to -2.03)	<0.001
YLDs	Guatemala	2017-2021	1.28<br>(0.68 to 2.15)	0.000

YLDs	Guinea	1990-1998	-0.72<br>(-1.67 to -0.12)	0.023
YLDs	Guinea	1998-2001	8.31<br>(4.87 to 9.58)	<0.001
YLDs	Guinea	2001-2021	-1.12<br>(-1.3 to -0.95)	<0.001
YLDs	Guinea-Bissau	1990-1996	-0.37<br>(-3.72 to 1.31)	0.556
YLDs	Guinea-Bissau	1996-1999	15.63<br>(8.25 to 18.25)	0.001
YLDs	Guinea-Bissau	1999-2021	-3.3<br>(-3.61 to -3.03)	<0.001
YLDs	Guyana	1990-2001	-0.55<br>(-0.69 to -0.43)	0.008
YLDs	Guyana	2001-2006	2.12<br>(0.08 to 2.49)	0.048
YLDs	Guyana	2006-2009	4.37<br>(2.41 to 4.83)	0.002
YLDs	Guyana	2009-2019	-2.54<br>(-2.8 to 3.83)	0.056
YLDs	Guyana	2019-2021	-1.08<br>(-2.38 to -0.42)	0.002
YLDs	Haiti	1990-2009	0.57<br>(-0.19 to 1.23)	0.128
YLDs	Haiti	2009-2012	111.27<br>(70.81 to 130.05)	<0.001

YLDs	Haiti	2012-2021	-9.71 (-14.81 to -5.52)	<0.001
YLDs	Honduras	1990-1997	1.16 (-0.02 to 2.07)	0.054
YLDs	Honduras	1997-2000	18.34 (12.13 to 20.53)	0.007
YLDs	Honduras	2000-2015	-2.8 (-3.3 to -2.18)	0.015
YLDs	Honduras	2015-2019	-8.89 (-11.89 to -6.16)	0.008
YLDs	Honduras	2019-2021	3.02 (-3.29 to 8.53)	0.264
YLDs	Hungary	1990-2000	-0.45 (-0.65 to -0.33)	<0.001
YLDs	Hungary	2000-2010	0.03 (-0.07 to 0.23)	0.516
YLDs	Hungary	2010-2018	-3.22 (-3.37 to -3.09)	<0.001
YLDs	Hungary	2018-2021	-0.04 (-0.67 to 0.52)	0.874
YLDs	Iceland	1990-2010	0.39 (0.32 to 0.47)	<0.001
YLDs	Iceland	2010-2016	-3.15 (-3.84 to -2.73)	<0.001
YLDs	Iceland	2016-2021	-0.58 (-1.13 to 0.18)	0.106

YLDs	India	1990-1997	-0.14<br>(-0.61 to 0.03)	0.087
YLDs	India	1997-2002	0.34<br>(-1.09 to 0.74)	0.068
YLDs	India	2002-2010	-1.38<br>(-1.53 to -1.2)	0.002
YLDs	India	2010-2014	-3.87<br>(-4.28 to -3.49)	<0.001
YLDs	India	2014-2018	-2.34<br>(-2.89 to -1.76)	<0.001
YLDs	India	2018-2021	-0.19<br>(-0.68 to 0.65)	0.384
YLDs	Indonesia	1990-2002	-2.47<br>(-2.9 to -2.05)	<0.001
YLDs	Indonesia	2002-2006	8.47<br>(5.73 to 11.99)	<0.001
YLDs	Indonesia	2006-2021	-3.14<br>(-3.46 to -2.86)	<0.001
YLDs	Iran (Islamic Republic of)	1990-1993	-2.33<br>(-3.34 to -1.54)	<0.001
YLDs	Iran (Islamic Republic of)	1993-2005	-1.23<br>(-1.34 to -0.99)	<0.001
YLDs	Iran (Islamic Republic of)	2005-2009	-4.85<br>(-5.32 to -3.91)	<0.001
YLDs	Iran (Islamic Republic of)	2009-2015	-7.17<br>(-7.72 to -6.87)	<0.001

YLDs	Iran (Islamic Republic of)	2015-2018	-5.11 (-6.2 to -3.82)	<0.001
YLDs	Iran (Islamic Republic of)	2018-2021	-0.5 (-1.24 to 0.55)	0.195
YLDs	Iraq	1990-1992	-20.95 (-23.6 to -16.68)	<0.001
YLDs	Iraq	1992-2002	-3.67 (-4.09 to -3.19)	<0.001
YLDs	Iraq	2002-2008	20.54 (19.29 to 21.61)	<0.001
YLDs	Iraq	2008-2012	-10.19 (-13.46 to -8.07)	<0.001
YLDs	Iraq	2012-2017	8.61 (6.17 to 12.09)	<0.001
YLDs	Iraq	2017-2021	-12.69 (-15.4 to -9.7)	<0.001
YLDs	Ireland	1990-1993	-0.41 (-0.92 to -0.07)	0.032
YLDs	Ireland	1993-1997	0.41 (0.14 to 0.72)	0.029
YLDs	Ireland	1997-2009	-0.44 (-0.5 to -0.38)	0.000
YLDs	Ireland	2009-2018	-1.42 (-1.51 to -1.33)	<0.001
YLDs	Ireland	2018-2021	0.89 (0.6 to 1.13)	<0.001

YLDs	Israel	1990-2000	-1.01 (-1.32 to -0.76)	0.002
YLDs	Israel	2000-2003	3.91 (2.22 to 4.67)	0.006
YLDs	Israel	2003-2009	0.19 (-0.69 to 0.77)	0.640
YLDs	Israel	2009-2021	-2.29 (-2.55 to -2.08)	<0.001
YLDs	Italy	1990-1996	-2.04 (-2.18 to -1.94)	<0.001
YLDs	Italy	1996-2004	-1.39 (-1.49 to -1.3)	<0.001
YLDs	Italy	2004-2010	-1 (-1.09 to -0.85)	<0.001
YLDs	Italy	2010-2015	2.26 (2.16 to 2.36)	<0.001
YLDs	Italy	2015-2019	-0.53 (-0.7 to -0.4)	<0.001
YLDs	Italy	2019-2021	0.93 (0.42 to 1.3)	<0.001
YLDs	Jamaica	1990-1997	-1.41 (-1.71 to -1.19)	<0.001
YLDs	Jamaica	1997-2011	0.26 (0.17 to 0.36)	<0.001
YLDs	Jamaica	2011-2017	-2.18 (-2.41 to -1.89)	<0.001

YLDs	Jamaica	2017-2021	3.94 (3.52 to 4.33)	<0.001
YLDs	Japan	1990-2003	-1.18 (-1.23 to -1.13)	<0.001
YLDs	Japan	2003-2011	-0.61 (-0.69 to -0.5)	<0.001
YLDs	Japan	2011-2015	-3.6 (-3.83 to -3.45)	<0.001
YLDs	Japan	2015-2018	-2.76 (-3.04 to -2.51)	<0.001
YLDs	Japan	2018-2021	-0.28 (-0.55 to 0.01)	0.054
YLDs	Jordan	1990-1998	-0.3 (-0.48 to 0.09)	0.083
YLDs	Jordan	1998-2006	-0.86 (-1.38 to -0.66)	<0.001
YLDs	Jordan	2006-2015	-2.24 (-2.5 to -2.07)	0.007
YLDs	Jordan	2015-2021	0.63 (0.32 to 1.05)	0.003
YLDs	Kazakhstan	1990-2004	0.12 (0.01 to 0.23)	0.042
YLDs	Kazakhstan	2004-2008	-2.26 (-2.93 to -0.49)	0.044
YLDs	Kazakhstan	2008-2015	-4.98 (-5.62 to -2.88)	<0.001

YLDs	Kazakhstan	2015-2018	-2.64 (-4.66 to -1.14)	<0.001
YLDs	Kazakhstan	2018-2021	0.68 (-0.31 to 2.08)	0.115
YLDs	Kenya	1990-1996	0.25 (-0.31 to 0.47)	0.150
YLDs	Kenya	1996-2002	1.15 (0.91 to 1.77)	<0.001
YLDs	Kenya	2002-2006	-0.47 (-1.11 to -0.12)	0.013
YLDs	Kenya	2006-2009	2.48 (2.02 to 2.91)	<0.001
YLDs	Kenya	2009-2014	-2.62 (-3.05 to -2.35)	<0.001
YLDs	Kenya	2014-2021	-0.66 (-0.84 to -0.41)	0.000
YLDs	Kiribati	1990-1993	1.89 (-1.8 to 3.74)	0.163
YLDs	Kiribati	1993-1996	10.22 (7.91 to 11.99)	0.002
YLDs	Kiribati	1996-1999	-6.29 (-7.54 to -3.93)	0.001
YLDs	Kiribati	1999-2011	-1.74 (-1.95 to 0.28)	0.062
YLDs	Kiribati	2011-2016	-3.32 (-5.17 to -2.39)	0.000

YLDs	Kiribati	2016-2021	-0.43 (-1.38 to 2.32)	0.522
YLDs	Kuwait	1990-1992	38.86 (31.26 to 45.67)	<0.001
YLDs	Kuwait	1992-1995	-14.72 (-16.99 to -11.22)	<0.001
YLDs	Kuwait	1995-2008	-4.73 (-5.2 to -4.24)	<0.001
YLDs	Kuwait	2008-2021	-1.48 (-1.88 to -0.97)	<0.001
YLDs	Kyrgyzstan	1990-1999	-1.28 (-2.15 to -0.93)	0.025
YLDs	Kyrgyzstan	1999-2003	-0.36 (-1.96 to 0.28)	0.243
YLDs	Kyrgyzstan	2003-2011	-2.11 (-2.6 to -0.56)	0.017
YLDs	Kyrgyzstan	2011-2015	-5.69 (-6.63 to -2.07)	<0.001
YLDs	Kyrgyzstan	2015-2018	-2.48 (-5.44 to -0.94)	0.002
YLDs	Kyrgyzstan	2018-2021	0.89 (-0.31 to 2.62)	0.100
YLDs	Lao People's Democratic Republic	1990-1992	-0.04 (-1.34 to 1.02)	0.863
YLDs	Lao People's Democratic Republic	1992-1995	-3.08 (-3.54 to -2.21)	<0.001

YLDs	Lao People's Democratic Republic	1995-2004	-1.25 (-1.49 to -0.98)	0.014
YLDs	Lao People's Democratic Republic	2004-2007	0.31 (-0.7 to 0.69)	0.451
YLDs	Lao People's Democratic Republic	2007-2018	-2.66 (-2.83 to -2.53)	<0.001
YLDs	Lao People's Democratic Republic	2018-2021	0.79 (-0.13 to 2.2)	0.080
YLDs	Latvia	1990-2001	0.25 (0.09 to 0.39)	0.012
YLDs	Latvia	2001-2009	-2.34 (-2.61 to -2.02)	0.020
YLDs	Latvia	2009-2015	-5.52 (-6.05 to -4.91)	<0.001
YLDs	Latvia	2015-2018	-2.37 (-4.91 to -1.54)	<0.001
YLDs	Latvia	2018-2021	0.85 (-0.13 to 2.21)	0.077
YLDs	Lebanon	1990-1995	-7.97 (-10.76 to -5.42)	<0.001
YLDs	Lebanon	1995-2001	-22.02 (-24.48 to -20.8)	<0.001
YLDs	Lebanon	2001-2004	-10.56 (-16.63 to -5.1)	<0.001
YLDs	Lebanon	2004-2008	2.05 (0.43 to 4.79)	0.011

YLDs	Lebanon	2008-2012	-5.3<br>(-7.22 to -3.62)	<0.001
YLDs	Lebanon	2012-2021	-1.46<br>(-1.95 to -0.43)	0.026
YLDs	Lesotho	1990-1997	1.38<br>(0.8 to 1.68)	0.009
YLDs	Lesotho	1997-2000	4.02<br>(2.65 to 4.63)	0.005
YLDs	Lesotho	2000-2021	-0.55<br>(-0.63 to -0.48)	0.001
YLDs	Liberia	1990-1996	18.72<br>(16.34 to 21.26)	<0.001
YLDs	Liberia	1996-2000	-16.78<br>(-21.78 to -13.15)	0.004
YLDs	Liberia	2000-2004	8.01<br>(3.39 to 15.07)	0.005
YLDs	Liberia	2004-2008	-17.1<br>(-21.2 to -13.35)	0.004
YLDs	Liberia	2008-2017	-8.68<br>(-9.96 to -7.4)	<0.001
YLDs	Liberia	2017-2021	-1.97<br>(-4.62 to 2.91)	0.260
YLDs	Libya	1990-1994	-5.21<br>(-11.89 to -2.28)	0.012
YLDs	Libya	1994-1997	7.98<br>(1.75 to 11.17)	0.020

YLDs	Libya	1997-2005	-4.95 (-6.87 to -3.76)	0.016
YLDs	Libya	2005-2012	15.94 (14.2 to 18.1)	0.003
YLDs	Libya	2012-2021	2.79 (1.36 to 4.06)	0.005
YLDs	Lithuania	1990-1994	-1.02 (-1.92 to -0.58)	<0.001
YLDs	Lithuania	1994-2003	0.1 (-0.04 to 0.42)	0.121
YLDs	Lithuania	2003-2010	-0.81 (-1.03 to -0.55)	0.019
YLDs	Lithuania	2010-2014	-4.71 (-5.14 to -1.32)	0.000
YLDs	Lithuania	2014-2018	-3.71 (-4.2 to -3.03)	<0.001
YLDs	Lithuania	2018-2021	-0.2 (-0.79 to 0.64)	0.418
YLDs	Luxembourg	1990-1993	-1.96 (-2.55 to -1.5)	<0.001
YLDs	Luxembourg	1993-2000	-1.2 (-1.3 to -0.33)	0.041
YLDs	Luxembourg	2000-2010	0.79 (0.71 to 0.87)	0.016
YLDs	Luxembourg	2010-2017	-2.06 (-2.21 to -1.94)	<0.001

YLDs	Luxembourg	2017-2021	-0.56<br>(-0.9 to -0.07)	0.029
YLDs	Madagascar	1990-1999	-1.28<br>(-1.5 to -1.18)	<0.001
YLDs	Madagascar	1999-2003	-0.46<br>(-1.03 to 0.05)	0.073
YLDs	Madagascar	2003-2010	0.47<br>(0.33 to 0.8)	0.020
YLDs	Madagascar	2010-2013	-1.68<br>(-1.92 to -1.08)	0.017
YLDs	Madagascar	2013-2019	-0.65<br>(-0.88 to -0.32)	0.003
YLDs	Madagascar	2019-2021	1.76<br>(0.91 to 2.22)	<0.001
YLDs	Malawi	1990-2000	-0.73<br>(-0.94 to -0.58)	0.009
YLDs	Malawi	2000-2011	-0.24<br>(-0.66 to 0.06)	0.081
YLDs	Malawi	2011-2014	-1.32<br>(-1.64 to -0.18)	0.024
YLDs	Malawi	2014-2019	0.33<br>(-1.14 to 0.65)	0.251
YLDs	Malawi	2019-2021	1.96<br>(0.82 to 2.6)	<0.001
YLDs	Malaysia	1990-2000	0.37<br>(0.22 to 0.79)	0.002

YLDs	Malaysia	2000-2010	0.03<br>(-1.6 to 0.16)	0.845
YLDs	Malaysia	2010-2018	-2.25<br>(-2.78 to -2)	0.000
YLDs	Malaysia	2018-2021	-0.3<br>(-1.14 to 0.84)	0.396
YLDs	Maldives	1990-2002	-1.47<br>(-2.18 to -0.83)	<0.001
YLDs	Maldives	2002-2005	20.97<br>(13.8 to 23.56)	<0.001
YLDs	Maldives	2005-2021	-2.92<br>(-3.45 to -2.48)	<0.001
YLDs	Mali	1990-1996	0.62<br>(-0.35 to 1.55)	0.170
YLDs	Mali	1996-1999	34.56<br>(32.16 to 37.06)	0.003
YLDs	Mali	1999-2011	-6.26<br>(-7.09 to -5.76)	0.008
YLDs	Mali	2011-2017	-2.06<br>(-4.3 to -0.35)	0.032
YLDs	Mali	2017-2021	3.96<br>(1.86 to 8.01)	0.000
YLDs	Malta	1990-1999	0.71<br>(0.51 to 0.86)	<0.001
YLDs	Malta	1999-2005	3.04<br>(2.7 to 3.42)	<0.001

YLDs	Malta	2005-2011	-0.18 (-0.41 to 0.06)	0.095
YLDs	Malta	2011-2019	-4.71 (-4.87 to -4.57)	<0.001
YLDs	Malta	2019-2021	0.42 (-0.58 to 1.14)	0.296
YLDs	Marshall Islands	1990-1999	1.29 (1.13 to 1.46)	<0.001
YLDs	Marshall Islands	1999-2009	-0.27 (-0.43 to -0.12)	0.002
YLDs	Marshall Islands	2009-2017	-1.71 (-2.07 to -1.51)	<0.001
YLDs	Marshall Islands	2017-2021	0.2 (-0.39 to 1.16)	0.354
YLDs	Mauritania	1990-1999	-0.34 (-0.57 to -0.23)	0.014
YLDs	Mauritania	1999-2006	0.04 (-0.27 to 0.39)	0.578
YLDs	Mauritania	2006-2011	-1.02 (-1.31 to 0.05)	0.069
YLDs	Mauritania	2011-2015	-2.86 (-3.23 to -1.05)	<0.001
YLDs	Mauritania	2015-2018	-1.16 (-2.77 to -0.76)	<0.001
YLDs	Mauritania	2018-2021	0.01 (-0.39 to 0.61)	0.863

YLDs	Mauritius	1990-2004	-0.83<br>(-0.94 to -0.74)	<0.001
YLDs	Mauritius	2004-2009	0.75<br>(0.3 to 1.65)	0.000
YLDs	Mauritius	2009-2021	-0.89<br>(-1.02 to -0.77)	<0.001
YLDs	Mexico	1990-1994	-8.19<br>(-9.21 to -7.14)	<0.001
YLDs	Mexico	1994-2006	1.96<br>(1.56 to 2.26)	0.002
YLDs	Mexico	2006-2009	8.1<br>(5.52 to 9.48)	0.002
YLDs	Mexico	2009-2012	-2.61<br>(-6.03 to -0.81)	0.032
YLDs	Mexico	2012-2019	-9.43<br>(-10.46 to -8.95)	<0.001
YLDs	Mexico	2019-2021	1.97<br>(-1.65 to 4.34)	0.176
YLDs	Micronesia<br>(Federated States of)	1990-2001	0.04<br>(-0.35 to 0.37)	0.816
YLDs	Micronesia<br>(Federated States of)	2001-2004	16.33<br>(12.31 to 17.62)	<0.001
YLDs	Micronesia<br>(Federated States of)	2004-2021	-2.18<br>(-2.41 to -1.94)	<0.001
YLDs	Monaco	1990-1992	-1.29<br>(-1.7 to -0.56)	0.000

YLDs	Monaco	1992-1998	0.23 (0.01 to 0.49)	0.040
YLDs	Monaco	1998-2010	1.14 (1.09 to 1.2)	0.001
YLDs	Monaco	2010-2019	-2.14 (-2.2 to -2.07)	<0.001
YLDs	Monaco	2019-2021	1.35 (0.89 to 1.72)	0.001
YLDs	Mongolia	1990-1993	-1.2 (-2.6 to -0.41)	0.004
YLDs	Mongolia	1993-2003	1.26 (1.09 to 1.49)	<0.001
YLDs	Mongolia	2003-2009	-0.54 (-0.87 to -0.14)	0.043
YLDs	Mongolia	2009-2016	-4.47 (-4.8 to -0.75)	0.003
YLDs	Mongolia	2016-2019	-1.81 (-4.42 to -1.37)	<0.001
YLDs	Mongolia	2019-2021	1.67 (0.21 to 2.67)	0.020
YLDs	Montenegro	1990-1993	-0.43 (-1.02 to 0.02)	0.060
YLDs	Montenegro	1993-2003	0.35 (0.24 to 0.72)	0.002
YLDs	Montenegro	2003-2006	-0.67 (-1.22 to 0.45)	0.230

YLDs	Montenegro	2006-2010	-1.4 (-2.67 to -1.05)	<0.001
YLDs	Montenegro	2010-2017	-2.4 (-2.62 to -2.08)	<0.001
YLDs	Montenegro	2017-2021	-0.18 (-0.45 to 0.25)	0.144
YLDs	Morocco	1990-1993	-1.48 (-2.1 to -0.92)	<0.001
YLDs	Morocco	1993-2001	-0.84 (-1.91 to -0.4)	0.000
YLDs	Morocco	2001-2010	-1.73 (-3.62 to -1.54)	<0.001
YLDs	Morocco	2010-2017	-3.55 (-3.75 to -3.25)	<0.001
YLDs	Morocco	2017-2021	-0.34 (-0.68 to 0.08)	0.064
YLDs	Mozambique	1990-1996	-11.39 (-12.01 to -10.51)	<0.001
YLDs	Mozambique	1996-2001	-14.71 (-15.37 to -14.2)	<0.001
YLDs	Mozambique	2001-2005	-6.95 (-7.41 to -6.48)	<0.001
YLDs	Mozambique	2005-2011	-1.54 (-2.2 to -1.21)	<0.001
YLDs	Mozambique	2011-2018	-0.43 (-0.69 to 0.1)	0.075

YLDs	Mozambique	2018-2021	5.81<br>(5.08 to 6.5)	<0.001
YLDs	Myanmar	1990-2006	-3.8<br>(-5.36 to -2.31)	<0.001
YLDs	Myanmar	2006-2009	47.85<br>(27.32 to 56.36)	<0.001
YLDs	Myanmar	2009-2021	-1.61<br>(-4.27 to 0.36)	0.082
YLDs	Namibia	1990-1993	-10.72<br>(-12.71 to -9.38)	<0.001
YLDs	Namibia	1993-1999	-7.63<br>(-8.02 to -6.84)	<0.001
YLDs	Namibia	1999-2005	-3.73<br>(-4.11 to -3.34)	0.001
YLDs	Namibia	2005-2012	-0.17<br>(-0.4 to 0.19)	0.226
YLDs	Namibia	2012-2015	-3.12<br>(-3.58 to -2.17)	0.001
YLDs	Namibia	2015-2021	-0.72<br>(-1 to -0.21)	0.018
YLDs	Nauru	1990-2004	1.58<br>(1.51 to 1.66)	<0.001
YLDs	Nauru	2004-2012	-1.07<br>(-1.19 to -0.82)	0.002
YLDs	Nauru	2012-2015	-2.59<br>(-2.96 to -1.85)	<0.001

YLDs	Nauru	2015-2021	-0.49 (-0.74 to -0.05)	0.037
YLDs	Nepal	1990-2000	1.26 (0.24 to 2.04)	0.028
YLDs	Nepal	2000-2004	18.31 (12.13 to 24.17)	0.007
YLDs	Nepal	2004-2013	-6.26 (-7.71 to -4.55)	0.038
YLDs	Nepal	2013-2016	7.57 (-4.44 to 10.34)	0.059
YLDs	Nepal	2016-2021	-2.37 (-6.58 to 0.56)	0.062
YLDs	Netherlands	1990-1994	-2.74 (-3.34 to -2.41)	<0.001
YLDs	Netherlands	1994-2000	-2.02 (-2.29 to -1.46)	<0.001
YLDs	Netherlands	2000-2003	-0.85 (-1.6 to 0.31)	0.125
YLDs	Netherlands	2003-2016	0.28 (0.13 to 0.46)	0.037
YLDs	Netherlands	2016-2019	1.67 (0.2 to 1.92)	0.027
YLDs	Netherlands	2019-2021	0.14 (-0.49 to 1.06)	0.529
YLDs	New Zealand	1990-1994	1.99 (1.27 to 2.34)	<0.001

YLDs	New Zealand	1994-2000	2.76 (2.52 to 3.22)	<0.001
YLDs	New Zealand	2000-2005	0.97 (0.66 to 1.21)	0.024
YLDs	New Zealand	2005-2013	-2.13 (-2.27 to -1.99)	<0.001
YLDs	New Zealand	2013-2019	-3.28 (-3.58 to -3.08)	<0.001
YLDs	New Zealand	2019-2021	-0.11 (-0.95 to 0.46)	0.710
YLDs	Nicaragua	1990-1992	-14.61 (-16.2 to -11.18)	<0.001
YLDs	Nicaragua	1992-2002	-9.18 (-9.5 to -8.7)	<0.001
YLDs	Nicaragua	2002-2006	-4.62 (-6.46 to -3.39)	<0.001
YLDs	Nicaragua	2006-2010	-1.58 (-2.36 to -0.53)	0.001
YLDs	Nicaragua	2010-2018	-3.47 (-4.03 to -3.22)	<0.001
YLDs	Nicaragua	2018-2021	0.87 (-0.2 to 3.15)	0.102
YLDs	Niger	1990-1999	-0.27 (-0.46 to -0.01)	0.046
YLDs	Niger	1999-2003	-2.01 (-2.77 to -1.31)	0.000

YLDs	Niger	2003-2014	-0.14 (-0.34 to 0.1)	0.158
YLDs	Niger	2014-2021	2.7 (2.38 to 3.19)	<0.001
YLDs	Nigeria	1990-1998	0.02 (-0.14 to 0.15)	0.831
YLDs	Nigeria	1998-2001	2.17 (1.38 to 2.45)	0.007
YLDs	Nigeria	2001-2013	-0.4 (-0.51 to -0.32)	0.015
YLDs	Nigeria	2013-2016	1.2 (0.55 to 1.54)	0.028
YLDs	Nigeria	2016-2019	-2.18 (-2.61 to -1.66)	0.028
YLDs	Nigeria	2019-2021	0.06 (-0.78 to 0.8)	0.859
YLDs	Niue	1990-2003	1.2 (0.85 to 1.49)	<0.001
YLDs	Niue	2003-2006	15.07 (11.34 to 16.47)	<0.001
YLDs	Niue	2006-2021	-2.25 (-2.56 to -1.93)	<0.001
YLDs	North Macedonia	1990-1999	0.45 (0.05 to 0.6)	0.041
YLDs	North Macedonia	1999-2002	1.32 (-0.26 to 1.6)	0.068

YLDs	North Macedonia	2002-2010	-0.38<br>(-1.3 to -0.24)	0.010
YLDs	North Macedonia	2010-2016	-3.37<br>(-3.66 to -3.08)	<0.001
YLDs	North Macedonia	2016-2021	-0.98<br>(-1.31 to -0.48)	0.003
YLDs	Northern Mariana Islands	1990-1994	-3.31<br>(-5.35 to -2.28)	<0.001
YLDs	Northern Mariana Islands	1994-2003	-0.73<br>(-1.37 to 0.42)	0.074
YLDs	Northern Mariana Islands	2003-2006	6.79<br>(-1.27 to 7.67)	0.160
YLDs	Northern Mariana Islands	2006-2009	2.2<br>(0.26 to 6.41)	0.022
YLDs	Northern Mariana Islands	2009-2021	0.36<br>(-0.5 to 0.69)	0.087
YLDs	Norway	1990-1994	-1.76<br>(-2.32 to -1.46)	<0.001
YLDs	Norway	1994-1999	-1<br>(-1.17 to -0.02)	0.047
YLDs	Norway	1999-2008	1.24<br>(1.15 to 1.39)	0.001
YLDs	Norway	2008-2011	-0.08<br>(-0.37 to 0.32)	0.669
YLDs	Norway	2011-2017	-3.79<br>(-3.95 to -3.65)	<0.001

YLDs	Norway	2017-2021	-0.44<br>(-0.68 to -0.2)	0.002
YLDs	Oman	1990-1993	-2.07<br>(-2.95 to -1.49)	<0.001
YLDs	Oman	1993-2007	-0.79<br>(-0.86 to -0.57)	0.000
YLDs	Oman	2007-2010	-1.72<br>(-2.88 to -0.85)	<0.001
YLDs	Oman	2010-2016	-3.94<br>(-4.5 to -3.34)	<0.001
YLDs	Oman	2016-2019	-2.03<br>(-3.65 to -1.61)	<0.001
YLDs	Oman	2019-2021	1.15<br>(-0.15 to 2.04)	0.076
YLDs	Pakistan	1990-2004	0.75<br>(0.2 to 1.2)	0.026
YLDs	Pakistan	2004-2007	12.7<br>(0.65 to 14.97)	0.038
YLDs	Pakistan	2007-2012	5.06<br>(-4.33 to 8.42)	0.082
YLDs	Pakistan	2012-2021	-5.32<br>(-6.48 to -4.3)	0.004
YLDs	Palau	1990-1994	-2.5<br>(-3.04 to -2.2)	<0.001
YLDs	Palau	1994-1998	-0.56<br>(-1.21 to 0.02)	0.055
YLDs	Palau	1998-2006	0.82<br>(0.69 to 1.11)	0.001

YLDs	Palau	2006-2010	-0.3<br>(-0.75 to 0.19)	0.138
YLDs	Palau	2010-2014	-1.81<br>(-2.27 to -1.45)	0.002
YLDs	Palau	2014-2021	0.23<br>(0.08 to 0.41)	0.006
YLDs	Palestine	1990-1993	-8.16<br>(-10.79 to -4.04)	<0.001
YLDs	Palestine	1993-1999	-14.58<br>(-15.66 to -13.88)	0.000
YLDs	Palestine	1999-2003	19.31<br>(17.88 to 20.84)	0.002
YLDs	Palestine	2003-2010	5.82<br>(5.04 to 6.68)	0.000
YLDs	Palestine	2010-2015	-13.07<br>(-14.62 to -12.07)	0.015
YLDs	Palestine	2015-2021	-10.3<br>(-11.01 to -8.82)	<0.001
YLDs	Panama	1990-1994	-5.2<br>(-6.3 to -4.59)	<0.001
YLDs	Panama	1994-2002	-1.56<br>(-2.04 to -1.37)	<0.001
YLDs	Panama	2002-2010	-0.76<br>(-0.96 to -0.13)	0.034
YLDs	Panama	2010-2018	-2.01<br>(-2.41 to -1.83)	<0.001

YLDs	Panama	2018-2021	0.1 (-0.69 to 1.23)	0.694
YLDs	Papua New Guinea	1990-1997	2.85 (1.32 to 4.16)	0.019
YLDs	Papua New Guinea	1997-2000	18.95 (3.27 to 21.52)	0.017
YLDs	Papua New Guinea	2000-2015	-2.72 (-5.62 to 18.12)	0.078
YLDs	Papua New Guinea	2015-2018	-8.61 (-10.36 to -0.25)	0.042
YLDs	Papua New Guinea	2018-2021	-0.82 (-5.4 to 5.06)	0.620
YLDs	Paraguay	1990-1994	-0.64 (-0.99 to -0.39)	<0.001
YLDs	Paraguay	1994-2003	0.27 (0.19 to 0.41)	0.002
YLDs	Paraguay	2003-2011	-0.21 (-0.32 to -0.11)	0.006
YLDs	Paraguay	2011-2015	-4.05 (-4.29 to -3.86)	0.000
YLDs	Paraguay	2015-2018	-2.18 (-2.91 to -1.83)	<0.001
YLDs	Paraguay	2018-2021	-0.56 (-0.88 to -0.03)	0.040
YLDs	Peru	1990-1992	0.81 (-0.77 to 2.22)	0.170

YLDs	Peru	1992-1997	-6.6 (-7.28 to -6.19)	<0.001
YLDs	Peru	1997-2004	-4.02 (-4.38 to -3.63)	<0.001
YLDs	Peru	2004-2011	-1.82 (-2.07 to -1.08)	0.000
YLDs	Peru	2011-2016	-2.95 (-3.86 to -2.52)	<0.001
YLDs	Peru	2016-2021	-1.21 (-1.65 to -0.21)	0.036
YLDs	Philippines	1990-1999	-4.51 (-5.05 to -4.05)	<0.001
YLDs	Philippines	1999-2012	-1.48 (-1.74 to -1.23)	<0.001
YLDs	Philippines	2012-2018	2.46 (1.74 to 3.97)	0.001
YLDs	Philippines	2018-2021	-2.08 (-4.54 to -0.43)	0.008
YLDs	Poland	1990-2000	-0.07 (-0.15 to 0)	0.061
YLDs	Poland	2000-2010	-1.49 (-1.56 to -1.41)	0.002
YLDs	Poland	2010-2015	-4.78 (-5.03 to -4.64)	<0.001
YLDs	Poland	2015-2018	-3.19 (-3.64 to -2.84)	<0.001
YLDs	Poland	2018-2021	0.09 (-0.32 to 0.5)	0.573

YLDs	Portugal	1990-1994	-2.4 (-2.74 to -1.96)	<0.001
YLDs	Portugal	1994-2002	-3.75 (-4.12 to -3.56)	<0.001
YLDs	Portugal	2002-2013	-2.09 (-3.74 to -1.93)	<0.001
YLDs	Portugal	2013-2018	-1.55 (-2.15 to -1.14)	<0.001
YLDs	Portugal	2018-2021	-0.32 (-1.05 to 0.45)	0.260
YLDs	Puerto Rico	1990-2010	-0.22 (-0.29 to -0.15)	0.003
YLDs	Puerto Rico	2010-2016	-1.38 (-1.96 to -1)	0.003
YLDs	Puerto Rico	2016-2019	5.97 (4.96 to 6.78)	0.014
YLDs	Puerto Rico	2019-2021	0.79 (-0.9 to 2.34)	0.265
YLDs	Qatar	1990-2001	-0.46 (-0.72 to -0.33)	0.007
YLDs	Qatar	2001-2006	0.38 (-0.07 to 1.03)	0.082
YLDs	Qatar	2006-2010	-2.19 (-2.76 to -1.26)	0.012
YLDs	Qatar	2010-2018	-3.35 (-3.79 to -3.19)	<0.001
YLDs	Qatar	2018-2021	-0.19 (-1 to 1.15)	0.496

YLDs	Republic of Korea	1990-1999	-2.39 (-2.48 to -2.29)	<0.001
YLDs	Republic of Korea	1999-2004	-3.67 (-3.93 to -3.46)	<0.001
YLDs	Republic of Korea	2004-2010	-0.67 (-0.82 to -0.52)	0.000
YLDs	Republic of Korea	2010-2015	-3.43 (-3.75 to -3.22)	<0.001
YLDs	Republic of Korea	2015-2019	-2.04 (-2.49 to -1.6)	<0.001
YLDs	Republic of Korea	2019-2021	0.78 (-0.01 to 1.32)	0.051
YLDs	Republic of Moldova	1990-1994	0.07 (-0.2 to 0.48)	0.589
YLDs	Republic of Moldova	1994-2002	-0.8 (-0.96 to -0.68)	<0.001
YLDs	Republic of Moldova	2002-2009	-2.04 (-2.2 to -1.89)	<0.001
YLDs	Republic of Moldova	2009-2016	-3.77 (-3.95 to -3.64)	<0.001
YLDs	Republic of Moldova	2016-2019	-1.84 (-2.46 to -1.57)	<0.001
YLDs	Republic of Moldova	2019-2021	1.5 (0.79 to 2.08)	0.003
YLDs	Romania	1990-1995	0.47 (0.31 to 0.65)	0.003

YLDs	Romania	1995-2001	-2.27 (-2.55 to -2.13)	0.010
YLDs	Romania	2001-2005	-1.63 (-1.96 to -1.3)	<0.001
YLDs	Romania	2005-2014	-2.73 (-2.95 to -2.63)	<0.001
YLDs	Romania	2014-2018	-1.98 (-2.49 to -1.46)	<0.001
YLDs	Romania	2018-2021	-0.25 (-0.71 to 0.5)	0.278
YLDs	Russian Federation	1990-2001	1.74 (1.62 to 1.89)	0.000
YLDs	Russian Federation	2001-2004	-2.52 (-3.17 to -0.58)	0.041
YLDs	Russian Federation	2004-2008	-5.17 (-5.89 to -4.46)	0.000
YLDs	Russian Federation	2008-2015	-6.86 (-7.4 to -6.64)	<0.001
YLDs	Russian Federation	2015-2018	-2.81 (-4.9 to -1.82)	0.001
YLDs	Russian Federation	2018-2021	1.31 (0.47 to 2.65)	0.008
YLDs	Rwanda	1990-1993	18.71 (-12.11 to 38.38)	0.141
YLDs	Rwanda	1993-1996	212.8 (118.63 to 265.44)	0.000

YLDs	Rwanda	1996-2015	-19.81 (-22.89 to -18.04)	<0.001
YLDs	Rwanda	2015-2021	-2.22 (-8.78 to 11.21)	0.583
YLDs	Saint Kitts and Nevis	1990-1995	-0.44 (-1.42 to 0.06)	0.068
YLDs	Saint Kitts and Nevis	1995-2010	1.29 (1.17 to 1.43)	0.005
YLDs	Saint Kitts and Nevis	2010-2015	-0.81 (-1.25 to 0.4)	0.073
YLDs	Saint Kitts and Nevis	2015-2019	-2.74 (-3.67 to -2.05)	0.007
YLDs	Saint Kitts and Nevis	2019-2021	0.2 (-1.66 to 1.24)	0.750
YLDs	Saint Lucia	1990-1996	0.41 (-0.2 to 0.63)	0.108
YLDs	Saint Lucia	1996-2011	0.83 (0.77 to 0.95)	0.005
YLDs	Saint Lucia	2011-2018	-1.79 (-2.07 to -1.65)	0.003
YLDs	Saint Lucia	2018-2021	-0.41 (-1.01 to 0.51)	0.264
YLDs	Saint Vincent and the Grenadines	1990-1992	-0.44 (-1.19 to 0.83)	0.367
YLDs	Saint Vincent and the Grenadines	1992-1996	1.47 (-0.99 to 2.12)	0.074

YLDs	Saint Vincent and the Grenadines	1996-2000	-1.18 (-1.91 to 0.99)	0.074
YLDs	Saint Vincent and the Grenadines	2000-2007	1.06 (-0.32 to 1.39)	0.053
YLDs	Saint Vincent and the Grenadines	2007-2021	-0.89 (-0.98 to -0.79)	0.001
YLDs	Samoa	1990-2008	-0.27 (-0.77 to 0.18)	0.214
YLDs	Samoa	2008-2011	43.54 (29.22 to 48.72)	<0.001
YLDs	Samoa	2011-2021	-5.57 (-7.25 to -4.03)	<0.001
YLDs	San Marino	1990-1993	-0.48 (-0.89 to 0.09)	0.081
YLDs	San Marino	1993-1999	-1.05 (-1.46 to -0.31)	0.007
YLDs	San Marino	1999-2005	-0.34 (-0.55 to 1.1)	0.135
YLDs	San Marino	2005-2010	1.05 (-1.13 to 1.31)	0.086
YLDs	San Marino	2010-2018	-1.19 (-1.33 to -1.05)	<0.001
YLDs	San Marino	2018-2021	0.78 (0.38 to 1.49)	0.002
YLDs	Sao Tome and Principe	1990-1996	-1.39 (-1.57 to -1.13)	<0.001

YLDs	Sao Tome and Principe	1996-1999	-3.29 (-3.61 to -2.63)	<0.001
YLDs	Sao Tome and Principe	1999-2006	-1.39 (-1.64 to -1.12)	<0.001
YLDs	Sao Tome and Principe	2006-2018	-0.16 (-0.27 to -0.06)	0.008
YLDs	Sao Tome and Principe	2018-2021	1.56 (0.92 to 2.61)	<0.001
YLDs	Saudi Arabia	1990-1996	-0.21 (-0.45 to 0.12)	0.151
YLDs	Saudi Arabia	1996-1999	-2.64 (-2.99 to -1.78)	0.014
YLDs	Saudi Arabia	1999-2006	-1.11 (-1.54 to -0.61)	0.010
YLDs	Saudi Arabia	2006-2009	0.42 (-0.66 to 0.8)	0.268
YLDs	Saudi Arabia	2009-2019	-2.48 (-2.73 to -2.36)	0.000
YLDs	Saudi Arabia	2019-2021	0.35 (-1.04 to 1.08)	0.451
YLDs	Senegal	1990-1999	1.8 (1.56 to 2.01)	<0.001
YLDs	Senegal	1999-2005	-1.79 (-2.44 to -1.23)	0.042
YLDs	Senegal	2005-2011	-0.98 (-1.32 to -0.22)	0.010

YLDs	Senegal	2011-2014	-3.16 (-3.65 to -2.22)	0.010
YLDs	Senegal	2014-2021	0.02 (-0.25 to 0.39)	0.867
YLDs	Serbia	1990-1992	7.8 (2.33 to 13.1)	0.008
YLDs	Serbia	1992-1997	-4.26 (-6.58 to -3.12)	0.008
YLDs	Serbia	1997-2000	19.6 (15.12 to 22.75)	0.008
YLDs	Serbia	2000-2004	-7.87 (-10.52 to -5.65)	0.004
YLDs	Serbia	2004-2017	-3.11 (-4.27 to -2.32)	0.007
YLDs	Serbia	2017-2021	0.08 (-2.41 to 4.42)	0.953
YLDs	Seychelles	1990-1995	-1.9 (-3.04 to -1.34)	0.001
YLDs	Seychelles	1995-1998	1.76 (0.55 to 2.45)	0.020
YLDs	Seychelles	1998-2009	0.39 (-2.71 to 0.54)	0.192
YLDs	Seychelles	2009-2018	-2.96 (-3.67 to -2.61)	0.000
YLDs	Seychelles	2018-2021	-0.2 (-1.55 to 1.73)	0.719

YLDs	Sierra Leone	1990-2000	16.28 (15.27 to 17.23)	<0.001
YLDs	Sierra Leone	2000-2004	-15.37 (-18.63 to -12.66)	0.006
YLDs	Sierra Leone	2004-2015	-8.65 (-9.34 to -7.77)	0.001
YLDs	Sierra Leone	2015-2021	1.43 (-0.1 to 3.28)	0.057
YLDs	Singapore	1990-1993	-2.63 (-3.65 to -2)	<0.001
YLDs	Singapore	1993-2003	-0.98 (-1.1 to -0.8)	0.000
YLDs	Singapore	2003-2007	2.38 (2.04 to 2.97)	0.008
YLDs	Singapore	2007-2010	0.33 (-1.05 to 0.89)	0.194
YLDs	Singapore	2010-2016	-3.69 (-4.06 to -3.46)	<0.001
YLDs	Singapore	2016-2021	-0.89 (-1.24 to -0.49)	0.002
YLDs	Slovakia	1990-1998	0.39 (0.27 to 0.7)	0.003
YLDs	Slovakia	1998-2002	0.01 (-1.22 to 0.29)	0.963
YLDs	Slovakia	2002-2010	-1.01 (-1.19 to -0.85)	<0.001

YLDs	Slovakia	2010-2014	-4.17 (-4.44 to -3.96)	<0.001
YLDs	Slovakia	2014-2018	-2.27 (-2.64 to -1.87)	<0.001
YLDs	Slovakia	2018-2021	-0.22 (-0.59 to 0.42)	0.190
YLDs	Slovenia	1990-1996	-0.4 (-0.59 to -0.23)	0.009
YLDs	Slovenia	1996-2004	1.56 (1.29 to 1.74)	0.027
YLDs	Slovenia	2004-2007	-0.32 (-0.76 to 1.51)	0.347
YLDs	Slovenia	2007-2010	-1.4 (-3.72 to -1)	<0.001
YLDs	Slovenia	2010-2018	-4.06 (-4.18 to -3.93)	<0.001
YLDs	Slovenia	2018-2021	0.34 (-0.01 to 0.67)	0.060
YLDs	Solomon Islands	1990-2003	-1.23 (-1.51 to -0.53)	0.029
YLDs	Solomon Islands	2003-2006	-3.87 (-5.07 to -1.43)	0.048
YLDs	Solomon Islands	2006-2009	7.24 (3.18 to 8.66)	0.033
YLDs	Solomon Islands	2009-2021	0.04 (-0.39 to 0.42)	0.895

YLDs	Somalia	1990-2006	-5.92 (-6.57 to -5.22)	<0.001
YLDs	Somalia	2006-2011	7.8 (5.52 to 12.88)	0.002
YLDs	Somalia	2011-2021	1.78 (0.39 to 2.68)	0.023
YLDs	South Africa	1990-1995	-1.54 (-1.82 to -1.15)	0.000
YLDs	South Africa	1995-2011	-2.36 (-2.4 to -2.32)	<0.001
YLDs	South Africa	2011-2014	-4.8 (-5.01 to -4.5)	<0.001
YLDs	South Africa	2014-2018	-2.28 (-2.51 to -2.03)	<0.001
YLDs	South Africa	2018-2021	0.37 (0.01 to 0.71)	0.048
YLDs	South Sudan	1990-1996	-6.41 (-8.35 to -4.43)	<0.001
YLDs	South Sudan	1996-1999	34.88 (24.5 to 39.85)	<0.001
YLDs	South Sudan	1999-2007	-8.21 (-10.68 to -6.6)	<0.001
YLDs	South Sudan	2007-2021	1.84 (1.05 to 2.74)	<0.001
YLDs	Spain	1990-1994	0.18 (-0.29 to 0.86)	0.373

YLDs	Spain	1994-2001	-1.47 (-1.97 to -1.23)	<0.001
YLDs	Spain	2001-2009	0.55 (0.31 to 0.89)	<0.001
YLDs	Spain	2009-2018	-1.47 (-1.69 to -1.29)	<0.001
YLDs	Spain	2018-2021	1.89 (1.04 to 3.35)	<0.001
YLDs	Sri Lanka	1990-1992	12.6 (3.28 to 18.41)	0.001
YLDs	Sri Lanka	1992-2000	-0.39 (-3.92 to 1.05)	0.472
YLDs	Sri Lanka	2000-2003	-11.66 (-13.68 to -4.23)	0.048
YLDs	Sri Lanka	2003-2010	11.66 (9.97 to 13.24)	0.024
YLDs	Sri Lanka	2010-2015	-12.54 (-15.39 to -10.56)	0.002
YLDs	Sri Lanka	2015-2021	-6.25 (-7.73 to -3.86)	0.004
YLDs	Sudan	1990-1992	1.62 (-2.51 to 4.3)	0.294
YLDs	Sudan	1992-1995	-4.8 (-6.12 to 1.11)	0.112
YLDs	Sudan	1995-2002	0.88 (-0.71 to 3.69)	0.144

YLDs	Sudan	2002-2005	3.99 (-4.52 to 5.11)	0.316
YLDs	Sudan	2005-2021	-3.77 (-4.79 to -3)	<0.001
YLDs	Suriname	1990-1992	-6.88 (-8.29 to -4.96)	<0.001
YLDs	Suriname	1992-1998	-3.18 (-3.57 to -2.13)	<0.001
YLDs	Suriname	1998-2005	-4.88 (-5.11 to -4.68)	0.001
YLDs	Suriname	2005-2010	-0.19 (-0.43 to 0.09)	0.128
YLDs	Suriname	2010-2016	-1.71 (-2.09 to -1.5)	<0.001
YLDs	Suriname	2016-2021	-0.37 (-0.64 to 0)	0.052
YLDs	Sweden	1990-1994	-1.57 (-2.26 to -1.15)	<0.001
YLDs	Sweden	1994-2008	0.68 (0.61 to 0.78)	0.003
YLDs	Sweden	2008-2011	-1.91 (-2.51 to -0.69)	0.029
YLDs	Sweden	2011-2015	-4.87 (-5.46 to -4.46)	<0.001
YLDs	Sweden	2015-2018	-1.8 (-3.43 to -1.18)	<0.001
YLDs	Sweden	2018-2021	0.53 (-0.07 to 1.47)	0.070

YLDs	Switzerland	1990-1994	0.77 (0.03 to 1.2)	0.043
YLDs	Switzerland	1994-2000	2.7 (2.47 to 3.04)	<0.001
YLDs	Switzerland	2000-2010	-0.67 (-0.78 to -0.56)	0.014
YLDs	Switzerland	2010-2015	-4.1 (-4.53 to -3.76)	0.001
YLDs	Switzerland	2015-2019	-2.8 (-3.48 to -2.29)	<0.001
YLDs	Switzerland	2019-2021	0.61 (-0.52 to 1.29)	0.154
YLDs	Syrian Arab Republic	1990-1997	-3.94 (-11.73 to 7.17)	0.066
YLDs	Syrian Arab Republic	1997-2010	-0.52 (-4.45 to 5.42)	0.603
YLDs	Syrian Arab Republic	2010-2014	66.28 (-3.85 to 76.09)	0.160
YLDs	Syrian Arab Republic	2014-2017	22.77 (5.05 to 69.2)	0.042
YLDs	Syrian Arab Republic	2017-2021	-6.02 (-23.48 to 6.34)	0.165
YLDs	Taiwan (Province of China)	1990-1997	-1.31 (-2.21 to -0.8)	<0.001
YLDs	Taiwan (Province of China)	1997-2000	3.69 (1.59 to 4.62)	<0.001

YLDs	Taiwan (Province of China)	2000-2013	-1.55 (-1.82 to -1.3)	<0.001
YLDs	Taiwan (Province of China)	2013-2021	-4.04 (-4.67 to -3.58)	<0.001
YLDs	Tajikistan	1990-1994	41.57 (28.35 to 50.22)	<0.001
YLDs	Tajikistan	1994-2015	-7.06 (-8.29 to -6.39)	0.002
YLDs	Tajikistan	2015-2021	-1 (-4.76 to 9.72)	0.630
YLDs	Thailand	1990-2003	-1.57 (-1.69 to -1.45)	<0.001
YLDs	Thailand	2003-2006	4.73 (3.63 to 5.25)	0.002
YLDs	Thailand	2006-2012	-0.81 (-1.2 to -0.28)	0.020
YLDs	Thailand	2012-2021	-2.19 (-2.54 to -1.97)	<0.001
YLDs	Timor-Leste	1990-1997	-4.04 (-8.57 to -1.48)	0.001
YLDs	Timor-Leste	1997-2000	25.21 (11.22 to 30.85)	<0.001
YLDs	Timor-Leste	2000-2016	-14.49 (-15.36 to -13.83)	<0.001
YLDs	Timor-Leste	2016-2021	-5.97 (-8.68 to -1.75)	0.026

YLDs	Togo	1990-1995	0.51<br>(-0.06 to 1.48)	0.079
YLDs	Togo	1995-2003	-1.13<br>(-2.02 to -0.85)	0.002
YLDs	Togo	2003-2006	3.8<br>(2.1 to 4.56)	0.002
YLDs	Togo	2006-2015	-1.9<br>(-2.58 to -1.66)	0.002
YLDs	Togo	2015-2021	-0.4<br>(-0.95 to 1.04)	0.264
YLDs	Tokelau	1990-1994	-1.08<br>(-2.14 to -0.44)	0.002
YLDs	Tokelau	1994-2000	1.75<br>(1.37 to 2.47)	<0.001
YLDs	Tokelau	2000-2004	-1.7<br>(-2.58 to -0.99)	<0.001
YLDs	Tokelau	2004-2010	1.31<br>(0.98 to 1.84)	<0.001
YLDs	Tokelau	2010-2014	-4.74<br>(-5.32 to -4.24)	<0.001
YLDs	Tokelau	2014-2021	-0.4<br>(-0.68 to -0.12)	0.024
YLDs	Tonga	1990-2007	-1.11<br>(-1.3 to -0.95)	<0.001
YLDs	Tonga	2007-2010	7.31<br>(4.31 to 8.26)	<0.001

YLDs	Tonga	2010-2021	-2.19 (-2.54 to -1.9)	<0.001
YLDs	Trinidad and Tobago	1990-1999	1.35 (0.92 to 2.48)	0.002
YLDs	Trinidad and Tobago	1999-2007	-0.01 (-1.31 to 0.56)	0.842
YLDs	Trinidad and Tobago	2007-2017	-2.17 (-2.89 to -1.81)	0.012
YLDs	Trinidad and Tobago	2017-2021	7.14 (5.78 to 9.42)	<0.001
YLDs	Tunisia	1990-1997	-1.66 (-1.97 to -1.51)	<0.001
YLDs	Tunisia	1997-2004	-1.09 (-1.25 to -0.7)	<0.001
YLDs	Tunisia	2004-2011	-2.11 (-2.26 to -1.92)	<0.001
YLDs	Tunisia	2011-2014	-3.48 (-3.74 to -2.95)	<0.001
YLDs	Tunisia	2014-2019	-1.79 (-2.2 to -1.46)	<0.001
YLDs	Tunisia	2019-2021	0.27 (-0.59 to 0.76)	0.446
YLDs	Turkey	1990-1995	1.91 (1.24 to 2.99)	0.002
YLDs	Turkey	1995-2000	-0.47 (-1.76 to 0.43)	0.248

YLDs	Turkey	2000-2009	-2.53 (-3.7 to -2.18)	0.000
YLDs	Turkey	2009-2021	-1.75 (-1.99 to -1.11)	0.006
YLDs	Turkmenistan	1990-1993	3.42 (2.84 to 3.98)	<0.001
YLDs	Turkmenistan	1993-2009	-1.44 (-1.52 to -1.37)	<0.001
YLDs	Turkmenistan	2009-2016	-3.68 (-4.04 to -3.38)	<0.001
YLDs	Turkmenistan	2016-2021	-1.08 (-1.52 to -0.45)	0.003
YLDs	Tuvalu	1990-1995	1.01 (0.6 to 1.6)	<0.001
YLDs	Tuvalu	1995-2006	-0.37 (-0.57 to -0.22)	<0.001
YLDs	Tuvalu	2006-2015	-2.41 (-2.72 to -2.19)	<0.001
YLDs	Tuvalu	2015-2021	-0.5 (-0.89 to 0.03)	0.067
YLDs	Uganda	1990-1995	-10.6 (-12.47 to -9.8)	<0.001
YLDs	Uganda	1995-2001	-7.92 (-8.31 to -7.13)	<0.001
YLDs	Uganda	2001-2005	1.41 (0.85 to 1.95)	0.005
YLDs	Uganda	2005-2008	-4.51 (-4.99 to -3.27)	0.004

YLDs	Uganda	2008-2017	-2.6 (-2.83 to -2.13)	<0.001
YLDs	Uganda	2017-2021	0.16 (-0.52 to 1.45)	0.466
YLDs	Ukraine	1990-2000	0.65 (0.45 to 0.87)	0.004
YLDs	Ukraine	2000-2006	-2.83 (-3.28 to -1.91)	0.016
YLDs	Ukraine	2006-2013	-4.26 (-4.83 to -3.94)	0.001
YLDs	Ukraine	2013-2016	1.78 (0.63 to 2.52)	0.021
YLDs	Ukraine	2016-2019	-1.97 (-2.75 to -1.1)	0.012
YLDs	Ukraine	2019-2021	2.43 (0.95 to 3.83)	0.012
YLDs	United Arab Emirates	1990-2000	0.8 (0.64 to 0.98)	0.001
YLDs	United Arab Emirates	2000-2005	-2.42 (-3.11 to -1.88)	0.010
YLDs	United Arab Emirates	2005-2008	1.61 (0.58 to 2.24)	0.035
YLDs	United Arab Emirates	2008-2013	-5.56 (-6.32 to -5.14)	0.011
YLDs	United Arab Emirates	2013-2019	-1.98 (-2.94 to -1.52)	<0.001

YLDs	United Arab Emirates	2019-2021	1.25 (-0.57 to 2.19)	0.128
YLDs	United Kingdom	1990-1995	-1.38 (-1.83 to -1.1)	<0.001
YLDs	United Kingdom	1995-2008	0.51 (0.43 to 0.6)	0.010
YLDs	United Kingdom	2008-2011	-1.17 (-1.84 to -0.45)	0.029
YLDs	United Kingdom	2011-2015	-4.39 (-4.92 to -4.04)	<0.001
YLDs	United Kingdom	2015-2018	-2.5 (-3.61 to -1.39)	<0.001
YLDs	United Kingdom	2018-2021	0.33 (-0.19 to 1.1)	0.115
YLDs	United Republic of Tanzania	1990-1996	-0.77 (-1.4 to -0.53)	0.006
YLDs	United Republic of Tanzania	1996-2005	-0.05 (-0.37 to 0.13)	0.452
YLDs	United Republic of Tanzania	2005-2011	0.46 (0.2 to 0.97)	0.019
YLDs	United Republic of Tanzania	2011-2016	-1.01 (-1.6 to -0.68)	0.010
YLDs	United Republic of Tanzania	2016-2021	0.22 (-0.09 to 0.9)	0.128
YLDs	United States Virgin Islands	1990-2010	-0.21 (-0.27 to -0.15)	<0.001

YLDs	United States Virgin Islands	2010-2015	-2.93 (-3.58 to -2.36)	<0.001
YLDs	United States Virgin Islands	2015-2021	0.03 (-0.33 to 0.56)	0.861
YLDs	United States of America	1990-1996	-0.52 (-0.76 to -0.26)	0.000
YLDs	United States of America	1996-1999	-9.9 (-10.38 to -9.47)	<0.001
YLDs	United States of America	1999-2002	-3.69 (-4.07 to -3.02)	<0.001
YLDs	United States of America	2002-2015	-1.39 (-1.48 to -1.28)	<0.001
YLDs	United States of America	2015-2019	1.68 (1.33 to 2.47)	<0.001
YLDs	United States of America	2019-2021	-1.65 (-2.67 to -0.42)	<0.001
YLDs	Uruguay	1990-1994	0.11 (-0.16 to 0.57)	0.382
YLDs	Uruguay	1994-1999	-0.71 (-1.15 to -0.47)	0.009
YLDs	Uruguay	1999-2011	-0.07 (-0.14 to 0.03)	0.105
YLDs	Uruguay	2011-2018	-2.27 (-2.41 to -2.15)	<0.001
YLDs	Uruguay	2018-2021	0.17 (-0.27 to 0.72)	0.278

YLDs	Uzbekistan	1990-1994	-0.27<br>(-1.33 to 0.29)	0.302
YLDs	Uzbekistan	1994-2003	0.98<br>(0.79 to 1.39)	0.001
YLDs	Uzbekistan	2003-2010	-1.5<br>(-1.8 to -1.1)	0.034
YLDs	Uzbekistan	2010-2015	-5.04<br>(-5.62 to -1.72)	<0.001
YLDs	Uzbekistan	2015-2019	-2.74<br>(-4.72 to -2.1)	<0.001
YLDs	Uzbekistan	2019-2021	0.02<br>(-1.54 to 0.91)	0.909
YLDs	Venezuela (Bolivarian Republic of)	1990-1998	-0.15<br>(-0.84 to 0.35)	0.458
YLDs	Venezuela (Bolivarian Republic of)	1998-2001	9.77<br>(6.4 to 11.1)	0.006
YLDs	Venezuela (Bolivarian Republic of)	2001-2011	-2.1<br>(-2.57 to -1.13)	0.014
YLDs	Venezuela (Bolivarian Republic of)	2011-2021	-3.59<br>(-4.72 to -3.14)	<0.001
YLDs	Viet Nam	1990-1995	-0.27<br>(-1.09 to 0.22)	0.224
YLDs	Viet Nam	1995-2000	2.3<br>(1.77 to 3.3)	<0.001
YLDs	Viet Nam	2000-2009	0.04<br>(-0.21 to 0.24)	0.673

YLDs	Viet Nam	2009-2018	-3.22 (-3.49 to -3.03)	<0.001
YLDs	Viet Nam	2018-2021	-0.42 (-1.42 to 1.11)	0.266
YLDs	Yemen	1990-2005	-3.11 (-3.42 to -2.81)	0.006
YLDs	Yemen	2005-2009	9.16 (7.07 to 12.69)	0.026
YLDs	Yemen	2009-2015	2.86 (1.31 to 3.94)	0.018
YLDs	Yemen	2015-2019	21.33 (18.61 to 25.21)	0.002
YLDs	Yemen	2019-2021	5.73 (-0.95 to 12.85)	0.061
YLDs	Zambia	1990-1993	-1.35 (-2.03 to -0.88)	<0.001
YLDs	Zambia	1993-2007	-0.44 (-0.5 to -0.37)	0.002
YLDs	Zambia	2007-2010	0.94 (0.44 to 1.19)	0.015
YLDs	Zambia	2010-2014	-2.76 (-3.2 to -2.44)	0.011
YLDs	Zambia	2014-2018	-1.28 (-1.82 to -0.75)	0.001
YLDs	Zambia	2018-2021	0.68 (0.18 to 1.47)	0.008

YLDs	Zimbabwe	1990-2006	-0.27 (-0.5 to -0.18)	0.006
YLDs	Zimbabwe	2006-2009	1.28 (0.1 to 1.75)	0.024
YLDs	Zimbabwe	2009-2017	-1.49 (-2.11 to -1.23)	0.001
YLDs	Zimbabwe	2017-2021	1.36 (0.53 to 2.81)	0.003

YLDs years lived with disability, *APC* annual percentage change, *CI* confidence interval.

Supplementary Table 20 Predicted case number and ASR of incidence, prevalence, and YLDs of spinal cord injury among children and adolescents globally to 2045

Measure	Sex	Year	Case number	ASR
Incidence	Male	2022	80702	5.79
Incidence	Male	2023	80124	5.72
Incidence	Male	2024	79517	5.65
Incidence	Male	2025	78674	5.58
Incidence	Male	2026	77776	5.5
Incidence	Male	2027	76821	5.42
Incidence	Male	2028	75803	5.35
Incidence	Male	2029	74728	5.27
Incidence	Male	2030	73963	5.22
Incidence	Male	2031	73149	5.17
Incidence	Male	2032	72315	5.12
Incidence	Male	2033	71362	5.07
Incidence	Male	2034	70360	5.01
Incidence	Male	2035	70037	5.01
Incidence	Male	2036	69693	5.01
Incidence	Male	2037	69306	5
Incidence	Male	2038	69019	5
Incidence	Male	2039	68710	4.99
Incidence	Male	2040	68378	4.99
Incidence	Male	2041	68030	4.98
Incidence	Male	2042	67671	4.98
Incidence	Male	2043	67301	4.97
Incidence	Male	2044	66927	4.97
Incidence	Male	2045	66555	4.96
Incidence	Female	2022	40705	3.17
Incidence	Female	2023	40194	3.12
Incidence	Female	2024	39669	3.08
Incidence	Female	2025	39180	3.03
Incidence	Female	2026	38663	2.99
Incidence	Female	2027	38117	2.95
Incidence	Female	2028	37539	2.91
Incidence	Female	2029	36935	2.87
Incidence	Female	2030	36592	2.85
Incidence	Female	2031	36228	2.83
Incidence	Female	2032	35851	2.81
Incidence	Female	2033	35452	2.78
Incidence	Female	2034	35035	2.76
Incidence	Female	2035	34911	2.76
Incidence	Female	2036	34781	2.76

Incidence	Female	2037	34642	2.76
Incidence	Female	2038	34526	2.77
Incidence	Female	2039	34403	2.77
Incidence	Female	2040	34272	2.77
Incidence	Female	2041	34138	2.77
Incidence	Female	2042	33999	2.77
Incidence	Female	2043	33856	2.77
Incidence	Female	2044	33711	2.77
Incidence	Female	2045	33564	2.77
Prevalence	Male	2022	649627	46.04
Prevalence	Male	2023	646917	45.61
Prevalence	Male	2024	643959	45.18
Prevalence	Male	2025	640825	44.77
Prevalence	Male	2026	637264	44.37
Prevalence	Male	2027	633317	43.96
Prevalence	Male	2028	628557	43.55
Prevalence	Male	2029	623259	43.14
Prevalence	Male	2030	620183	42.92
Prevalence	Male	2031	616657	42.7
Prevalence	Male	2032	612905	42.48
Prevalence	Male	2033	608288	42.26
Prevalence	Male	2034	603174	42.04
Prevalence	Male	2035	600735	42.02
Prevalence	Male	2036	598050	42
Prevalence	Male	2037	594928	41.98
Prevalence	Male	2038	592684	41.95
Prevalence	Male	2039	590227	41.93
Prevalence	Male	2040	587563	41.91
Prevalence	Male	2041	584766	41.89
Prevalence	Male	2042	581870	41.87
Prevalence	Male	2043	578896	41.85
Prevalence	Male	2044	575907	41.82
Prevalence	Male	2045	572968	41.8
Prevalence	Female	2022	434628	33.01
Prevalence	Female	2023	429320	32.46
Prevalence	Female	2024	423953	31.9
Prevalence	Female	2025	418833	31.4
Prevalence	Female	2026	413417	30.89
Prevalence	Female	2027	407681	30.39
Prevalence	Female	2028	401417	29.88
Prevalence	Female	2029	394818	29.38
Prevalence	Female	2030	391879	29.16

Prevalence	Female	2031	388663	28.94
Prevalence	Female	2032	385272	28.72
Prevalence	Female	2033	381456	28.5
Prevalence	Female	2034	377352	28.28
Prevalence	Female	2035	375505	28.24
Prevalence	Female	2036	373561	28.19
Prevalence	Female	2037	371439	28.15
Prevalence	Female	2038	369737	28.1
Prevalence	Female	2039	367901	28.06
Prevalence	Female	2040	365946	28.01
Prevalence	Female	2041	363914	27.97
Prevalence	Female	2042	361822	27.92
Prevalence	Female	2043	359681	27.88
Prevalence	Female	2044	357526	27.83
Prevalence	Female	2045	355391	27.79
YLDs	Male	2022	218119	15.46
YLDs	Male	2023	216647	15.28
YLDs	Male	2024	215085	15.1
YLDs	Male	2025	213623	14.93
YLDs	Male	2026	212014	14.77
YLDs	Male	2027	210271	14.61
YLDs	Male	2028	208260	14.44
YLDs	Male	2029	206068	14.28
YLDs	Male	2030	204782	14.19
YLDs	Male	2031	203347	14.1
YLDs	Male	2032	201836	14.01
YLDs	Male	2033	200044	13.92
YLDs	Male	2034	198090	13.83
YLDs	Male	2035	197253	13.82
YLDs	Male	2036	196336	13.81
YLDs	Male	2037	195278	13.8
YLDs	Male	2038	194504	13.79
YLDs	Male	2039	193662	13.78
YLDs	Male	2040	192754	13.77
YLDs	Male	2041	191803	13.76
YLDs	Male	2042	190820	13.75
YLDs	Male	2043	189812	13.74
YLDs	Male	2044	188800	13.73
YLDs	Male	2045	187804	13.72
YLDs	Female	2022	142703	10.85
YLDs	Female	2023	140499	10.63
YLDs	Female	2024	138270	10.42

YLDs	Female	2025	136268	10.23
YLDs	Female	2026	134166	10.04
YLDs	Female	2027	131957	9.85
YLDs	Female	2028	129578	9.66
YLDs	Female	2029	127090	9.47
YLDs	Female	2030	125941	9.39
YLDs	Female	2031	124703	9.3
YLDs	Female	2032	123409	9.22
YLDs	Female	2033	121984	9.14
YLDs	Female	2034	120469	9.05
YLDs	Female	2035	119837	9.03
YLDs	Female	2036	119174	9.02
YLDs	Female	2037	118457	9
YLDs	Female	2038	117870	8.98
YLDs	Female	2039	117242	8.96
YLDs	Female	2040	116578	8.95
YLDs	Female	2041	115891	8.93
YLDs	Female	2042	115186	8.91
YLDs	Female	2043	114466	8.89
YLDs	Female	2044	113742	8.88
YLDs	Female	2045	113024	8.86

YLDs years lived with disability, *ASR* age-standardized rate.

Supplementary Table 21 Predicted case number and rate of incidence, prevalence, and YLDs of spinal cord injury among children and adolescents globally to 2045, stratified by age

Measure	Sex	Age (y)	Year	Case number	Rate
Incidence	Male	0 to 4	2022	12372.84	3.57
Incidence	Male	0 to 4	2023	12299.16	3.56
Incidence	Male	0 to 4	2024	12217.04	3.55
Incidence	Male	0 to 4	2025	12137.8	3.54
Incidence	Male	0 to 4	2026	12051.28	3.53
Incidence	Male	0 to 4	2027	11959.21	3.52
Incidence	Male	0 to 4	2028	11863.38	3.5
Incidence	Male	0 to 4	2029	11766.68	3.49
Incidence	Male	0 to 4	2030	11683.24	3.49
Incidence	Male	0 to 4	2031	11602.22	3.48
Incidence	Male	0 to 4	2032	11523.36	3.47
Incidence	Male	0 to 4	2033	11445.9	3.47
Incidence	Male	0 to 4	2034	11370.33	3.46
Incidence	Male	0 to 4	2035	11308.64	3.46
Incidence	Male	0 to 4	2036	11249.37	3.45
Incidence	Male	0 to 4	2037	11192.31	3.45
Incidence	Male	0 to 4	2038	11136.37	3.45
Incidence	Male	0 to 4	2039	11080.36	3.44
Incidence	Male	0 to 4	2040	11024.02	3.44
Incidence	Male	0 to 4	2041	10967.06	3.44
Incidence	Male	0 to 4	2042	10909.13	3.43
Incidence	Male	0 to 4	2043	10849.55	3.43
Incidence	Male	0 to 4	2044	10787.59	3.43
Incidence	Male	0 to 4	2045	10722.37	3.42
Incidence	Male	5 to 9	2022	14874.61	4.26
Incidence	Male	5 to 9	2023	14322.82	4.11
Incidence	Male	5 to 9	2024	13754.97	3.95
Incidence	Male	5 to 9	2025	13672.24	3.94
Incidence	Male	5 to 9	2026	13582.38	3.93
Incidence	Male	5 to 9	2027	13475.74	3.92
Incidence	Male	5 to 9	2028	13411.58	3.91
Incidence	Male	5 to 9	2029	13338.05	3.9
Incidence	Male	5 to 9	2030	13266.95	3.89
Incidence	Male	5 to 9	2031	13187.72	3.88
Incidence	Male	5 to 9	2032	13102.28	3.87
Incidence	Male	5 to 9	2033	13012.52	3.87
Incidence	Male	5 to 9	2034	12921.62	3.86
Incidence	Male	5 to 9	2035	12844.69	3.86
Incidence	Male	5 to 9	2036	12770.3	3.85

Incidence	Male	5 to 9	2037	12698.03	3.85
Incidence	Male	5 to 9	2038	12627.13	3.84
Incidence	Male	5 to 9	2039	12558.12	3.84
Incidence	Male	5 to 9	2040	12491.85	3.84
Incidence	Male	5 to 9	2041	12428.21	3.83
Incidence	Male	5 to 9	2042	12366.97	3.83
Incidence	Male	5 to 9	2043	12306.91	3.83
Incidence	Male	5 to 9	2044	12246.71	3.82
Incidence	Male	5 to 9	2045	12186.05	3.82
Incidence	Male	10 to 14	2022	15251.68	4.48
Incidence	Male	10 to 14	2023	15165.58	4.43
Incidence	Male	10 to 14	2024	15067.7	4.37
Incidence	Male	10 to 14	2025	14774.81	4.27
Incidence	Male	10 to 14	2026	14466.12	4.17
Incidence	Male	10 to 14	2027	14157.72	4.07
Incidence	Male	10 to 14	2028	13784.52	3.96
Incidence	Male	10 to 14	2029	13395.27	3.86
Incidence	Male	10 to 14	2030	13327.9	3.85
Incidence	Male	10 to 14	2031	13253.47	3.84
Incidence	Male	10 to 14	2032	13162.54	3.84
Incidence	Male	10 to 14	2033	13113.01	3.83
Incidence	Male	10 to 14	2034	13054.23	3.82
Incidence	Male	10 to 14	2035	12997.59	3.82
Incidence	Male	10 to 14	2036	12932.88	3.81
Incidence	Male	10 to 14	2037	12861.93	3.81
Incidence	Male	10 to 14	2038	12786.62	3.81
Incidence	Male	10 to 14	2039	12710.03	3.8
Incidence	Male	10 to 14	2040	12634.73	3.8
Incidence	Male	10 to 14	2041	12561.92	3.8
Incidence	Male	10 to 14	2042	12491.18	3.79
Incidence	Male	10 to 14	2043	12421.77	3.79
Incidence	Male	10 to 14	2044	12354.22	3.78
Incidence	Male	10 to 14	2045	12289.31	3.78
Incidence	Male	15 to 19	2022	38202.4	11.67
Incidence	Male	15 to 19	2023	38336.13	11.62
Incidence	Male	15 to 19	2024	38477.61	11.57
Incidence	Male	15 to 19	2025	38088.92	11.37
Incidence	Male	15 to 19	2026	37676.11	11.16
Incidence	Male	15 to 19	2027	37228.1	10.96
Incidence	Male	15 to 19	2028	36743.94	10.76
Incidence	Male	15 to 19	2029	36228.27	10.55
Incidence	Male	15 to 19	2030	35685.24	10.35

Incidence	Male	15 to 19	2031	35105.16	10.15
Incidence	Male	15 to 19	2032	34526.77	9.94
Incidence	Male	15 to 19	2033	33790.46	9.74
Incidence	Male	15 to 19	2034	33014.15	9.54
Incidence	Male	15 to 19	2035	32886.4	9.53
Incidence	Male	15 to 19	2036	32740.78	9.52
Incidence	Male	15 to 19	2037	32553.9	9.52
Incidence	Male	15 to 19	2038	32469.28	9.51
Incidence	Male	15 to 19	2039	32361.6	9.5
Incidence	Male	15 to 19	2040	32227.42	9.49
Incidence	Male	15 to 19	2041	32073.08	9.48
Incidence	Male	15 to 19	2042	31903.23	9.48
Incidence	Male	15 to 19	2043	31722.48	9.47
Incidence	Male	15 to 19	2044	31538.54	9.46
Incidence	Male	15 to 19	2045	31357.64	9.45
Incidence	Female	0 to 4	2022	9799.83	3.02
Incidence	Female	0 to 4	2023	9789.78	3.03
Incidence	Female	0 to 4	2024	9772.31	3.03
Incidence	Female	0 to 4	2025	9745.33	3.03
Incidence	Female	0 to 4	2026	9711.97	3.03
Incidence	Female	0 to 4	2027	9673.65	3.03
Incidence	Female	0 to 4	2028	9631.77	3.03
Incidence	Female	0 to 4	2029	9588.56	3.03
Incidence	Female	0 to 4	2030	9544.75	3.03
Incidence	Female	0 to 4	2031	9502.39	3.03
Incidence	Female	0 to 4	2032	9461.3	3.03
Incidence	Female	0 to 4	2033	9420.77	3.03
Incidence	Female	0 to 4	2034	9381.25	3.04
Incidence	Female	0 to 4	2035	9342.21	3.04
Incidence	Female	0 to 4	2036	9304.62	3.04
Incidence	Female	0 to 4	2037	9268.38	3.04
Incidence	Female	0 to 4	2038	9232.68	3.04
Incidence	Female	0 to 4	2039	9196.61	3.04
Incidence	Female	0 to 4	2040	9159.98	3.04
Incidence	Female	0 to 4	2041	9122.65	3.04
Incidence	Female	0 to 4	2042	9084.4	3.04
Incidence	Female	0 to 4	2043	9044.73	3.04
Incidence	Female	0 to 4	2044	9003	3.04
Incidence	Female	0 to 4	2045	8958.55	3.04
Incidence	Female	5 to 9	2022	8793.81	2.69
Incidence	Female	5 to 9	2023	8450.45	2.59
Incidence	Female	5 to 9	2024	8098.21	2.49

Incidence	Female	5 to 9	2025	8076.93	2.49
Incidence	Female	5 to 9	2026	8053.78	2.49
Incidence	Female	5 to 9	2027	8024.22	2.49
Incidence	Female	5 to 9	2028	8016.46	2.49
Incidence	Female	5 to 9	2029	8002.58	2.49
Incidence	Female	5 to 9	2030	7980.83	2.49
Incidence	Female	5 to 9	2031	7953.85	2.5
Incidence	Female	5 to 9	2032	7922.79	2.5
Incidence	Female	5 to 9	2033	7888.79	2.5
Incidence	Female	5 to 9	2034	7853.69	2.5
Incidence	Female	5 to 9	2035	7818.08	2.5
Incidence	Female	5 to 9	2036	7783.67	2.5
Incidence	Female	5 to 9	2037	7750.18	2.5
Incidence	Female	5 to 9	2038	7717.14	2.5
Incidence	Female	5 to 9	2039	7684.88	2.5
Incidence	Female	5 to 9	2040	7653.89	2.5
Incidence	Female	5 to 9	2041	7624.05	2.5
Incidence	Female	5 to 9	2042	7595.3	2.5
Incidence	Female	5 to 9	2043	7566.96	2.5
Incidence	Female	5 to 9	2044	7538.29	2.5
Incidence	Female	5 to 9	2045	7509.12	2.5
Incidence	Female	10 to 14	2022	10035	3.15
Incidence	Female	10 to 14	2023	9900.12	3.09
Incidence	Female	10 to 14	2024	9758.17	3.03
Incidence	Female	10 to 14	2025	9524.52	2.94
Incidence	Female	10 to 14	2026	9278.34	2.86
Incidence	Female	10 to 14	2027	9028.29	2.77
Incidence	Female	10 to 14	2028	8740.25	2.69
Incidence	Female	10 to 14	2029	8442.75	2.6
Incidence	Female	10 to 14	2030	8419.86	2.6
Incidence	Female	10 to 14	2031	8395	2.6
Incidence	Female	10 to 14	2032	8363.46	2.6
Incidence	Female	10 to 14	2033	8354.65	2.6
Incidence	Female	10 to 14	2034	8339.45	2.6
Incidence	Female	10 to 14	2035	8316.06	2.6
Incidence	Female	10 to 14	2036	8287.2	2.6
Incidence	Female	10 to 14	2037	8254.09	2.6
Incidence	Female	10 to 14	2038	8217.91	2.6
Incidence	Female	10 to 14	2039	8180.59	2.61
Incidence	Female	10 to 14	2040	8143.71	2.61
Incidence	Female	10 to 14	2041	8108.08	2.61
Incidence	Female	10 to 14	2042	8073.39	2.61

Incidence	Female	10 to 14	2043	8039.16	2.61
Incidence	Female	10 to 14	2044	8005.74	2.61
Incidence	Female	10 to 14	2045	7973.64	2.61
Incidence	Female	15 to 19	2022	12076.6	3.93
Incidence	Female	15 to 19	2023	12053.44	3.9
Incidence	Female	15 to 19	2024	12039.89	3.87
Incidence	Female	15 to 19	2025	11832.82	3.77
Incidence	Female	15 to 19	2026	11618.45	3.68
Incidence	Female	15 to 19	2027	11390.56	3.58
Incidence	Female	15 to 19	2028	11150.43	3.49
Incidence	Female	15 to 19	2029	10901.36	3.39
Incidence	Female	15 to 19	2030	10646.24	3.3
Incidence	Female	15 to 19	2031	10377.11	3.2
Incidence	Female	15 to 19	2032	10103.66	3.11
Incidence	Female	15 to 19	2033	9787.62	3.01
Incidence	Female	15 to 19	2034	9460.93	2.92
Incidence	Female	15 to 19	2035	9434.46	2.92
Incidence	Female	15 to 19	2036	9405.76	2.92
Incidence	Female	15 to 19	2037	9369.51	2.92
Incidence	Female	15 to 19	2038	9358.76	2.92
Incidence	Female	15 to 19	2039	9340.84	2.92
Incidence	Female	15 to 19	2040	9314.85	2.92
Incidence	Female	15 to 19	2041	9282.73	2.92
Incidence	Female	15 to 19	2042	9245.83	2.92
Incidence	Female	15 to 19	2043	9205.48	2.92
Incidence	Female	15 to 19	2044	9163.86	2.92
Incidence	Female	15 to 19	2045	9122.71	2.92
Prevalence	Male	0 to 4	2022	34307.72	9.91
Prevalence	Male	0 to 4	2023	34140.63	9.88
Prevalence	Male	0 to 4	2024	33949.9	9.86
Prevalence	Male	0 to 4	2025	33757	9.83
Prevalence	Male	0 to 4	2026	33543.61	9.81
Prevalence	Male	0 to 4	2027	33314.55	9.79
Prevalence	Male	0 to 4	2028	33074.75	9.77
Prevalence	Male	0 to 4	2029	32832.21	9.75
Prevalence	Male	0 to 4	2030	32617.12	9.74
Prevalence	Male	0 to 4	2031	32408.58	9.72
Prevalence	Male	0 to 4	2032	32205.93	9.71
Prevalence	Male	0 to 4	2033	32006.99	9.69
Prevalence	Male	0 to 4	2034	31813.17	9.68
Prevalence	Male	0 to 4	2035	31649.23	9.67
Prevalence	Male	0 to 4	2036	31491.95	9.67

Prevalence	Male	0 to 4	2037	31340.82	9.66
Prevalence	Male	0 to 4	2038	31192.75	9.65
Prevalence	Male	0 to 4	2039	31044.39	9.65
Prevalence	Male	0 to 4	2040	30895	9.64
Prevalence	Male	0 to 4	2041	30743.79	9.63
Prevalence	Male	0 to 4	2042	30589.8	9.62
Prevalence	Male	0 to 4	2043	30431.11	9.62
Prevalence	Male	0 to 4	2044	30265.65	9.61
Prevalence	Male	0 to 4	2045	30090.98	9.6
Prevalence	Male	5 to 9	2022	105069.68	30.1
Prevalence	Male	5 to 9	2023	103560.98	29.7
Prevalence	Male	5 to 9	2024	101927.75	29.3
Prevalence	Male	5 to 9	2025	101432.53	29.25
Prevalence	Male	5 to 9	2026	100883.63	29.2
Prevalence	Male	5 to 9	2027	100209.1	29.15
Prevalence	Male	5 to 9	2028	99849.62	29.1
Prevalence	Male	5 to 9	2029	99419.87	29.05
Prevalence	Male	5 to 9	2030	98967.12	29.01
Prevalence	Male	5 to 9	2031	98453.15	28.98
Prevalence	Male	5 to 9	2032	97892.14	28.95
Prevalence	Male	5 to 9	2033	97298.16	28.91
Prevalence	Male	5 to 9	2034	96694.8	28.88
Prevalence	Male	5 to 9	2035	96156.88	28.86
Prevalence	Male	5 to 9	2036	95637.57	28.85
Prevalence	Male	5 to 9	2037	95133.76	28.83
Prevalence	Male	5 to 9	2038	94639.87	28.81
Prevalence	Male	5 to 9	2039	94159.81	28.8
Prevalence	Male	5 to 9	2040	93699.74	28.78
Prevalence	Male	5 to 9	2041	93259.07	28.76
Prevalence	Male	5 to 9	2042	92836.13	28.75
Prevalence	Male	5 to 9	2043	92421.78	28.73
Prevalence	Male	5 to 9	2044	92006.05	28.71
Prevalence	Male	5 to 9	2045	91586.59	28.7
Prevalence	Male	10 to 14	2022	191243.99	56.12
Prevalence	Male	10 to 14	2023	190092.93	55.47
Prevalence	Male	10 to 14	2024	188793.39	54.81
Prevalence	Male	10 to 14	2025	187491.37	54.2
Prevalence	Male	10 to 14	2026	186007.19	53.6
Prevalence	Male	10 to 14	2027	184543.76	52.99
Prevalence	Male	10 to 14	2028	182242.13	52.38
Prevalence	Male	10 to 14	2029	179719.58	51.77
Prevalence	Male	10 to 14	2030	178978.38	51.72

Prevalence	Male	10 to 14	2031	178141.23	51.67
Prevalence	Male	10 to 14	2032	177081	51.61
Prevalence	Male	10 to 14	2033	176576.63	51.56
Prevalence	Male	10 to 14	2034	175946.99	51.51
Prevalence	Male	10 to 14	2035	175263.71	51.48
Prevalence	Male	10 to 14	2036	174470.91	51.46
Prevalence	Male	10 to 14	2037	173593.43	51.43
Prevalence	Male	10 to 14	2038	172656.15	51.4
Prevalence	Male	10 to 14	2039	171700.93	51.38
Prevalence	Male	10 to 14	2040	170761.88	51.35
Prevalence	Male	10 to 14	2041	169855.63	51.32
Prevalence	Male	10 to 14	2042	168976.73	51.3
Prevalence	Male	10 to 14	2043	168115.09	51.27
Prevalence	Male	10 to 14	2044	167277.83	51.24
Prevalence	Male	10 to 14	2045	166475.76	51.22
Prevalence	Male	15 to 19	2022	319005.64	97.44
Prevalence	Male	15 to 19	2023	319122.22	96.74
Prevalence	Male	15 to 19	2024	319287.84	96.04
Prevalence	Male	15 to 19	2025	318143.66	94.96
Prevalence	Male	15 to 19	2026	316829.6	93.89
Prevalence	Male	15 to 19	2027	315249.49	92.81
Prevalence	Male	15 to 19	2028	313390.65	91.74
Prevalence	Male	15 to 19	2029	311287.7	90.66
Prevalence	Male	15 to 19	2030	309620.18	89.79
Prevalence	Male	15 to 19	2031	307654.53	88.92
Prevalence	Male	15 to 19	2032	305725.44	88.05
Prevalence	Male	15 to 19	2033	302406.3	87.18
Prevalence	Male	15 to 19	2034	298718.67	86.31
Prevalence	Male	15 to 19	2035	297665.52	86.27
Prevalence	Male	15 to 19	2036	296450	86.23
Prevalence	Male	15 to 19	2037	294860.04	86.19
Prevalence	Male	15 to 19	2038	294195.59	86.15
Prevalence	Male	15 to 19	2039	293321.68	86.11
Prevalence	Male	15 to 19	2040	292206.59	86.07
Prevalence	Male	15 to 19	2041	290907.86	86.03
Prevalence	Male	15 to 19	2042	289467.69	85.99
Prevalence	Male	15 to 19	2043	287927.64	85.95
Prevalence	Male	15 to 19	2044	286357.62	85.91
Prevalence	Male	15 to 19	2045	284814.28	85.87
Prevalence	Female	0 to 4	2022	27883.35	8.6
Prevalence	Female	0 to 4	2023	27607.84	8.53
Prevalence	Female	0 to 4	2024	27312.3	8.46

Prevalence	Female	0 to 4	2025	27057.89	8.41
Prevalence	Female	0 to 4	2026	26787.02	8.35
Prevalence	Female	0 to 4	2027	26503.91	8.3
Prevalence	Female	0 to 4	2028	26212.61	8.25
Prevalence	Female	0 to 4	2029	25919.41	8.2
Prevalence	Female	0 to 4	2030	25686.8	8.16
Prevalence	Female	0 to 4	2031	25459.2	8.13
Prevalence	Female	0 to 4	2032	25236.01	8.09
Prevalence	Female	0 to 4	2033	25015.39	8.06
Prevalence	Female	0 to 4	2034	24798.43	8.02
Prevalence	Female	0 to 4	2035	24640.14	8.01
Prevalence	Female	0 to 4	2036	24486.14	7.99
Prevalence	Female	0 to 4	2037	24336.14	7.97
Prevalence	Female	0 to 4	2038	24188	7.96
Prevalence	Female	0 to 4	2039	24039.33	7.94
Prevalence	Female	0 to 4	2040	23890.07	7.92
Prevalence	Female	0 to 4	2041	23739.42	7.91
Prevalence	Female	0 to 4	2042	23586.85	7.89
Prevalence	Female	0 to 4	2043	23431.03	7.87
Prevalence	Female	0 to 4	2044	23270.39	7.86
Prevalence	Female	0 to 4	2045	23103.2	7.84
Prevalence	Female	5 to 9	2022	75571.22	23.15
Prevalence	Female	5 to 9	2023	74078.99	22.72
Prevalence	Female	5 to 9	2024	72503.63	22.29
Prevalence	Female	5 to 9	2025	71915.36	22.17
Prevalence	Female	5 to 9	2026	71312.96	22.06
Prevalence	Female	5 to 9	2027	70656.67	21.94
Prevalence	Female	5 to 9	2028	70194.44	21.83
Prevalence	Female	5 to 9	2029	69679.96	21.72
Prevalence	Female	5 to 9	2030	69233.73	21.64
Prevalence	Female	5 to 9	2031	68743.76	21.57
Prevalence	Female	5 to 9	2032	68220.61	21.49
Prevalence	Female	5 to 9	2033	67674.26	21.41
Prevalence	Female	5 to 9	2034	67120.87	21.34
Prevalence	Female	5 to 9	2035	66692.15	21.3
Prevalence	Female	5 to 9	2036	66274.95	21.27
Prevalence	Female	5 to 9	2037	65866.61	21.23
Prevalence	Female	5 to 9	2038	65463.18	21.19
Prevalence	Female	5 to 9	2039	65067.44	21.15
Prevalence	Female	5 to 9	2040	64684.23	21.12
Prevalence	Female	5 to 9	2041	64311.85	21.08
Prevalence	Female	5 to 9	2042	63949.54	21.04

Prevalence	Female	5 to 9	2043	63591.61	21.01
Prevalence	Female	5 to 9	2044	63231.79	20.97
Prevalence	Female	5 to 9	2045	62868.82	20.93
Prevalence	Female	10 to 14	2022	134779.35	42.28
Prevalence	Female	10 to 14	2023	132103.42	41.2
Prevalence	Female	10 to 14	2024	129323.66	40.13
Prevalence	Female	10 to 14	2025	127911.03	39.51
Prevalence	Female	10 to 14	2026	126343.38	38.9
Prevalence	Female	10 to 14	2027	124734.42	38.29
Prevalence	Female	10 to 14	2028	122604.07	37.68
Prevalence	Female	10 to 14	2029	120334.53	37.06
Prevalence	Female	10 to 14	2030	119606.68	36.95
Prevalence	Female	10 to 14	2031	118853.45	36.83
Prevalence	Female	10 to 14	2032	118008.39	36.72
Prevalence	Female	10 to 14	2033	117486.24	36.6
Prevalence	Female	10 to 14	2034	116875.47	36.49
Prevalence	Female	10 to 14	2035	116351.47	36.43
Prevalence	Female	10 to 14	2036	115752.29	36.37
Prevalence	Female	10 to 14	2037	115095.24	36.32
Prevalence	Female	10 to 14	2038	114397.05	36.26
Prevalence	Female	10 to 14	2039	113684.79	36.2
Prevalence	Female	10 to 14	2040	112981.44	36.15
Prevalence	Female	10 to 14	2041	112297.29	36.09
Prevalence	Female	10 to 14	2042	111627.85	36.03
Prevalence	Female	10 to 14	2043	110966.44	35.98
Prevalence	Female	10 to 14	2044	110317.83	35.92
Prevalence	Female	10 to 14	2045	109688.92	35.86
Prevalence	Female	15 to 19	2022	196394.49	63.99
Prevalence	Female	15 to 19	2023	195529.36	63.29
Prevalence	Female	15 to 19	2024	194813.42	62.59
Prevalence	Female	15 to 19	2025	191949.13	61.2
Prevalence	Female	15 to 19	2026	188974.05	59.81
Prevalence	Female	15 to 19	2027	185786.21	58.41
Prevalence	Female	15 to 19	2028	182405.46	57.02
Prevalence	Female	15 to 19	2029	178884.44	55.63
Prevalence	Female	15 to 19	2030	177351.83	54.91
Prevalence	Female	15 to 19	2031	175607.05	54.18
Prevalence	Female	15 to 19	2032	173807.23	53.46
Prevalence	Female	15 to 19	2033	171280.58	52.74
Prevalence	Female	15 to 19	2034	168557.61	52.02
Prevalence	Female	15 to 19	2035	167821.59	51.95
Prevalence	Female	15 to 19	2036	167047.41	51.87

Prevalence	Female	15 to 19	2037	166141.04	51.79
Prevalence	Female	15 to 19	2038	165688.31	51.72
Prevalence	Female	15 to 19	2039	165109.49	51.64
Prevalence	Female	15 to 19	2040	164390.66	51.57
Prevalence	Female	15 to 19	2041	163565.26	51.49
Prevalence	Female	15 to 19	2042	162657.74	51.42
Prevalence	Female	15 to 19	2043	161691.7	51.34
Prevalence	Female	15 to 19	2044	160705.55	51.27
Prevalence	Female	15 to 19	2045	159730.19	51.19
YLDs	Male	0 to 4	2022	12022.67	3.47
YLDs	Male	0 to 4	2023	11953.04	3.46
YLDs	Male	0 to 4	2024	11875.19	3.45
YLDs	Male	0 to 4	2025	11799.61	3.44
YLDs	Male	0 to 4	2026	11716.92	3.43
YLDs	Male	0 to 4	2027	11628.84	3.42
YLDs	Male	0 to 4	2028	11537.09	3.41
YLDs	Male	0 to 4	2029	11444.46	3.4
YLDs	Male	0 to 4	2030	11364.24	3.39
YLDs	Male	0 to 4	2031	11286.36	3.39
YLDs	Male	0 to 4	2032	11210.57	3.38
YLDs	Male	0 to 4	2033	11136.13	3.37
YLDs	Male	0 to 4	2034	11063.52	3.37
YLDs	Male	0 to 4	2035	11003.95	3.36
YLDs	Male	0 to 4	2036	10946.73	3.36
YLDs	Male	0 to 4	2037	10891.66	3.36
YLDs	Male	0 to 4	2038	10837.67	3.35
YLDs	Male	0 to 4	2039	10783.6	3.35
YLDs	Male	0 to 4	2040	10729.22	3.35
YLDs	Male	0 to 4	2041	10674.22	3.34
YLDs	Male	0 to 4	2042	10618.28	3.34
YLDs	Male	0 to 4	2043	10560.72	3.34
YLDs	Male	0 to 4	2044	10500.85	3.33
YLDs	Male	0 to 4	2045	10437.8	3.33
YLDs	Male	5 to 9	2022	35628.66	10.21
YLDs	Male	5 to 9	2023	35000.97	10.04
YLDs	Male	5 to 9	2024	34331.59	9.87
YLDs	Male	5 to 9	2025	34145.3	9.85
YLDs	Male	5 to 9	2026	33941.08	9.82
YLDs	Male	5 to 9	2027	33694.76	9.8
YLDs	Male	5 to 9	2028	33554.51	9.78
YLDs	Male	5 to 9	2029	33390.72	9.76
YLDs	Male	5 to 9	2030	33225.95	9.74

YLDs	Male	5 to 9	2031	33040.72	9.73
YLDs	Male	5 to 9	2032	32839.81	9.71
YLDs	Male	5 to 9	2033	32627.96	9.7
YLDs	Male	5 to 9	2034	32413.09	9.68
YLDs	Male	5 to 9	2035	32226.58	9.67
YLDs	Male	5 to 9	2036	32046.36	9.67
YLDs	Male	5 to 9	2037	31871.4	9.66
YLDs	Male	5 to 9	2038	31699.82	9.65
YLDs	Male	5 to 9	2039	31532.93	9.64
YLDs	Male	5 to 9	2040	31372.82	9.64
YLDs	Male	5 to 9	2041	31219.26	9.63
YLDs	Male	5 to 9	2042	31071.68	9.62
YLDs	Male	5 to 9	2043	30927.02	9.61
YLDs	Male	5 to 9	2044	30781.95	9.61
YLDs	Male	5 to 9	2045	30635.67	9.6
YLDs	Male	10 to 14	2022	63956.8	18.77
YLDs	Male	10 to 14	2023	63436.04	18.51
YLDs	Male	10 to 14	2024	62864.26	18.25
YLDs	Male	10 to 14	2025	62267.61	18
YLDs	Male	10 to 14	2026	61609.22	17.75
YLDs	Male	10 to 14	2027	60956.56	17.5
YLDs	Male	10 to 14	2028	60026.61	17.25
YLDs	Male	10 to 14	2029	59024.46	17
YLDs	Male	10 to 14	2030	58760.64	16.98
YLDs	Male	10 to 14	2031	58465.44	16.96
YLDs	Male	10 to 14	2032	58097.21	16.93
YLDs	Male	10 to 14	2033	57911.49	16.91
YLDs	Male	10 to 14	2034	57684.76	16.89
YLDs	Male	10 to 14	2035	57450.73	16.88
YLDs	Male	10 to 14	2036	57180.88	16.86
YLDs	Male	10 to 14	2037	56883.35	16.85
YLDs	Male	10 to 14	2038	56566.33	16.84
YLDs	Male	10 to 14	2039	56243.52	16.83
YLDs	Male	10 to 14	2040	55926.16	16.82
YLDs	Male	10 to 14	2041	55619.64	16.81
YLDs	Male	10 to 14	2042	55322.16	16.79
YLDs	Male	10 to 14	2043	55030.43	16.78
YLDs	Male	10 to 14	2044	54746.76	16.77
YLDs	Male	10 to 14	2045	54474.69	16.76
YLDs	Male	15 to 19	2022	106510.63	32.53
YLDs	Male	15 to 19	2023	106256.63	32.21
YLDs	Male	15 to 19	2024	106014.43	31.89

YLDs	Male	15 to 19	2025	105410.2	31.46
YLDs	Male	15 to 19	2026	104746.31	31.04
YLDs	Male	15 to 19	2027	103991.27	30.62
YLDs	Male	15 to 19	2028	103141.39	30.19
YLDs	Male	15 to 19	2029	102208.59	29.77
YLDs	Male	15 to 19	2030	101431.37	29.41
YLDs	Male	15 to 19	2031	100554.71	29.06
YLDs	Male	15 to 19	2032	99688.37	28.71
YLDs	Male	15 to 19	2033	98368.16	28.36
YLDs	Male	15 to 19	2034	96928.85	28
YLDs	Male	15 to 19	2035	96571.81	27.99
YLDs	Male	15 to 19	2036	96162.2	27.97
YLDs	Male	15 to 19	2037	95631.26	27.95
YLDs	Male	15 to 19	2038	95400.59	27.93
YLDs	Male	15 to 19	2039	95102.06	27.92
YLDs	Male	15 to 19	2040	94725.49	27.9
YLDs	Male	15 to 19	2041	94289.5	27.88
YLDs	Male	15 to 19	2042	93807.79	27.87
YLDs	Male	15 to 19	2043	93293.85	27.85
YLDs	Male	15 to 19	2044	92770.35	27.83
YLDs	Male	15 to 19	2045	92255.64	27.81
YLDs	Female	0 to 4	2022	9684.64	2.99
YLDs	Female	0 to 4	2023	9573.55	2.96
YLDs	Female	0 to 4	2024	9455.58	2.93
YLDs	Female	0 to 4	2025	9356.54	2.91
YLDs	Female	0 to 4	2026	9251.89	2.89
YLDs	Female	0 to 4	2027	9143.11	2.86
YLDs	Female	0 to 4	2028	9031.6	2.84
YLDs	Female	0 to 4	2029	8919.54	2.82
YLDs	Female	0 to 4	2030	8832.45	2.81
YLDs	Female	0 to 4	2031	8747.16	2.79
YLDs	Female	0 to 4	2032	8663.44	2.78
YLDs	Female	0 to 4	2033	8580.68	2.76
YLDs	Female	0 to 4	2034	8499.23	2.75
YLDs	Female	0 to 4	2035	8441.56	2.74
YLDs	Female	0 to 4	2036	8385.39	2.74
YLDs	Female	0 to 4	2037	8330.62	2.73
YLDs	Female	0 to 4	2038	8276.51	2.72
YLDs	Female	0 to 4	2039	8222.25	2.72
YLDs	Female	0 to 4	2040	8167.87	2.71
YLDs	Female	0 to 4	2041	8113.05	2.7
YLDs	Female	0 to 4	2042	8057.6	2.69

YLDs	Female	0 to 4	2043	8001.07	2.69
YLDs	Female	0 to 4	2044	7942.92	2.68
YLDs	Female	0 to 4	2045	7882.58	2.67
YLDs	Female	5 to 9	2022	25416.22	7.79
YLDs	Female	5 to 9	2023	24808.67	7.61
YLDs	Female	5 to 9	2024	24173.64	7.43
YLDs	Female	5 to 9	2025	23953.22	7.39
YLDs	Female	5 to 9	2026	23728.24	7.34
YLDs	Female	5 to 9	2027	23485.51	7.29
YLDs	Female	5 to 9	2028	23307.41	7.25
YLDs	Female	5 to 9	2029	23112.05	7.2
YLDs	Female	5 to 9	2030	22948.26	7.17
YLDs	Female	5 to 9	2031	22770.08	7.14
YLDs	Female	5 to 9	2032	22581.03	7.11
YLDs	Female	5 to 9	2033	22384.44	7.08
YLDs	Female	5 to 9	2034	22185.67	7.05
YLDs	Female	5 to 9	2035	22036.29	7.04
YLDs	Female	5 to 9	2036	21890.78	7.02
YLDs	Female	5 to 9	2037	21748.27	7.01
YLDs	Female	5 to 9	2038	21607.45	6.99
YLDs	Female	5 to 9	2039	21469.23	6.98
YLDs	Female	5 to 9	2040	21335.33	6.97
YLDs	Female	5 to 9	2041	21205.06	6.95
YLDs	Female	5 to 9	2042	21078.17	6.94
YLDs	Female	5 to 9	2043	20952.78	6.92
YLDs	Female	5 to 9	2044	20826.83	6.91
YLDs	Female	5 to 9	2045	20699.89	6.89
YLDs	Female	10 to 14	2022	44285.39	13.89
YLDs	Female	10 to 14	2023	43270.3	13.5
YLDs	Female	10 to 14	2024	42219.67	13.1
YLDs	Female	10 to 14	2025	41627.18	12.86
YLDs	Female	10 to 14	2026	40983.22	12.62
YLDs	Female	10 to 14	2027	40325	12.38
YLDs	Female	10 to 14	2028	39497.96	12.14
YLDs	Female	10 to 14	2029	38626.55	11.9
YLDs	Female	10 to 14	2030	38368.25	11.85
YLDs	Female	10 to 14	2031	38101.95	11.81
YLDs	Female	10 to 14	2032	37806.4	11.76
YLDs	Female	10 to 14	2033	37614.42	11.72
YLDs	Female	10 to 14	2034	37394.16	11.67
YLDs	Female	10 to 14	2035	37214.4	11.65
YLDs	Female	10 to 14	2036	37010.67	11.63

YLDs	Female	10 to 14	2037	36788.54	11.61
YLDs	Female	10 to 14	2038	36553.35	11.59
YLDs	Female	10 to 14	2039	36313.78	11.56
YLDs	Female	10 to 14	2040	36077.34	11.54
YLDs	Female	10 to 14	2041	35847.13	11.52
YLDs	Female	10 to 14	2042	35621.72	11.5
YLDs	Female	10 to 14	2043	35398.98	11.48
YLDs	Female	10 to 14	2044	35180.43	11.45
YLDs	Female	10 to 14	2045	34968.25	11.43
YLDs	Female	15 to 19	2022	63316.86	20.63
YLDs	Female	15 to 19	2023	62846.4	20.34
YLDs	Female	15 to 19	2024	62421.19	20.06
YLDs	Female	15 to 19	2025	61330.73	19.55
YLDs	Female	15 to 19	2026	60202.2	19.05
YLDs	Female	15 to 19	2027	59003.34	18.55
YLDs	Female	15 to 19	2028	57740.91	18.05
YLDs	Female	15 to 19	2029	56431.93	17.55
YLDs	Female	15 to 19	2030	55792	17.27
YLDs	Female	15 to 19	2031	55084.1	17
YLDs	Female	15 to 19	2032	54357.9	16.72
YLDs	Female	15 to 19	2033	53404.06	16.44
YLDs	Female	15 to 19	2034	52389.55	16.17
YLDs	Female	15 to 19	2035	52144.45	16.14
YLDs	Female	15 to 19	2036	51887.58	16.11
YLDs	Female	15 to 19	2037	51589.78	16.08
YLDs	Female	15 to 19	2038	51432.92	16.05
YLDs	Female	15 to 19	2039	51236.98	16.03
YLDs	Female	15 to 19	2040	50997.88	16
YLDs	Female	15 to 19	2041	50725.82	15.97
YLDs	Female	15 to 19	2042	50428.41	15.94
YLDs	Female	15 to 19	2043	50113	15.91
YLDs	Female	15 to 19	2044	49791.51	15.88
YLDs	Female	15 to 19	2045	49473.5	15.86

YLDs years lived with disability.

Supplementary Table 22 Causes of incidence, prevalence, and YLDs of spinal cord injury among children and adolescents in China in 2021

Measure	Cause	Number	Rate
Incidence	Self-harm	204.92 (78.68 to 434.54)	0.06 (0.02 to 0.13)
Incidence	Poisonings	62.14 (25.15 to 115.62)	0.02 (0.01 to 0.03)
Incidence	Exposure to forces of nature	22.53 (9.08 to 45.98)	0.01 (0.00 to 0.01)
Incidence	Animal contact	135.65 (52.76 to 272.80)	0.04 (0.02 to 0.08)
Incidence	Drowning	92.69 (43.67 to 165.44)	0.03 (0.01 to 0.05)
Incidence	Interpersonal violence	1578.41 (857.65 to 2660.66)	0.47 (0.26 to 0.80)
Incidence	Road injuries	1324.83 (874.22 to 1968.63)	0.40 (0.26 to 0.59)
Incidence	Exposure to mechanical forces	295.74 (144.14 to 545.56)	0.09 (0.04 to 0.16)
Incidence	Falls	5951.76 (3642.53 to 9643.86)	1.78 (1.09 to 2.88)
Incidence	Foreign body	268.08 (142.30 to 478.03)	0.08 (0.04 to 0.14)
Incidence	Environmental heat and cold exposure	180.45 (87.88 to 324.62)	0.05 (0.03 to 0.10)
Incidence	Adverse effects of medical treatment	0.00 (0.00 to 0.00)	0.00 (0.00 to 0.00)

Incidence	Other transport injuries	362.53 (158.13 to 740.40)	0.11 (0.05 to 0.22)
Incidence	Fire, heat, and hot substances	60.58 (27.74 to 116.53)	0.02 (0.01 to 0.03)
Incidence	Police conflict and executions	13.56 (5.19 to 28.79)	0.00 (0.00 to 0.01)
Incidence	Conflict and terrorism	3.95 (1.68 to 7.70)	0.00 (0.00 to 0.00)
Prevalence	Self-harm	1032.80 (658.81 to 1546.59)	0.31 (0.20 to 0.46)
Prevalence	Poisonings	638.73 (513.89 to 813.48)	0.19 (0.15 to 0.24)
Prevalence	Exposure to forces of nature	4614.94 (1852.15 to 9812.98)	1.38 (0.55 to 2.94)
Prevalence	Animal contact	10.40 (4.04 to 21.67)	0.00 (0.00 to 0.01)
Prevalence	Drowning	1105.52 (806.76 to 1438.59)	0.33 (0.24 to 0.43)
Prevalence	Road injuries	10836.88 (9616.30 to 12559.92)	3.24 (2.88 to 3.76)
Prevalence	Interpersonal violence	29308.58 (25311.45 to 34489.62)	8.77 (7.57 to 10.32)
Prevalence	Exposure to mechanical forces	3518.38 (2864.94 to 4345.08)	1.05 (0.86 to 1.30)
Prevalence	Foreign body	3454.30 (2845.60 to 4112.54)	1.03 (0.85 to 1.23)

Prevalence	Environmental heat and cold exposure	2613.17 (1973.65 to 3478.80)	0.78 (0.59 to 1.04)
Prevalence	Falls	51739.69 (38715.09 to 71263.30)	15.48 (11.58 to 21.32)
Prevalence	Adverse effects of medical treatment	0.00 (0.00 to 0.00)	0.00 (0.00 to 0.00)
Prevalence	Fire, heat, and hot substances	594.37 (380.77 to 874.97)	0.18 (0.11 to 0.26)
Prevalence	Other transport injuries	4496.98 (3260.13 to 6190.01)	1.35 (0.98 to 1.85)
Prevalence	Conflict and terrorism	59.82 (23.68 to 122.92)	0.02 (0.01 to 0.04)
Prevalence	Police conflict and executions	84.32 (34.40 to 165.73)	0.03 (0.01 to 0.05)
YLDs	Self-harm	301.42 (170.41 to 494.84)	0.09 (0.05 to 0.15)
YLDs	Exposure to forces of nature	1356.57 (429.62 to 3148.59)	0.41 (0.13 to 0.94)
YLDs	Poisonings	188.01 (120.14 to 262.80)	0.06 (0.04 to 0.08)
YLDs	Animal contact	7.05 (2.88 to 14.64)	0.00 (0.00 to 0.00)
YLDs	Interpersonal violence	8345.12 (5536.78 to 11341.56)	2.50 (1.66 to 3.39)
YLDs	Road injuries	3200.55 (2198.32 to 4316.72)	0.96 (0.66 to 1.29)

YLDs	Drowning	333.38 (212.55 to 471.91)	0.10 (0.06 to 0.14)
YLDs	Exposure to mechanical forces	1065.04 (714.35 to 1466.49)	0.32 (0.21 to 0.44)
YLDs	Foreign body	927.29 (637.28 to 1271.91)	0.28 (0.19 to 0.38)
YLDs	Falls	15096.27 (9470.77 to 21764.67)	4.52 (2.83 to 6.51)
YLDs	Environmental heat and cold exposure	793.87 (509.13 to 1151.35)	0.24 (0.15 to 0.34)
YLDs	Adverse effects of medical treatment	0.00 (0.00 to 0.00)	0.00 (0.00 to 0.00)
YLDs	Other transport injuries	1326.99 (776.44 to 2001.76)	0.40 (0.23 to 0.60)
YLDs	Fire, heat, and hot substances	171.77 (100.22 to 268.90)	0.05 (0.03 to 0.08)
YLDs	Police conflict and executions	25.07 (9.43 to 55.98)	0.01 (0.00 to 0.02)
YLDs	Conflict and terrorism	19.19 (6.91 to 43.33)	0.01 (0.00 to 0.01)

YLDs years lived with disability.

Supplementary Table 23 Predicted case number and ASR of incidence, prevalence, and YLDs of spinal cord injury among children and adolescents in China to 2045				
**Measure**	**Sex**	**Year**	**Case number**	**ASR**
Incidence	Male	2022	6480	3.97
Incidence	Male	2023	6485	4.00
Incidence	Male	2024	6483	4.03
Incidence	Male	2025	6422	4.03
Incidence	Male	2026	6358	4.03
Incidence	Male	2027	6295	4.03
Incidence	Male	2028	6223	4.03
Incidence	Male	2029	6149	4.02
Incidence	Male	2030	6083	4.03
Incidence	Male	2031	6018	4.03
Incidence	Male	2032	5958	4.03
Incidence	Male	2033	5862	4.03
Incidence	Male	2034	5751	4.03
Incidence	Male	2035	5649	4.05
Incidence	Male	2036	5512	4.06
Incidence	Male	2037	5317	4.08
Incidence	Male	2038	5190	4.09
Incidence	Male	2039	5079	4.11
Incidence	Male	2040	4982	4.12
Incidence	Male	2041	4896	4.14
Incidence	Male	2042	4819	4.15
Incidence	Male	2043	4750	4.17
Incidence	Male	2044	4690	4.19
Incidence	Male	2045	4638	4.20
Incidence	Female	2022	3362	2.46
Incidence	Female	2023	3434	2.54
Incidence	Female	2024	3503	2.62
Incidence	Female	2025	3528	2.67
Incidence	Female	2026	3551	2.72
Incidence	Female	2027	3569	2.77
Incidence	Female	2028	3580	2.82
Incidence	Female	2029	3588	2.87
Incidence	Female	2030	3583	2.90
Incidence	Female	2031	3575	2.94
Incidence	Female	2032	3563	2.98
Incidence	Female	2033	3532	3.02
Incidence	Female	2034	3493	3.06
Incidence	Female	2035	3463	3.10
Incidence	Female	2036	3418	3.14

Incidence	Female	2037	3346	3.18
Incidence	Female	2038	3307	3.22
Incidence	Female	2039	3277	3.26
Incidence	Female	2040	3257	3.30
Incidence	Female	2041	3242	3.35
Incidence	Female	2042	3232	3.39
Incidence	Female	2043	3226	3.43
Incidence	Female	2044	3223	3.48
Incidence	Female	2045	3224	3.52
Incidence	Both	2022	9758	3.25
Incidence	Both	2023	9817	3.29
Incidence	Both	2024	9870	3.34
Incidence	Both	2025	9815	3.35
Incidence	Both	2026	9757	3.37
Incidence	Both	2027	9698	3.38
Incidence	Both	2028	9624	3.39
Incidence	Both	2029	9546	3.41
Incidence	Both	2030	9467	3.42
Incidence	Both	2031	9385	3.43
Incidence	Both	2032	9306	3.44
Incidence	Both	2033	9172	3.45
Incidence	Both	2034	9015	3.47
Incidence	Both	2035	8875	3.49
Incidence	Both	2036	8684	3.51
Incidence	Both	2037	8407	3.53
Incidence	Both	2038	8230	3.55
Incidence	Both	2039	8080	3.57
Incidence	Both	2040	7950	3.59
Incidence	Both	2041	7837	3.61
Incidence	Both	2042	7738	3.63
Incidence	Both	2043	7652	3.66
Incidence	Both	2044	7577	3.68
Incidence	Both	2045	7515	3.70
Prevalence	Male	2022	65596	39.17
Prevalence	Male	2023	65945	39.43
Prevalence	Male	2024	66218	39.70
Prevalence	Male	2025	65923	39.66
Prevalence	Male	2026	65690	39.61
Prevalence	Male	2027	65629	39.57
Prevalence	Male	2028	65324	39.53
Prevalence	Male	2029	64945	39.48
Prevalence	Male	2030	64884	39.64

Prevalence	Male	2031	64780	39.80
Prevalence	Male	2032	64713	39.96
Prevalence	Male	2033	64162	40.11
Prevalence	Male	2034	63375	40.27
Prevalence	Male	2035	62204	40.40
Prevalence	Male	2036	60496	40.54
Prevalence	Male	2037	57917	40.67
Prevalence	Male	2038	56217	40.80
Prevalence	Male	2039	54711	40.93
Prevalence	Male	2040	53347	41.07
Prevalence	Male	2041	52103	41.20
Prevalence	Male	2042	50976	41.34
Prevalence	Male	2043	49959	41.47
Prevalence	Male	2044	49056	41.61
Prevalence	Male	2045	48271	41.74
Prevalence	Female	2022	39010	27.58
Prevalence	Female	2023	39468	27.97
Prevalence	Female	2024	39907	28.36
Prevalence	Female	2025	39628	28.26
Prevalence	Female	2026	39390	28.16
Prevalence	Female	2027	39237	28.06
Prevalence	Female	2028	38946	27.96
Prevalence	Female	2029	38619	27.86
Prevalence	Female	2030	38643	28.01
Prevalence	Female	2031	38622	28.16
Prevalence	Female	2032	38580	28.30
Prevalence	Female	2033	38252	28.45
Prevalence	Female	2034	37775	28.60
Prevalence	Female	2035	37153	28.76
Prevalence	Female	2036	36230	28.92
Prevalence	Female	2037	34810	29.08
Prevalence	Female	2038	33890	29.24
Prevalence	Female	2039	33081	29.41
Prevalence	Female	2040	32358	29.57
Prevalence	Female	2041	31704	29.73
Prevalence	Female	2042	31120	29.90
Prevalence	Female	2043	30599	30.06
Prevalence	Female	2044	30145	30.23
Prevalence	Female	2045	29758	30.39
Prevalence	Both	2022	104341	33.77
Prevalence	Both	2023	105123	34.09
Prevalence	Both	2024	105820	34.41

Prevalence	Both	2025	105227	34.33
Prevalence	Both	2026	104740	34.25
Prevalence	Both	2027	104510	34.17
Prevalence	Both	2028	103900	34.09
Prevalence	Both	2029	103184	34.02
Prevalence	Both	2030	103156	34.17
Prevalence	Both	2031	103038	34.32
Prevalence	Both	2032	102930	34.47
Prevalence	Both	2033	102050	34.62
Prevalence	Both	2034	100783	34.77
Prevalence	Both	2035	99000	34.91
Prevalence	Both	2036	96383	35.06
Prevalence	Both	2037	92403	35.21
Prevalence	Both	2038	89796	35.35
Prevalence	Both	2039	87493	35.50
Prevalence	Both	2040	85415	35.65
Prevalence	Both	2041	83527	35.80
Prevalence	Both	2042	81824	35.95
Prevalence	Both	2043	80296	36.10
Prevalence	Both	2044	78946	36.25
Prevalence	Both	2045	77781	36.39
YLDs	Male	2022	19122	11.43
YLDs	Male	2023	19135	11.46
YLDs	Male	2024	19126	11.49
YLDs	Male	2025	18994	11.46
YLDs	Male	2026	18875	11.42
YLDs	Male	2027	18794	11.39
YLDs	Male	2028	18644	11.36
YLDs	Male	2029	18469	11.32
YLDs	Male	2030	18445	11.38
YLDs	Male	2031	18401	11.44
YLDs	Male	2032	18352	11.50
YLDs	Male	2033	18177	11.56
YLDs	Male	2034	17939	11.62
YLDs	Male	2035	17642	11.68
YLDs	Male	2036	17204	11.75
YLDs	Male	2037	16535	11.82
YLDs	Male	2038	16099	11.88
YLDs	Male	2039	15716	11.95
YLDs	Male	2040	15374	12.02
YLDs	Male	2041	15064	12.09
YLDs	Male	2042	14788	12.16

YLDs	Male	2043	14542	12.23
YLDs	Male	2044	14327	12.29
YLDs	Male	2045	14145	12.36
YLDs	Female	2022	11028	7.81
YLDs	Female	2023	11108	7.88
YLDs	Female	2024	11182	7.96
YLDs	Female	2025	11070	7.91
YLDs	Female	2026	10968	7.86
YLDs	Female	2027	10885	7.81
YLDs	Female	2028	10765	7.76
YLDs	Female	2029	10633	7.71
YLDs	Female	2030	10620	7.74
YLDs	Female	2031	10592	7.78
YLDs	Female	2032	10554	7.81
YLDs	Female	2033	10442	7.84
YLDs	Female	2034	10292	7.87
YLDs	Female	2035	10123	7.92
YLDs	Female	2036	9877	7.97
YLDs	Female	2037	9501	8.01
YLDs	Female	2038	9257	8.06
YLDs	Female	2039	9043	8.11
YLDs	Female	2040	8852	8.16
YLDs	Female	2041	8680	8.21
YLDs	Female	2042	8528	8.25
YLDs	Female	2043	8392	8.30
YLDs	Female	2044	8275	8.35
YLDs	Female	2045	8176	8.40
YLDs	Both	2022	30045	9.73
YLDs	Both	2023	30124	9.78
YLDs	Both	2024	30179	9.82
YLDs	Both	2025	29903	9.77
YLDs	Both	2026	29654	9.71
YLDs	Both	2027	29471	9.66
YLDs	Both	2028	29182	9.60
YLDs	Both	2029	28862	9.55
YLDs	Both	2030	28802	9.58
YLDs	Both	2031	28714	9.61
YLDs	Both	2032	28620	9.64
YLDs	Both	2033	28319	9.67
YLDs	Both	2034	27915	9.70
YLDs	Both	2035	27420	9.75
YLDs	Both	2036	26702	9.79

YLDs	Both	2037	25618	9.83
YLDs	Both	2038	24906	9.87
YLDs	Both	2039	24278	9.91
YLDs	Both	2040	23712	9.96
YLDs	Both	2041	23199	10.00
YLDs	Both	2042	22738	10.04
YLDs	Both	2043	22325	10.09
YLDs	Both	2044	21961	10.13
YLDs	Both	2045	21648	10.17

YLDs years lived with disability, *ASR* age-standardized rate.
